# Optimizing construction company selection using einstein weighted aggregation operators for q-rung orthopair fuzzy hypersoft set

**DOI:** 10.1038/s41598-023-32818-8

**Published:** 2023-04-20

**Authors:** Rana Muhammad Zulqarnain, Imran Siddique, Abid Mahboob, Hijaz Ahmad, Sameh Askar, Shahid Hussain Gurmani

**Affiliations:** 1 School of Mathematical Sciences, Zhejiang Normal University, Jinhua, 321004 Zhejiang China; 2 Department of Mathematics, University of Management and Technology, Lahore, 54770 Pakistan; 3 Department of Mathematics, Division of Science and Technology, University of Education Lahore, Lahore, Pakistan; 4 Section of Mathematics, International Telematic University Uninettuno, Corso Vittorio Emanuele II, 39, 00186 Roma, Italy; 5 Operational Research Center in Healthcare, Near East Boulevard, Near East University, 99138 Mersin, Turkey; 6 Department of Statistics and Operations Research, College of Science, King Saud University, P.O. Box 2455, Riyadh, 11451 Saudi Arabia

**Keywords:** Mathematics and computing, Applied mathematics, Computational science

## Abstract

Infrastructure development and the economy heavily rely on the construction industry. However, decision-making in construction projects can be intricate and difficult due to conflicting standards and requirements. To address this challenge, the q-rung orthopair fuzzy soft set (q-ROFSS) has emerged as a useful tool incorporating fuzzy and uncertain contractions. In many cases, further characterization of attributes is necessary as their values are not mutually exclusive. The prevalent q-ROFSS structures cannot resolve this state. The q-rung orthopair fuzzy hypersoft sets (q-ROFHSS) is a leeway of q-ROFSS that use multi-parameter approximation functions to scare the scarcities of predominant fuzzy sets structures. The fundamental objective of this research is to introduce the Einstein weighted aggregation operators (AOs) for q-rung orthopair fuzzy hypersoft sets (q-ROFHSS), such as q-rung orthopair fuzzy hypersoft Einstein weighted average and geometric operators, and discuss their fundamental properties. Mathematical explanations of decision-making (DM) contractions is present to approve the rationality of the developed approach. Einstein AOs, based on predictions, carried an animated multi-criteria group decision (MCGDM) method with the most substantial significance with the prominent MCGDM structures. Moreover, we utilize our proposed MCGDM model to select the most suitable construction company for a given construction project. The proposed method is evaluated through a statistical analysis, which helps ensure the DM process's efficiency. This analysis demonstrates that the proposed method is more realistic and reliable than other DM approaches. Overall, the research provides valuable insights for decision-makers in the construction industry who seek to optimize their DM processes and improve the outcomes of their projects.

## Introduction

The construction industry is a vital sector that plays a significant role in developing infrastructure and the economy. This industry involves the construction of buildings, roads, bridges, tunnels, airports, and other structures. The construction process involves various stages, from the project's conceptualization to the final implementation. The industry comprises various professionals, including architects, engineers, project managers, construction workers, and building materials and equipment suppliers. The success of a construction project depends on the collaboration and coordination of these professionals, and effective communication is vital to ensure that the project is completed on time, within budget, and to the required standards. The construction industry faces various challenges, including adopting new technologies, rising materials, labor costs, and compliance with safety and environmental regulations. These challenges require innovative solutions, and the industry must continue to adapt to the changing landscape. Despite the challenges, the construction industry provides numerous opportunities for employment, innovation, and economic growth. It is a critical sector that plays a vital role in creating a better quality of life for people by providing them with the infrastructure they need to live and work comfortably. Selecting a construction industry is a crucial decision that requires careful consideration of various factors. One of the main hurdles in selecting the right construction industry is finding a company that can deliver the project within the required timeframe, budget, and quality standards.

MCGDM has been rated as the superlative intelligent approach to accomplish an appropriate alternative because of all the concrete expectations, criteria, or configurations that originate with it. A comprehensive judgment arises when representative objectives and limitations are often imprecise or partial. Zadeh^1^ projected the theory of fuzzy sets (FS) to demonstrate this fabricated and conflicting data. Excessive and insecure DM circumstances should be handled compactly. The FS model has been widely used in various fields. Current FS cannot handle a situation in which DM scheme professionals normally contemplate membership degrees (MD) and non-membership degrees (NMDs). Jana^2^ extended the MABAC model to resolve multi-attribute decision-making (MADM) and AOs for bipolar fuzzy numbers. Mahmood and Ali^3^ developed the fuzzy superior Mandelbrot set, the generalized form of FS and the superior Mandelbrot set. Atanassov^4^ incredulous these boundaries and proposed intuitionistic fuzzy sets (IFS). Wang and Liu^5^ delivered basic operations and AOs in their deliberated framework. Xu^6^ prolonged the IFS theory and determined the score and accuracy functions to connotation among two intuitionistic fuzzy numbers. Garg^7^ lengthened the cosine similarity measures (SMs) and used them to resolve DM hurdles. Lin et al.^8^ extended the IFS philosophy and confirmed progressive multi-criteria decision-making (MCDM) models. Mahmood et al.^9^ developed the T-spherical fuzzy set (TSFS) with its basic operations and properties. Garg et al.^10^ extended the interaction AOs for TSFS and developed a MADM model. Liu et al.^11^ prolonged novel operational laws for TSFS and proposed the Muirhead mean operators using their developed operational laws to resolve MADM complications. Ullah et al.^12^ presented the correlation coefficients for TSFS and developed a MADM approach based on their established correlation measures. De et al.^13^ resolute IFS concentration, normalization, and dilation operations. Jana and Pal^14^ developed some dynamic weighted Dombi AOs for IFS and interval-valued IFS and established a dynamic hybrid MADM model. The IFS cannot detain the unsteady and baffling details, as it visualizes a straight indiscretion between $\mathrm{MD}$ and $\mathrm{NMD}$. If the board chooses MD and NMD, such as $MD+NMD>1$, present IFS models flop to contract with this consequence.

Yager^15^ proposed that the Pythagorean fuzzy set (PFS) insists on this deficiency by modifying the fundamental states $\mathrm{f}+\mathrm{g }\le 1$ to ${\mathrm{f}}^{2}+{\mathrm{g}}^{2}\le 1$. Xiao and Ding^16^ presented the divergence measures for PFS and used their developed measures for medical diagnosis. Thao and Smarandache^17^ established an MCDM scheme built on entropy measures under the PFS setting. Zhang et al.^18^ introduced novel SMs for PFS and proved they are proficiently equated to prevalent SMs. Rahman et al.^19^ prolonged the multi-attribute group decision-making (MAGDM) model using Einstein weighted geometric operator on PFS. Zhang and Xu^20^ extended the TOPSIS method to remove MCDM constraints in PFS. Jana et al.^21^ prolonged the power dombi AOs for PFS and settled a MADM approach to determine real-life hurdles. Wei and Lu^22^ developed the power AO for PFS with its important belongings. Garg et al.^23^ developed the hammy mean AOs for complex PFS and established the TOPSIS scheme to resolve MADM hurdles. Wang and Li^24^ prolonged the Bonferroni mean AOs for PFS considering the interaction among Pythagorean fuzzy numbers (PFN). Liu et al.^25^ proposed the confidence complex Pythagorean fuzzy Archimedean AOs and established a novel MADM technique based on their presented operators. Zhang^26^ planned a radical DM technique using SMs to solve the problem of MCGDM under PFS configuration. Yager^27^ established a generalized theory of IFS and PFS, known as a q-rung orthopair fuzzy set (q-ROFS). He developed numerous necessary operations of q-ROFS and discussed their desirable belongings. The above structures have a wide range of demonstrations, but all the above structures cannot handle the alternative parameters. Liu et al.^28^ presented the Einstein interaction geometric AOs for complex q-ROFS with their desirable properties. Ali and Mahmood^29^ prolonged the Dombi AOs for complex q-ROFS and established an MADM model to resolve DM complications.

Molodtsov^30^ proposed the soft set (SS) philosophy to contract with the parametric standards of the alternates. Maji et al.^31^ introduced several fundamental operations for SS and discussed their significant properties. Cagman and Enginoglu^32^ extended the SS model to fuzzy parametrized SS with some important tasks. They also protracted the DM methodology to validate their established theory. Ali et al.^33^ introduced several fundamental operations for SS. Maji et al.^34^ fused two eminent models, FS and SS, and offered the fuzzy soft set (FSS) theory. Roy and Maji^35^ elongated a theoretical DM tool for FSS to contract with obscure and invalid information. Maji et al.^36^ developed the intuitionistic FSS (IFSS) with its complementary properties. Arora and Garg^37^ planned an MCDM technique for IFSS to resolve DM complications using their developed AOs. Çağman and Karataş^38^ prolonged the idea of IFSS and debated its elementary operations with a DM model to resolve real-life complications. Muthukumar and Krishnan^39^ proposed some novel SMs with important properties for IFSS. Peng et al.^40^ constructed the Pythagorean fuzzy soft set (PFSS) with a mixture of PFS and SS. Athira et al.^41,42^ protracted the idea of PFSS and introduced entropy and distance measures. Zulqarnain et al.^43–45^ offered the Einstein operational laws and prolonged the Einstein-weighted and Einstein-ordered weighted AOs under PFSS with their DM approaches. Hussain et al.^46^ expanded the PFSS to a q-ROFSS and developed the AOs based on algebraic operational laws. Zulqarnain et al.^47,48^ protracted the Einstein AOs for q-ROFSS and established the DM methodologies based on their developed operators.

The models with SS configuration compact with single-parameter estimation functions, although hypersoft sets (HSS), a leeway of SS, and contract with multi-parameter approximation. The SS cannot grip states wherever parameters must be divided into further sub-attributes. In voluminous DM states, impost parameters must be characterized into sub-parameters. To overcome such complications, Smarandache^49^ extended the SS to the hypersoft set (HSS), the most generalized model to handle the sub-parameters of the deliberated parameters. Rahman et al.^50^ developed the SMs for the possibility intuitionistic fuzzy hypersoft set (IFHSS). Zulqarnain et al.^51^ presented the AOs for IFHSS engaging their raised algebraic operational laws. They also introduced the Pythagorean fuzzy hypersoft set (PFHSS)^52^ and discussed their significant properties. Siddique et al.^53^ delivered a creative MCDM system for PFHSS using their developed AOs. Sunthrayuth et al.^54^ and Zulqarnain et al.^55^ predicted the Einstein AOs for PFHSS to obstinacy MCDM impediments and used them for ari-farming and material selection consistently. Zulqarnain et al.^56^ developed the Einstein-ordered AOs for PFHSS and assembled an MCDM approach to resolve DM complexities. Khan et al.^57^ extended the q-ROFSS to q-ROFHSS and introduced several fundamental operations. Gurmani et al.^58^ protracted the TOPSIS technique to q-ROFHSS built on correlation coefficient (CC). Khan et al.^59^ offered the operational laws for q-ROFHSS and developed the AOs. They also built a DM methodology using their offered AOs and utilized it in the cryptocurrency market. Zulqarnain et al.^60^ pushed the interaction AOs of q-ROFHSS to cryptocurrency analysis. A better-integrated organization fascinates detectives with inadequate, incredible, and irregular facts to debate these flaws. They explained the importance of deliberation, q-ROFHSS spectacles a robust portion in DM by accumulating affluent cradles in a specific judgment.

### Motivation and drawback of existing approaches

The predominant Einstein-weighted AOs for PFHSS^54,55^ only assess PFHSS impacts and only deliberate the PFHSS approximations, not the q-ROFHSS impacts. Also, from the above AOs for q-ROFHSS^59^, it is stated that, in confident surroundings, these AOs deliver some disgusting consequences. To confine these deficiencies, we will bargain Einstein operational laws for q-ROFHSS. The q-ROFHSS is a mixed rational structure of HSS and q-ROFS, the basic mathematical tool for dealing with hesitancies, discrepancy, and imperfect details. AOs perform a vital role in DM, so facts of communal judgments from various causes can be inscribed into distinctive assessments. Einstein's operational laws have no application in literature with the hybridization of HSS and q-ROFS. So, the prevalent method has not quantitatively concise q-rung orthopair fuzzy hypersoft numbers (q-ROFHSNs) nor deliberately correlated with $\mathrm{MD}$ and $\mathrm{NMD}$. The effect of $MD (NMD)$ on the subsequent AOs did not interfere with the procedure. Furthermore, the model ranks the whole level of the $MD(NMD)$ function as independent of the level of the $NMD (MD)$ function. Therefore, by giving these AOs, the outcomes are obstructive, and consequently, the applicable partiality for alternatives is not determined. Therefore, how to incorporate these q-ROFHSNs for Einstein operational laws is a well-designed query. To resolve such queries, we will introduce q-ROFHSEWA and q- ROFHSEWG operators for q-ROFHSS. The prevalent Einstein-weighted AOs become the special cases of q-ROFHSS. So, it can be determined that the proposed model is more competent than existing Einstein-weighted AOs. Thus, the consequence of the prevalent models is adverse, and the favoritism of the alternative cannot be configured appropriately. Therefore, incorporating these q-ROFHSNs into Einstein's specification is an exciting subject. The methodologies labeled in^59^ are inadequate to check the facts on flexible perspectives to accomplish well thoughts and specific outcomes. For example, we consider the set of two experts such as $\mathcal{H}=\left\{{\mathcal{H}}_{1}, {\mathcal{H}}_{2}\right\}$ whose weights are given as ${\uptheta }_{\mathrm{i}}={\left(.7, .3\right)}^{\mathrm{T}}$, also ${\mathrm{d}}_{1}, {\mathrm{d}}_{2}$ be two considered parameters. Let ${\mathrm{d}}_{1}=\left\{{\mathrm{d}}_{11}, {\mathrm{d}}_{12}\right\}$ and ${\mathrm{d}}_{2}=\left\{{\mathrm{d}}_{21}\right\}$ be the conforming sub-parameters of the deliberated parameters. It can be identified as ${\mathfrak{L}}^{{^{\prime}}}={\mathrm{d}}_{1}\times {\mathrm{d}}_{2}=\left\{{\mathrm{d}}_{11}, {\mathrm{d}}_{12}\right\}\times \left\{{\mathrm{d}}_{21}\right\}=$ $\left\{\left({\mathrm{d}}_{11}, {\mathrm{d}}_{21}\right), \left({\mathrm{d}}_{12}, {\mathrm{d}}_{21}\right)\right\}=\left\{{\hat{\mathrm{d}}}_{1}, {\hat{\mathrm{d}}}_{2}\right\}$ with weights ${\upomega }_{\mathrm{j}}={\left(0.4, .0.6\right)}^{\mathrm{T}}$ and $\mathrm{\aleph }$ be an alternate. The preferences of the experts can be precise as $$\mathrm{\aleph }=\left[\begin{array}{cc}\left(0.7, 0.0\right)& \left(0.6, 0.7\right)\\ \left(0.8, 0.7\right)& \left(0.7, 0.2\right)\end{array}\right]$$ in q-ROFHSNs form. So, we conquered the $$\langle \begin{array}{c}0.6819, 0\end{array}.0\rangle$$ and $$\langle \begin{array}{c}0.6667, 0.0\end{array}\rangle$$ collective values using q-ROFHSWA^59^ operator. The above outcomes show that there is no impact on the collective consequence ${\mathrm{g}}_{{\hat{\mathrm{d}}}_{\mathrm{k}}}$. Meanwhile ${\mathrm{g}}_{{\hat{\mathrm{d}}}_{11}}$ = 0.0, ${\mathrm{g}}_{{\hat{\mathrm{d}}}_{12}}$ = 0.7, ${\mathrm{g}}_{{\hat{\mathrm{d}}}_{21}}$ = 0.7, and ${\mathrm{g}}_{{\hat{\mathrm{d}}}_{22}}$ = 0.2, which is unreasonable. The existing Einstein AOs^54,55^ for PFHSS cannot handle the abovementioned problem. Because ${\mathrm{f}}_{{\hat{\mathrm{d}}}_{21}}$ = 0.8 and ${\mathrm{g}}_{{\hat{\mathrm{d}}}_{21}}$ = 0.7, where ${\left(0.8\right)}^{2}+{\left(0.7\right)}^{2}>1$. So, the existing Einstein-ordered weighted AOs of PFHSS cannot deal with such scenarios. To overcome these deficiencies, we will propose an improved organizing methodology considering the Einstein operational laws under the q-ROFHSS setting to attract researchers to smash inexplicable and deficient information. Deducing the investigation effects, q-ROFHSS is active in DM by accumulating numerous structures into a specific value.

### Contribution

Einstein's weighted AOs are sure to fascinate the assessed AOs. It has been perceived that the general AOs feature does not respond to the finding of direct effects by the DM scheme under apparent conditions. These AOs need to be reformed to eliminate these thorny problems. Therefore, to irradiate the current study of q-ROFHSS and the above limitations, we will assign Einstein-weighted AOs founded on uncertain facts, with the primary purpose of the research given as:

1. The Einstein-weighted AOs under q-ROFHSS settings are acquainted with attractive estimation AOs. It is believed that in some states, the main conceptual feature is the lack of sympathetic labeling of particular consequences of the DM process. To surprise such rigorous impairments, we prolonged the idea of q-ROFHSS and extended some novel AOs for q-ROFHSS considering the Einstein operational laws.
2. The q-ROFHSS is a model designed to clarify the responsibility of the multiple sub-attributes of intellectual aspects in DM structures. This model provides a comprehensive framework for understanding how different sub-attributes of intellectual aspects, such as rationality, creativity, and intuition, can influence DM processes. To ensure that the support provided by the q-ROFHSS is preserved, it is essential to represent it accurately. For this reason, we strongly recommend using Einstein's weighted AOs to portray the q-ROFHSS. These AOs are specifically designed to account for the uncertain nature of the sub-attributes of intellectual aspects that influence DM processes.
3. Introduce the q-ROFHSEWA and q-ROFHSEWG operators, which are two mathematical operators to improve our understanding of DM processes. The q-ROFHSEWA operator captures the weight of each sub-attribute of intellectual aspects, while the q-ROFHSEWG operator incorporates Einstein's weighted AOs to better represent uncertainty. Both operators have specific properties that make them useful in different scenarios and can be used to develop more accurate models for decision-making.
4. To ensure the practicality of our proposed Einstein-weighted AOs, a novel DM model is established to integrate MCGDM anxieties into the q-ROFHSS setting to assert DM negligence. Moreover, it is used in construction projects to choose the most suitable company.
5. A comprehensive analysis of the advanced MCGDM methodology and predominant approaches is performed to confirm the validity and excellence of the intentional MCGDM approach.

The correspondence prospects in this research are as follows: in “Preliminaries” section deals with some of the fundamental notions that sustain our structure of follow-up exploration. In “Einstein weighted average aggregation operator for q-rung orthopair fuzzy hypersoft set” section proposes Einstein's operational laws for q-ROFHSN. Also, the q-ROFHSEWA is introduced in the same section with some significant results and properties. The q-ROFHSEWG operator with its necessary possessions is offered in “Einstein weighted geometric aggregation operator for q-rung orthopair fuzzy hypersoft set” section. The MCGDM scheme in “MCGDM Model under q-ROFHSS Information” section is built using the projected AOs. Moreover, we employed the developed MCGDM model to select the most appropriate construction company. Also, brief sensitivity analysis and comparative studies are complemented to distinguish the facts of the established structure in “Sensitivity analysis and comparative studies” section.

## Preliminaries

This section recalls compulsory notions such as SS, HSS, PFHSS, and q-ROFHSS.

### Definition^30^

Let $\mathcal{U}$ be a universe of discourse and $\mathcal{E}$ be the set of attributes. Suppose $\mathcal{P}(\mathcal{U})$ be the power set of $\mathcal{U}$ and $\mathcal{A}$ is any subset of attributes. Then, a pair $\left(\mathcal{F},\mathcal{A}\right)$ is named as a soft set over $\mathcal{U}$, and its mapping is defined as:

$$\mathcal{F}:\mathcal{A} \to \mathcal{P}(\mathcal{U})$$

### Definition^49^

Let $\mathcal{U}$ be a universe of discourse and $\mathcal{P}\left(\mathcal{U}\right)$ be a power set of $\mathcal{U}$ and $\mathrm{k}=\left\{{\mathrm{k}}_{1}, {\mathrm{k}}_{2}, {\mathrm{k}}_{3},..., {\mathrm{k}}_{\mathrm{n}}\right\}, \left(\mathrm{n }\ge 1\right)$ and ${\mathrm{K}}_{\mathrm{i}}$ showed the set of parameters and their correspondent sub-parameters, such as ${\mathrm{K}}_{\mathrm{i}}\cap {\mathrm{K}}_{\mathrm{j}} =\mathrm{ \varphi }$, where $\mathrm{i }\ne \mathrm{ j}$ for each $\mathrm{n }\ge 1$ and $\mathrm{i},\mathrm{ j \epsilon }\{\mathrm{1,2},3 \dots \mathrm{ n}\}$. Assume ${\mathrm{K}}_{1} \times {\mathrm{K}}_{2} \times {\mathrm{K}}_{3}\times \dots \times {\mathrm{K}}_{\mathrm{n}} = \mathop{{\mathcal{A}}}\limits^{\ldots} = \left\{{\mathrm{d}}_{1\mathrm{h}}\times {\mathrm{d}}_{2\mathrm{k}}\times \cdots \times {\mathrm{d}}_{\mathrm{nl}}\right\}$ is an collection of sub-attributes, where $1\le \mathrm{h}$ $\le$ $\mathrm{\alpha }$, $1\le \mathrm{k}$ $\le$ $\upbeta$, and $1\le \mathrm{l}\le\upgamma$, and $\mathrm{\alpha },\upbeta ,\upgamma \in {\mathbb{N}}$. Then the pair $\left(\mathcal{F}, {\mathrm{K}}_{1} \times {\mathrm{K}}_{2} \times {\mathrm{K}}_{3}\times \dots \times {\mathrm{K}}_{\mathrm{n}}\right)=\left(\mathcal{F}, \mathop{{\mathcal{A}}}\limits^{\ldots}\right)$ is called HSS and is defined as:

$$\mathcal{F}:{\mathrm{K}}_{1}\times {\mathrm{K}}_{2} \times {\mathrm{K}}_{3}\times \dots \times {\mathrm{K}}_{\mathrm{n}} = \mathop{{\mathcal{A}}}\limits^{\ldots} \to \mathcal{P}\left(\mathcal{U}\right).$$

Also, it can be defined as:

$$\left(\mathcal{F}, \mathop{{\mathcal{A}}}\limits^{\ldots}\right)=\left\{\hat{\mathrm{d}}, {\mathcal{F}}_{\mathop{{\mathcal{A}}}\limits^{\ldots}}\left(\hat{\mathrm{d}}\right): \hat{\mathrm{d}}\in \mathop{{\mathcal{A}}}\limits^{\ldots}, {\mathcal{F}}_{\mathop{{\mathcal{A}}}\limits^{\ldots}}\left(\hat{\mathrm{d}}\right)\in \mathcal{P}(\mathcal{U})\right\}$$

### Definition^52^

Let $\mathcal{U}$ be a universe of discourse and $\mathcal{P}$($\mathcal{U}$) be a power set of $\mathcal{U}$ and $\mathrm{k}=\left\{{\mathrm{k}}_{1}, {\mathrm{k}}_{2}, {\mathrm{k}}_{3},..., {\mathrm{k}}_{\mathrm{n}}\right\}, \left(\mathrm{n }\ge 1\right)$ and ${\mathrm{K}}_{\mathrm{i}}$ showed the set of parameters and their correspondent sub-parameters, such as ${\mathrm{K}}_{\mathrm{i}}\cap {\mathrm{K}}_{\mathrm{j}} =\mathrm{ \varphi }$, where $\mathrm{i }\ne \mathrm{ j}$ for each $\mathrm{n }\ge 1$ and $\mathrm{i},\mathrm{ j \epsilon }\{\mathrm{1,2},3 \dots \mathrm{ n}\}$. Assume ${\mathrm{K}}_{1} \times {\mathrm{K}}_{2} \times {\mathrm{K}}_{3}\times \dots \times {\mathrm{K}}_{\mathrm{n}} = \mathop{{\mathcal{A}}}\limits^{\ldots} = \left\{{\mathrm{d}}_{1\mathrm{h}}\times {\mathrm{d}}_{2\mathrm{k}}\times \cdots \times {\mathrm{d}}_{\mathrm{nl}}\right\}$ is an collection of sub-parameters, where $1\le \mathrm{h}\le \mathrm{\alpha }$, $1\le \mathrm{k}\le\upbeta$, and $1\le \mathrm{l}\le\upgamma$, and $\mathrm{\alpha },\upbeta ,\upgamma \in {\mathbb{N}}$, and ${\mathrm{PFS}}^{\mathcal{U}}$ represents the collection of all subsets of Pythagorean fuzzy hyper subsets over $\mathcal{U}$. Then $\left(\mathcal{F}, {\mathrm{K}}_{1} \times {\mathrm{K}}_{2} \times {\mathrm{K}}_{3}\times \dots \times {\mathrm{K}}_{\mathrm{n}}\right)=\left(\mathcal{F}, \mathop{{\mathcal{A}}}\limits^{\ldots}\right)$ is called PFHSS and can be defined as:

$$\mathcal{F}:{\mathrm{K}}_{1} \times {\mathrm{K}}_{2} \times {\mathrm{K}}_{3}\times \dots \times {\mathrm{K}}_{\mathrm{n}}=\mathop{{\mathcal{A}}}\limits^{\ldots}\to {\mathrm{PFHS}}^{\mathcal{U}}.$$

Also, it can be defined as:

$\left(\mathcal{F},\mathop{{\mathcal{A}}}\limits^{\ldots}\right)=\left\{\left(\hat{\mathrm{d}}, {\mathcal{F}}_{\mathop{{\mathcal{A}}}\limits^{\ldots}}\left(\hat{\mathrm{d}}\right)\right): \hat{\mathrm{d}}\in \mathop{{\mathcal{A}}}\limits^{\ldots}, {\mathcal{F}}_{\mathop{{\mathcal{A}}}\limits^{\ldots}}\left(\hat{\mathrm{d}}\right) \in \mathrm{ PFH}{\mathrm{S}}^{\mathcal{U}}\in \left[0, 1\right]\right\}$, where ${\mathcal{F}}_{\mathop{{\mathcal{A}}}\limits^{\ldots}}\left(\hat{\mathrm{d}}\right)=\left\{ \Bigg\langle \updelta , {\mathrm{f}}_{{\hat{\mathrm{d}}}_{\mathrm{ij}}}\left(\updelta \right), {\mathrm{g}}_{{\hat{\mathrm{d}}}_{\mathrm{ij}}}\left(\updelta \right)\Bigg\rangle :\updelta \in \mathcal{U}\right\}$, where ${\mathrm{f}}_{{\hat{\mathrm{d}}}_{\mathrm{ij}}}\left(\updelta \right)$ and ${\mathrm{g}}_{{\hat{\mathrm{d}}}_{\mathrm{ij}}}\left(\updelta \right)$ shows the MD and NMD. ${\mathrm{f}}_{{\hat{\mathrm{d}}}_{\mathrm{ij}}}\left(\updelta \right),{\mathrm{g}}_{{\hat{\mathrm{d}}}_{\mathrm{ij}}}\left(\updelta \right)\in \left[0, 1\right]$, and $0\le {\left({\mathrm{f}}_{{\hat{\mathrm{d}}}_{\mathrm{ij}}}\left(\updelta \right)\right)}^{2}+{\left({\mathrm{g}}_{{\hat{\mathrm{d}}}_{\mathrm{ij}}}\left(\updelta \right)\right)}^{2}\le 1$.

For readers' aptness, the PFHSN ${\mathcal{F}}_{\mathop{{\mathcal{A}}}\limits^{\ldots}}\left(\hat{\mathrm{d}}\right)=\left\{\Bigg\langle \updelta , {\mathrm{f}}_{\mathcal{F}\left(\hat{\mathrm{d}}\right)}\left(\updelta \right), {\mathrm{g}}_{\mathcal{F}\left(\hat{\mathrm{d}}\right)}\left(\updelta \right)\Bigg\rangle :\updelta \in \mathcal{U}\right\}$, can be engraved as ${\mathrm{J}}_{{\hat{\mathrm{d}}}_{\mathrm{ij}}}=\langle {\mathrm{f}}_{{\hat{\mathrm{d}}}_{\mathrm{ij}}}\left(\updelta \right), {\mathrm{g}}_{{\hat{\mathrm{d}}}_{\mathrm{ij}}}\left(\updelta \right)\rangle$. The score function^53^ for ${\mathrm{J}}_{{\hat{\mathrm{d}}}_{\mathrm{ij}}}$ is stated as:

$$\mathrm{S}\left({\mathrm{J}}_{{\hat{\mathrm{d}}}_{\mathrm{ij}}}\right)={{\mathrm{f}}_{{\hat{\mathrm{d}}}_{\mathrm{ij}}}}^{2}-{{\mathrm{g}}_{{\hat{\mathrm{d}}}_{\mathrm{ij}}}}^{2}, \mathrm{S}({\mathrm{J}}_{{\hat{\mathrm{d}}}_{\mathrm{ij}}})\in \left[-1, 1\right]$$

While occasionally, the scoring function does not deliver an appropriate result for calculating PFHSNs. It is challenging to draw conclusions about which alternative is informal. To scare these barriers, accuracy functions have been acknowledged.

$$\mathrm{A}\left({\mathrm{J}}_{{\hat{\mathrm{d}}}_{\mathrm{ij}}}\right)={\left({\mathrm{f}}_{{\hat{\mathrm{d}}}_{\mathrm{ij}}}\left(\updelta \right)\right)}^{2}+{\left({\mathrm{g}}_{{\hat{\mathrm{d}}}_{\mathrm{ij}}}\left(\updelta \right)\right)}^{2},\mathrm{A}\left({\mathrm{J}}_{{\hat{\mathrm{d}}}_{\mathrm{ij}}}\right)\in \left[-1, 1\right].$$

To compare the two PFHSNs ${\mathrm{J}}_{{\hat{\mathrm{d}}}_{\mathrm{ij}}}$ and ${\mathfrak{T}}_{{\hat{\mathrm{d}}}_{\mathrm{ij}}}$ comparison rules are given as:

1. If $$\mathrm{S}\left({\mathrm{J}}_{{\hat{\mathrm{d}}}_{\mathrm{ij}}}\right)>\mathrm{S}\left({\mathfrak{T}}_{{\hat{\mathrm{d}}}_{\mathrm{ij}}}\right)$$, then $${\mathrm{J}}_{{\hat{\mathrm{d}}}_{\mathrm{ij}}}>{\mathfrak{T}}_{{\hat{\mathrm{d}}}_{\mathrm{ij}}}$$.
2. If $$\mathrm{S}\left({\mathrm{J}}_{{\hat{\mathrm{d}}}_{\mathrm{ij}}}\right)=\mathrm{S}\left({\mathfrak{T}}_{{\hat{\mathrm{d}}}_{\mathrm{ij}}}\right)$$, then
  If $$\mathrm{A}\left({\mathrm{J}}_{{\hat{\mathrm{d}}}_{\mathrm{ij}}}\right)>\mathrm{A}\left({\mathfrak{T}}_{{\hat{\mathrm{d}}}_{\mathrm{ij}}}\right)$$, then $${\mathrm{J}}_{{\hat{\mathrm{d}}}_{\mathrm{ij}}}>{\mathfrak{T}}_{{\hat{\mathrm{d}}}_{\mathrm{ij}}}$$
  If $$\mathrm{A}\left({\mathrm{J}}_{{\hat{\mathrm{d}}}_{\mathrm{ij}}}\right)=\mathrm{A}\left({\mathfrak{T}}_{{\hat{\mathrm{d}}}_{\mathrm{ij}}}\right)$$, then $${\mathrm{J}}_{{\hat{\mathrm{d}}}_{\mathrm{ij}}}$$ $$=$$ $${\mathfrak{T}}_{{\hat{\mathrm{d}}}_{\mathrm{ij}}}$$.

### Definition^54^

Let ${\mathrm{J}}_{{\hat{\mathrm{d}}}_{\mathrm{k}}}=\left({\mathrm{f}}_{{\hat{\mathrm{d}}}_{\mathrm{k}}}, {\mathrm{g}}_{{\hat{\mathrm{d}}}_{\mathrm{k}}}\right)$, ${\mathrm{J}}_{{\hat{\mathrm{d}}}_{11}}=\left({\mathrm{f}}_{{\hat{\mathrm{d}}}_{11}}, {\mathrm{g}}_{{\hat{\mathrm{d}}}_{11}}\right)$, and ${\mathrm{J}}_{{\hat{\mathrm{d}}}_{12}}=\left({\mathrm{f}}_{{\hat{\mathrm{d}}}_{12}}, {\mathrm{g}}_{{\hat{\mathrm{d}}}_{12}}\right)$ denotes the PFHSNs, and $\upgamma >0$. Then, the Einstein operational laws for PFHSNs are given as:

1. $${\mathrm{J}}_{{\hat{\mathrm{d}}}_{11}}{\oplus}_{\upvarepsilon }{\mathrm{J}}_{{\hat{\mathrm{d}}}_{12}}=\Bigg\langle \frac{\sqrt{\left(1+{\mathrm{f}}_{{\hat{\mathrm{d}}}_{11}}^{2}\right)- \left(1-{\mathrm{f}}_{{\hat{\mathrm{d}}}_{12}}^{2}\right)}}{\sqrt{\left(1+{\mathrm{f}}_{{\hat{\mathrm{d}}}_{11}}^{2}\right)+\left(1-{\mathrm{f}}_{{\hat{\mathrm{d}}}_{12}}^{2}\right)}}, \frac{\sqrt{2{\mathrm{g}}_{{\hat{\mathrm{d}}}_{12}}^{2}}}{\sqrt{\left(2-{\mathrm{g}}_{{\hat{\mathrm{d}}}_{11}}^{2}\right)+{\mathrm{g}}_{{\hat{\mathrm{d}}}_{12}}^{2}}}\Bigg\rangle$$
2. $${\mathrm{J}}_{{\hat{\mathrm{d}}}_{11}}{\otimes}_{\upvarepsilon } {\mathrm{J}}_{{\hat{\mathrm{d}}}_{12}}= \Bigg\langle \frac{\sqrt{2{\mathrm{f}}_{{\hat{\mathrm{d}}}_{11}}^{2}}}{\sqrt{\left(2-{\mathrm{f}}_{{\hat{\mathrm{d}}}_{11}}^{2}\right)+{\mathrm{f}}_{{\hat{\mathrm{d}}}_{12}}^{2}}} , \frac{\sqrt{\left(1+{\mathrm{g}}_{{\hat{\mathrm{d}}}_{11}}^{2}\right)-\left(1-{\mathrm{g}}_{{\hat{\mathrm{d}}}_{12}}^{2}\right)}}{\sqrt{\left(1+{\mathrm{g}}_{{\hat{\mathrm{d}}}_{11}}^{2}\right)+\left(1-{\mathrm{g}}_{{\hat{\mathrm{d}}}_{12}}^{2}\right)}}\Bigg\rangle$$
3. $$\upgamma {\mathrm{J}}_{{\hat{\mathrm{d}}}_{\mathrm{k}}}=\Bigg\langle \frac{\sqrt{{\left(1+{{\mathrm{f}}_{{\hat{\mathrm{d}}}_{\mathrm{k}}}}^{2}\right)}^{\upgamma }-{\left(1-{{\mathrm{f}}_{{\hat{\mathrm{d}}}_{\mathrm{k}}}}^{2}\right)}^{\upgamma }}}{\sqrt{{\left(1+{{\mathrm{f}}_{{\hat{\mathrm{d}}}_{\mathrm{k}}}}^{2}\right)}^{\upgamma }+{\left(1-{{\mathrm{f}}_{{\hat{\mathrm{d}}}_{\mathrm{k}}}}^{2}\right)}^{\upgamma }}} , \frac{\sqrt{2{\left({{\mathrm{g}}_{{\hat{\mathrm{d}}}_{\mathrm{k}}}}^{2}\right)}^{\upgamma }}}{\sqrt{{\left(2-{{\mathrm{g}}_{{\hat{\mathrm{d}}}_{\mathrm{k}}}}^{2}\right)}^{\upgamma }+{\left({{\mathrm{g}}_{{\hat{\mathrm{d}}}_{\mathrm{k}}}}^{2}\right)}^{\upgamma }}}\Bigg\rangle$$
4. $${{\mathrm{J}}_{{\hat{\mathrm{d}}}_{\mathrm{k}}}}^{\upgamma }=\Bigg\langle \frac{\sqrt{2{\left({{\mathrm{f}}_{{\hat{\mathrm{d}}}_{\mathrm{k}}}}^{2}\right)}^{\upgamma }}}{\sqrt{{\left(2-{{\mathrm{f}}_{{\hat{\mathrm{d}}}_{\mathrm{k}}}}^{2}\right)}^{\upgamma }+{\left({{\mathrm{f}}_{{\hat{\mathrm{d}}}_{\mathrm{k}}}}^{2}\right)}^{\upgamma }}} , \frac{\sqrt{{\left(1+{{\mathrm{g}}_{{\hat{\mathrm{d}}}_{\mathrm{k}}}}^{2}\right)}^{\upgamma }-{\left(1-{{\mathrm{g}}_{{\hat{\mathrm{d}}}_{\mathrm{k}}}}^{2}\right)}^{\upgamma }}}{\sqrt{{\left(1+{{\mathrm{g}}_{{\hat{\mathrm{d}}}_{\mathrm{k}}}}^{2}\right)}^{\upgamma }+{\left(1-{{\mathrm{g}}_{{\hat{\mathrm{d}}}_{\mathrm{k}}}}^{2}\right)}^{\upgamma }}}\Bigg\rangle$$.

Sunthrayuth et al.^54^ and Zulqarnain et al.^55^ defined the Einstein weighted AOs for PFHSNs by above deliberated Einstein operational laws with confident environments ${\uptheta }_{\mathrm{i}}>0,\sum_{\mathrm{i}=1}^{\mathrm{n}}{\uptheta }_{\mathrm{i}}=1; {\upomega }_{\mathrm{j}}>0, \sum_{\mathrm{j}=1}^{\mathrm{m}}{\upomega }_{\mathrm{j}}=1$.

1 $$\mathrm{PFHSEWA}\left({\mathrm{J}}_{{\hat{\mathrm{d}}}_{11}}, {\mathrm{J}}_{{\hat{\mathrm{d}}}_{12}},\dots , {\mathrm{J}}_{{\hat{\mathrm{d}}}_{\mathrm{nm}}}\right)=\Bigg\langle \begin{array}{c}\frac{\sqrt{\prod_{\mathrm{j}=1}^{\mathrm{m}}{\left({\prod }_{\mathrm{i}=1}^{\mathrm{n}}{\left(1+{\mathrm{f}}_{{\hat{\mathrm{d}}}_{\mathrm{ ij}}}^{2}\right)}^{{\uptheta }_{\mathrm{i}}}\right)}^{{\upomega }_{\mathrm{j}}}-\prod_{\mathrm{j}=1}^{\mathrm{m}}{\left({\prod }_{\mathrm{i}=1}^{\mathrm{n}}{\left(1-{\mathrm{f}}_{{\hat{\mathrm{d}}}_{\mathrm{ ij}}}^{2}\right)}^{{\uptheta }_{\mathrm{i}}}\right)}^{{\upomega }_{\mathrm{j}}}}}{\sqrt{\prod_{\mathrm{j}=1}^{\mathrm{m}}{\left({\prod }_{\mathrm{i}=1}^{\mathrm{n}}{\left(1+{\mathrm{f}}_{{\hat{\mathrm{d}}}_{\mathrm{ ij}}}^{2}\right)}^{{\uptheta }_{\mathrm{i}}}\right)}^{{\upomega }_{\mathrm{j}}}+ \prod_{\mathrm{j}=1}^{\mathrm{m}}{\left({\prod }_{\mathrm{i}=1}^{\mathrm{n}}{\left(1-{\mathrm{f}}_{{\hat{\mathrm{d}}}_{\mathrm{ ij}}}^{2}\right)}^{{\uptheta }_{\mathrm{i}}}\right)}^{{\upomega }_{\mathrm{j}}}}},\\ \frac{\sqrt{2\prod_{\mathrm{j}=1}^{\mathrm{m}}{\left({\prod }_{\mathrm{i}=1}^{\mathrm{n}}{\left({\mathrm{g}}_{{\hat{\mathrm{d}}}_{\mathrm{ ij}}}^{2}\right)}^{{\uptheta }_{\mathrm{i}}}\right)}^{{\upomega }_{\mathrm{j}}}}}{\sqrt{\prod_{\mathrm{j}=1}^{\mathrm{m}}{\left(\prod_{\mathrm{i}=1}^{\mathrm{n}}{\left(2-{\mathrm{g}}_{{\hat{\mathrm{d}}}_{\mathrm{ ij}}}^{2}\right)}^{{\uptheta }_{\mathrm{i}}}\right)}^{{\upomega }_{\mathrm{j}}}+\prod_{\mathrm{j}=1}^{\mathrm{m}}{\left({\prod }_{\mathrm{i}=1}^{\mathrm{n}}{\left({\mathrm{g}}_{{\hat{\mathrm{d}}}_{\mathrm{ ij}}}^{2}\right)}^{{\uptheta }_{\mathrm{i}}}\right)}^{{\upomega }_{\mathrm{j}}}}}\end{array}\Bigg\rangle$$

2 $$\mathrm{PFHSEWG}\left({\mathrm{J}}_{{\hat{\mathrm{d}}}_{11}}, {\mathrm{J}}_{{\hat{\mathrm{d}}}_{12}},\dots , {\mathrm{J}}_{{\hat{\mathrm{d}}}_{\mathrm{nm}}}\right)=\Bigg\langle \begin{array}{c}\frac{\sqrt{2\prod_{\mathrm{j}=1}^{\mathrm{m}}{\left({\prod }_{\mathrm{i}=1}^{\mathrm{n}}{\left({\mathrm{f}}_{{\hat{\mathrm{d}}}_{\mathrm{ ij}}}^{2}\right)}^{{\uptheta }_{\mathrm{i}}}\right)}^{{\upomega }_{\mathrm{j}}}}}{\sqrt{\prod_{\mathrm{j}=1}^{\mathrm{m}}{\left(\prod_{\mathrm{i}=1}^{\mathrm{n}}{\left(2-{\mathrm{f}}_{{\hat{\mathrm{d}}}_{\mathrm{ ij}}}^{2}\right)}^{{\uptheta }_{\mathrm{i}}}\right)}^{{\upomega }_{\mathrm{j}}}+\prod_{\mathrm{j}=1}^{\mathrm{m}}{\left({\prod }_{\mathrm{i}=1}^{\mathrm{n}}{\left({\mathrm{f}}_{{\hat{\mathrm{d}}}_{\mathrm{ ij}}}^{2}\right)}^{{\uptheta }_{\mathrm{i}}}\right)}^{{\upomega }_{\mathrm{j}}}}},\\ \frac{\sqrt{\prod_{\mathrm{j}=1}^{\mathrm{m}}{\left({\prod }_{\mathrm{i}=1}^{\mathrm{n}}{\left(1+{\mathrm{g}}_{{\hat{\mathrm{d}}}_{\mathrm{ ij}}}^{2}\right)}^{{\uptheta }_{\mathrm{i}}}\right)}^{{\upomega }_{\mathrm{j}}}- \prod_{\mathrm{j}=1}^{\mathrm{m}}{\left({\prod }_{\mathrm{i}=1}^{\mathrm{n}}{\left(1-{\mathrm{g}}_{{\hat{\mathrm{d}}}_{\mathrm{ ij}}}^{2}\right)}^{{\uptheta }_{\mathrm{i}}}\right)}^{{\upomega }_{\mathrm{j}}}}}{\sqrt{\prod_{\mathrm{j}=1}^{\mathrm{m}}{\left({\prod }_{\mathrm{i}=1}^{\mathrm{n}}{\left(1+{\mathrm{g}}_{{\hat{\mathrm{d}}}_{\mathrm{ ij}}}^{2}\right)}^{{\uptheta }_{\mathrm{i}}}\right)}^{{\upomega }_{\mathrm{j}}}+ \prod_{\mathrm{j}=1}^{\mathrm{m}}{\left({\prod }_{\mathrm{i}=1}^{\mathrm{n}}{\left(1-{\mathrm{g}}_{{\hat{\mathrm{d}}}_{\mathrm{ ij}}}^{2}\right)}^{{\uptheta }_{\mathrm{i}}}\right)}^{{\upomega }_{\mathrm{j}}}}}\end{array}\Bigg\rangle$$

These existing AOs for PFHSS were developed based on algebraic operational laws, and Einstein's operational laws failed to handle the situation when the ${\left({\mathrm{f}}_{{\hat{\mathrm{d}}}_{\mathrm{ij}}}\right)}^{2}+{\left({\mathrm{g}}_{{\hat{\mathrm{d}}}_{\mathrm{ij}}}\right)}^{2}>1$. To overawed these confines, Khan et al.^59^ offered the enlightened structure acknowledged as a q-ROFHSS that adroitly contracts with the abovementioned anxieties.

### Definition^57^

Let $\mathcal{U}$ be a universe of discourse and $\mathcal{P}$($\mathcal{U}$) be a power set of $\mathcal{U}$ and $\mathrm{k}=\left\{{\mathrm{k}}_{1}, {\mathrm{k}}_{2}, {\mathrm{k}}_{3},..., {\mathrm{k}}_{\mathrm{n}}\right\},\left(\mathrm{n }\ge 1\right)$ and ${\mathrm{K}}_{\mathrm{i}}$ showed the set of parameters and their correspondent sub-parameters, such as ${\mathrm{K}}_{\mathrm{i}}\cap {\mathrm{K}}_{\mathrm{j}}=\mathrm{\varphi }$, where $\mathrm{i }\ne \mathrm{ j}$ for each *n* ≥ 1 and $\mathrm{i},\mathrm{ j}\in \{\mathrm{1,2},3 \dots \mathrm{ n}\}$. Assume ${\mathrm{K}}_{1} \times {\mathrm{K}}_{2} \times {\mathrm{K}}_{3}\times \dots \times {\mathrm{K}}_{\mathrm{n}} = \mathop{{\mathcal{A}}}\limits^{\ldots} = \left\{{\mathrm{d}}_{1\mathrm{h}}\times {\mathrm{d}}_{2\mathrm{k}}\times \cdots \times {\mathrm{d}}_{\mathrm{nl}}\right\}$ is an collection of sub-parameters, where $1\le \mathrm{h}\le \mathrm{\alpha }$, $1\le \mathrm{k}\le\upbeta$, and $1\le \mathrm{l}\le\upgamma$, and $\mathrm{\alpha },\upbeta ,\upgamma \in {\mathbb{N}}$. and ${\mathrm{q}-\mathrm{ROFS}}^{\mathcal{U}}$ represents the collection of all subsets of q-ROFS over $\mathcal{U}$. Then $\left(\mathcal{F}, {\mathrm{K}}_{1} \times {\mathrm{K}}_{2} \times {\mathrm{K}}_{3}\times \dots \times {\mathrm{K}}_{\mathrm{n}}\right)=\left(\mathcal{F}, \mathop{{\mathcal{A}}}\limits^{\ldots}\right)$ is called q-ROFHSS and is defined as:

$$\mathcal{F}:{\mathrm{K}}_{1} \times {\mathrm{K}}_{2} \times {\mathrm{K}}_{3}\times \dots \times {\mathrm{K}}_{\mathrm{n}}=\mathop{{\mathcal{A}}}\limits^{\ldots}\to {\mathrm{q}-\mathrm{ROFS}}^{\mathcal{U}}.$$

Also, it can be defined as

$\left(\mathcal{F}, \mathop{{\mathcal{A}}}\limits^{\ldots}\right)=\left\{\left(\hat{\mathrm{d}}, {\mathcal{F}}_{\mathop{{\mathcal{A}}}\limits^{\ldots}}\left(\hat{\mathrm{d}}\right)\right): \hat{\mathrm{d}}\in \mathop{{\mathcal{A}}}\limits^{\ldots}, {\mathcal{F}}_{\mathop{{\mathcal{A}}}\limits^{\ldots}}\left(\hat{\mathrm{d}}\right)\in {\mathrm{PFS}}^{\mathcal{U}}\in \left[0, 1\right]\right\}$, where ${\mathcal{F}}_{\mathop{{\mathcal{A}}}\limits^{\ldots}}\left(\hat{\mathrm{d}}\right)=\left\{\Bigg\langle \updelta , {\mathrm{f}}_{{\hat{\mathrm{d}}}_{\mathrm{ij}}}\left(\updelta \right), {\mathrm{g}}_{{\hat{\mathrm{d}}}_{\mathrm{ij}}}\left(\updelta \right)\Bigg\rangle :\updelta \in \mathcal{U}\right\}$, where ${\mathrm{f}}_{{\hat{\mathrm{d}}}_{\mathrm{ij}}}\left(\updelta \right)$ and ${\mathrm{g}}_{{\hat{\mathrm{d}}}_{\mathrm{ij}}}\left(\updelta \right)$ shows the MD and NMD, such as ${\mathrm{f}}_{{\hat{\mathrm{d}}}_{\mathrm{ij}}}\left(\updelta \right), {\mathrm{g}}_{{\hat{\mathrm{d}}}_{\mathrm{ij}}}\left(\updelta \right)\in \left[0, 1\right]$, and $0\le {\left({\mathrm{f}}_{{\hat{\mathrm{d}}}_{\mathrm{ij}}}\left(\updelta \right)\right)}^{\mathrm{q}}+{\left({\mathrm{g}}_{{\hat{\mathrm{d}}}_{\mathrm{ij}}}\left(\updelta \right)\right)}^{\mathrm{q}}\le 1$.

A q-ROFHSN is stated as $\mathcal{F}=\left\{\left( {\mathrm{f}}_{{\hat{\mathrm{d}}}_{\mathrm{ij}}}\left(\updelta \right), {\mathrm{g}}_{{\hat{\mathrm{d}}}_{\mathrm{ij}}}\left(\updelta \right)\right)\right\}$, where $0\le {\left({\mathrm{f}}_{{\hat{\mathrm{d}}}_{\mathrm{ij}}}\left(\updelta \right)\right)}^{\mathrm{q}}+{\left({\mathrm{g}}_{{\hat{\mathrm{d}}}_{\mathrm{ij}}}\left(\updelta \right)\right)}^{\mathrm{q}}\le 1$.

### Definition^59^

Let ${\mathrm{J}}_{{\hat{\mathrm{d}}}_{\mathrm{k}}}=\left({\mathrm{f}}_{{\hat{\mathrm{d}}}_{\mathrm{k}}}, {\mathrm{g}}_{{\hat{\mathrm{d}}}_{\mathrm{k}}}\right)$, ${\mathrm{J}}_{{\hat{\mathrm{d}}}_{11}}=\left({\mathrm{f}}_{{\hat{\mathrm{d}}}_{11}}, {\mathrm{g}}_{{\hat{\mathrm{d}}}_{11}}\right)$, and ${\mathrm{J}}_{{\hat{\mathrm{d}}}_{12}}=\left({\mathrm{f}}_{{\hat{\mathrm{d}}}_{12}}, {\mathrm{g}}_{{\hat{\mathrm{d}}}_{12}}\right)$ be the q-ROFHSNs, and $\upgamma >0$. Then, their operational laws are defined as:

1. $${\mathrm{J}}_{{\hat{\mathrm{d}}}_{11}}\oplus {\mathrm{J}}_{{\hat{\mathrm{d}}}_{12}}=\Bigg\langle \sqrt[\mathrm{q}]{{{\mathrm{f}}_{{\hat{\mathrm{d}}}_{11}}}^{\mathrm{q}}+{{\mathrm{f}}_{{\hat{\mathrm{d}}}_{12}}}^{\mathrm{q}}-{{\mathrm{f}}_{{\hat{\mathrm{d}}}_{11}}}^{\mathrm{q}}{{\mathrm{f}}_{{\hat{\mathrm{d}}}_{12}}}^{\mathrm{q}}}, {\mathrm{g}}_{{\hat{\mathrm{d}}}_{11}}{\mathrm{g}}_{{\hat{\mathrm{d}}}_{12}}\Bigg\rangle$$
2. $${\mathrm{J}}_{{\hat{\mathrm{d}}}_{11}}\otimes {\mathrm{J}}_{{\hat{\mathrm{d}}}_{12}}=\Bigg\langle {\mathrm{f}}_{{\hat{\mathrm{d}}}_{11}}{\mathrm{f}}_{{\hat{\mathrm{d}}}_{12}}, \sqrt[\mathrm{q}]{{{\mathrm{g}}_{{\hat{\mathrm{d}}}_{11}}}^{\mathrm{q}}+{{\mathrm{g}}_{{\hat{\mathrm{d}}}_{12}}}^{\mathrm{q}}-{{\mathrm{g}}_{{\hat{\mathrm{d}}}_{11}}}^{\mathrm{q}}{{\mathrm{g}}_{{\hat{\mathrm{d}}}_{12}}}^{\mathrm{q}}}\Bigg\rangle$$
3. $$\upgamma {\mathrm{J}}_{{\hat{\mathrm{d}}}_{\mathrm{k}}}=\Bigg\langle \sqrt[\mathrm{q}]{1-{\left(1-{{\mathrm{f}}_{{\hat{\mathrm{d}}}_{\mathrm{k}}}}^{\mathrm{q}}\right)}^{\upgamma }}, {{\mathrm{g}}_{{\hat{\mathrm{d}}}_{\mathrm{k}}}}^{\upgamma }\Bigg\rangle$$
4. $${\mathrm{J}}_{{\hat{\mathrm{d}}}_{\mathrm{k}}}^{\upgamma }=\Bigg\langle {{\mathrm{f}}_{{\hat{\mathrm{d}}}_{\mathrm{k}}}}^{\upgamma }, \sqrt[\mathrm{q}]{1-{\left(1-{{\mathrm{g}}_{{\hat{\mathrm{d}}}_{\mathrm{k}}}}^{\mathrm{q}}\right)}^{\upgamma }}\Bigg\rangle$$

For the multiplicity of q-ROFHSNs ${\mathrm{J}}_{{\hat{\mathrm{d}}}_{\mathrm{k}}}$, where ${\uptheta }_{\mathrm{i}}$ and ${\upomega }_{\mathrm{j}}$ represents the weights experts and sub-parameters, such as ${\uptheta }_{\mathrm{i}}>0,\sum_{\mathrm{i}=1}^{\mathrm{n}}{\uptheta }_{\mathrm{i}}=1; {\upomega }_{\mathrm{j}}>0, \sum_{\mathrm{j}=1}^{\mathrm{m}}{\upomega }_{\mathrm{j}}=1$. The AOs^59^ for q-ROFHSS are given as follows:

3 $$\mathrm{q}-\mathrm{ROFHSWA }\left({\mathrm{J}}_{{\hat{\mathrm{d}}}_{11}}, {\mathrm{J}}_{{\hat{\mathrm{d}}}_{12}},\dots , {\mathrm{J}}_{{\hat{\mathrm{d}}}_{\mathrm{nm}}}\right)=\Bigg\langle \begin{array}{c}\sqrt[\mathrm{q}]{1-\prod_{\mathrm{j}=1}^{\mathrm{m}}{\left(\prod_{\mathrm{i}=1}^{\mathrm{n}}{\left(1-{{\mathrm{f}}_{{\hat{\mathrm{d}}}_{\mathrm{ij}}}}^{\mathrm{q}}\right)}^{{\uptheta }_{\mathrm{i}}}\right)}^{{\upomega }_{\mathrm{j}}}}, \\ \prod_{\mathrm{j}=1}^{\mathrm{m}}{\left(\prod_{\mathrm{i}=1}^{\mathrm{n}}{\left({\mathrm{g}}_{{\hat{\mathrm{d}}}_{\mathrm{ij}}}\right)}^{{\uptheta }_{\mathrm{i}}}\right)}^{{\upomega }_{\mathrm{j}}}\end{array}\Bigg\rangle$$

4 $$\mathrm{q}-\mathrm{ROFHSWG }\left({\mathrm{J}}_{{\hat{\mathrm{d}}}_{11}}, {\mathrm{J}}_{{\hat{\mathrm{d}}}_{12}},\dots , {\mathrm{J}}_{{\hat{\mathrm{d}}}_{\mathrm{nm}}}\right)=\Bigg\langle \begin{array}{c} \prod_{\mathrm{j}=1}^{\mathrm{m}}{\left(\prod_{\mathrm{i}=1}^{\mathrm{n}}{\left({\mathrm{f}}_{{\hat{\mathrm{d}}}_{\mathrm{ij}}}\right)}^{{\uptheta }_{\mathrm{i}}}\right)}^{{\upomega }_{\mathrm{j}}}, \\ \sqrt[\mathrm{q}]{1-\prod_{\mathrm{j}=1}^{\mathrm{m}}{\left(\prod_{\mathrm{i}=1}^{\mathrm{n}}{\left(1-{{\mathrm{g}}_{{\hat{\mathrm{d}}}_{\mathrm{ij}}}}^{\mathrm{q}}\right)}^{{\uptheta }_{\mathrm{i}}}\right)}^{{\upomega }_{\mathrm{j}}}}\end{array}\Bigg\rangle$$

#### Remark 1

1. If ${\left({\mathrm{f}}_{{\hat{\mathrm{d}}}_{\mathrm{ij}}}\left(\updelta \right)\right)}^{\mathrm{q}}+{\left({\mathrm{g}}_{{\hat{\mathrm{d}}}_{\mathrm{ij}}}\left(\updelta \right)\right)}^{\mathrm{q}}\le 1$ and ${\left({\mathrm{f}}_{{\hat{\mathrm{d}}}_{\mathrm{ij}}}\left(\updelta \right)\right)}^{2}+{\left({\mathrm{g}}_{{\hat{\mathrm{d}}}_{\mathrm{ij}}}\left(\updelta \right)\right)}^{2}\le 1$ holds. Then, q-ROFHSS becomes the PFHSS^52^.
2. If ${\left({\mathrm{f}}_{{\hat{\mathrm{d}}}_{\mathrm{ij}}}\left(\updelta \right)\right)}^{\mathrm{q}}+{\left({\mathrm{g}}_{{\hat{\mathrm{d}}}_{\mathrm{ij}}}\left(\updelta \right)\right)}^{\mathrm{q}}\le 1$ and ${\mathrm{f}}_{{\hat{\mathrm{d}}}_{\mathrm{ij}}}\left(\updelta \right)+{\mathrm{g}}_{{\hat{\mathrm{d}}}_{\mathrm{ij}}}\left(\updelta \right)\le 1$ holds. Then, q-ROFHSS becomes the IFHSS^49^.

The q-ROFHSN ${\mathcal{F}}_{{\updelta }_{\mathrm{i}}}\left({\hat{\mathrm{d}}}_{\mathrm{j}}\right)=\left\{\left({\mathrm{f}}_{\mathcal{F}({\hat{\mathrm{d}}}_{\mathrm{j}})}\left({\updelta }_{\mathrm{i}}\right), {\mathrm{g}}_{\mathcal{F}({\hat{\mathrm{d}}}_{\mathrm{j}})}\left({\updelta }_{\mathrm{i}}\right)\right)\mathrm{ \vert}{\updelta }_{\mathrm{i}}\in \mathcal{U}\right\}$ is described as ${\mathrm{J}}_{{\hat{\mathrm{d}}}_{\mathrm{ij}}}=\Bigg\langle {\mathrm{f}}_{{\hat{\mathrm{d}}}_{\mathrm{ij}}}, {\mathrm{g}}_{{\hat{\mathrm{d}}}_{\mathrm{ij}}}\Bigg\rangle$. The score function for ${\mathrm{J}}_{{\hat{\mathrm{d}}}_{\mathrm{ij}}}$ is stated as:

5 $$\mathrm{S}\left({\mathrm{J}}_{{\hat{\mathrm{d}}}_{\mathrm{ij}}}\right)={\mathrm{f}}_{{\hat{\mathrm{d}}}_{\mathrm{ij}}}^{\mathrm{q}}-{\mathrm{g}}_{{\hat{\mathrm{d}}}_{\mathrm{ij}}}^{\mathrm{q}}+\left(\frac{{\mathrm{e}}^{{\mathrm{f}}_{{\hat{\mathrm{d}}}_{\mathrm{ij}}}^{\mathrm{q}}-{\mathrm{g}}_{{\hat{\mathrm{d}}}_{\mathrm{ij}}}^{\mathrm{q}}}}{{\mathrm{e}}^{{\mathrm{f}}_{{\hat{\mathrm{d}}}_{\mathrm{ij}}}^{\mathrm{q}}-{\mathrm{g}}_{{\hat{\mathrm{d}}}_{\mathrm{ij}}}^{\mathrm{q}}}+1}-\frac{1}{2}\right){\mathrm{\beth }}_{{\mathrm{J}}_{{\hat{\mathrm{d}}}_{\mathrm{ij}}}}^{\mathrm{q}},\mathrm{ for q}\ge 3\mathrm{ and S}\left({\mathrm{J}}_{{\hat{\mathrm{d}}}_{\mathrm{ij}}}\right)\in \left[-1, 1\right].$$

Let ${\mathrm{J}}_{{\hat{\mathrm{d}}}_{11}}=\left({\mathrm{f}}_{{\hat{\mathrm{d}}}_{11}}, {\mathrm{g}}_{{\hat{\mathrm{d}}}_{11}}\right)$ and ${\mathrm{J}}_{{\hat{\mathrm{d}}}_{12}}=\left({\mathrm{f}}_{{\hat{\mathrm{d}}}_{12}}, {\mathrm{g}}_{{\hat{\mathrm{d}}}_{12}}\right)$ be two q-ROFHSNs. Then

If $$\mathrm{S}\left({\mathrm{J}}_{{\hat{\mathrm{d}}}_{11}}\right)>\mathrm{S}\left({\mathrm{J}}_{{\hat{\mathrm{d}}}_{12}}\right)$$, then $${\mathrm{J}}_{{\hat{\mathrm{d}}}_{11}}\succcurlyeq {\mathrm{J}}_{{\hat{\mathrm{d}}}_{12}}$$.

If $$\mathrm{S}\left({\mathrm{J}}_{{\hat{\mathrm{d}}}_{11}}\right)<\mathrm{S}\left({\mathrm{J}}_{{\hat{\mathrm{d}}}_{12}}\right)$$, then $${\mathrm{J}}_{{\hat{\mathrm{d}}}_{11}}\preccurlyeq {\mathrm{J}}_{{\hat{\mathrm{d}}}_{12}}$$.

If $$\mathrm{S}\left({\mathrm{J}}_{{\hat{\mathrm{d}}}_{11}}\right)=\mathrm{S}\left({\mathrm{J}}_{{\hat{\mathrm{d}}}_{12}}\right)$$, then

If $${\mathrm{\beth }}_{{\mathrm{J}}_{{\hat{\mathrm{d}}}_{11}}}>{\mathrm{\beth }}_{{\mathrm{J}}_{{\hat{\mathrm{d}}}_{12}}}$$, then $${\mathrm{J}}_{{\hat{\mathrm{d}}}_{11}}<{\mathrm{J}}_{{\hat{\mathrm{d}}}_{12}}$$

If $${\mathrm{\beth }}_{{\mathrm{J}}_{{\hat{\mathrm{d}}}_{11}}}^{\mathrm{q}}={\mathrm{\beth }}_{{\mathrm{J}}_{{\hat{\mathrm{d}}}_{12}}}^{\mathrm{q}}$$, then $${\mathrm{J}}_{{\hat{\mathrm{d}}}_{11}}={\mathrm{J}}_{{\hat{\mathrm{d}}}_{12}}$$

For the comparison among two q-ROFHSNs ${\mathrm{J}}_{{\hat{\mathrm{d}}}_{\mathrm{ij}}}$ and ${\mathfrak{T}}_{{\hat{\mathrm{d}}}_{\mathrm{ij}}}$, comparison laws are defined as:

If $$\mathrm{S}\left({\mathrm{J}}_{{\hat{\mathrm{d}}}_{\mathrm{ij}}}\right)>\mathrm{S}\left({\mathfrak{T}}_{{\hat{\mathrm{d}}}_{\mathrm{ij}}}\right)$$, then $${\mathrm{J}}_{{\hat{\mathrm{d}}}_{\mathrm{ij}}}>{\mathfrak{T}}_{{\hat{\mathrm{d}}}_{\mathrm{ij}}}$$.

If $$\mathrm{S}\left({\mathrm{J}}_{{\hat{\mathrm{d}}}_{\mathrm{ij}}}\right)=\mathrm{S}\left({\mathfrak{T}}_{{\hat{\mathrm{d}}}_{\mathrm{ij}}}\right)$$, then

1. If $$\mathrm{A}\left({\mathrm{J}}_{{\hat{\mathrm{d}}}_{\mathrm{ij}}}\right)>\mathrm{A}\left({\mathfrak{T}}_{{\hat{\mathrm{d}}}_{\mathrm{ij}}}\right)$$, then $${\mathrm{J}}_{{\hat{\mathrm{d}}}_{\mathrm{ij}}}>{\mathfrak{T}}_{{\hat{\mathrm{d}}}_{\mathrm{ij}}}$$
2. If $$\mathrm{A}\left({\mathrm{J}}_{{\hat{\mathrm{d}}}_{\mathrm{ij}}}\right)=\mathrm{A}\left({\mathfrak{T}}_{{\hat{\mathrm{d}}}_{\mathrm{ij}}}\right)$$, then $${\mathrm{J}}_{{\hat{\mathrm{d}}}_{\mathrm{ij}}}={\mathfrak{T}}_{{\hat{\mathrm{d}}}_{\mathrm{ij}}}$$.

The prevailing Einstein-ordered weighted AOs for PFHSS only evaluate PFHSS influences and only contemplate the ordered positions of the PFHSS estimations, not the q-ROFHSS influences themselves. Similarly, from the above AOs for q-ROFHSS, it is remarked that, in assertive environments, these AOs convey some repulsive significance.

## Einstein weighted average aggregation operator for q-rung orthopair fuzzy hypersoft set

This section will present an innovative Einstein-weighted average aggregation operator for q-ROFHSNs with the most necessary properties.

### Definition

Let ${\mathrm{J}}_{{\hat{\mathrm{d}}}_{\mathrm{k}}}=\left({\mathrm{f}}_{{\hat{\mathrm{d}}}_{\mathrm{k}}}, {\mathrm{g}}_{{\hat{\mathrm{d}}}_{\mathrm{k}}}\right)$, ${\mathrm{J}}_{{\hat{\mathrm{d}}}_{11}}=\left({\mathrm{f}}_{{\hat{\mathrm{d}}}_{11}}, {\mathrm{g}}_{{\hat{\mathrm{d}}}_{11}}\right)$ and ${\mathrm{J}}_{{\hat{\mathrm{d}}}_{12}}=\left({\mathrm{f}}_{{\hat{\mathrm{d}}}_{12}}, {\mathrm{g}}_{{\hat{\mathrm{d}}}_{12}}\right)$ represent the q-ROFHSNs, and $\upgamma >0$. Then, Einstein's operational laws are defined as:

1. $${\mathrm{J}}_{{\hat{\mathrm{d}}}_{11}}{\oplus}_{\mathrm{\upepsilon }} {\mathrm{J}}_{{\hat{\mathrm{d}}}_{12}}=\Bigg\langle \sqrt[\mathrm{q}]{\frac{\left(1+{\mathrm{f}}_{{\hat{\mathrm{d}}}_{11}}^{\mathrm{q}}\right)-\left(1-{\mathrm{f}}_{{\hat{\mathrm{d}}}_{12}}^{\mathrm{q}}\right)}{\left(1+{\mathrm{f}}_{{\hat{\mathrm{d}}}_{11}}^{\mathrm{q}}\right)-\left(1-{\mathrm{f}}_{{\hat{\mathrm{d}}}_{12}}^{\mathrm{q}}\right)}}, \sqrt[\mathrm{q}]{\frac{2\left({\mathrm{g}}_{{\hat{\mathrm{d}}}_{12}}^{\mathrm{q}}\right)}{\left(2-{\mathrm{g}}_{{\hat{\mathrm{d}}}_{11}}^{\mathrm{q}}\right)+\left({\mathrm{g}}_{{\hat{\mathrm{d}}}_{12}}^{\mathrm{q}}\right)}}\Bigg\rangle$$
2. $${\mathrm{J}}_{{\hat{\mathrm{d}}}_{11}}{\otimes}_{\mathrm{\upepsilon }} {\mathrm{J}}_{{\hat{\mathrm{d}}}_{12}} =\Bigg\langle \sqrt[\mathrm{q}]{\frac{2\left({\mathrm{f}}_{{\hat{\mathrm{d}}}_{12}}^{\mathrm{q}}\right)}{\left(2-{\mathrm{f}}_{12}^{\mathrm{q}}\right)+\left({\mathrm{f}}_{{\hat{\mathrm{d}}}_{12}}^{\mathrm{q}}\right)}}, \sqrt[\mathrm{q}]{\frac{\left(1+{\mathrm{g}}_{{\hat{\mathrm{d}}}_{11}}^{\mathrm{q}}\right)-\left(1-{\mathrm{g}}_{{\hat{\mathrm{d}}}_{12}}^{\mathrm{q}}\right)}{\left(1+{\mathrm{g}}_{{\hat{\mathrm{d}}}_{11}}^{\mathrm{q}}\right)-\left(1-{\mathrm{g}}_{{\hat{\mathrm{d}}}_{12}}^{\mathrm{q}}\right)}}\Bigg\rangle$$
3. $$\upgamma {\mathrm{J}}_{{\hat{\mathrm{d}}}_{\mathrm{k}}}=\Bigg\langle \sqrt[\mathrm{q}]{\frac{{\left(1+{\mathrm{f}}_{{\hat{\mathrm{d}}}_{\mathrm{k}}}^{\mathrm{q}}\right)}^{\upgamma }-{\left(1-{\mathrm{f}}_{{\hat{\mathrm{d}}}_{\mathrm{k}}}^{\mathrm{q}}\right)}^{\upgamma }}{{\left(1+{\mathrm{f}}_{{\hat{\mathrm{d}}}_{\mathrm{k}}}^{\mathrm{q}}\right)}^{\upgamma }+{\left(1-{\mathrm{f}}_{{\hat{\mathrm{d}}}_{\mathrm{k}}}^{\mathrm{q}}\right)}^{\upgamma }}}, \sqrt[\mathrm{q}]{\frac{{2\left({\mathrm{g}}_{{\hat{\mathrm{d}}}_{\mathrm{k}}}^{\mathrm{q}}\right)}^{\upgamma }}{{\left(2-{\mathrm{g}}_{\mathrm{k}}^{\mathrm{q}}\right)}^{\upgamma }+{\left({\mathrm{g}}_{{\hat{\mathrm{d}}}_{\mathrm{k}}}^{\mathrm{q}}\right)}^{\upgamma }}}\Bigg\rangle$$
4. $${\mathrm{J}}_{{\hat{\mathrm{d}}}_{\mathrm{k}}}^{\upgamma }=\Bigg\langle \sqrt[\mathrm{q}]{\frac{2{\left({\mathrm{f}}_{{\hat{\mathrm{d}}}_{\mathrm{k}}}^{\mathrm{q}}\right)}^{\upgamma }}{{\left(2-{\mathrm{f}}_{{\hat{\mathrm{d}}}_{\mathrm{k}}}^{\mathrm{q}}\right)}^{\upgamma }+{\left({\mathrm{f}}_{{\hat{\mathrm{d}}}_{\mathrm{k}}}^{\mathrm{q}}\right)}^{\upgamma }}}, \sqrt[\mathrm{q}]{\frac{{\left(1+{\mathrm{g}}_{{\hat{\mathrm{d}}}_{\mathrm{k}}}^{\mathrm{q}}\right)}^{\upgamma }-{\left(1-{\mathrm{g}}_{{\hat{\mathrm{d}}}_{\mathrm{k}}}^{\mathrm{q}}\right)}^{\upgamma }}{{\left(1+{\mathrm{g}}_{{\hat{\mathrm{d}}}_{\mathrm{k}}}^{\mathrm{q}}\right)}^{\upgamma }+{\left(1-{\mathrm{g}}_{{\hat{\mathrm{d}}}_{\mathrm{k}}}^{\mathrm{q}}\right)}^{\upgamma }}}\Bigg\rangle$$

### Definition

Let ${\mathrm{J}}_{{\hat{\mathrm{d}}}_{\mathrm{k}}}=\left({\mathrm{f}}_{{\hat{\mathrm{d}}}_{\mathrm{k}}}, {\mathrm{g}}_{{\hat{\mathrm{d}}}_{\mathrm{k}}}\right)$ be a collection of q-ROFHSNs; then the q-ROFHSEWA operator is defined as follows:

6 $$\mathrm{q}-\mathrm{ROFHSEWA}\left({\mathrm{J}}_{{\hat{\mathrm{d}}}_{11}}, {\mathrm{J}}_{{\hat{\mathrm{d}}}_{12}}, \dots ,{\mathrm{J}}_{{\hat{\mathrm{d}}}_{\mathrm{nm}}}\right)= {{\oplus}_{\mathrm{\upepsilon }}}_{\mathrm{j}=1}^{\mathrm{m}}{\upomega }_{\mathrm{j}}\left({{\oplus}_{\mathrm{\upepsilon }}}_{\mathrm{i}=1}^{\mathrm{n}}{\uptheta }_{\mathrm{i}}{\mathrm{J}}_{{\hat{\mathrm{d}}}_{\mathrm{ij}}}\right)$$

where ${\uptheta }_{\mathrm{i}}$ and ${\upomega }_{\mathrm{j}}$ denote the weights such as: ${\uptheta }_{\mathrm{i}}>0$, ${\sum }_{\mathrm{i}=1}^{\mathrm{n}}{\uptheta }_{\mathrm{i}}=1$, and ${\upomega }_{\mathrm{j}}>0,$ ${\sum }_{\mathrm{j}=1}^{\mathrm{m}}{\upomega }_{\mathrm{j}}=1$.

### Theorem

Let ${\mathrm{J}}_{{\hat{\mathrm{d}}}_{\mathrm{ij}}}=\left({\mathrm{f}}_{{\hat{\mathrm{d}}}_{\mathrm{ij}}}, {\mathrm{g}}_{{\hat{\mathrm{d}}}_{\mathrm{ij}}}\right)$ be a collection of q-ROFHSNs; then the attained aggregated value using Eq. (6) is given as:

7 $$\begin{aligned} & \mathrm{q}-\mathrm{ROFHSEWA}\left({\mathrm{J}}_{{\hat{\mathrm{d}}}_{11}}, {\mathrm{J}}_{{\hat{\mathrm{d}}}_{12}}, \dots ,{\mathrm{J}}_{{\hat{\mathrm{d}}}_{\mathrm{nm}}}\right)= {{\oplus}_{\mathrm{\upepsilon }}}_{\mathrm{j}=1}^{\mathrm{m}}{\upomega }_{\mathrm{j}}\left({{\oplus}_{\mathrm{\upepsilon }}}_{\mathrm{i}=1}^{\mathrm{n}}{\uptheta }_{\mathrm{i}}{\mathrm{J}}_{{\hat{\mathrm{d}}}_{\mathrm{ij}}}\right)\\ &\quad=\Bigg\langle \frac{\sqrt[\mathrm{q}]{\prod_{\mathrm{j}=1}^{\mathrm{m}}{\left(\prod_{\mathrm{i}=1}^{\mathrm{n}}{\left(1+{\mathrm{f}}_{{\hat{\mathrm{d}}}_{\mathrm{ij}}}^{\mathrm{q}}\right)}^{{\uptheta }_{\mathrm{i}}}\right)}^{{\upomega }_{\mathrm{j}}}- \prod_{\mathrm{j}=1}^{\mathrm{m}}{\left(\prod_{\mathrm{i}=1}^{\mathrm{n}}{\left(1-{\mathrm{f}}_{{\hat{\mathrm{d}}}_{\mathrm{ij}}}^{\mathrm{q}}\right)}^{{\uptheta }_{\mathrm{i}}}\right)}^{{\upomega }_{\mathrm{j}}}}}{\sqrt[\mathrm{q}]{\prod_{\mathrm{j}=1}^{\mathrm{m}}{\left(\prod_{\mathrm{i}=1}^{\mathrm{n}}{\left(1+{\mathrm{f}}_{{\hat{\mathrm{d}}}_{\mathrm{ij}}}^{\mathrm{q}}\right)}^{{\uptheta }_{\mathrm{i}}}\right)}^{{\upomega }_{\mathrm{j}}}+ \prod_{\mathrm{j}=1}^{\mathrm{m}}{\left(\prod_{\mathrm{i}=1}^{\mathrm{n}}{\left(1-{\mathrm{f}}_{{\hat{\mathrm{d}}}_{\mathrm{ij}}}^{\mathrm{q}}\right)}^{{\uptheta }_{\mathrm{i}}}\right)}^{{\upomega }_{\mathrm{j}}}}},\\&\qquad \frac{\sqrt[\mathrm{q}]{2\prod_{\mathrm{j}=1}^{\mathrm{m}}{\left({\prod_{\mathrm{i}=1}^{\mathrm{n}}\left({\mathrm{g}}_{{\hat{\mathrm{d}}}_{\mathrm{ij}}}^{\mathrm{q}}\right)}^{{\uptheta }_{\mathrm{i}}}\right)}^{{\upomega }_{\mathrm{j}}}}}{\sqrt[\mathrm{q}]{\prod_{\mathrm{j}=1}^{\mathrm{m}}{\left(\prod_{\mathrm{i}=1}^{\mathrm{n}}{\left(2-{\mathrm{g}}_{{\hat{\mathrm{d}}}_{\mathrm{ij}}}^{\mathrm{q}}\right)}^{{\uptheta }_{\mathrm{i}}}\right)}^{{\upomega }_{\mathrm{j}}}+ \prod_{\mathrm{j}=1}^{\mathrm{m}}{\left({\prod_{\mathrm{i}=1}^{\mathrm{n}}\left({\mathrm{g}}_{{\hat{\mathrm{d}}}_{\mathrm{ij}}}^{\mathrm{q}}\right)}^{{\uptheta }_{\mathrm{i}}}\right)}^{{\upomega }_{\mathrm{j}}}}}\Bigg\rangle \end{aligned}$$

where ${\uptheta }_{\mathrm{i}}$ and ${\upomega }_{\mathrm{j}}$ denote the weights such as: ${\uptheta }_{\mathrm{i}}>0$, ${\sum }_{\mathrm{i}=1}^{\mathrm{n}}{\uptheta }_{\mathrm{i}}=1$ and ${\upomega }_{\mathrm{j}}>0,$ ${\sum }_{\mathrm{j}=1}^{\mathrm{m}}{\upomega }_{\mathrm{j}}=1$.

#### Proof

We will demonstrate it by employing mathematical induction.

For $\mathrm{n}=1,$ we get ${\uptheta }_{\mathrm{i}}=1$

$$\begin{aligned}&=\Bigg\langle \frac{\sqrt[\mathrm{q}]{\prod_{\mathrm{j}=1}^{\mathrm{m}}{\left(\prod_{\mathrm{i}=1}^{\mathrm{n}}{\left(1+{\mathrm{f}}_{{\hat{\mathrm{d}}}_{\mathrm{ij}}}^{\mathrm{q}}\right)}^{{\uptheta }_{\mathrm{i}}}\right)}^{{\upomega }_{\mathrm{j}}}- \prod_{\mathrm{j}=1}^{\mathrm{m}}{\left(\prod_{\mathrm{i}=1}^{\mathrm{n}}{\left(1-{\mathrm{f}}_{{\hat{\mathrm{d}}}_{\mathrm{ij}}}^{\mathrm{q}}\right)}^{{\uptheta }_{\mathrm{i}}}\right)}^{{\upomega }_{\mathrm{j}}}}}{\sqrt[\mathrm{q}]{\prod_{\mathrm{j}=1}^{\mathrm{m}}{\left(\prod_{\mathrm{i}=1}^{\mathrm{n}}{\left(1+{\mathrm{f}}_{{\hat{\mathrm{d}}}_{\mathrm{ij}}}^{\mathrm{q}}\right)}^{{\uptheta }_{\mathrm{i}}}\right)}^{{\upomega }_{\mathrm{j}}}+ \prod_{\mathrm{j}=1}^{\mathrm{m}}{\left(\prod_{\mathrm{i}=1}^{\mathrm{n}}{\left(1-{\mathrm{f}}_{{\hat{\mathrm{d}}}_{\mathrm{ij}}}^{\mathrm{q}}\right)}^{{\uptheta }_{\mathrm{i}}}\right)}^{{\upomega }_{\mathrm{j}}}}},\\&\quad \frac{\sqrt[\mathrm{q}]{2\prod_{\mathrm{j}=1}^{\mathrm{m}}{\left({\prod_{\mathrm{i}=1}^{\mathrm{n}}\left({\mathrm{g}}_{{\hat{\mathrm{d}}}_{\mathrm{ij}}}^{\mathrm{q}}\right)}^{{\uptheta }_{\mathrm{i}}}\right)}^{{\upomega }_{\mathrm{j}}}}}{\sqrt[\mathrm{q}]{\prod_{\mathrm{j}=1}^{\mathrm{m}}{\left(\prod_{\mathrm{i}=1}^{\mathrm{n}}{\left(2-{\mathrm{g}}_{{\hat{\mathrm{d}}}_{\mathrm{ij}}}^{\mathrm{q}}\right)}^{{\uptheta }_{\mathrm{i}}}\right)}^{{\upomega }_{\mathrm{j}}}+ \prod_{\mathrm{j}=1}^{\mathrm{m}}{\left({\prod_{\mathrm{i}=1}^{\mathrm{n}}\left({\mathrm{g}}_{{\hat{\mathrm{d}}}_{\mathrm{ij}}}^{\mathrm{q}}\right)}^{{\uptheta }_{\mathrm{i}}}\right)}^{{\upomega }_{\mathrm{j}}}}}\Bigg\rangle \end{aligned}$$

$$\begin{aligned} & \mathrm{q}-\mathrm{ROFHSEWA}\left({\mathrm{J}}_{{\hat{\mathrm{d}}}_{11}}, {\mathrm{J}}_{{\hat{\mathrm{d}}}_{12}}, \dots ,{\mathrm{J}}_{{\hat{\mathrm{d}}}_{\mathrm{nm}}}\right)= {{\oplus}_{\mathrm{\upepsilon }}}_{\mathrm{j}=1}^{\mathrm{m}}{\upomega }_{\mathrm{j}}{\mathrm{J}}_{{\hat{\mathrm{d}}}_{1\mathrm{j}}}\\ &\quad=\Bigg\langle \frac{\sqrt[\mathrm{q}]{\prod_{\mathrm{j}=1}^{\mathrm{m}}{\left(1+{\mathrm{f}}_{{\hat{\mathrm{d}}}_{1\mathrm{j}}}^{\mathrm{q}}\right)}^{{\upomega }_{\mathrm{j}}}- \prod_{\mathrm{j}=1}^{\mathrm{m}}{\left(1-{\mathrm{f}}_{{\hat{\mathrm{d}}}_{1\mathrm{j}}}^{\mathrm{q}}\right)}^{{\upomega }_{\mathrm{j}}}}}{\sqrt[\mathrm{q}]{\prod_{\mathrm{j}=1}^{\mathrm{m}}{\left(1+{\mathrm{f}}_{{\hat{\mathrm{d}}}_{1\mathrm{j}}}^{\mathrm{q}}\right)}^{{\upomega }_{\mathrm{j}}}+ \prod_{\mathrm{j}=1}^{\mathrm{m}}{\left(1-{\mathrm{f}}_{{\hat{\mathrm{d}}}_{1\mathrm{j}}}^{\mathrm{q}}\right)}^{{\upomega }_{\mathrm{j}}}}}, \frac{\sqrt[\mathrm{q}]{2\prod_{\mathrm{j}=1}^{\mathrm{m}}{\left({\mathrm{g}}_{{\hat{\mathrm{d}}}_{1\mathrm{j}}}^{\mathrm{q}}\right)}^{{\upomega }_{\mathrm{j}}}}}{\sqrt[\mathrm{q}]{\prod_{\mathrm{j}=1}^{\mathrm{m}}{\left(2-{\mathrm{g}}_{{\hat{\mathrm{d}}}_{1\mathrm{j}}}^{\mathrm{q}}\right)}^{{\upomega }_{\mathrm{j}}}+ \prod_{\mathrm{j}=1}^{\mathrm{m}}{\left({\mathrm{g}}_{{\hat{\mathrm{d}}}_{1\mathrm{j}}}^{\mathrm{q}}\right)}^{{\upomega }_{\mathrm{j}}}}}\Bigg\rangle \end{aligned}$$

$$\begin{aligned}& \mathrm{q}-\mathrm{ROFHSEWA}\left({\mathrm{J}}_{{\hat{\mathrm{d}}}_{11}}, {\mathrm{J}}_{{\hat{\mathrm{d}}}_{12}}, \dots ,{\mathrm{J}}_{{\hat{\mathrm{d}}}_{\mathrm{nm}}}\right)= {{\oplus}_{\mathrm{\upepsilon }}}_{\mathrm{i}=1}^{\mathrm{n}}{\uptheta }_{\mathrm{i}}{\mathrm{J}}_{{\hat{\mathrm{d}}}_{\mathrm{i}1}}\\ &\quad=\Bigg\langle \frac{\sqrt[\mathrm{q}]{\prod_{\mathrm{i}=1}^{\mathrm{n}}{\left(1+{\mathrm{f}}_{{\hat{\mathrm{d}}}_{\mathrm{i}1}}^{\mathrm{q}}\right)}^{{\uptheta }_{\mathrm{i}}}- \prod_{\mathrm{i}=1}^{\mathrm{n}}{\left(1-{\mathrm{f}}_{{\hat{\mathrm{d}}}_{\mathrm{i}1}}^{\mathrm{q}}\right)}^{{\uptheta }_{\mathrm{i}}}}}{\sqrt[\mathrm{q}]{\prod_{\mathrm{i}=1}^{\mathrm{n}}{\left(1+{\mathrm{f}}_{{\hat{\mathrm{d}}}_{\mathrm{i}1}}^{\mathrm{q}}\right)}^{{\uptheta }_{\mathrm{i}}}+ \prod_{\mathrm{i}=1}^{\mathrm{n}}{\left(1-{\mathrm{f}}_{{\hat{\mathrm{d}}}_{\mathrm{i}1}}^{\mathrm{q}}\right)}^{{\uptheta }_{\mathrm{i}}}}}, \frac{\sqrt[\mathrm{q}]{2\prod_{\mathrm{i}=1}^{\mathrm{n}}{\left({\mathrm{g}}_{{\hat{\mathrm{d}}}_{\mathrm{i}1}}^{\mathrm{q}}\right)}^{{\uptheta }_{\mathrm{i}}}}}{\sqrt[\mathrm{q}]{\prod_{\mathrm{i}=1}^{\mathrm{n}}{\left(2-{\mathrm{g}}_{{\hat{\mathrm{d}}}_{\mathrm{i}1}}^{\mathrm{q}}\right)}^{{\uptheta }_{\mathrm{i}}}+ \prod_{\mathrm{i}=1}^{\mathrm{n}}{\left({\mathrm{g}}_{{\hat{\mathrm{d}}}_{\mathrm{i}1}}^{\mathrm{q}}\right)}^{{\uptheta }_{\mathrm{i}}}}}\Bigg\rangle \end{aligned}$$

For $\mathrm{m}=1$, we get ${\upomega }_{\mathrm{j}}$= 1.

$$\begin{aligned} & \mathrm{q}-\mathrm{ROFHSEWA}\left({\mathrm{J}}_{{\hat{\mathrm{d}}}_{11}}, {\mathrm{J}}_{{\hat{\mathrm{d}}}_{12}}, \dots ,{\mathrm{J}}_{{\hat{\mathrm{d}}}_{\mathrm{nm}}}\right)= {{\oplus}_{\mathrm{\upepsilon }}}_{\mathrm{i}=1}^{\mathrm{n}}{\uptheta }_{\mathrm{i}}{\mathrm{J}}_{{\hat{\mathrm{d}}}_{\mathrm{i}1}}\\ &\quad=\Bigg\langle \frac{\sqrt[\mathrm{q}]{\prod_{\mathrm{i}=1}^{\mathrm{n}}{\left(1+{\mathrm{f}}_{{\hat{\mathrm{d}}}_{\mathrm{i}1}}^{\mathrm{q}}\right)}^{{\uptheta }_{\mathrm{i}}}- \prod_{\mathrm{i}=1}^{\mathrm{n}}{\left(1-{\mathrm{f}}_{{\hat{\mathrm{d}}}_{\mathrm{i}1}}^{\mathrm{q}}\right)}^{{\uptheta }_{\mathrm{i}}}}}{\sqrt[\mathrm{q}]{\prod_{\mathrm{i}=1}^{\mathrm{n}}{\left(1+{\mathrm{f}}_{{\hat{\mathrm{d}}}_{\mathrm{i}1}}^{\mathrm{q}}\right)}^{{\uptheta }_{\mathrm{i}}}+ \prod_{\mathrm{i}=1}^{\mathrm{n}}{\left(1-{\mathrm{f}}_{{\hat{\mathrm{d}}}_{\mathrm{i}1}}^{\mathrm{q}}\right)}^{{\uptheta }_{\mathrm{i}}}}}, \frac{\sqrt[\mathrm{q}]{2\prod_{\mathrm{i}=1}^{\mathrm{n}}{\left({\mathrm{g}}_{{\hat{\mathrm{d}}}_{\mathrm{i}1}}^{\mathrm{q}}\right)}^{{\uptheta }_{\mathrm{i}}}}}{\sqrt[\mathrm{q}]{\prod_{\mathrm{i}=1}^{\mathrm{n}}{\left(2-{\mathrm{g}}_{{\hat{\mathrm{d}}}_{\mathrm{i}1}}^{\mathrm{q}}\right)}^{{\uptheta }_{\mathrm{i}}}+ \prod_{\mathrm{i}=1}^{\mathrm{n}}{\left({\mathrm{g}}_{{\hat{\mathrm{d}}}_{\mathrm{i}1}}^{\mathrm{q}}\right)}^{{\uptheta }_{\mathrm{i}}}}}\Bigg\rangle \end{aligned}$$

$$\begin{aligned}&=\Bigg\langle \frac{\sqrt[\mathrm{q}]{\prod_{\mathrm{j}=1}^{1}{\left(\prod_{\mathrm{i}=1}^{\mathrm{n}}{\left(1+{\mathrm{f}}_{{\hat{\mathrm{d}}}_{\mathrm{ij}}}^{\mathrm{q}}\right)}^{{\uptheta }_{\mathrm{i}}}\right)}^{{\upomega }_{\mathrm{j}}}- \prod_{\mathrm{j}=1}^{1}{\left(\prod_{\mathrm{i}=1}^{\mathrm{n}}{\left(1-{\mathrm{f}}_{{\hat{\mathrm{d}}}_{\mathrm{ij}}}^{\mathrm{q}}\right)}^{{\uptheta }_{\mathrm{i}}}\right)}^{{\upomega }_{\mathrm{j}}}}}{\sqrt[\mathrm{q}]{\prod_{\mathrm{j}=1}^{1}{\left(\prod_{\mathrm{i}=1}^{\mathrm{n}}{\left(1+{\mathrm{f}}_{{\hat{\mathrm{d}}}_{\mathrm{ij}}}^{\mathrm{q}}\right)}^{{\uptheta }_{\mathrm{i}}}\right)}^{{\upomega }_{\mathrm{j}}}+ \prod_{\mathrm{j}=1}^{1}{\left(\prod_{\mathrm{i}=1}^{\mathrm{n}}{\left(1-{\mathrm{f}}_{{\hat{\mathrm{d}}}_{\mathrm{ij}}}^{\mathrm{q}}\right)}^{{\uptheta }_{\mathrm{i}}}\right)}^{{\upomega }_{\mathrm{j}}}}},\\ &\\ &\quad \frac{\sqrt[\mathrm{q}]{2\prod_{\mathrm{j}=1}^{1}{\left({\prod_{\mathrm{i}=1}^{\mathrm{n}}\left({\mathrm{g}}_{{\hat{\mathrm{d}}}_{\mathrm{ij}}}^{\mathrm{q}}\right)}^{{\uptheta }_{\mathrm{i}}}\right)}^{{\upomega }_{\mathrm{j}}}}}{\sqrt[\mathrm{q}]{\prod_{\mathrm{j}=1}^{1}{\left(\prod_{\mathrm{i}=1}^{\mathrm{n}}{\left(2-{\mathrm{g}}_{{\hat{\mathrm{d}}}_{\mathrm{ij}}}^{\mathrm{q}}\right)}^{{\uptheta }_{\mathrm{i}}}\right)}^{{\upomega }_{\mathrm{j}}}+ \prod_{\mathrm{j}=1}^{1}{\left({\prod_{\mathrm{i}=1}^{\mathrm{n}}\left({\mathrm{g}}_{{\hat{\mathrm{d}}}_{\mathrm{ij}}}^{\mathrm{q}}\right)}^{{\uptheta }_{\mathrm{i}}}\right)}^{{\upomega }_{\mathrm{j}}}}}\Bigg\rangle \end{aligned}$$

So, Eq. (7) is hold for $\mathrm{n}=1$ and $\mathrm{m}=1$.

Assume Eq. (7) holds for $\mathrm{n}={\mathrm{n}}_{1}\mathrm{ and m}={\mathrm{m}}_{1}$

$$\begin{aligned}{{\oplus}_{\mathrm{\upepsilon }}}_{\mathrm{j}=1}^{{\mathrm{m}}_{1}}{\upomega }_{\mathrm{j}}\left({{\oplus}_{\mathrm{\upepsilon }}}_{\mathrm{i}=1}^{{\mathrm{n}}_{1}}{\uptheta }_{\mathrm{i}}{\mathrm{J}}_{{\hat{\mathrm{d}}}_{\mathrm{ij}}}\right)&=\Bigg\langle \frac{\sqrt[\mathrm{q}]{\prod_{\mathrm{j}=1}^{{\mathrm{m}}_{1}}{\left(\prod_{\mathrm{i}=1}^{{\mathrm{n}}_{1}}{\left(1+{\mathrm{f}}_{{\hat{\mathrm{d}}}_{\mathrm{ij}}}^{\mathrm{q}}\right)}^{{\uptheta }_{\mathrm{i}}}\right)}^{{\upomega }_{\mathrm{j}}}- \prod_{\mathrm{j}=1}^{{\mathrm{m}}_{1}}{\left(\prod_{\mathrm{i}=1}^{{\mathrm{n}}_{1}}{\left(1-{\mathrm{f}}_{{\hat{\mathrm{d}}}_{\mathrm{ij}}}^{\mathrm{q}}\right)}^{{\uptheta }_{\mathrm{i}}}\right)}^{{\upomega }_{\mathrm{j}}}}}{\sqrt[\mathrm{q}]{\prod_{\mathrm{j}=1}^{{\mathrm{m}}_{1}}{\left(\prod_{\mathrm{i}=1}^{{\mathrm{n}}_{1}}{\left(1+{\mathrm{f}}_{{\hat{\mathrm{d}}}_{\mathrm{ij}}}^{\mathrm{q}}\right)}^{{\uptheta }_{\mathrm{i}}}\right)}^{{\upomega }_{\mathrm{j}}}+ \prod_{\mathrm{j}=1}^{{\mathrm{m}}_{1}}{\left(\prod_{\mathrm{i}=1}^{{\mathrm{n}}_{1}}{\left(1-{\mathrm{f}}_{{\hat{\mathrm{d}}}_{\mathrm{ij}}}^{\mathrm{q}}\right)}^{{\uptheta }_{\mathrm{i}}}\right)}^{{\upomega }_{\mathrm{j}}}}},\\ &\quad\frac{\sqrt[\mathrm{q}]{2\prod_{\mathrm{j}=1}^{{\mathrm{m}}_{1}}{\left({\prod_{\mathrm{i}=1}^{{\mathrm{n}}_{1}}\left({\mathrm{g}}_{{\hat{\mathrm{d}}}_{\mathrm{ij}}}^{\mathrm{q}}\right)}^{{\uptheta }_{\mathrm{i}}}\right)}^{{\upomega }_{\mathrm{j}}}}}{\sqrt[\mathrm{q}]{\prod_{\mathrm{j}=1}^{{\mathrm{m}}_{1}}{\left(\prod_{\mathrm{i}=1}^{{\mathrm{n}}_{1}}{\left(2-{\mathrm{g}}_{{\hat{\mathrm{d}}}_{\mathrm{ij}}}^{\mathrm{q}}\right)}^{{\uptheta }_{\mathrm{i}}}\right)}^{{\upomega }_{\mathrm{j}}}+ \prod_{\mathrm{j}=1}^{{\mathrm{m}}_{1}}{\left({\prod_{\mathrm{i}=1}^{{\mathrm{n}}_{1}}\left({\mathrm{g}}_{{\hat{\mathrm{d}}}_{\mathrm{ij}}}^{\mathrm{q}}\right)}^{{\uptheta }_{\mathrm{i}}}\right)}^{{\upomega }_{\mathrm{j}}}}}\Bigg\rangle \end{aligned}$$

Also, suppose that Eq. (7) holds for $\mathrm{n}={\mathrm{n}}_{1}+1$ and $\mathrm{m}={\mathrm{m}}_{1}+1$

$$\begin{aligned}{{\oplus}_{\mathrm{\upepsilon }}}_{\mathrm{j}=1}^{{\mathrm{m}}_{1}+1}{\upomega }_{\mathrm{j}}\left({{\oplus}_{\mathrm{\upepsilon }}}_{\mathrm{i}=1}^{{\mathrm{n}}_{1}+1}{\uptheta }_{\mathrm{i}}{\mathrm{J}}_{{\hat{\mathrm{d}}}_{\mathrm{ij}}}\right)&=\Bigg\langle \frac{\sqrt[\mathrm{q}]{\prod_{\mathrm{j}=1}^{{\mathrm{m}}_{1}+1}{\left(\prod_{\mathrm{i}=1}^{{\mathrm{n}}_{1}+1}{\left(1+{\mathrm{f}}_{{\hat{\mathrm{d}}}_{\mathrm{ij}}}^{\mathrm{q}}\right)}^{{\uptheta }_{\mathrm{i}}}\right)}^{{\upomega }_{\mathrm{j}}}- \prod_{\mathrm{j}=1}^{{\mathrm{m}}_{1}+1}{\left(\prod_{\mathrm{i}=1}^{{\mathrm{n}}_{1}+1}{\left(1-{\mathrm{f}}_{{\hat{\mathrm{d}}}_{\mathrm{ij}}}^{\mathrm{q}}\right)}^{{\uptheta }_{\mathrm{i}}}\right)}^{{\upomega }_{\mathrm{j}}}}}{\sqrt[\mathrm{q}]{\prod_{\mathrm{j}=1}^{{\mathrm{m}}_{1}+1}{\left(\prod_{\mathrm{i}=1}^{{\mathrm{n}}_{1}+1}{\left(1+{\mathrm{f}}_{{\hat{\mathrm{d}}}_{\mathrm{ij}}}^{\mathrm{q}}\right)}^{{\uptheta }_{\mathrm{i}}}\right)}^{{\upomega }_{\mathrm{j}}}+ \prod_{\mathrm{j}=1}^{{\mathrm{m}}_{1}+1}{\left(\prod_{\mathrm{i}=1}^{{\mathrm{n}}_{1}+1}{\left(1-{\mathrm{f}}_{{\hat{\mathrm{d}}}_{\mathrm{ij}}}^{\mathrm{q}}\right)}^{{\uptheta }_{\mathrm{i}}}\right)}^{{\upomega }_{\mathrm{j}}}}},\\ &\quad\frac{\sqrt[\mathrm{q}]{2\prod_{\mathrm{j}=1}^{{\mathrm{m}}_{1}+1}{\left({\prod_{\mathrm{i}=1}^{{\mathrm{n}}_{1}+1}\left({\mathrm{g}}_{{\hat{\mathrm{d}}}_{\mathrm{ij}}}^{\mathrm{q}}\right)}^{{\uptheta }_{\mathrm{i}}}\right)}^{{\upomega }_{\mathrm{j}}}}}{\sqrt[\mathrm{q}]{\prod_{\mathrm{j}=1}^{{\mathrm{m}}_{1}+1}{\left(\prod_{\mathrm{i}=1}^{{\mathrm{n}}_{1}+1}{\left(2-{\mathrm{g}}_{{\hat{\mathrm{d}}}_{\mathrm{ij}}}^{\mathrm{q}}\right)}^{{\uptheta }_{\mathrm{i}}}\right)}^{{\upomega }_{\mathrm{j}}}+ \prod_{\mathrm{j}=1}^{{\mathrm{m}}_{1}+1}{\left(\begin{array}{c}{\prod_{\mathrm{i}=1}^{{\mathrm{n}}_{1}+1}\left({\mathrm{g}}_{{\hat{\mathrm{d}}}_{\mathrm{ij}}}^{\mathrm{q}}\right)}^{{\uptheta }_{\mathrm{i}}}\end{array}\right)}^{{\upomega }_{\mathrm{j}}}}}\Bigg\rangle \end{aligned}$$

Now we prove the Eq. (7) for $\mathrm{n}={\mathrm{n}}_{1}+2$ and $\mathrm{m}={\mathrm{m}}_{1}+2$

$$\begin{aligned}{{\oplus}_{\mathrm{\upepsilon }}}_{\mathrm{j}=1}^{{\mathrm{m}}_{1}+2}{\upomega }_{\mathrm{j}}\left({{\oplus}_{\mathrm{\upepsilon }}}_{\mathrm{i}=1}^{{\mathrm{n}}_{1}+2}{\uptheta }_{\mathrm{i}}{\mathrm{J}}_{{\hat{\mathrm{d}}}_{\mathrm{ij}}}\right)&=\Bigg\langle \frac{\sqrt[\mathrm{q}]{\prod_{\mathrm{j}=1}^{{\mathrm{m}}_{1}+2}{\left(\prod_{\mathrm{i}=1}^{{\mathrm{n}}_{1}}{\left(1+{\mathrm{f}}_{{\hat{\mathrm{d}}}_{\mathrm{ij}}}^{\mathrm{q}}\right)}^{{\uptheta }_{\mathrm{i}}}\right)}^{{\upomega }_{\mathrm{j}}}- \prod_{\mathrm{j}=1}^{{\mathrm{m}}_{1}+2}{\left(\prod_{\mathrm{i}=1}^{{\mathrm{n}}_{1}}{\left(1-{\mathrm{f}}_{{\hat{\mathrm{d}}}_{\mathrm{ij}}}^{\mathrm{q}}\right)}^{{\uptheta }_{\mathrm{i}}}\right)}^{{\upomega }_{\mathrm{j}}}}}{\sqrt[\mathrm{q}]{\prod_{\mathrm{j}=1}^{{\mathrm{m}}_{1}+2}{\left(\prod_{\mathrm{i}=1}^{{\mathrm{n}}_{1}}{\left(1+{\mathrm{f}}_{{\hat{\mathrm{d}}}_{\mathrm{ij}}}^{\mathrm{q}}\right)}^{{\uptheta }_{\mathrm{i}}}\right)}^{{\upomega }_{\mathrm{j}}}- \prod_{\mathrm{j}=1}^{{\mathrm{m}}_{1}+2}{\left(\prod_{\mathrm{i}=1}^{{\mathrm{n}}_{1}}{\left(1-{\mathrm{f}}_{{\hat{\mathrm{d}}}_{\mathrm{ij}}}^{\mathrm{q}}\right)}^{{\uptheta }_{\mathrm{i}}}\right)}^{{\upomega }_{\mathrm{j}}}}}\\&\quad{\oplus}_{\mathrm{\upepsilon }}\frac{\sqrt[\mathrm{q}]{\prod_{\mathrm{j}=1}^{{\mathrm{m}}_{1}+2}{\left({\left((1+{\mathrm{f}}_{{\hat{\mathrm{d}}}_{\left({\mathrm{n}}_{1}+2\right)\mathrm{j}}}^{\mathrm{q}}\right)}^{{\uptheta }_{{\mathrm{n}}_{1}+2}}\right)}^{{\upomega }_{\mathrm{j}}}- \prod_{\mathrm{j}=1}^{{\mathrm{m}}_{1}+2}{\left({\left((1-{\mathrm{f}}_{{\hat{\mathrm{d}}}_{\left({\mathrm{n}}_{1}+2\right)\mathrm{j}}}^{\mathrm{q}}\right)}^{{\uptheta }_{{\mathrm{n}}_{1}+2}}\right)}^{{\upomega }_{\mathrm{j}}}}}{\sqrt[\mathrm{q}]{\prod_{\mathrm{j}=1}^{{\mathrm{m}}_{1}+2}{\left({\left((1+{\mathrm{f}}_{{\hat{\mathrm{d}}}_{\left({\mathrm{n}}_{1}+2\right)\mathrm{j}}}^{\mathrm{q}}\right)}^{{\uptheta }_{{\mathrm{n}}_{1}+2}}\right)}^{{\upomega }_{\mathrm{j}}}- \prod_{\mathrm{j}=1}^{{\mathrm{m}}_{1}+2}{\left({\left(1-{\mathrm{f}}_{{\hat{\mathrm{d}}}_{\left({\mathrm{n}}_{1}+2\right)\mathrm{j}}}^{\mathrm{q}}\right)}^{{\uptheta }_{{\mathrm{n}}_{1}+2}}\right)}^{{\upomega }_{\mathrm{j}}}}}, \\&\quad \frac{\sqrt[\mathrm{q}]{2\prod_{\mathrm{j}=1}^{{\mathrm{m}}_{1}+2}{\left({\prod_{\mathrm{i}=1}^{{\mathrm{n}}_{1}+2}\left(2-{\mathrm{g}}_{{\hat{\mathrm{d}}}_{\mathrm{ij}}}^{\mathrm{q}}\right)}^{{\uptheta }_{\mathrm{i}}}\right)}^{{\upomega }_{\mathrm{j}}}}}{\sqrt[\mathrm{q}]{\prod_{\mathrm{j}=1}^{{\mathrm{m}}_{1}+2}{\left(\prod_{\mathrm{i}=1}^{{\mathrm{n}}_{1}+2}{\left(2-{\mathrm{g}}_{{\hat{\mathrm{d}}}_{\mathrm{ij}}}^{\mathrm{q}}\right)}^{{\uptheta }_{\mathrm{i}}}\right)}^{{\upomega }_{\mathrm{j}}}+ \prod_{\mathrm{j}=1}^{{\mathrm{m}}_{1}+2}{\left({\prod_{\mathrm{i}=1}^{{\mathrm{n}}_{1}+2}\left({\mathrm{g}}_{{\hat{\mathrm{d}}}_{\mathrm{ij}}}^{\mathrm{q}}\right)}^{{\uptheta }_{\mathrm{i}}}\right)}^{{\upomega }_{\mathrm{j}}}}}\\&\quad{\oplus}_{{\upepsilon }}\frac{\sqrt[\mathrm{q}]{2\prod_{\mathrm{j}=1}^{{\mathrm{m}}_{1}+2}{\left({\left({\mathrm{g}}_{{\hat{\mathrm{d}}}_{({\mathrm{n}}_{1}+2)\mathrm{j}}}^{\mathrm{q}}\right)}^{{\uptheta }_{({\mathrm{n}}_{1}+2)}}\right)}^{{\upomega }_{\mathrm{j}}}}}{\sqrt[\mathrm{q}]{\prod_{\mathrm{j}=1}^{{\mathrm{m}}_{1}+2}{\left({\left(2-{\mathrm{g}}_{{\hat{\mathrm{d}}}_{\mathrm{ij}}}^{\mathrm{q}}\right)}^{{\uptheta }_{({\mathrm{n}}_{1}+2)}}\right)}^{{\upomega }_{\mathrm{j}}}+ \prod_{\mathrm{j}=1}^{{\mathrm{m}}_{1}+2}{\left({\left({\mathrm{g}}_{{\hat{\mathrm{d}}}_{({\mathrm{n}}_{1}+2)\mathrm{j}}}^{\mathrm{q}}\right)}^{{\uptheta }_{({\mathrm{n}}_{1}+2)}}\right)}^{{\upomega }_{\mathrm{j}}}}}\Bigg\rangle \end{aligned}$$

$$\begin{aligned} & =\Bigg\langle \frac{\sqrt[\mathrm{q}]{\prod_{\mathrm{j}=1}^{{\mathrm{m}}_{1}+2}{\left(\prod_{\mathrm{i}=1}^{{\mathrm{n}}_{1}+2}{\left(1+{\mathrm{f}}_{{\hat{\mathrm{d}}}_{\mathrm{ij}}}^{\mathrm{q}}\right)}^{{\uptheta }_{\mathrm{i}}}\right)}^{{\upomega }_{\mathrm{j}}}- \prod_{\mathrm{j}=1}^{{\mathrm{m}}_{1}+2}{\left(\prod_{\mathrm{i}=1}^{{\mathrm{n}}_{1}+2}{\left(1-{\mathrm{f}}_{{\hat{\mathrm{d}}}_{\mathrm{ij}}}^{\mathrm{q}}\right)}^{{\uptheta }_{\mathrm{i}}}\right)}^{{\upomega }_{\mathrm{j}}}}}{\sqrt[\mathrm{q}]{\prod_{\mathrm{j}=1}^{{\mathrm{m}}_{1}+2}{\left(\prod_{\mathrm{i}=1}^{{\mathrm{n}}_{1}+2}{\left(1+{\mathrm{f}}_{{\hat{\mathrm{d}}}_{\mathrm{ij}}}^{\mathrm{q}}\right)}^{{\uptheta }_{\mathrm{i}}}\right)}^{{\upomega }_{\mathrm{j}}}+ \prod_{\mathrm{j}=1}^{{\mathrm{m}}_{1}+2}{\left(\prod_{\mathrm{i}=1}^{{\mathrm{n}}_{1}+2}{\left(1-{\mathrm{f}}_{{\hat{\mathrm{d}}}_{\mathrm{ij}}}^{\mathrm{q}}\right)}^{{\uptheta }_{\mathrm{i}}}\right)}^{{\upomega }_{\mathrm{j}}}}},\\&\quad\frac{\sqrt[\mathrm{q}]{2\prod_{\mathrm{j}=1}^{{\mathrm{m}}_{1}+2}{\left({\prod_{\mathrm{i}=1}^{{\mathrm{n}}_{1}+2}\left({\mathrm{g}}_{{\hat{\mathrm{d}}}_{\mathrm{ij}}}^{\mathrm{q}}\right)}^{{\uptheta }_{\mathrm{i}}}\right)}^{{\upomega }_{\mathrm{j}}}}}{\sqrt[\mathrm{q}]{\prod_{\mathrm{j}=1}^{{\mathrm{m}}_{1}+2}{\left(\prod_{\mathrm{i}=1}^{{\mathrm{n}}_{1}+2}{\left(2-{\mathrm{g}}_{{\hat{\mathrm{d}}}_{\mathrm{ij}}}^{\mathrm{q}}\right)}^{{\uptheta }_{\mathrm{i}}}\right)}^{{\upomega }_{\mathrm{j}}}+ \prod_{\mathrm{j}=1}^{{\mathrm{m}}_{1}+2}{\left(\begin{array}{c}{\prod_{\mathrm{i}=1}^{{\mathrm{n}}_{1}+2}\left({\mathrm{g}}_{{\hat{\mathrm{d}}}_{\mathrm{ij}}}^{\mathrm{q}}\right)}^{{\uptheta }_{\mathrm{i}}}\end{array}\right)}^{{\upomega }_{\mathrm{j}}}}}\Bigg\rangle \end{aligned}$$

$$={{\oplus}_{\mathrm{\upepsilon }}}_{\mathrm{j}=1}^{{\mathrm{m}}_{1}+2}{\upomega }_{\mathrm{j}}\left({{\oplus}_{\mathrm{\upepsilon }}}_{\mathrm{i}=1}^{{\mathrm{n}}_{1}+2}{\uptheta }_{\mathrm{i}}{\mathrm{J}}_{{\hat{\mathrm{d}}}_{\mathrm{ij}}}\right)$$

So, it holds for $\mathrm{m}={\mathrm{m}}_{1}+2$ and $\mathrm{n}={\mathrm{n}}_{1}+2$, also it is true $\forall \mathrm{ m},\mathrm{ n }\ge 0$.

#### Example

Let $\mathrm{H}={\{\mathrm{H}}_{1}, {\mathrm{H}}_{2}, {\mathrm{H}}_{3}\}$ be a team of professionals with the most appropriate weighted vectors ${\uptheta }_{\mathrm{i}}={\left(0.3, 0.4, 0.3\right) }^{\mathrm{T}}$. The team of experts decided to buy a house under the set of attributes which are $\mathrm{A}=\left\{{\mathrm{d}}_{1}=\mathrm{infrastructure},{\mathrm{d}}_{2}=\mathrm{facilities},{\mathrm{d}}_{3}=\mathrm{sewerage system}, {\mathrm{d}}_{4}=\mathrm{security}\right\}$. For the selection of the house, the team of experts considered the multi-sub-attributes of the deliberated parameters, such as $\left\{{\mathrm{d}}_{1}=\text{Infrastructure}= \left\{{\text{d}}_{11}=\text{old style}, {\text{d}}_{11}=\text{new style}\right\} ,{\text{d}}_{2}=\text{Facilities}=\left\{{\text{d}}_{21}=\text{hospital}, {\text{d}}_{22}=\text{school}\right\} ,{\text{d}}_{3}=\text{Sewerage system}= \left\{{\text{d}}_{31}=\text{excellent}\right\}, {\mathrm{d}}_{4}=\text{Secuirty}= \left\{{\mathrm{d}}_{41}=\text{excellent}\right\}\right\}$. Let ${\mathfrak{L}}^{{^{\prime}}}={\mathrm{d}}_{1}\times {\mathrm{d}}_{2}\times {\mathrm{d}}_{3}\times {\mathrm{d}}_{4}$ represents the collection of multi-sub-attributes. $${\mathfrak{L}}^{{^{\prime}}}={\mathrm{d}}_{1}\times {\mathrm{d}}_{2}\times {\mathrm{d}}_{3}\times {\mathrm{d}}_{4}=\left\{{\mathrm{d}}_{11}, {\mathrm{d}}_{12}\right\}\times \left\{{\mathrm{d}}_{21}, {\mathrm{d}}_{22}\right\}\times \left\{{\mathrm{d}}_{31}\right\}\times \left\{{\mathrm{d}}_{42}\right\}=\left\{\begin{array}{c}\left({\mathrm{d}}_{11}, {\mathrm{d}}_{21}, {\mathrm{d}}_{31}, {\mathrm{d}}_{41}\right), \left({\mathrm{d}}_{11}, {\mathrm{d}}_{22}, {\mathrm{d}}_{31}, {\mathrm{d}}_{41}\right), \\ \left({\mathrm{d}}_{12}, {\mathrm{d}}_{21}, {\mathrm{d}}_{31}, {\mathrm{d}}_{41}\right), \left({\mathrm{d}}_{12}, {\mathrm{d}}_{22}, {\mathrm{d}}_{31}, {\mathrm{d}}_{41}\right)\end{array}\right\}=\left\{{\hat{\mathrm{d}}}_{1},{\hat{\mathrm{d}}}_{2}, {\hat{\mathrm{d}}}_{3}, {\hat{\mathrm{d}}}_{4}\right\}$$ describes the sub-attributes collection with weights ${\upomega }_{\mathrm{j}}={\left(0.2, 0.3, 0.4, 0.1\right)}^{\mathrm{T}}$. The team of experts assumes rating values $\left({\mathrm{J}}_{3\times 4},{\mathfrak{L}}^{{^{\prime}}}\right)={\left({\mathrm{f}}_{{\hat{\mathrm{d}}}_{\mathrm{ij}}},{\mathrm{g}}_{{\hat{\mathrm{d}}}_{\mathrm{ij}}}\right)}_{3\times 4}$ are given as follows:

$$\left({\mathrm{J}}_{3\times 4}, {\mathfrak{L}}^{{^{\prime}}}\right)=\left[\begin{array}{cccc}\left(0.5, 0.3\right)& \left(0.8, 0.7\right)& \left(0.6, 0.3\right)& \left(0.2, 0.9\right)\\ \left(0.6, 0.3\right)& \left(0.4, 0.7\right)& \left(0.4, 0.5\right)& \left(0.5, 0.6\right)\\ \left(0.3, 0.4\right)& \left(0.6, 0.8\right)& \left(0.3, 0.9\right)& \left(0.2, 0.7\right)\end{array}\right]$$

Use Eq. (7)

$$\begin{aligned}&=\Bigg\langle \frac{\sqrt[\mathrm{q}]{\prod_{\mathrm{j}=1}^{\mathrm{m}}{\left(\prod_{\mathrm{i}=1}^{\mathrm{n}}{\left(1+{\mathrm{f}}_{{\hat{\mathrm{d}}}_{\mathrm{ij}}}^{\mathrm{q}}\right)}^{{\uptheta }_{\mathrm{i}}}\right)}^{{\upomega }_{\mathrm{j}}}- \prod_{\mathrm{j}=1}^{\mathrm{m}}{\left(\prod_{\mathrm{i}=1}^{\mathrm{n}}{\left(1-{\mathrm{f}}_{{\hat{\mathrm{d}}}_{\mathrm{ij}}}^{\mathrm{q}}\right)}^{{\uptheta }_{\mathrm{i}}}\right)}^{{\upomega }_{\mathrm{j}}}}}{\sqrt[\mathrm{q}]{\prod_{\mathrm{j}=1}^{\mathrm{m}}{\left(\prod_{\mathrm{i}=1}^{\mathrm{n}}{\left(1+{\mathrm{f}}_{{\hat{\mathrm{d}}}_{\mathrm{ij}}}^{\mathrm{q}}\right)}^{{\uptheta }_{\mathrm{i}}}\right)}^{{\upomega }_{\mathrm{j}}}+ \prod_{\mathrm{j}=1}^{\mathrm{m}}{\left(\prod_{\mathrm{i}=1}^{\mathrm{n}}{\left(1-{\mathrm{f}}_{{\hat{\mathrm{d}}}_{\mathrm{ij}}}^{\mathrm{q}}\right)}^{{\uptheta }_{\mathrm{i}}}\right)}^{{\upomega }_{\mathrm{j}}}}},\\&\quad\frac{\sqrt[\mathrm{q}]{2\prod_{\mathrm{j}=1}^{\mathrm{m}}{\left({\prod_{\mathrm{i}=1}^{\mathrm{n}}\left({\mathrm{g}}_{{\hat{\mathrm{d}}}_{\mathrm{ij}}}^{\mathrm{q}}\right)}^{{\uptheta }_{\mathrm{i}}}\right)}^{{\upomega }_{\mathrm{j}}}}}{\sqrt[\mathrm{q}]{\prod_{\mathrm{j}=1}^{\mathrm{m}}{\left(\prod_{\mathrm{i}=1}^{\mathrm{n}}{\left(2-{\mathrm{g}}_{{\hat{\mathrm{d}}}_{\mathrm{ij}}}^{\mathrm{q}}\right)}^{{\uptheta }_{\mathrm{i}}}\right)}^{{\upomega }_{\mathrm{j}}}+ \prod_{\mathrm{j}=1}^{\mathrm{m}}{\left({\prod_{\mathrm{i}=1}^{\mathrm{n}}\left({\mathrm{g}}_{{\hat{\mathrm{d}}}_{\mathrm{ij}}}^{\mathrm{q}}\right)}^{{\uptheta }_{\mathrm{i}}}\right)}^{{\upomega }_{\mathrm{j}}}}}\Bigg\rangle \end{aligned}$$

For $$\mathrm{q}=3$$

$$\begin{aligned}&=\Bigg\langle \frac{\sqrt[3]{\prod_{\mathrm{j}=1}^{\mathrm{m}}{\left(\prod_{\mathrm{i}=1}^{\mathrm{n}}{\left(1+{\mathrm{f}}_{{\hat{\mathrm{d}}}_{\mathrm{ij}}}^{3}\right)}^{{\uptheta }_{\mathrm{i}}}\right)}^{{\upomega }_{\mathrm{j}}}- \prod_{\mathrm{j}=1}^{\mathrm{m}}{\left(\prod_{\mathrm{i}=1}^{\mathrm{n}}{\left(1-{\mathrm{f}}_{{\hat{\mathrm{d}}}_{\mathrm{ij}}}^{3}\right)}^{{\uptheta }_{\mathrm{i}}}\right)}^{{\upomega }_{\mathrm{j}}}}}{\sqrt[3]{\prod_{\mathrm{j}=1}^{\mathrm{m}}{\left(\prod_{\mathrm{i}=1}^{\mathrm{n}}{\left(1+{\mathrm{f}}_{{\hat{\mathrm{d}}}_{\mathrm{ij}}}^{3}\right)}^{{\uptheta }_{\mathrm{i}}}\right)}^{{\upomega }_{\mathrm{j}}}+ \prod_{\mathrm{j}=1}^{\mathrm{m}}{\left(\prod_{\mathrm{i}=1}^{\mathrm{n}}{\left(1-{\mathrm{f}}_{{\hat{\mathrm{d}}}_{\mathrm{ij}}}^{3}\right)}^{{\uptheta }_{\mathrm{i}}}\right)}^{{\upomega }_{\mathrm{j}}}}},\\ &\quad \frac{\sqrt[3]{2\prod_{\mathrm{j}=1}^{\mathrm{m}}{\left({\prod_{\mathrm{i}=1}^{\mathrm{n}}\left({\mathrm{g}}_{{\hat{\mathrm{d}}}_{\mathrm{ij}}}^{3}\right)}^{{\uptheta }_{\mathrm{i}}}\right)}^{{\upomega }_{\mathrm{j}}}}}{\sqrt[3]{\prod_{\mathrm{j}=1}^{\mathrm{m}}{\left(\prod_{\mathrm{i}=1}^{\mathrm{n}}{\left(2-{\mathrm{g}}_{{\hat{\mathrm{d}}}_{\mathrm{ij}}}^{3}\right)}^{{\uptheta }_{\mathrm{i}}}\right)}^{{\upomega }_{\mathrm{j}}}+ \prod_{\mathrm{j}=1}^{\mathrm{m}}{\left({\prod_{\mathrm{i}=1}^{\mathrm{n}}\left({\mathrm{g}}_{{\hat{\mathrm{d}}}_{\mathrm{ij}}}^{3}\right)}^{{\uptheta }_{\mathrm{i}}}\right)}^{{\upomega }_{\mathrm{j}}}}}\Bigg\rangle \end{aligned}$$

$$=\Bigg\langle \begin{array}{c}\frac{\sqrt[3]{\begin{array}{c}{\left\{\left(1.0359\right)\left(1.0814\right)\left(1.0080\right)\right\}}^{0.2} {\left\{\left(1.1320\right)\left(1.0251\right)\left(1.0604\right)\right\}}^{0.3} {\left\{\left(1.0604\right)\left(1.0251\right)\left(1.0080\right)\right\}}^{0.4} {\left\{\left(1.0024\right)\left(1.0482\right)\left(1.0024\right)\right\}}^{0.1}-\\ {\left\{\left(0.9607\right)\left(0.9072\right)\left(0.9918\right)\right\}}^{0.2} {\left\{\left(0.8064\right)\left(0.9739\right)\left(0.9296\right)\right\}}^{0.3} {\left\{\left(0.9296\right)\left(0.9739\right)\left(0.9918\right)\right\}}^{0.4} {\left\{\left(0.9976\right)\left(0.9479\right)\left(0.9976\right)\right\}}^{0.1}\end{array}}}{\sqrt[3]{\begin{array}{c}{\left\{\left(1.0359\right)\left(1.0814\right)\left(1.0080\right)\right\}}^{0.2} {\left\{\left(1.1320\right)\left(1.0251\right)\left(1.0604\right)\right\}}^{0.3} {\left\{\left(1.0604\right)\left(1.0251\right)\left(1.0080\right)\right\}}^{0.4} {\left\{\left(1.0024\right)\left(1.0482\right)\left(1.0024\right)\right\}}^{0.1}+\\ {\left\{\left(0.9607\right)\left(0.9072\right)\left(0.9918\right)\right\}}^{0.2} {\left\{\left(0.8064\right)\left(0.9739\right)\left(0.9296\right)\right\}}^{0.3} {\left\{\left(0.9296\right)\left(0.9739\right)\left(0.9918\right)\right\}}^{0.4} {\left\{\left(0.9976\right)\left(0.9479\right)\left(0.9976\right)\right\}}^{0.1}\end{array}}},\\ \frac{\sqrt[3]{2{\left\{\left(0.3384\right)\left(0.2358\right)\left(0.4384\right)\right\}}^{0.2}{\left\{\left(0.7254\right)\left(0.6518\right)\left(0.8181\right)\right\}}^{0.3}{\left\{\left(0.3384\right)\left(0.4353\right)\left(0.9095\right)\right\}}^{0.4}{\left\{\left(0.9095\right)\left(0.5417\right)\left(0.7254\right)\right\}}^{0.1}}}{\begin{array}{c}\sqrt[3]{\begin{array}{c}{\left\{\left(1.2261\right)\left(1.3124\right)\left(1.2192\right)\right\}}^{0.2}{\left\{\left(1.1636\right)\left(1.2239\right)\left(1.1266\right)\right\}}^{0.3}{\left\{\left(1.2261\right)\left(1.2859\right)\left(1.0746\right)\right\}}^{0.4}{\left\{\left(1.0746\right)\left(1.2605\right)\left(1.1636\right)\right\}}^{0.1}+\\ {\left\{\left(0.3384\right)\left(0.2358\right)\left(0.4384\right)\right\}}^{0.2}{\left\{\left(0.7254\right)\left(0.6518\right)\left(0.8181\right)\right\}}^{0.3}{\left\{\left(0.3384\right)\left(0.4353\right)\left(0.9095\right)\right\}}^{0.4}{\left\{\left(0.9095\right)\left(0.5417\right)\left(0.7254\right)\right\}}^{0.1}\end{array}}\end{array}}\end{array}\Bigg\rangle$$

$$=\Bigg\langle \begin{array}{c}\frac{\sqrt[3]{\begin{array}{c}\left(1.0246\right) \left(1.0433\right)\left(1.0115\right)\left(1.0279\right)-\left(0.9694\right)\left(0.9448\right)\left(0.9865\right)\left(0.9665\right)\end{array}}}{\sqrt[3]{\begin{array}{c}\left(1.0246\right) \left(1.0433\right)\left(1.0115\right)\left(1.0279\right)+\left(0.9694\right)\left(0.9448\right)\left(0.9865\right)\left(0.9665\right)\end{array}}},\\ \frac{\sqrt[3]{2\left\{\left(0.5114\right)\left(0.7521\right)\left(0.4475\right)\left(0.9022\right)\right\}}}{\begin{array}{c}\sqrt[3]{\left\{\left(1.1443\right)\left(1.1524\right)\left(1.1935\right)\left(1.0465\right)\right\}+\left\{\left(0.5114\right)\left(0.7521\right)\left(0.4475\right)\left(0.9022\right)\right\}}\end{array}}\end{array}\Bigg\rangle$$

$$=\langle \begin{array}{c}\mathrm{0.4967,0.7748}\end{array}\rangle .$$

### Lemma

Let ${\mathrm{J}}_{{\hat{\mathrm{d}}}_{\mathrm{ij}}}=\left({\mathrm{f}}_{{\hat{\mathrm{d}}}_{\mathrm{ij}}}, {\mathrm{g}}_{{\hat{\mathrm{d}}}_{\mathrm{ij}}}\right)$ , where ${\uptheta }_{\mathrm{i}}>0$, ${\sum }_{\mathrm{i}=1}^{\mathrm{n}}{\uptheta }_{\mathrm{i}} =1$, and ${\upomega }_{\mathrm{j}}>0,$ ${\sum }_{\mathrm{j}=1}^{\mathrm{m}}{\upomega }_{\mathrm{j}}=1$, then

$${\prod }_{\mathrm{j}=1}^{\mathrm{m}}{\left({\prod }_{\mathrm{i}=1}^{\mathrm{n}}{\left({\mathrm{J}}_{{\hat{\mathrm{d}}}_{\mathrm{ij}}}\right)}^{{\uptheta }_{\mathrm{i}}}\right)}^{{\upomega }_{\mathrm{j}}}= {\sum }_{\mathrm{j}=1}^{\mathrm{m}}{\upomega }_{\mathrm{j}} \left({\sum }_{\mathrm{i}=1}^{\mathrm{n}}{\uptheta }_{\mathrm{i}}\left({\mathrm{J}}_{{\hat{\mathrm{d}}}_{\mathrm{ij}}}\right)\right)$$

### Theorem

Let ${\mathrm{J}}_{{\hat{\mathrm{d}}}_{\mathrm{ij}}}=\left({\mathrm{f}}_{{\hat{\mathrm{d}}}_{\mathrm{ij}}}, {\mathrm{g}}_{{\hat{\mathrm{d}}}_{\mathrm{ij}}}\right)$ be a collection of q-ROFHSNs, then $\mathrm{q}-\mathrm{ROFHSWA}\left({\mathrm{J}}_{{\hat{\mathrm{d}}}_{11}}, {\mathrm{J}}_{{\hat{\mathrm{d}}}_{12}}, \dots ,{\mathrm{J}}_{{\hat{\mathrm{d}}}_{\mathrm{nm}}}\right)\ge \mathrm{q}-\mathrm{ROFHSEWA}\left({\mathrm{J}}_{{\hat{\mathrm{d}}}_{11}}, {\mathrm{J}}_{{\hat{\mathrm{d}}}_{12}}, \dots ,{\mathrm{J}}_{{\hat{\mathrm{d}}}_{\mathrm{nm}}}\right)$. Where ${\uptheta }_{\mathrm{i}}$ and ${\upomega }_{\mathrm{j}}$ represent the weighted vectors such that ${\uptheta }_{\mathrm{i}}>0$, ${\sum }_{\mathrm{i}=1}^{\mathrm{n}}{\uptheta }_{\mathrm{i}}=1$, and ${\upomega }_{\mathrm{j}}>0,$ ${\sum }_{\mathrm{j}=1}^{\mathrm{m}}{\upomega }_{\mathrm{j}}=1$.

#### Proof

We know that

$$\begin{aligned} & \sqrt[\mathrm{q}]{\prod_{\mathrm{j}=1}^{\mathrm{m}}{\left(\prod_{\mathrm{i}=1}^{\mathrm{n}}{\left(1+{\mathrm{f}}_{{\hat{\mathrm{d}}}_{\mathrm{ij}}}^{\mathrm{q}}\right)}^{{\uptheta }_{\mathrm{i}}}\right)}^{{\upomega }_{\mathrm{j}}}+\prod_{\mathrm{j}=1}^{\mathrm{m}}{\left(\prod_{\mathrm{i}=1}^{\mathrm{n}}{\left(1-{\mathrm{f}}_{{\hat{\mathrm{d}}}_{\mathrm{ij}}}^{\mathrm{q}}\right)}^{{\uptheta }_{\mathrm{i}}}\right)}^{{\upomega }_{\mathrm{j}}}}\\ &\quad\le \sqrt[\mathrm{q}]{{\sum }_{\mathrm{j}=1}^{\mathrm{m}}{\upomega }_{\mathrm{j}}{\sum }_{\mathrm{i}=1}^{\mathrm{n}}{\uptheta }_{\mathrm{i}}\left(1+{\mathrm{f}}_{{\hat{\mathrm{d}}}_{\mathrm{ij}}}^{\mathrm{q}}\right)+{\sum }_{\mathrm{j}=1}^{\mathrm{m}}{\upomega }_{\mathrm{j}}{\sum }_{\mathrm{i}=1}^{\mathrm{n}}{\uptheta }_{\mathrm{i}}\left(1-{\mathrm{f}}_{{\hat{\mathrm{d}}}_{\mathrm{ij}}}^{\mathrm{q}}\right)}\end{aligned}$$

$$\sqrt[\mathrm{q}]{{\sum }_{\mathrm{j}=1}^{\mathrm{m}}{\upomega }_{\mathrm{j}}{\sum }_{\mathrm{i}=1}^{\mathrm{n}}{\uptheta }_{\mathrm{i}}\left(1+{\mathrm{f}}_{{\hat{\mathrm{d}}}_{\mathrm{ij}}}^{\mathrm{q}}\right)+{\sum }_{\mathrm{j}=1}^{\mathrm{m}}{\upomega }_{\mathrm{j}}{\sum }_{\mathrm{i}=1}^{\mathrm{n}}{\uptheta }_{\mathrm{i}}\left(1-{\mathrm{f}}_{{\hat{\mathrm{d}}}_{\mathrm{ij}}}^{\mathrm{q}}\right)}=\sqrt[\mathrm{q}]{2}$$

$$\sqrt[\mathrm{q}]{\prod_{\mathrm{j}=1}^{\mathrm{m}}{\left(\prod_{\mathrm{i}=1}^{\mathrm{n}}{\left(1+{\mathrm{f}}_{{\hat{\mathrm{d}}}_{\mathrm{ij}}}^{\mathrm{q}}\right)}^{{\uptheta }_{\mathrm{i}}}\right)}^{{\upomega }_{\mathrm{j}}}+ \prod_{\mathrm{j}=1}^{\mathrm{m}}{\left(\prod_{\mathrm{i}=1}^{\mathrm{n}}{\left(1-{\mathrm{f}}_{{\hat{\mathrm{d}}}_{\mathrm{ij}}}^{\mathrm{q}}\right)}^{{\uptheta }_{\mathrm{i}}}\right)}^{{\upomega }_{\mathrm{j}}}}\le \sqrt[\mathrm{q}]{2}$$

8 $$\frac{\sqrt[\mathrm{q}]{\prod_{\mathrm{j}=1}^{\mathrm{m}}{\left(\prod_{\mathrm{i}=1}^{\mathrm{n}}{\left(1+{\mathrm{f}}_{{\hat{\mathrm{d}}}_{\mathrm{ij}}}^{\mathrm{q}}\right)}^{{\uptheta }_{\mathrm{i}}}\right)}^{{\upomega }_{\mathrm{j}}}- \prod_{\mathrm{j}=1}^{\mathrm{m}}{\left(\prod_{\mathrm{i}=1}^{\mathrm{n}}{\left(1-{\mathrm{f}}_{{\hat{\mathrm{d}}}_{\mathrm{ij}}}^{\mathrm{q}}\right)}^{{\uptheta }_{\mathrm{i}}}\right)}^{{\upomega }_{\mathrm{j}}}}}{\sqrt[\mathrm{q}]{\prod_{\mathrm{j}=1}^{\mathrm{m}}{\left(\prod_{\mathrm{i}=1}^{\mathrm{n}}{\left(1+{\mathrm{f}}_{{\hat{\mathrm{d}}}_{\mathrm{ij}}}^{\mathrm{q}}\right)}^{{\uptheta }_{\mathrm{i}}}\right)}^{{\upomega }_{\mathrm{j}}}+ \prod_{\mathrm{j}=1}^{\mathrm{m}}{\left(\prod_{\mathrm{i}=1}^{\mathrm{n}}{\left(1-{\mathrm{f}}_{{\hat{\mathrm{d}}}_{\mathrm{ij}}}^{\mathrm{q}}\right)}^{{\uptheta }_{\mathrm{i}}}\right)}^{{\upomega }_{\mathrm{j}}}}}\le \sqrt[\mathrm{q}]{1-\prod_{\mathrm{j}=1}^{\mathrm{m}}{\left(\prod_{\mathrm{i}=1}^{\mathrm{n}}{\left(1-{\mathrm{f}}_{{\hat{\mathrm{d}}}_{\mathrm{ij}}}^{\mathrm{q}}\right)}^{{\uptheta }_{\mathrm{i}}}\right)}^{{\upomega }_{\mathrm{j}}}}$$

Again

$$\begin{aligned} & \sqrt[\mathrm{q}]{\prod_{\mathrm{j}=1}^{\mathrm{m}}{\left(\prod_{\mathrm{i}=1}^{\mathrm{n}}{\left(2-{\mathrm{g}}_{{\hat{\mathrm{d}}}_{\mathrm{ij}}}^{\mathrm{q}}\right)}^{{\uptheta }_{\mathrm{i}}}\right)}^{{\upomega }_{\mathrm{j}}}+ \prod_{\mathrm{j}=1}^{\mathrm{m}}{\left({\prod_{\mathrm{i}=1}^{\mathrm{n}}\left({\mathrm{g}}_{{\hat{\mathrm{d}}}_{\mathrm{ij}}}^{\mathrm{q}}\right)}^{{\uptheta }_{\mathrm{i}}}\right)}^{{\upomega }_{\mathrm{j}}} }\\ &\quad\le \sqrt[\mathrm{q}]{{\sum }_{\mathrm{j}=1}^{\mathrm{m}}{\upomega }_{\mathrm{j}}{\sum }_{\mathrm{i}=1}^{\mathrm{n}}{\uptheta }_{\mathrm{i}}\left(2-{\mathrm{g}}_{{\hat{\mathrm{d}}}_{\mathrm{ij}}}^{\mathrm{q}}\right)+{\sum }_{\mathrm{j}=1}^{\mathrm{m}}{\upomega }_{\mathrm{j}}{\sum }_{\mathrm{i}=1}^{\mathrm{n}}{\uptheta }_{\mathrm{i}}\left({\mathrm{g}}_{{\hat{\mathrm{d}}}_{\mathrm{ij}}}^{\mathrm{q}}\right)}\end{aligned}$$

$$\sqrt[\mathrm{q}]{{\sum }_{\mathrm{j}=1}^{\mathrm{m}}{\upomega }_{\mathrm{j}}{\sum }_{\mathrm{i}=1}^{\mathrm{n}}{\uptheta }_{\mathrm{i}}\left(2-{\mathrm{g}}_{{\hat{\mathrm{d}}}_{\mathrm{ij}}}^{\mathrm{q}}\right)+{\sum }_{\mathrm{j}=1}^{\mathrm{m}}{\upomega }_{\mathrm{j}}{\sum }_{\mathrm{i}=1}^{\mathrm{n}}{\uptheta }_{\mathrm{i}}\left({\mathrm{g}}_{{\hat{\mathrm{d}}}_{\mathrm{ij}}}^{\mathrm{q}}\right)}\le \sqrt[\mathrm{q}]{2}$$

$$\sqrt[\mathrm{q}]{\prod_{\mathrm{j}=1}^{\mathrm{m}}{\left(\prod_{\mathrm{i}=1}^{\mathrm{n}}{\left(2-{\mathrm{g}}_{{\hat{\mathrm{d}}}_{\mathrm{ij}}}^{\mathrm{q}}\right)}^{{\uptheta }_{\mathrm{i}}}\right)}^{{\upomega }_{\mathrm{j}}}+ \prod_{\mathrm{j}=1}^{\mathrm{m}}{\left({\prod_{\mathrm{i}=1}^{\mathrm{n}}\left({\mathrm{g}}_{{\hat{\mathrm{d}}}_{\mathrm{ij}}}^{\mathrm{q}}\right)}^{{\uptheta }_{\mathrm{i}}}\right)}^{{\upomega }_{\mathrm{j}}}}\le \sqrt[\mathrm{q}]{2}$$

9 $$\frac{\sqrt[\mathrm{q}]{2\prod_{\mathrm{j}=1}^{\mathrm{m}}{\left({\prod_{\mathrm{i}=1}^{\mathrm{n}}\left({\mathrm{g}}_{{\hat{\mathrm{d}}}_{\mathrm{ij}}}^{\mathrm{q}}\right)}^{{\uptheta }_{\mathrm{i}}}\right)}^{{\upomega }_{\mathrm{j}}}} }{\sqrt[\mathrm{q}]{\prod_{\mathrm{j}=1}^{\mathrm{m}}{\left(\prod_{\mathrm{i}=1}^{\mathrm{n}}{\left(2-{\mathrm{g}}_{{\hat{\mathrm{d}}}_{\mathrm{ij}}}^{\mathrm{q}}\right)}^{{\uptheta }_{\mathrm{i}}}\right)}^{{\upomega }_{\mathrm{j}}}+ \prod_{\mathrm{j}=1}^{\mathrm{m}}{\left({\prod_{\mathrm{i}=1}^{\mathrm{n}}\left({\mathrm{g}}_{{\hat{\mathrm{d}}}_{\mathrm{ij}}}^{\mathrm{q}}\right)}^{{\uptheta }_{\mathrm{i}}}\right)}^{{\upomega }_{\mathrm{j}}}}}\ge \prod_{\mathrm{j}=1}^{\mathrm{m}}\begin{array}{c}{\left({\prod_{\mathrm{i}=1}^{\mathrm{n}}\left({\mathrm{g}}_{{\hat{\mathrm{d}}}_{\mathrm{ij}}}\right)}^{{\uptheta }_{\mathrm{i}}}\right)}^{{\upomega }_{\mathrm{j}}}\end{array}$$

Let $\mathrm{q}-\mathrm{ROFHSWA}\left({\mathrm{J}}_{{\hat{\mathrm{d}}}_{11}}, {\mathrm{J}}_{{\hat{\mathrm{d}}}_{12}}, \dots ,{\mathrm{J}}_{{\hat{\mathrm{d}}}_{\mathrm{nm}}}\right)={\mathrm{J}}_{{\hat{\mathrm{d}}}_{\mathrm{k}}}=\left({\mathrm{f}}_{{\mathrm{J}}_{{\hat{\mathrm{d}}}_{\mathrm{k}}}}, {\mathrm{g}}_{{\mathrm{J}}_{{\hat{\mathrm{d}}}_{\mathrm{k}}}}\right)$ and $\mathrm{q}-\mathrm{ROFHSEWA }\left({\mathrm{J}}_{{\hat{\mathrm{d}}}_{11}}, {\mathrm{J}}_{{\hat{\mathrm{d}}}_{12}}, \dots ,{\mathrm{J}}_{{\hat{\mathrm{d}}}_{\mathrm{nm}}}\right)={\mathrm{J}}_{{\hat{\mathrm{d}}}_{\mathrm{k}}}^{\mathrm{\upepsilon }}=\left({\mathrm{f}}_{{\mathrm{J}}_{{\hat{\mathrm{d}}}_{\mathrm{k}}}^{\mathrm{\upepsilon }}},{\mathrm{g}}_{{\mathrm{J}}_{{\hat{\mathrm{d}}}_{\mathrm{k}}}^{\mathrm{\upepsilon }}}\right)$. Then the inequalities (i) and (ii) can be transformed into the forms of ${\mathrm{f}}_{{\mathrm{J}}_{{\hat{\mathrm{d}}}_{\mathrm{k}}}}\ge {\mathrm{f}}_{{\mathrm{J}}_{{\hat{\mathrm{d}}}_{\mathrm{k}}}^{\mathrm{\upepsilon }}}$ and also the ${\mathrm{g}}_{{\mathrm{J}}_{{\hat{\mathrm{d}}}_{\mathrm{k}}}}\le {\mathrm{g}}_{{\mathrm{J}}_{{\hat{\mathrm{d}}}_{\mathrm{k}}}^{\mathrm{\upepsilon }}}$ respectively. So,$\mathrm{S}\left({\mathrm{J}}_{{\hat{\mathrm{d}}}_{\mathrm{k}}}\right)={\mathrm{f}}_{{\hat{\mathrm{d}}}_{\mathrm{ij}}}^{\mathrm{q}}-{\mathrm{g}}_{{\hat{\mathrm{d}}}_{\mathrm{ij}}}^{\mathrm{q}}+\left(\frac{{\mathrm{e}}^{{\mathrm{f}}_{{\hat{\mathrm{d}}}_{\mathrm{ij}}}^{\mathrm{q}}-{\mathrm{g}}_{{\hat{\mathrm{d}}}_{\mathrm{ij}}}^{\mathrm{q}}}}{{\mathrm{e}}^{{\mathrm{f}}_{{\hat{\mathrm{d}}}_{\mathrm{ij}}}^{\mathrm{q}}-{\mathrm{g}}_{{\hat{\mathrm{d}}}_{\mathrm{ij}}}^{\mathrm{q}}}+1}-\frac{1}{2}\right){\mathrm{\beth }}_{{\mathrm{J}}_{{\hat{\mathrm{d}}}_{\mathrm{ij}}}}^{\mathrm{q}}\le {\mathrm{f}}_{{\hat{\mathrm{d}}}_{\mathrm{ij}}^{\upvarepsilon }}^{\mathrm{q}}-{\mathrm{g}}_{{\hat{\mathrm{d}}}_{\mathrm{ij}}^{\upvarepsilon }}^{\mathrm{q}}+\left(\frac{{\mathrm{e}}^{{\mathrm{f}}_{{\hat{\mathrm{d}}}_{\mathrm{ij}}^{\upvarepsilon }}^{\mathrm{q}}-{\mathrm{g}}_{{\hat{\mathrm{d}}}_{\mathrm{ij}}^{\upvarepsilon }}^{\mathrm{q}}}}{{\mathrm{e}}^{{\mathrm{f}}_{{\hat{\mathrm{d}}}_{\mathrm{ij}}^{\upvarepsilon }}^{\mathrm{q}}-{\mathrm{g}}_{{\hat{\mathrm{d}}}_{\mathrm{ij}}^{\upvarepsilon }}^{\mathrm{q}}}+1}-\frac{1}{2}\right){\mathrm{\beth }}_{{\mathrm{J}}_{{\hat{\mathrm{d}}}_{\mathrm{ij}}^{\upvarepsilon }}}^{\mathrm{q}}=\mathrm{S}\left({\mathrm{J}}_{{\hat{\mathrm{d}}}_{\mathrm{k}}}^{\mathrm{\upepsilon }}\right).$

If $$\mathrm{S}\left({\mathrm{J}}_{{\hat{\mathrm{d}}}_{\mathrm{k}}}\right)>\mathrm{S}\left({\mathrm{J}}_{{\hat{\mathrm{d}}}_{\mathrm{k}}}^{\mathrm{\upepsilon }}\right)$$, then

11 $$\mathrm{q}-\mathrm{ROFHSWA}\left({\mathrm{J}}_{{\hat{\mathrm{d}}}_{11}}, {\mathrm{J}}_{{\hat{\mathrm{d}}}_{12}}, \dots ,{\mathrm{J}}_{{\hat{\mathrm{d}}}_{\mathrm{nm}}}\right)>\mathrm{q}-\mathrm{ROFHSEWA}\left({\mathrm{J}}_{{\hat{\mathrm{d}}}_{11}}, {\mathrm{J}}_{{\hat{\mathrm{d}}}_{12}}, \dots ,{\mathrm{J}}_{{\hat{\mathrm{d}}}_{\mathrm{nm}}}\right).$$

If $$\mathrm{S}\left({\mathrm{J}}_{{\hat{\mathrm{d}}}_{\mathrm{k}}}\right)=\mathrm{S}\left({\mathrm{J}}_{{\hat{\mathrm{d}}}_{\mathrm{k}}}^{\mathrm{\upepsilon }}\right)$$, then

12 $$\mathrm{q}-\mathrm{ROFHSWA}\left({\mathrm{J}}_{{\hat{\mathrm{d}}}_{11}}, {\mathrm{J}}_{{\hat{\mathrm{d}}}_{12}}, \dots ,{\mathrm{J}}_{{\hat{\mathrm{d}}}_{\mathrm{nm}}}\right)=\mathrm{ q}-\mathrm{ROFHSEWA }\left({\mathrm{J}}_{{\hat{\mathrm{d}}}_{11}}, {\mathrm{J}}_{{\hat{\mathrm{d}}}_{12}}, \dots ,{\mathrm{J}}_{{\hat{\mathrm{d}}}_{\mathrm{nm}}}\right).$$

From (11) and (12), we get

$$\mathrm{q}-\mathrm{ROFHSWA }\left({\mathrm{J}}_{{\hat{\mathrm{d}}}_{11}}, {\mathrm{J}}_{{\hat{\mathrm{d}}}_{12}}, \dots ,{\mathrm{J}}_{{\hat{\mathrm{d}}}_{\mathrm{nm}}}\right)\ge \mathrm{q}-\mathrm{ROFHSEWA }\left({\mathrm{J}}_{{\hat{\mathrm{d}}}_{11}}, {\mathrm{J}}_{{\hat{\mathrm{d}}}_{12}}, \dots ,{\mathrm{J}}_{{\hat{\mathrm{d}}}_{\mathrm{nm}}}\right).$$

### Example

Let $\mathrm{H}={\{\mathrm{H}}_{1}, {\mathrm{H}}_{2}, {\mathrm{H}}_{3}\}$ be a team of professionals with the most appropriate weighted vectors ${\uptheta }_{\mathrm{i}}={\left(0.3, 0.4, 0.3\right) }^{\mathrm{T}}$. The team of experts decided to buy a house under the set of attributes which are $\mathrm{A}=\left\{{\mathrm{d}}_{1}=\mathrm{infrastructure},{\mathrm{d}}_{2}=\mathrm{facilities},{\mathrm{d}}_{3}=\mathrm{sewerage system}, {\mathrm{d}}_{4}=\mathrm{security}\right\}$. For the selection of house, the team of experts considered the multi sub-attributes of the deliberated parameters such as $\left\{{\mathrm{d}}_{1}=\mathrm{Infrastructure}= \left\{{\mathrm{d}}_{11}=\mathrm{old style}, {\mathrm{d}}_{11}=\mathrm{new style}\right\} ,{\mathrm{d}}_{2}=\mathrm{Facilities}=\left\{{\mathrm{d}}_{21}=\mathrm{hospital}, {\mathrm{d}}_{22}=\mathrm{school}\right\} ,{\mathrm{d}}_{3}=\mathrm{Sewerage system}= \left\{{\mathrm{d}}_{31}=\mathrm{excellent}\right\}, {\mathrm{d}}_{4}=\mathrm{Secuirty}= \left\{{\mathrm{d}}_{41}=\mathrm{excellent}\right\}\right\}$. Let ${\mathfrak{L}}^{{^{\prime}}}={\mathrm{d}}_{1}\times {\mathrm{d}}_{2}\times {\mathrm{d}}_{3}\times {\mathrm{d}}_{4}$ represents the collection of multi-sub-attributes. $${\mathfrak{L}}^{{^{\prime}}}={\mathrm{d}}_{1}\times {\mathrm{d}}_{2}\times {\mathrm{d}}_{3}\times {\mathrm{d}}_{4}=\left\{{\mathrm{d}}_{11}, {\mathrm{d}}_{12}\right\}\times \left\{{\mathrm{d}}_{21}, {\mathrm{d}}_{22}\right\}\times \left\{{\mathrm{d}}_{31}\right\}\times \left\{{\mathrm{d}}_{42}\right\}=\left\{\begin{array}{c}\left({\mathrm{d}}_{11}, {\mathrm{d}}_{21}, {\mathrm{d}}_{31}, {\mathrm{d}}_{41}\right), \left({\mathrm{d}}_{11}, {\mathrm{d}}_{22}, {\mathrm{d}}_{31}, {\mathrm{d}}_{41}\right), \\ \left({\mathrm{d}}_{12}, {\mathrm{d}}_{21}, {\mathrm{d}}_{31}, {\mathrm{d}}_{41}\right), \left({\mathrm{d}}_{12}, {\mathrm{d}}_{22}, {\mathrm{d}}_{31}, {\mathrm{d}}_{41}\right)\end{array}\right\}=\left\{{\hat{\mathrm{d}}}_{1},{\hat{\mathrm{d}}}_{2}, {\hat{\mathrm{d}}}_{3}, {\hat{\mathrm{d}}}_{4}\right\}$$ describes the sub-attributes collection with weights ${\upomega }_{\mathrm{j}}={\left(0.2, 0.3, 0.4, 0.1\right)}^{\mathrm{T}}$. The team of experts assumes rating values for each sub-attribute in the q-ROFHSNs form: $\left({\mathrm{J}}_{3\times 4} ,{\mathfrak{L}}^{{^{\prime}}}\right)={\left({\mathrm{f}}_{{\hat{\mathrm{d}}}_{\mathrm{ij}}},{\mathrm{g}}_{{\hat{\mathrm{d}}}_{\mathrm{ij}}}\right)}_{3\times 4}$ are given as follows:

$$\left({\mathrm{J}}_{3\times 4}, {\mathfrak{L}}^{{^{\prime}}}\right)=\left[\begin{array}{cccc}\left(0.5, 0.3\right)& \left(0.8, 0.7\right)& \left(0.6, 0.3\right)& \left(0.2, 0.9\right)\\ \left(0.6, 0.3\right)& \left(0.4, 0.7\right)& \left(0.4, 0.5\right)& \left(0.5, 0.6\right)\\ \left(0.3, 0.4\right)& \left(0.6, 0.8\right)& \left(0.3, 0.9\right)& \left(0.2, 0.7\right)\end{array}\right]$$

$$\mathrm{q}-\mathrm{ROFHSWA}\left({\mathrm{J}}_{{\hat{\mathrm{d}}}_{11}}, {\mathrm{J}}_{{\hat{\mathrm{d}}}_{12}}, \dots ,{\mathrm{J}}_{{\hat{\mathrm{d}}}_{\mathrm{nm}}}\right)=\Bigg\langle \sqrt[3]{1-\prod_{\mathrm{j}=1}^{4}{\left(\prod_{\mathrm{i}=1}^{3}{\left(1-{\mathrm{f}}_{{\hat{\mathrm{d}}}_{\mathrm{ij}}}^{3}\right)}^{{\uptheta }_{\mathrm{i}}}\right)}^{{\upomega }_{\mathrm{j}}}}, \prod_{\mathrm{j}=1}^{4}\begin{array}{c}{\left({\prod_{\mathrm{i}=1}^{3}\left({\mathrm{g}}_{{\hat{\mathrm{d}}}_{\mathrm{ij}}}\right)}^{{\uptheta }_{\mathrm{i}}}\right)}^{{\upomega }_{\mathrm{j}}}\end{array}\Bigg\rangle$$

$$=\Bigg\langle \begin{array}{c}\sqrt[3]{1-\left[\begin{array}{c}{\left\{\left(0.9607\right)\left(0.9072\right)\left(0.9918\right)\right\}}^{0.2}{\left\{\left(0.8063\right)\left(0.9739\right)\left(0.9296\right)\right\}}^{0.3}\\ {\left\{\left(0.9804\right)\left(0.9739\right)\left(0.9918\right)\right\}}^{0.4}{\left\{\left(0.9976\right)\left(0.9479\right)\left(0.9976\right)\right\}}^{0.1}\end{array}\right]}, \\ \left[\begin{array}{c}{\left\{\left(0.6968\right)\left(0.6178\right)\left(0.7597\right)\right\}}^{0.2}{\left\{\left(0.8985\right)\left(0.8670\right)\left(0.9352\right)\right\}}^{0.3}\\ {\left\{\left(0.6968\right)\left(0.7579\right)\left(0.9689\right)\right\}}^{0.4}{\left\{\left(0.9689\right)\left(0.8152\right)\left(0.8985\right)\right\}}^{0.1}\end{array}\right]\end{array}\Bigg\rangle$$

$$=\Bigg\langle \begin{array}{c}\sqrt[3]{1-\left[\begin{array}{c}\left(0.9713 \right)\left(0.9099\right)\end{array}\left(0.9785\right)\left(0.9942\right)\right]},\\ \left[\begin{array}{c}\left(0.7997 \right)\left(0.9099\right)\end{array}\left(0.7649\right)\left(0.9663\right)\right]\end{array}\Bigg\rangle$$

$$=\Bigg\langle \begin{array}{c}0.5198, \end{array}0.5375\Bigg\rangle$$

From Examples 3.4 and 3.7, it is proved that

$$\mathrm{q}-\mathrm{ROFHSWA }\left({\mathrm{J}}_{{\hat{\mathrm{d}}}_{11}}, {\mathrm{J}}_{{\hat{\mathrm{d}}}_{12}}, \dots ,{\mathrm{J}}_{{\hat{\mathrm{d}}}_{\mathrm{nm}}}\right)\ge \mathrm{q}-\mathrm{ROFHSEWA}\left({\mathrm{J}}_{{\hat{\mathrm{d}}}_{11}}, {\mathrm{J}}_{{\hat{\mathrm{d}}}_{12}}, \dots ,{\mathrm{J}}_{{\hat{\mathrm{d}}}_{\mathrm{nm}}}\right).$$

### Properties of q-ROFHSEWA operator

#### Idempotency

If $${\mathrm{J}}_{{\hat{\mathrm{d}}}_{\mathrm{ij}}}=\left({\mathrm{f}}_{{\hat{\mathrm{d}}}_{\mathrm{ij}}}, {\mathrm{g}}_{{\hat{\mathrm{d}}}_{\mathrm{ij}}}\right)$$ = $${\mathrm{J}}_{{\hat{\mathrm{d}}}_{\mathrm{k}}}$$ $$\forall$$ $$\mathrm{i},\mathrm{ j}.$$ Then $$\mathrm{q}-\mathrm{ROFHSEWA}\left({\mathrm{J}}_{{\hat{\mathrm{d}}}_{11}}, {\mathrm{J}}_{{\hat{\mathrm{d}}}_{12}}, \dots ,{\mathrm{J}}_{{\hat{\mathrm{d}}}_{\mathrm{nm}}}\right)= {\mathrm{J}}_{{\hat{\mathrm{d}}}_{\mathrm{k}}}$$.

##### Proof

We know that

$$\begin{aligned}\mathrm{q}-\mathrm{ROFHSEWA }\left({\mathrm{J}}_{{\hat{\mathrm{d}}}_{11}}, {\mathrm{J}}_{{\hat{\mathrm{d}}}_{12}}, \dots ,{\mathrm{J}}_{{\hat{\mathrm{d}}}_{\mathrm{nm}}}\right)&=\Bigg\langle \frac{\sqrt[\mathrm{q}]{\prod_{\mathrm{j}=1}^{\mathrm{m}}{\left(\prod_{\mathrm{i}=1}^{\mathrm{n}}{(1+{\mathrm{f}}_{{\hat{\mathrm{d}}}_{\mathrm{ij}}}^{\mathrm{q}})}^{{\uptheta }_{\mathrm{i}}}\right)}^{{\upomega }_{\mathrm{j}}}- \prod_{\mathrm{j}=1}^{\mathrm{m}}{\left(\prod_{\mathrm{i}=1}^{\mathrm{n}}{(1-{\mathrm{f}}_{{\hat{\mathrm{d}}}_{\mathrm{ij}}}^{\mathrm{q}})}^{{\uptheta }_{\mathrm{i}}}\right)}^{{\upomega }_{\mathrm{j}}}}}{\sqrt[\mathrm{q}]{\prod_{\mathrm{j}=1}^{\mathrm{m}}{\left(\prod_{\mathrm{i}=1}^{\mathrm{n}}{(1+{\mathrm{f}}_{{\hat{\mathrm{d}}}_{\mathrm{ij}}}^{\mathrm{q}})}^{{\uptheta }_{\mathrm{i}}}\right)}^{{\upomega }_{\mathrm{j}}}+ \prod_{\mathrm{j}=1}^{\mathrm{m}}{\left(\prod_{\mathrm{i}=1}^{\mathrm{n}}{(1-{\mathrm{f}}_{{\hat{\mathrm{d}}}_{\mathrm{ij}}}^{\mathrm{q}})}^{{\uptheta }_{\mathrm{i}}}\right)}^{{\upomega }_{\mathrm{j}}}}},\\&\quad\frac{\sqrt[\mathrm{q}]{2\prod_{\mathrm{j}=1}^{\mathrm{m}}{\left({\prod_{\mathrm{i}=1}^{\mathrm{n}}({\mathrm{g}}_{{\hat{\mathrm{d}}}_{\mathrm{ij}}}^{\mathrm{q}})}^{{\uptheta }_{\mathrm{i}}}\right)}^{{\upomega }_{\mathrm{j}}}}}{\sqrt[\mathrm{q}]{\prod_{\mathrm{j}=1}^{\mathrm{m}}{\left(\prod_{\mathrm{i}=1}^{\mathrm{n}}{(2-{\mathrm{g}}_{{\hat{\mathrm{d}}}_{\mathrm{ij}}}^{\mathrm{q}})}^{{\uptheta }_{\mathrm{i}}}\right)}^{{\upomega }_{\mathrm{j}}}+ \prod_{\mathrm{j}=1}^{\mathrm{m}}{\left({\prod_{\mathrm{i}=1}^{\mathrm{n}}({\mathrm{g}}_{{\hat{\mathrm{d}}}_{\mathrm{ij}}}^{\mathrm{q}})}^{{\uptheta }_{\mathrm{i}}}\right)}^{{\upomega }_{\mathrm{j}}}}}\Bigg\rangle \end{aligned}$$

$$=\Bigg\langle \frac{\sqrt[\mathrm{q}]{{\left({\left(1+{\mathrm{f}}_{{\hat{\mathrm{d}}}_{\mathrm{ij}}}^{\mathrm{q}}\right)}^{{\sum }_{\mathrm{i}=1}^{\mathrm{n}}{\uptheta }_{\mathrm{i}}}\right)}^{{\sum }_{\mathrm{j}=1}^{\mathrm{m}}{\upomega }_{\mathrm{j}}}- {\left({\left(1-{\mathrm{f}}_{{\hat{\mathrm{d}}}_{\mathrm{ij}}}^{\mathrm{q}}\right)}^{{\sum }_{\mathrm{i}=1}^{\mathrm{n}}{\uptheta }_{\mathrm{i}}}\right)}^{{\sum }_{\mathrm{j}=1}^{\mathrm{m}}{\upomega }_{\mathrm{j}}}}}{\sqrt[\mathrm{q}]{{\left({\left(1+{\mathrm{f}}_{{\hat{\mathrm{d}}}_{\mathrm{ij}}}^{\mathrm{q}}\right)}^{{\sum }_{\mathrm{i}=1}^{\mathrm{n}}{\uptheta }_{\mathrm{i}}}\right)}^{{\sum }_{\mathrm{j}=1}^{\mathrm{m}}{\upomega }_{\mathrm{j}}}+ {\left({\left(1-{\mathrm{f}}_{{\hat{\mathrm{d}}}_{\mathrm{ij}}}^{\mathrm{q}}\right)}^{{\sum }_{\mathrm{i}=1}^{\mathrm{n}}{\uptheta }_{\mathrm{i}}}\right)}^{{\sum }_{\mathrm{j}=1}^{\mathrm{m}}{\upomega }_{\mathrm{j}}}}}, \frac{\sqrt[\mathrm{q}]{2{\left({\left({\mathrm{g}}_{{\hat{\mathrm{d}}}_{\mathrm{ij}}}^{\mathrm{q}}\right)}^{{\sum }_{\mathrm{i}=1}^{\mathrm{n}}{\uptheta }_{\mathrm{i}}}\right)}^{{\sum }_{\mathrm{j}=1}^{\mathrm{m}}{\upomega }_{\mathrm{j}}}}}{\sqrt[\mathrm{q}]{\left({\left(2-{\mathrm{g}}_{{\hat{\mathrm{d}}}_{\mathrm{ij}}}^{\mathrm{q}}\right)}^{{\sum }_{\mathrm{i}=1}^{\mathrm{n}}{\uptheta }_{\mathrm{i}}}\right) + {\left({\left({\mathrm{g}}_{{\hat{\mathrm{d}}}_{\mathrm{ij}}}^{\mathrm{q}}\right)}^{{\sum }_{\mathrm{i}=1}^{\mathrm{n}}{\uptheta }_{\mathrm{i}}}\right)}^{{\sum }_{\mathrm{j}=1}^{\mathrm{m}}{\upomega }_{\mathrm{j}}}}}\Bigg\rangle$$

$$=\Bigg\langle \frac{\sqrt[\mathrm{q}]{\begin{array}{c}\left(1+{\mathrm{f}}_{{\hat{\mathrm{d}}}_{\mathrm{ij}}}^{\mathrm{q}}\right)-\left(1-{\mathrm{f}}_{{\hat{\mathrm{d}}}_{\mathrm{ij}}}^{\mathrm{q}}\right)\end{array}}}{\sqrt[\mathrm{q}]{\begin{array}{c}\left(1+{\mathrm{f}}_{{\hat{\mathrm{d}}}_{\mathrm{ij}}}^{\mathrm{q}}\right)+\left(1-{\mathrm{f}}_{{\hat{\mathrm{d}}}_{\mathrm{ij}}}^{\mathrm{q}}\right)\end{array}}}, \frac{\sqrt[\mathrm{q}]{2\left({\mathrm{g}}_{{\hat{\mathrm{d}}}_{\mathrm{ij}}}^{\mathrm{q}}\right)}}{\sqrt[\mathrm{q}]{\left(2-{\mathrm{g}}_{{\hat{\mathrm{d}}}_{\mathrm{ij}}}^{\mathrm{q}}\right)+\left({\mathrm{g}}_{{\hat{\mathrm{d}}}_{\mathrm{ij}}}^{\mathrm{q}}\right)}}\Bigg\rangle$$

$$=\Bigg\langle {\mathrm{f}}_{{\hat{\mathrm{d}}}_{\mathrm{ij}} }, {\mathrm{g}}_{{\hat{\mathrm{d}}}_{\mathrm{ij}}}\Bigg\rangle ={\mathrm{J}}_{\begin{array}{c}{\hat{\mathrm{d}}}_{\mathrm{ij}}\end{array}}$$

#### Boundedness

Let ${\mathrm{J}}_{{\hat{\mathrm{d}}}_{\mathrm{ij}} }= \left({\mathrm{f}}_{{\hat{\mathrm{d}}}_{\mathrm{ij}} }, {\mathrm{g}}_{{\hat{\mathrm{d}}}_{\mathrm{ij}}}\right)$ be a collection of q-ROFHSNs and ${\mathrm{J}}_{\mathrm{min}}={\mathrm{J}}_{{\hat{\mathrm{d}}}_{\mathrm{ij}}\mathrm{ min}}$ $={\mathrm{J}}_{\mathrm{max}}={\mathrm{J}}_{{\hat{\mathrm{d}}}_{\mathrm{ij}}\mathrm{ max}}$. Then

$${\mathrm{J}}_{{\hat{\mathrm{d}}}_{\mathrm{ij}}\mathrm{ min}}\le \mathrm{ q}-\mathrm{ROFHSEWA}\left({\mathrm{J}}_{{\hat{\mathrm{d}}}_{11}}, {\mathrm{J}}_{{\hat{\mathrm{d}}}_{12}}, \dots ,{\mathrm{J}}_{{\hat{\mathrm{d}}}_{\mathrm{nm}}}\right)\le {\mathrm{J}}_{{\hat{\mathrm{d}}}_{\mathrm{ij}}\mathrm{ max}}.$$

##### Proof

Let $\mathrm{h}\left(\mathrm{x}\right)=\sqrt[\mathrm{q}]{\frac{1-{\mathrm{x}}^{\mathrm{q}}}{1+{\mathrm{x}}^{\mathrm{q}}}}$, $\mathrm{x}\in \left[0, 1\right]$, then $\frac{\mathrm{d}}{\mathrm{d}\left(\mathrm{y}\right)}\mathrm{h}\left(\mathrm{x}\right)=-\frac{1}{\mathrm{q}}{\left(\frac{1-{\mathrm{x}}^{\mathrm{q}}}{1+{\mathrm{x}}^{\mathrm{q}}}\right)}^{\frac{1}{\mathrm{q}}-1}\left\{\frac{{\mathrm{qx}}^{2\mathrm{q}-1}+{\mathrm{qx}}^{2\mathrm{q}-1}}{{\left(1+{\mathrm{x}}^{\mathrm{q}}\right)}^{2}}\right\}$ < 0, so $\mathrm{h}(\mathrm{x})$ is decreasing function on $\left[0, 1\right]$. So,

$${\mathrm{f}}_{{\hat{\mathrm{d}}}_{\mathrm{ij}}\mathrm{min}}\le {\mathrm{f}}_{{\hat{\mathrm{d}}}_{\mathrm{ij}}} \le {\mathrm{f}}_{{\hat{\mathrm{d}}}_{\mathrm{ij}}\mathrm{max}}. \mathrm{Hence},\mathrm{ h } \left({\mathrm{f}}_{{\hat{\mathrm{d}}}_{\mathrm{ij}}\mathrm{max}}\right)\le \mathrm{h}\left({\mathrm{f}}_{{\hat{\mathrm{d}}}_{\mathrm{ij}}}\right) \le \mathrm{h}\left({\mathrm{f}}_{{\hat{\mathrm{d}}}_{\mathrm{ij}}\mathrm{min}}\right)$$

$$\begin{aligned} & \sqrt[\mathrm{q}]{{\prod }_{\mathrm{j}=1}^{\mathrm{m}}{\left({\prod }_{\mathrm{i}=1}^{\mathrm{n}}{\left(\frac{1-{\mathrm{f}}_{{\hat{\mathrm{d}}}_{\mathrm{ij}}\mathrm{max}}^{\mathrm{q}}}{1+{\mathrm{f}}_{{\hat{\mathrm{d}}}_{\mathrm{ij}}\mathrm{max}}^{\mathrm{q}}}\right)}^{{\uptheta }_{\mathrm{i}}}\right)}^{{\upomega }_{\mathrm{j}}}}\le \sqrt[\mathrm{q}]{{\prod }_{\mathrm{j}=1}^{\mathrm{m}}{\left({\prod }_{\mathrm{i}=1}^{\mathrm{n}}{\left(\frac{1-{\mathrm{f}}_{{\hat{\mathrm{d}}}_{\mathrm{ij}}}^{\mathrm{q}}}{1+{\mathrm{f}}_{{\hat{\mathrm{d}}}_{\mathrm{ij}}}^{\mathrm{q}}}\right)}^{{\uptheta }_{\mathrm{i}}}\right)}^{{\upomega }_{\mathrm{j}}}} \\ &\quad \le \sqrt[\mathrm{q}]{{\prod }_{\mathrm{j}=1}^{\mathrm{m}}{\left({\prod }_{\mathrm{i}=1}^{\mathrm{n}}{\left(\frac{1-{\mathrm{f}}_{{\hat{\mathrm{d}}}_{\mathrm{ij}}\mathrm{min}}^{\mathrm{q}}}{1+{\mathrm{f}}_{{\hat{\mathrm{d}}}_{\mathrm{ij}}\mathrm{min}}^{\mathrm{q}}}\right)}^{{\uptheta }_{\mathrm{i}}}\right)}^{{\upomega }_{\mathrm{j}}}}\end{aligned}$$

$$\begin{aligned} & \sqrt[\mathrm{q}]{{\left({\left(\frac{1-{\mathrm{f}}_{{\hat{\mathrm{d}}}_{\mathrm{ij}}\mathrm{max}}^{\mathrm{q}}}{1+{\mathrm{f}}_{{\hat{\mathrm{d}}}_{\mathrm{ij}}\mathrm{max}}^{\mathrm{q}}}\right)}^{\sum_{\mathrm{i}=1}^{\mathrm{n}}{\uptheta }_{\mathrm{i}}}\right)}^{{\sum }_{\mathrm{j}=1}^{\mathrm{m}}{\upomega }_{\mathrm{j}}} }\le \sqrt[\mathrm{q}]{{\prod }_{\mathrm{j}=1}^{\mathrm{m}}{\left({\prod }_{\mathrm{i}=1}^{\mathrm{n}}{\left(\frac{1-{\mathrm{f}}_{{\hat{\mathrm{d}}}_{\mathrm{ij}}}^{\mathrm{q}}}{1+{\mathrm{f}}_{{\hat{\mathrm{d}}}_{\mathrm{ij}}}^{\mathrm{q}}}\right)}^{{\uptheta }_{\mathrm{i}}}\right)}^{{\upomega }_{\mathrm{j}}}}\\ &\quad\le \sqrt[\mathrm{q}]{{\left({\left(\frac{1-{\mathrm{f}}_{{\hat{\mathrm{d}}}_{\mathrm{ij}}\mathrm{min}}^{\mathrm{q}}}{1+{\mathrm{f}}_{{\hat{\mathrm{d}}}_{\mathrm{ij}}\mathrm{min}}^{\mathrm{q}}}\right)}^{\sum_{\mathrm{i}=1}^{\mathrm{n}}{\uptheta }_{\mathrm{i}}}\right)}^{{\sum }_{\mathrm{j}=1}^{\mathrm{m}}{\upomega }_{\mathrm{j}}}}\end{aligned}$$

$$\sqrt[\mathrm{q}]{1+\left(\frac{1-{\mathrm{f}}_{{\hat{\mathrm{d}}}_{\mathrm{ij}}\mathrm{max}}^{\mathrm{q}}}{1+{\mathrm{f}}_{{\hat{\mathrm{d}}}_{\mathrm{ij}}\mathrm{max}}^{\mathrm{q}}}\right) }\le \sqrt[\mathrm{q}]{1+{\prod }_{\mathrm{j}=1}^{\mathrm{m}}{\left({\prod }_{\mathrm{i}=1}^{\mathrm{n}}{\left(\frac{1-{\mathrm{f}}_{{\hat{\mathrm{d}}}_{\mathrm{ij}}}^{\mathrm{q}}}{1+{\mathrm{f}}_{{\hat{\mathrm{d}}}_{\mathrm{ij}}}^{\mathrm{q}}}\right)}^{{\uptheta }_{\mathrm{i}}}\right)}^{{\upomega }_{\mathrm{j}}}} \le \sqrt[\mathrm{q}]{1+\left(\frac{1-{\mathrm{f}}_{{\hat{\mathrm{d}}}_{\mathrm{ij}}\mathrm{min}}^{\mathrm{q}}}{1+{\mathrm{f}}_{{\hat{\mathrm{d}}}_{\mathrm{ij}}\mathrm{min}}^{\mathrm{q}}}\right)}$$

$$\sqrt[\mathrm{q}]{\frac{2}{1+{\mathrm{f}}_{{\hat{\mathrm{d}}}_{\mathrm{ij}}\mathrm{max}}^{\mathrm{q}}}} \le \sqrt[\mathrm{q}]{1+{\prod }_{\mathrm{j}=1}^{\mathrm{m}}{\left({\prod }_{\mathrm{i}=1}^{\mathrm{n}}{\left(\frac{1-{\mathrm{f}}_{{\hat{\mathrm{d}}}_{\mathrm{ij}}}^{\mathrm{q}}}{1+{\mathrm{f}}_{{\hat{\mathrm{d}}}_{\mathrm{ij}}}^{\mathrm{q}}}\right)}^{{\uptheta }_{\mathrm{i}}}\right)}^{{\upomega }_{\mathrm{j}}} } \le \sqrt[\mathrm{q}]{\frac{2}{1+{\mathrm{f}}_{{\hat{\mathrm{d}}}_{\mathrm{ij}}\mathrm{min}}^{\mathrm{q}}}}$$

$$\sqrt[\mathrm{q}]{\frac{1+{\mathrm{f}}_{{\hat{\mathrm{d}}}_{\mathrm{ij}}\mathrm{min}}^{\mathrm{q}}}{2}} \le \sqrt[\mathrm{q}]{\frac{1}{1+{\prod }_{\mathrm{j}=1}^{\mathrm{m}}{\left({\prod }_{\mathrm{i}=1}^{\mathrm{n}}{\left(\frac{1-{\mathrm{f}}_{{\hat{\mathrm{d}}}_{\mathrm{ij}}}^{\mathrm{q}}}{1+{\mathrm{f}}_{{\hat{\mathrm{d}}}_{\mathrm{ij}}}^{\mathrm{q}}}\right)}^{{\uptheta }_{\mathrm{i}}}\right)}^{{\upomega }_{\mathrm{j}}}}}\le \sqrt[\mathrm{q}]{\frac{1+{\mathrm{f}}_{{\hat{\mathrm{d}}}_{\mathrm{ij}}\mathrm{max}}^{\mathrm{q}}}{2}}$$

$$\sqrt[\mathrm{q}]{1+{\mathrm{f}}_{{\hat{\mathrm{d}}}_{\mathrm{ij}}\mathrm{min}}^{\mathrm{q}}}\le \sqrt[\mathrm{q}]{\frac{2}{1+{\prod }_{\mathrm{j}=1}^{\mathrm{m}}{\left({\prod }_{\mathrm{i}=1}^{\mathrm{n}}{\left(\frac{1-{\mathrm{f}}_{{\hat{\mathrm{d}}}_{\mathrm{ij}}}^{\mathrm{q}}}{1+{\mathrm{f}}_{{\hat{\mathrm{d}}}_{\mathrm{ij}}}^{\mathrm{q}}}\right)}^{{\uptheta }_{\mathrm{i}}}\right)}^{{\upomega }_{\mathrm{j}}}}}\le \sqrt[\mathrm{q}]{1+{\mathrm{f}}_{{\hat{\mathrm{d}}}_{\mathrm{ij}}\mathrm{max}}^{\mathrm{q}}}$$

$$\sqrt[\mathrm{q}]{{\mathrm{f}}_{{\hat{\mathrm{d}}}_{\mathrm{ij}}\mathrm{min}}^{\mathrm{q}}}\le \sqrt[\mathrm{q}]{\frac{2}{1+{\prod }_{\mathrm{j}=1}^{\mathrm{m}}{\left({\prod }_{\mathrm{i}=1}^{\mathrm{n}}{\left(\frac{1-{\mathrm{f}}_{{\hat{\mathrm{d}}}_{\mathrm{ij}}}^{\mathrm{q}}}{1+{\mathrm{f}}_{{\hat{\mathrm{d}}}_{\mathrm{ij}}}^{\mathrm{q}}}\right)}^{{\uptheta }_{\mathrm{i}}}\right)}^{{\upomega }_{\mathrm{j}}}}-1}\le \sqrt[\mathrm{q}]{{\mathrm{f}}_{{\hat{\mathrm{d}}}_{\mathrm{ij}}\mathrm{max}}^{\mathrm{q}}}$$

13 $${\mathrm{f}}_{{\hat{\mathrm{d}}}_{\mathrm{ij}}\mathrm{min}}\le \sqrt[\mathrm{q}]{\frac{{\prod }_{\mathrm{j}=1}^{\mathrm{m}}{\left({\prod }_{\mathrm{i}=1}^{\mathrm{n}}{\left(1+{\mathrm{f}}_{{\hat{\mathrm{d}}}_{\mathrm{ij}}}^{\mathrm{q}}\right)}^{{\uptheta }_{\mathrm{i}}}\right)}^{{\upomega }_{\mathrm{j}}}-{\prod }_{\mathrm{j}=1}^{\mathrm{m}}{\left({\prod }_{\mathrm{i}=1}^{\mathrm{n}}{\left(1-{\mathrm{f}}_{{\hat{\mathrm{d}}}_{\mathrm{ij}}}^{\mathrm{q}}\right)}^{{\uptheta }_{\mathrm{i}}}\right)}^{{\upomega }_{\mathrm{j}}}}{{\prod }_{\mathrm{j}=1}^{\mathrm{m}}{\left({\prod }_{\mathrm{i}=1}^{\mathrm{n}}{\left(1+{\mathrm{f}}_{{\hat{\mathrm{d}}}_{\mathrm{ij}}}^{\mathrm{q}}\right)}^{{\uptheta }_{\mathrm{i}}}\right)}^{{\upomega }_{\mathrm{j}}}+{\prod }_{\mathrm{j}=1}^{\mathrm{m}}{\left({\prod }_{\mathrm{i}=1}^{\mathrm{n}}{\left(1-{\mathrm{f}}_{{\hat{\mathrm{d}}}_{\mathrm{ij}}}^{\mathrm{q}}\right)}^{{\uptheta }_{\mathrm{i}}}\right)}^{{\upomega }_{\mathrm{j}}}}}\le {\mathrm{f}}_{{\hat{\mathrm{d}}}_{\mathrm{ij}}\mathrm{max}}$$

$${\mathrm{f}}_{{\hat{\mathrm{d}}}_{\mathrm{ij}}\mathrm{min}}\le {\mathrm{f}}_{{\hat{\mathrm{d}}}_{\mathrm{ij}}}\le {\mathrm{f}}_{{\hat{\mathrm{d}}}_{\mathrm{ij}}\mathrm{max}}.$$

Let $\mathrm{k}\left(\mathrm{y}\right)=\sqrt[\mathrm{q}]{\frac{2-{\mathrm{y}}^{\mathrm{q}}}{{\mathrm{y}}^{\mathrm{q}}}}$, $\mathrm{y}\in \left[0, 1\right]$, then $\frac{\mathrm{d}}{\mathrm{dy}}\left(\mathrm{k}(\mathrm{y})\right)=-\frac{1}{\mathrm{q}}{\left(\frac{2-{\mathrm{y}}^{\mathrm{q}}}{{\mathrm{y}}^{\mathrm{q}}}\right)}^{\frac{1}{\mathrm{q}}-1}\left(\frac{2}{{\left({\mathrm{y}}^{\mathrm{q}}\right)}^{2}}\right)$. So, $\frac{\mathrm{d}}{\mathrm{dy}}\left(\mathrm{k}(\mathrm{y})\right)=-\frac{1}{\mathrm{q}}{\left(\frac{2-{\mathrm{y}}^{\mathrm{q}}}{{\mathrm{y}}^{\mathrm{q}}}\right)}^{\frac{1}{\mathrm{q}}-1}\left(\frac{2}{{\left({\mathrm{y}}^{\mathrm{q}}\right)}^{2}}\right)<0$, which shows that $\mathrm{k}(\mathrm{y})$ is decreasing function on $\left[0, 1\right]$. So, ${\mathrm{g}}_{{\hat{\mathrm{d}}}_{\mathrm{ij}}\mathrm{min}}^{\mathrm{q}}\le {\mathrm{g}}_{{\hat{\mathrm{d}}}_{\mathrm{ij}}}\le {\mathrm{g}}_{{\hat{\mathrm{d}}}_{\mathrm{ij}}\mathrm{max}}^{\mathrm{q}}$ $\forall$ $\mathrm{i},\mathrm{j}$. Hence, $\mathrm{k}\left({\mathrm{g}}_{{\hat{\mathrm{d}}}_{\mathrm{ij}}\mathrm{max}}^{\mathrm{q}}\right)\le \mathrm{k}\left({\mathrm{g}}_{{\hat{\mathrm{d}}}_{\mathrm{ij}}}\right)\le \mathrm{k}\left({\mathrm{g}}_{{\hat{\mathrm{d}}}_{\mathrm{ij}}\mathrm{min}}^{\mathrm{q}}\right)$, $\forall$ $\mathrm{i},\mathrm{j}$.

$$\sqrt[\mathrm{q}]{\frac{2-{\mathrm{g}}_{{\hat{\mathrm{d}}}_{\mathrm{ij}}\mathrm{max}}^{\mathrm{q}}}{{\mathrm{g}}_{{\hat{\mathrm{d}}}_{\mathrm{ij}}\mathrm{max}}^{\mathrm{q}}}} \le \sqrt[\mathrm{q}]{\frac{2-{\mathrm{g}}_{{\hat{\mathrm{d}}}_{\mathrm{ij}}}^{\mathrm{q}}}{{\mathrm{g}}_{{\hat{\mathrm{d}}}_{\mathrm{ij}}}^{\mathrm{q}}}}\le \sqrt[\mathrm{q}]{\frac{2-{\mathrm{g}}_{{\hat{\mathrm{d}}}_{\mathrm{ij}}\mathrm{min}}^{\mathrm{q}}}{{\mathrm{g}}_{{\hat{\mathrm{d}}}_{\mathrm{ij}}\mathrm{min}}^{\mathrm{q}}}}$$

We have,

$$\begin{aligned} & \sqrt[\mathrm{q}]{{\prod }_{\mathrm{j}=1}^{\mathrm{m}}{\left({\prod }_{\mathrm{i}=1}^{\mathrm{n}}{\left(\frac{2-{\mathrm{g}}_{{\hat{\mathrm{d}}}_{\mathrm{ij}}\mathrm{max}}^{\mathrm{q}}}{{\mathrm{g}}_{{\hat{\mathrm{d}}}_{\mathrm{ij}}\mathrm{max}}^{\mathrm{q}}}\right)}^{{\uptheta }_{\mathrm{i}}}\right)}^{{\upomega }_{\mathrm{j}}}}\le \sqrt[\mathrm{q}]{{\prod }_{\mathrm{j}=1}^{\mathrm{m}}{\left({\prod }_{\mathrm{i}=1}^{\mathrm{n}}{\left(\frac{2-{\mathrm{g}}_{{\hat{\mathrm{d}}}_{\mathrm{ij}}}^{\mathrm{q}}}{{\mathrm{g}}_{{\hat{\mathrm{d}}}_{\mathrm{ij}}}^{\mathrm{q}}}\right)}^{{\uptheta }_{\mathrm{i}}}\right)}^{{\upomega }_{\mathrm{j}}}}\\ &\quad\le \sqrt[\mathrm{q}]{{\prod }_{\mathrm{j}=1}^{\mathrm{m}}{\left({\prod }_{\mathrm{i}=1}^{\mathrm{n}}{\left(\frac{2-{\mathrm{g}}_{{\hat{\mathrm{d}}}_{\mathrm{ij}}\mathrm{min}}^{\mathrm{q}}}{{\mathrm{g}}_{{\hat{\mathrm{d}}}_{\mathrm{ij}}\mathrm{min}}^{\mathrm{q}}}\right)}^{{\uptheta }_{\mathrm{i}}}\right)}^{{\upomega }_{\mathrm{j}}}}\end{aligned}$$

$$\begin{aligned} & \sqrt[\mathrm{q}]{{\left({\left(\frac{2-{\mathrm{g}}_{{\hat{\mathrm{d}}}_{\mathrm{ij}}\mathrm{max}}^{\mathrm{q}}}{{\mathrm{g}}_{{\hat{\mathrm{d}}}_{\mathrm{ij}}\mathrm{max}}^{\mathrm{q}}}\right)}^{\sum_{\mathrm{i}=1}^{\mathrm{n}}{\uptheta }_{\mathrm{i}}}\right)}^{{\sum }_{\mathrm{j}=1}^{\mathrm{m}}{\upomega }_{\mathrm{j}}}}\le \sqrt[\mathrm{q}]{{\prod }_{\mathrm{j}=1}^{\mathrm{m}}{\left({\prod }_{\mathrm{i}=1}^{\mathrm{n}}{\left(\frac{2-{\mathrm{g}}_{{\hat{\mathrm{d}}}_{\mathrm{ij}}}^{\mathrm{q}}}{{\mathrm{g}}_{{\hat{\mathrm{d}}}_{\mathrm{ij}}}^{\mathrm{q}}}\right)}^{{\uptheta }_{\mathrm{i}}}\right)}^{{\upomega }_{\mathrm{j}}}}\\ &\quad\le \sqrt[\mathrm{q}]{{\left({\left(\frac{2-{\mathrm{g}}_{{\hat{\mathrm{d}}}_{\mathrm{ij}}\mathrm{min}}^{\mathrm{q}}}{{\mathrm{g}}_{{\hat{\mathrm{d}}}_{\mathrm{ij}}\mathrm{min}}^{\mathrm{q}}}\right)}^{\sum_{\mathrm{i}=1}^{\mathrm{n}}{\uptheta }_{\mathrm{i}}}\right)}^{{\sum }_{\mathrm{j}=1}^{\mathrm{m}}{\upomega }_{\mathrm{j}}}}\end{aligned}$$

$$\sqrt[\mathrm{q}]{1+\left(\frac{2-{\mathrm{g}}_{{\hat{\mathrm{d}}}_{\mathrm{ij}}\mathrm{max}}^{\mathrm{q}}}{{\mathrm{g}}_{{\hat{\mathrm{d}}}_{\mathrm{ij}}\mathrm{max}}^{\mathrm{q}}}\right)}\le \sqrt[\mathrm{q}]{1+{\prod }_{\mathrm{j}=1}^{\mathrm{m}}{\left({\prod }_{\mathrm{i}=1}^{\mathrm{n}}{\left(\frac{2-{\mathrm{g}}_{{\hat{\mathrm{d}}}_{\mathrm{ij}}}^{\mathrm{q}}}{{\mathrm{g}}_{{\hat{\mathrm{d}}}_{\mathrm{ij}}}^{\mathrm{q}}}\right)}^{{\uptheta }_{\mathrm{i}}}\right)}^{{\upomega }_{\mathrm{j}}} }\le \sqrt[\mathrm{q}]{1+\left(\frac{2-{\mathrm{g}}_{{\hat{\mathrm{d}}}_{\mathrm{ij}}\mathrm{min}}^{\mathrm{q}}}{{\mathrm{g}}_{{\hat{\mathrm{d}}}_{\mathrm{ij}}\mathrm{min}}^{\mathrm{q}}}\right)}$$

$$\sqrt[\mathrm{q}]{\frac{2}{{\mathrm{g}}_{{\hat{\mathrm{d}}}_{\mathrm{ij}}\mathrm{max}}^{\mathrm{q}}}}\le \sqrt[\mathrm{q}]{1+{\prod }_{\mathrm{j}=1}^{\mathrm{m}}{\left({\prod }_{\mathrm{i}=1}^{\mathrm{n}}{\left(\frac{2-{\mathrm{g}}_{{\hat{\mathrm{d}}}_{\mathrm{ij}}}^{\mathrm{q}}}{{\mathrm{g}}_{{\hat{\mathrm{d}}}_{\mathrm{ij}}}^{\mathrm{q}}}\right)}^{{\uptheta }_{\mathrm{i}}}\right)}^{{\upomega }_{\mathrm{j}}}}\le \sqrt[\mathrm{q}]{\begin{array}{c}\frac{2}{{\mathrm{g}}_{{\hat{\mathrm{d}}}_{\mathrm{ij}}\mathrm{min}}^{\mathrm{q}}}\end{array}}$$

$$\sqrt[\mathrm{q}]{\frac{{\mathrm{g}}_{{\hat{\mathrm{d}}}_{\mathrm{ij}}\mathrm{min}}^{\mathrm{q}}}{2}}\le \sqrt[\mathrm{q}]{\frac{1}{1+{\prod }_{\mathrm{j}=1}^{\mathrm{m}}{\left({\prod }_{\mathrm{i}=1}^{\mathrm{n}}{\left(\frac{2-{\mathrm{g}}_{{\hat{\mathrm{d}}}_{\mathrm{ij}}}^{\mathrm{q}}}{{\mathrm{g}}_{{\hat{\mathrm{d}}}_{\mathrm{ij}}}^{\mathrm{q}}}\right)}^{{\uptheta }_{\mathrm{i}}}\right)}^{{\upomega }_{\mathrm{j}}}} }\le \sqrt[\mathrm{q}]{\frac{{\mathrm{g}}_{{\hat{\mathrm{d}}}_{\mathrm{ij}}\mathrm{max}}^{\mathrm{q}}}{2}}$$

$$\sqrt[\mathrm{q}]{{\mathrm{g}}_{{\hat{\mathrm{d}}}_{\mathrm{ij}}\mathrm{min}}^{\mathrm{q}}}\le \sqrt[\mathrm{q}]{\frac{2}{1+{\prod }_{\mathrm{j}=1}^{\mathrm{m}}{\left({\prod }_{\mathrm{i}=1}^{\mathrm{n}}{\left(\frac{2-{\mathrm{g}}_{{\hat{\mathrm{d}}}_{\mathrm{ij}}}^{\mathrm{q}}}{{\mathrm{g}}_{{\hat{\mathrm{d}}}_{\mathrm{ij}}}^{\mathrm{q}}}\right)}^{{\uptheta }_{\mathrm{i}}}\right)}^{{\upomega }_{\mathrm{j}}}}}\le \sqrt[\mathrm{q}]{{\mathrm{g}}_{{\hat{\mathrm{d}}}_{\mathrm{ij}}\mathrm{max}}^{\mathrm{q}}}$$

14 $${\mathrm{g}}_{{\hat{\mathrm{d}}}_{\mathrm{ij}}\mathrm{min}}\le \sqrt[\mathrm{q}]{\frac{{\prod }_{\mathrm{j}=1}^{\mathrm{m}}{\left({\prod }_{\mathrm{i}=1}^{\mathrm{n}}{\left(2{\mathrm{g}}_{{\hat{\mathrm{d}}}_{\mathrm{ij}}}^{\mathrm{q}}\right)}^{{\uptheta }_{\mathrm{i}}}\right)}^{{\upomega }_{\mathrm{j}}}}{{\prod }_{\mathrm{j}=1}^{\mathrm{m}}{\left({\prod }_{\mathrm{i}=1}^{\mathrm{n}}{\left(2-{\mathrm{g}}_{{\hat{\mathrm{d}}}_{\mathrm{ij}}}^{\mathrm{q}}\right)}^{{\uptheta }_{\mathrm{i}}}\right)}^{{\upomega }_{\mathrm{j}}}+{\prod }_{\mathrm{j}=1}^{\mathrm{m}}{\left({\prod }_{\mathrm{i}=1}^{\mathrm{n}}{\left({\mathrm{g}}_{{\hat{\mathrm{d}}}_{\mathrm{ij}}}^{\mathrm{q}}\right)}^{{\uptheta }_{\mathrm{i}}}\right)}^{{\upomega }_{\mathrm{j}}}}}\le {\mathrm{g}}_{{\hat{\mathrm{d}}}_{\mathrm{ij}}\mathrm{max}}$$

Let $\mathrm{q}-\mathrm{ROFHSEWA}\left({\mathrm{J}}_{{\hat{\mathrm{d}}}_{11}}, {\mathrm{J}}_{{\hat{\mathrm{d}}}_{12}}, \dots ,{\mathrm{J}}_{{\hat{\mathrm{d}}}_{\mathrm{nm}}}\right)={\mathrm{J}}_{{\hat{\mathrm{d}}}_{\mathrm{k}}}$ Then the inequalities can be written as ${\mathrm{g}}_{{\hat{\mathrm{d}}}_{\mathrm{ij}}\mathrm{min}}^{\mathrm{q}}\le {\mathrm{f}}_{{\hat{\mathrm{d}}}_{\mathrm{ij}}}\le {\mathrm{g}}_{{\hat{\mathrm{d}}}_{\mathrm{ij}}\mathrm{max}}^{\mathrm{q}}$ and ${\mathrm{g}}_{{\hat{\mathrm{d}}}_{\mathrm{ij}}\mathrm{min}}^{\mathrm{q}}\le {\mathrm{g}}_{{\hat{\mathrm{d}}}_{\mathrm{ij}}}\le {\mathrm{g}}_{{\hat{\mathrm{d}}}_{\mathrm{ij}}\mathrm{max}}^{\mathrm{q}}.$ Thus

$$\begin{aligned}\mathrm{S}\left({\mathrm{J}}_{{\hat{\mathrm{d}}}_{\mathrm{k}}}\right)&={\mathrm{f}}_{{\hat{\mathrm{d}}}_{\mathrm{k}}}^{\mathrm{q}}-{\mathrm{g}}_{{\hat{\mathrm{d}}}_{\mathrm{k}}}^{\mathrm{q}}+\left(\frac{{\mathrm{e}}^{{\mathrm{f}}_{{\hat{\mathrm{d}}}_{\mathrm{k}}}^{\mathrm{q}}-{\mathrm{g}}_{{\hat{\mathrm{d}}}_{\mathrm{k}}}^{\mathrm{q}}}}{{\mathrm{e}}^{{\mathrm{f}}_{{\hat{\mathrm{d}}}_{\mathrm{k}}}^{\mathrm{q}}-{\mathrm{g}}_{{\hat{\mathrm{d}}}_{\mathrm{k}}}^{\mathrm{q}}}+1}-\frac{1}{2}\right){\mathrm{\beth }}_{{\mathrm{J}}_{{\hat{\mathrm{d}}}_{\mathrm{k}}}}^{\mathrm{q}}\le {\left(\genfrac{}{}{0pt}{}{\mathrm{max}}{\mathrm{j}}\genfrac{}{}{0pt}{}{\mathrm{max}}{\mathrm{i}}\left\{{\mathrm{f}}_{{\hat{\mathrm{d}}}_{\mathrm{ij}}}\right\}\right)}^{\mathrm{q}}-{\left(\genfrac{}{}{0pt}{}{\mathrm{min}}{\mathrm{j}}\genfrac{}{}{0pt}{}{\mathrm{min}}{\mathrm{i}}\left\{{\mathrm{g}}_{{\hat{\mathrm{d}}}_{\mathrm{ij}}}\right\}\right)}^{\mathrm{q}}\\ &\quad+\left(\frac{{\mathrm{e}}^{{\left(\genfrac{}{}{0pt}{}{\mathrm{max}}{\mathrm{j}}\genfrac{}{}{0pt}{}{\mathrm{max}}{\mathrm{i}}\left\{{\mathrm{f}}_{{\hat{\mathrm{d}}}_{\mathrm{ij}}}\right\}\right)}^{\mathrm{q}}-{\left(\genfrac{}{}{0pt}{}{\mathrm{min}}{\mathrm{j}}\genfrac{}{}{0pt}{}{\mathrm{min}}{\mathrm{i}}\left\{{\mathrm{g}}_{{\hat{\mathrm{d}}}_{\mathrm{ij}}}\right\}\right)}^{\mathrm{q}}}}{{\mathrm{e}}^{{\left(\genfrac{}{}{0pt}{}{\mathrm{max}}{\mathrm{j}}\genfrac{}{}{0pt}{}{\mathrm{max}}{\mathrm{i}}\left\{{\mathrm{f}}_{{\hat{\mathrm{d}}}_{\mathrm{ij}}}\right\}\right)}^{\mathrm{q}}-{\left(\genfrac{}{}{0pt}{}{\mathrm{min}}{\mathrm{j}}\genfrac{}{}{0pt}{}{\mathrm{min}}{\mathrm{i}}\left\{{\mathrm{g}}_{{\hat{\mathrm{d}}}_{\mathrm{ij}}}\right\}\right)}^{\mathrm{q}}}+1}- \frac{1}{2}\right){{\mathrm{\beth }}_{{{\mathrm{J}}_{{\hat{\mathrm{d}}}_{\mathrm{ij}}}}^{+}}}^{\mathrm{q}}=\mathrm{S}\left({\mathrm{J}}_{{\hat{\mathrm{d}}}_{\mathrm{ij}}\mathrm{max}}\right)\end{aligned}$$

$$\Rightarrow \mathrm{S}\left({\mathrm{J}}_{{\hat{\mathrm{d}}}_{\mathrm{k}}}\right)\le \mathrm{ S}\left({\mathrm{J}}_{{\hat{\mathrm{d}}}_{\mathrm{ij}}\mathrm{max}}\right)$$ and

$$\begin{aligned}\mathrm{S}\left({\mathrm{J}}_{{\hat{\mathrm{d}}}_{\mathrm{k}}}\right)&={\mathrm{f}}_{{\hat{\mathrm{d}}}_{\mathrm{k}}}^{\mathrm{q}}-{\mathrm{g}}_{{\hat{\mathrm{d}}}_{\mathrm{k}}}^{\mathrm{q}}+\left(\frac{{\mathrm{e}}^{{\mathrm{f}}_{{\hat{\mathrm{d}}}_{\mathrm{k}}}^{\mathrm{q}}-{\mathrm{g}}_{{\hat{\mathrm{d}}}_{\mathrm{k}}}^{\mathrm{q}}}}{{\mathrm{e}}^{{\mathrm{f}}_{{\hat{\mathrm{d}}}_{\mathrm{k}}}^{\mathrm{q}}-{\mathrm{g}}_{{\hat{\mathrm{d}}}_{\mathrm{k}}}^{\mathrm{q}}}+1}-\frac{1}{2}\right){\mathrm{\beth }}_{{\mathrm{J}}_{{\hat{\mathrm{d}}}_{\mathrm{k}}}}^{\mathrm{q}}\ge {\left(\genfrac{}{}{0pt}{}{\mathrm{min}}{\mathrm{j}}\genfrac{}{}{0pt}{}{\mathrm{min}}{\mathrm{i}}\left\{{\mathrm{f}}_{{\hat{\mathrm{d}}}_{\mathrm{ij}}}\right\}\right)}^{\mathrm{q}}-{\left(\genfrac{}{}{0pt}{}{\mathrm{max}}{\mathrm{j}}\genfrac{}{}{0pt}{}{\mathrm{max}}{\mathrm{i}}\left\{{\mathrm{g}}_{{\hat{\mathrm{d}}}_{\mathrm{ij}}}\right\}\right)}^{\mathrm{q}}\\ &\quad+\left(\frac{{\mathrm{e}}^{{\left(\genfrac{}{}{0pt}{}{\mathrm{min}}{\mathrm{j}}\genfrac{}{}{0pt}{}{\mathrm{min}}{\mathrm{i}}\left\{{\mathrm{f}}_{{\hat{\mathrm{d}}}_{\mathrm{ij}}}\right\}\right)}^{\mathrm{q}}-{\left(\genfrac{}{}{0pt}{}{\mathrm{max}}{\mathrm{j}}\genfrac{}{}{0pt}{}{\mathrm{max}}{\mathrm{i}}\left\{{\mathrm{g}}_{{\hat{\mathrm{d}}}_{\mathrm{ij}}}\right\}\right)}^{\mathrm{q}}}}{{\mathrm{e}}^{{\left(\genfrac{}{}{0pt}{}{\mathrm{min}}{\mathrm{j}}\genfrac{}{}{0pt}{}{\mathrm{min}}{\mathrm{i}}\left\{{\mathrm{f}}_{{\hat{\mathrm{d}}}_{\mathrm{ij}}}\right\}\right)}^{\mathrm{q}}-{\left(\genfrac{}{}{0pt}{}{\mathrm{max}}{\mathrm{j}}\genfrac{}{}{0pt}{}{\mathrm{max}}{\mathrm{i}}\left\{{\mathrm{g}}_{{\hat{\mathrm{d}}}_{\mathrm{ij}}}\right\}\right)}^{\mathrm{q}}}+1}- \frac{1}{2}\right){{\mathrm{\beth }}_{{{\mathrm{J}}_{{\hat{\mathrm{d}}}_{\mathrm{ij}}}}^{-}}}^{\mathrm{q}}=\mathrm{S}\left({\mathrm{J}}_{{\hat{\mathrm{d}}}_{\mathrm{ij}}\mathrm{min}}\right)\end{aligned}$$

$$\Rightarrow \mathrm{S}\left({\mathrm{J}}_{{\hat{\mathrm{d}}}_{\mathrm{k}}}\right)\ge \mathrm{S}\left({\mathrm{J}}_{{\hat{\mathrm{d}}}_{\mathrm{ij}}\mathrm{min}}\right).$$

If $$\mathrm{S}\left( {\mathrm{J}}_{{\hat{\mathrm{d}}}_{\mathrm{k}}}\right)<\mathrm{ S}\left({\mathrm{J}}_{{\hat{\mathrm{d}}}_{\mathrm{ij}}\mathrm{max}}\right)\mathrm{ and S}\left( {\mathrm{J}}_{{\hat{\mathrm{d}}}_{\mathrm{k}}}\right)>\mathrm{ S}\left( {\mathrm{J}}_{{\hat{\mathrm{d}}}_{\mathrm{ij}}\mathrm{min}}\right)$$, then

15 $$\left({\mathrm{J}}_{{\hat{\mathrm{d}}}_{\mathrm{ij}}\mathrm{min}}\right)<\mathrm{q}-\mathrm{ROFHSEWA}\left({\mathrm{J}}_{{\hat{\mathrm{d}}}_{11}}, {\mathrm{J}}_{{\hat{\mathrm{d}}}_{12}}, \dots ,{\mathrm{J}}_{{\hat{\mathrm{d}}}_{\mathrm{nm}}}\right)<\left( {\mathrm{J}}_{{\hat{\mathrm{d}}}_{\mathrm{ij}}\mathrm{max}}\right)$$

If $$\mathrm{S}\left({\mathrm{J}}_{{\hat{\mathrm{d}}}_{\mathrm{k}}}\right)=\mathrm{S}\left({\mathrm{J}}_{{\hat{\mathrm{d}}}_{\mathrm{ij}}\mathrm{max}}\right),$$ then

$$\begin{aligned}& {\mathrm{f}}_{{\hat{\mathrm{d}}}_{\mathrm{k}}}^{\mathrm{q}}-{\mathrm{g}}_{{\hat{\mathrm{d}}}_{\mathrm{k}}}^{\mathrm{q}}+\left(\frac{{\mathrm{e}}^{{\mathrm{f}}_{{\hat{\mathrm{d}}}_{\mathrm{k}}}^{\mathrm{q}}-{\mathrm{g}}_{{\hat{\mathrm{d}}}_{\mathrm{k}}}^{\mathrm{q}}}}{{\mathrm{e}}^{{\mathrm{f}}_{{\hat{\mathrm{d}}}_{\mathrm{k}}}^{\mathrm{q}}-{\mathrm{g}}_{{\hat{\mathrm{d}}}_{\mathrm{k}}}^{\mathrm{q}}}+1}-\frac{1}{2}\right){\mathrm{\beth }}_{{\mathrm{J}}_{{\hat{\mathrm{d}}}_{\mathrm{k}}}}^{\mathrm{q}}\le {\left(\genfrac{}{}{0pt}{}{\mathrm{max}}{\mathrm{j}}\genfrac{}{}{0pt}{}{\mathrm{max}}{\mathrm{i}}\left\{{\mathrm{f}}_{{\hat{\mathrm{d}}}_{\mathrm{ij}}}\right\}\right)}^{\mathrm{q}}-{\left(\genfrac{}{}{0pt}{}{\mathrm{min}}{\mathrm{j}}\genfrac{}{}{0pt}{}{\mathrm{min}}{\mathrm{i}}\left\{{\mathrm{g}}_{{\hat{\mathrm{d}}}_{\mathrm{ij}}}\right\}\right)}^{\mathrm{q}}\\ &\quad+\left(\frac{{\mathrm{e}}^{{\left(\genfrac{}{}{0pt}{}{\mathrm{max}}{\mathrm{j}}\genfrac{}{}{0pt}{}{\mathrm{max}}{\mathrm{i}}\left\{{\mathrm{f}}_{{\hat{\mathrm{d}}}_{\mathrm{ij}}}\right\}\right)}^{\mathrm{q}}-{\left(\genfrac{}{}{0pt}{}{\mathrm{min}}{\mathrm{j}}\genfrac{}{}{0pt}{}{\mathrm{min}}{\mathrm{i}}\left\{{\mathrm{g}}_{{\hat{\mathrm{d}}}_{\mathrm{ij}}}\right\}\right)}^{\mathrm{q}}}}{{\mathrm{e}}^{{\left(\genfrac{}{}{0pt}{}{\mathrm{max}}{\mathrm{j}}\genfrac{}{}{0pt}{}{\mathrm{max}}{\mathrm{i}}\left\{{\mathrm{f}}_{{\hat{\mathrm{d}}}_{\mathrm{ij}}}\right\}\right)}^{\mathrm{q}}-{\left(\genfrac{}{}{0pt}{}{\mathrm{min}}{\mathrm{j}}\genfrac{}{}{0pt}{}{\mathrm{min}}{\mathrm{i}}\left\{{\mathrm{g}}_{{\hat{\mathrm{d}}}_{\mathrm{ij}}}\right\}\right)}^{\mathrm{q}}}+1}- \frac{1}{2}\right){{\mathrm{\beth }}_{{{\mathrm{J}}_{{\hat{\mathrm{d}}}_{\mathrm{ij}}}}^{+}}}^{\mathrm{q}},\text{ using the above inequalities}\end{aligned}$$

$${\mathrm{f}}_{{\hat{\mathrm{d}}}_{\mathrm{k}}}= \genfrac{}{}{0pt}{}{\mathrm{max}}{\mathrm{j}}\genfrac{}{}{0pt}{}{\mathrm{max}}{\mathrm{i}}\left\{{\mathrm{f}}_{{\hat{\mathrm{d}}}_{\mathrm{ij}}}\right\}$$, and $${\mathrm{g}}_{{\hat{\mathrm{d}}}_{\mathrm{k}}}= \genfrac{}{}{0pt}{}{\mathrm{min}}{\mathrm{j}}\genfrac{}{}{0pt}{}{\mathrm{min}}{\mathrm{i}}\left\{{\mathrm{g}}_{{\hat{\mathrm{d}}}_{\mathrm{ij}}}\right\}.\mathrm{Hence},{{\mathrm{\beth }}_{{\mathrm{J}}_{{\hat{\mathrm{d}}}_{\mathrm{k}}}}}^{\mathrm{q}}={{\mathrm{\beth }}_{{{\mathrm{J}}_{{\hat{\mathrm{d}}}_{\mathrm{ij}}}}^{+}}}^{\mathrm{q}}.\mathrm{ Then}$$

$$\mathrm{q}-\mathrm{ROFHSEWA}\left({\mathrm{J}}_{{\hat{\mathrm{d}}}_{11}}, {\mathrm{J}}_{{\hat{\mathrm{d}}}_{12}}, \dots ,{\mathrm{J}}_{{\hat{\mathrm{d}}}_{\mathrm{nm}}}\right)={\mathrm{J}}_{{\hat{\mathrm{d}}}_{\mathrm{ij}}\mathrm{max}}.$$

If $$\mathrm{S}\left({\mathrm{J}}_{{\hat{\mathrm{d}}}_{\mathrm{k}}}\right)=\mathrm{S}\left({\mathrm{J}}_{{\hat{\mathrm{d}}}_{\mathrm{ij}}\mathrm{min}}\right),\mathrm{ then}$$$${\mathrm{f}}_{{\hat{\mathrm{d}}}_{\mathrm{k}}}^{\mathrm{q}}-{\mathrm{g}}_{{\hat{\mathrm{d}}}_{\mathrm{k}}}^{\mathrm{q}}+\left(\frac{{\mathrm{e}}^{{\mathrm{f}}_{{\hat{\mathrm{d}}}_{\mathrm{k}}}^{\mathrm{q}}-{\mathrm{g}}_{{\hat{\mathrm{d}}}_{\mathrm{k}}}^{\mathrm{q}}}}{{\mathrm{e}}^{{\mathrm{f}}_{{\hat{\mathrm{d}}}_{\mathrm{k}}}^{\mathrm{q}}-{\mathrm{g}}_{{\hat{\mathrm{d}}}_{\mathrm{k}}}^{\mathrm{q}}}+1}-\frac{1}{2}\right){\mathrm{\beth }}_{{\mathrm{J}}_{{\hat{\mathrm{d}}}_{\mathrm{k}}}}^{\mathrm{q}}\le {\left(\genfrac{}{}{0pt}{}{\mathrm{min}}{\mathrm{j}}\genfrac{}{}{0pt}{}{\mathrm{min}}{\mathrm{i}}\left\{{\mathrm{f}}_{{\hat{\mathrm{d}}}_{\mathrm{ij}}}\right\}\right)}^{\mathrm{q}}-{\left(\genfrac{}{}{0pt}{}{\mathrm{max}}{\mathrm{j}}\genfrac{}{}{0pt}{}{\mathrm{max}}{\mathrm{i}}\left\{{\mathrm{g}}_{{\hat{\mathrm{d}}}_{\mathrm{ij}}}\right\}\right)}^{\mathrm{q}}+\left(\frac{{\mathrm{e}}^{{\left(\genfrac{}{}{0pt}{}{\mathrm{min}}{\mathrm{j}}\genfrac{}{}{0pt}{}{\mathrm{min}}{\mathrm{i}}\left\{{\mathrm{f}}_{{\hat{\mathrm{d}}}_{\mathrm{ij}}}\right\}\right)}^{\mathrm{q}}-{\left(\genfrac{}{}{0pt}{}{\mathrm{max}}{\mathrm{j}}\genfrac{}{}{0pt}{}{\mathrm{max}}{\mathrm{i}}\left\{{\mathrm{g}}_{{\hat{\mathrm{d}}}_{\mathrm{ij}}}\right\}\right)}^{\mathrm{q}}}}{{\mathrm{e}}^{{\left(\genfrac{}{}{0pt}{}{\mathrm{min}}{\mathrm{j}}\genfrac{}{}{0pt}{}{\mathrm{min}}{\mathrm{i}}\left\{{\mathrm{f}}_{{\hat{\mathrm{d}}}_{\mathrm{ij}}}\right\}\right)}^{\mathrm{q}}-{\left(\genfrac{}{}{0pt}{}{\mathrm{max}}{\mathrm{j}}\genfrac{}{}{0pt}{}{\mathrm{max}}{\mathrm{i}}\left\{{\mathrm{g}}_{{\hat{\mathrm{d}}}_{\mathrm{ij}}}\right\}\right)}^{\mathrm{q}}}+1}- \frac{1}{2}\right){{\mathrm{\beth }}_{{{\mathrm{J}}_{{\hat{\mathrm{d}}}_{\mathrm{ij}}}}^{-}}}^{\mathrm{q}}$$, using the above inequalities$${\mathrm{f}}_{{\hat{\mathrm{d}}}_{\mathrm{k}}}= \genfrac{}{}{0pt}{}{\mathrm{min}}{\mathrm{j}}\genfrac{}{}{0pt}{}{\mathrm{min}}{\mathrm{i}}\left\{{\mathrm{f}}_{{\hat{\mathrm{d}}}_{\mathrm{ij}}}\right\}$$, and $${\mathrm{g}}_{{\hat{\mathrm{d}}}_{\mathrm{k}}}= \genfrac{}{}{0pt}{}{\mathrm{max}}{\mathrm{j}}\genfrac{}{}{0pt}{}{\mathrm{max}}{\mathrm{i}}\left\{{\mathrm{g}}_{{\hat{\mathrm{d}}}_{\mathrm{ij}}}\right\}$$. Hence, $${{\mathrm{\beth }}_{{\mathrm{J}}_{{\hat{\mathrm{d}}}_{\mathrm{k}}}}}^{\mathrm{q}}={{\mathrm{\beth }}_{{{\mathrm{J}}_{{\hat{\mathrm{d}}}_{\mathrm{ij}}}}^{-}}}^{\mathrm{q}}$$. Then$$\mathrm{q}-\mathrm{ROFHSEWA }\left({\mathrm{J}}_{{\hat{\mathrm{d}}}_{11}}, {\mathrm{J}}_{{\hat{\mathrm{d}}}_{12}}, \dots ,{\mathrm{J}}_{{\hat{\mathrm{d}}}_{\mathrm{nm}}}\right)$$ = $${\mathrm{J}}_{{\hat{\mathrm{d}}}_{\mathrm{ij}}\mathrm{min}}.$$

So, it is proven that

$${\mathrm{J}}_{{\hat{\mathrm{d}}}_{\mathrm{ij}}\mathrm{min}}\le \mathrm{q}-\mathrm{ROFHSEWA}\left({\mathrm{J}}_{{\hat{\mathrm{d}}}_{11}}, {\mathrm{J}}_{{\hat{\mathrm{d}}}_{12}}, \dots ,{\mathrm{J}}_{{\hat{\mathrm{d}}}_{\mathrm{nm}}}\right)\le {\mathrm{J}}_{{\hat{\mathrm{d}}}_{\mathrm{ij}}\mathrm{max}}.$$

#### Homogeneity

Prove that $\mathrm{q}-\mathrm{ROFHSEWA}\left({\mathrm{J}}_{{\hat{\mathrm{d}}}_{11}}, {\mathrm{J}}_{{\hat{\mathrm{d}}}_{12}}, \dots ,{\mathrm{J}}_{{\hat{\mathrm{d}}}_{\mathrm{nm}}}\right)=\mathrm{ \gamma q}-\mathrm{ROFHSEWA}\left({\mathrm{J}}_{{\hat{\mathrm{d}}}_{11}}, {\mathrm{J}}_{{\hat{\mathrm{d}}}_{12}}, \dots ,{\mathrm{J}}_{{\hat{\mathrm{d}}}_{\mathrm{nm}}}\right)$ for $\upgamma >0.$

##### Proof

Let ${\mathrm{J}}_{{\hat{\mathrm{d}}}_{\mathrm{ij}}}$ be a collection of $\upgamma$ is a positive number, then

$$\upgamma {\mathrm{J}}_{{\hat{\mathrm{d}}}_{\mathrm{ij}} }=\Bigg\langle \sqrt[\mathrm{q}]{\frac{{\left(1+{\mathrm{f}}_{{\hat{\mathrm{d}}}_{\mathrm{ij}}}^{\mathrm{q}}\right)}^{\upgamma }-{\left(1-{\mathrm{f}}_{{\hat{\mathrm{d}}}_{\mathrm{ij}}}^{\mathrm{q}}\right)}^{\upgamma }}{{\left(1+{\mathrm{f}}_{{\hat{\mathrm{d}}}_{\mathrm{ij}}}^{\mathrm{q}}\right)}^{\upgamma }+{\left(1-{\mathrm{f}}_{{\hat{\mathrm{d}}}_{\mathrm{ij}}}^{\mathrm{q}}\right)}^{\upgamma }}}, \sqrt[\mathrm{q}]{\frac{2{\left({\mathrm{g}}_{{\hat{\mathrm{d}}}_{\mathrm{ij}}}^{\mathrm{q}}\right)}^{\upgamma }}{{\left(2-{\mathrm{g}}_{{\hat{\mathrm{d}}}_{\mathrm{ij}}}^{\mathrm{q}}\right)}^{\upgamma }+{\left({\mathrm{g}}_{{\hat{\mathrm{d}}}_{\mathrm{ij}}}^{\mathrm{q}}\right)}^{\upgamma }}}\Bigg\rangle$$

So we have,

$$\begin{aligned}\mathrm{q}-\mathrm{ROFHSEWA}\left({\mathrm{J}}_{{\hat{\mathrm{d}}}_{11}}, {\mathrm{J}}_{{\hat{\mathrm{d}}}_{12}}, \dots ,{\mathrm{J}}_{{\hat{\mathrm{d}}}_{\mathrm{nm}}}\right)&=\Bigg\langle \sqrt[\mathrm{q}]{\frac{{\prod }_{\mathrm{j}=1}^{\mathrm{m}}{\left({\prod }_{\mathrm{i}=1}^{\mathrm{n}}{\left(1+{\mathrm{f}}_{{\hat{\mathrm{d}}}_{\mathrm{ij}}}^{\mathrm{q}}\right)}^{{\uptheta }_{\mathrm{i}}}\right)}^{{\upomega }_{\mathrm{j}}}-{\prod }_{\mathrm{j}=1}^{\mathrm{m}}{\left({\prod }_{\mathrm{i}=1}^{\mathrm{n}}{\left(1-{\mathrm{f}}_{{\hat{\mathrm{d}}}_{\mathrm{ij}}}^{\mathrm{q}}\right)}^{{\uptheta }_{\mathrm{i}}}\right)}^{{\upomega }_{\mathrm{j}}}}{{\prod }_{\mathrm{j}=1}^{\mathrm{m}}{\left({\prod }_{\mathrm{i}=1}^{\mathrm{n}}{\left(1+{\mathrm{f}}_{{\hat{\mathrm{d}}}_{\mathrm{ij}}}^{\mathrm{q}}\right)}^{{\uptheta }_{\mathrm{i}}}\right)}^{{\upomega }_{\mathrm{j}}}+{\prod }_{\mathrm{j}=1}^{\mathrm{m}}{\left({\prod }_{\mathrm{i}=1}^{\mathrm{n}}{\left(1-{\mathrm{f}}_{{\hat{\mathrm{d}}}_{\mathrm{ij}}}^{\mathrm{q}}\right)}^{{\uptheta }_{\mathrm{i}}}\right)}^{{\upomega }_{\mathrm{j}}}}},\\ &\quad \sqrt[\mathrm{q}]{\frac{{\prod }_{\mathrm{j}=1}^{\mathrm{m}}{\left({\prod }_{\mathrm{i}=1}^{\mathrm{n}}{\left(2{\mathrm{g}}_{{\hat{\mathrm{d}}}_{\mathrm{ij}}}^{\mathrm{q}}\right)}^{{\uptheta }_{\mathrm{i}}}\right)}^{{\upomega }_{\mathrm{j}}}}{{\prod }_{\mathrm{j}=1}^{\mathrm{m}}{\left({\prod }_{\mathrm{i}=1}^{\mathrm{n}}{\left(2-{\mathrm{g}}_{{\hat{\mathrm{d}}}_{\mathrm{ij}}}^{\mathrm{q}}\right)}^{{\uptheta }_{\mathrm{i}}}\right)}^{{\upomega }_{\mathrm{j}}}+{\prod }_{\mathrm{j}=1}^{\mathrm{m}}{\left({\prod }_{\mathrm{i}=1}^{\mathrm{n}}{\left({\mathrm{g}}_{{\hat{\mathrm{d}}}_{\mathrm{ij}}}^{\mathrm{q}}\right)}^{{\uptheta }_{\mathrm{i}}}\right)}^{{\upomega }_{\mathrm{j}}}}}\Bigg\rangle \end{aligned}$$

$$=\Bigg\langle \begin{array}{c}\sqrt[\mathrm{q}]{\frac{{\left({\prod }_{\mathrm{j}=1}^{\mathrm{m}}{\left({\prod }_{\mathrm{i}=1}^{\mathrm{n}}{\left(1+{\mathrm{f}}_{{\hat{\mathrm{d}}}_{\mathrm{ij}}}^{\mathrm{q}}\right)}^{{\uptheta }_{\mathrm{i}}}\right)}^{{\upomega }_{\mathrm{j}}}\right)}^{\upgamma }-{\left({\prod }_{\mathrm{j}=1}^{\mathrm{m}}{\left({\prod }_{\mathrm{i}=1}^{\mathrm{n}}{\left(1-{\mathrm{f}}_{{\hat{\mathrm{d}}}_{\mathrm{ij}}}^{\mathrm{q}}\right)}^{{\uptheta }_{\mathrm{i}}}\right)}^{{\upomega }_{\mathrm{j}}}\right)}^{\upgamma }}{{\left({\prod }_{\mathrm{j}=1}^{\mathrm{m}}{\left({\prod }_{\mathrm{i}=1}^{\mathrm{n}}{\left(1+{\mathrm{f}}_{{\hat{\mathrm{d}}}_{\mathrm{ij}}}^{\mathrm{q}}\right)}^{{\uptheta }_{\mathrm{i}}}\right)}^{{\upomega }_{\mathrm{j}}}\right)}^{\upgamma }+{\left({\prod }_{\mathrm{j}=1}^{\mathrm{m}}{\left({\prod }_{\mathrm{i}=1}^{\mathrm{n}}{\left(1-{\mathrm{f}}_{{\hat{\mathrm{d}}}_{\mathrm{ij}}}^{\mathrm{q}}\right)}^{{\uptheta }_{\mathrm{i}}}\right)}^{{\upomega }_{\mathrm{j}}}\right)}^{\upgamma }}}, \\ \sqrt[\mathrm{q}]{\frac{{\left({\prod }_{\mathrm{j}=1}^{\mathrm{m}}{\left({\prod }_{\mathrm{i}=1}^{\mathrm{n}}{\left(2{\mathrm{g}}_{{\hat{\mathrm{d}}}_{\mathrm{ij}}}^{\mathrm{q}}\right)}^{{\uptheta }_{\mathrm{i}}}\right)}^{{\upomega }_{\mathrm{j}}}\right)}^{\upgamma }}{{\left({\prod }_{\mathrm{j}=1}^{\mathrm{m}}{\left({\prod }_{\mathrm{i}=1}^{\mathrm{n}}{\left(2-{\mathrm{g}}_{{\hat{\mathrm{d}}}_{\mathrm{ij}}}^{\mathrm{q}}\right)}^{{\uptheta }_{\mathrm{i}}}\right)}^{{\upomega }_{\mathrm{j}}}\right)}^{\upgamma }+{\left({\prod }_{\mathrm{j}=1}^{\mathrm{m}}{\left({\prod }_{\mathrm{i}=1}^{\mathrm{n}}{\left(2{\mathrm{g}}_{{\hat{\mathrm{d}}}_{\mathrm{ij}}}^{\mathrm{q}}\right)}^{{\uptheta }_{\mathrm{i}}}\right)}^{{\upomega }_{\mathrm{j}}}\right)}^{\upgamma }}}\end{array}\Bigg\rangle$$

$$=\mathrm{\gamma q}-\mathrm{ROFHSEWA}\left({\mathrm{J}}_{{\hat{\mathrm{d}}}_{11}}, {\mathrm{J}}_{{\hat{\mathrm{d}}}_{12}}, \dots ,{\mathrm{J}}_{{\hat{\mathrm{d}}}_{\mathrm{nm}}}\right).$$

#### Monotonicity

Let ${\mathrm{J}}_{{\hat{\mathrm{d}}}_{\mathrm{ij}} }= \left({\mathrm{f}}_{{\hat{\mathrm{d}}}_{\mathrm{ij}} }, {\mathrm{g}}_{{\hat{\mathrm{d}}}_{\mathrm{ij}}}\right)$ and ${\mathrm{J}}_{{\hat{\mathrm{d}}}_{\mathrm{ij}}}^{*}=\left({\mathrm{f}}_{{\hat{\mathrm{d}}}_{\mathrm{ij}}}^{*}, {\mathrm{g}}_{{\hat{\mathrm{d}}}_{\mathrm{ij}}}^{*}\right)$ be the collection of q-ROFHSNs. Then$\mathrm{q}-\mathrm{ROFHSEWA}\left({\mathrm{J}}_{{\hat{\mathrm{d}}}_{11}}, {\mathrm{J}}_{{\hat{\mathrm{d}}}_{12}}, \dots ,{\mathrm{J}}_{{\hat{\mathrm{d}}}_{\mathrm{nm}}}\right)\le \mathrm{q}-\mathrm{ROFHSEWA}\left({\mathrm{J}}_{{\hat{\mathrm{d}}}_{11}}^{*}, {\mathrm{J}}_{{\hat{\mathrm{d}}}_{12}}^{*}, \dots ,{\mathrm{J}}_{{\hat{\mathrm{d}}}_{\mathrm{nm}}}^{*}\right)$, if ${\mathrm{J}}_{{\hat{\mathrm{d}}}_{\mathrm{ij}} }\le {\mathrm{J}}_{{\hat{\mathrm{d}}}_{\mathrm{ij}}}^{*}$ $\forall \mathrm{ i},\mathrm{ j}$.

##### Proof

Let $\mathrm{h}\left(\mathrm{x}\right)=\sqrt[\mathrm{q}]{\frac{1-{\mathrm{x}}^{\mathrm{q}}}{1+{\mathrm{x}}^{\mathrm{q}}}}$, $\mathrm{x}\in \left[0, 1\right]$, then $\frac{\mathrm{d}}{\mathrm{d}\left(\mathrm{y}\right)}\mathrm{h}\left(\mathrm{x}\right)=-\frac{1}{\mathrm{q}}{\left(\frac{1-{\mathrm{x}}^{\mathrm{q}}}{1+{\mathrm{x}}^{\mathrm{q}}}\right)}^{\frac{1}{\mathrm{q}}-1}\left\{\frac{{\mathrm{qx}}^{2\mathrm{q}-1}+{\mathrm{qx}}^{2\mathrm{q}-1}}{{\left(1+{\mathrm{x}}^{\mathrm{q}}\right)}^{2}}\right\}$ < 0, so $\mathrm{h}(\mathrm{x})$ is decreasing function on $\left[0, 1\right]$. If ${\mathrm{f}}_{{\hat{\mathrm{d}}}_{\mathrm{ij}} }\le {\mathrm{f}}_{{\hat{\mathrm{d}}}_{\mathrm{ij}}}^{*}$, then $\mathrm{h}\left({\mathrm{f}}_{{\hat{\mathrm{d}}}_{\mathrm{ij}}}^{*}\right)\le \mathrm{h}\left({\mathrm{f}}_{{\hat{\mathrm{d}}}_{\mathrm{ij}}}\right)\forall \mathrm{ i},\mathrm{ j}$.

$$1-{\mathrm{f}}_{{\hat{\mathrm{d}}}_{\mathrm{ij}}}^{*}\le 1-{\mathrm{f}}_{{\hat{\mathrm{d}}}_{\mathrm{ij}}}$$

$$\Rightarrow 1-{\mathrm{f}}_{{\hat{\mathrm{d}}}_{\mathrm{ij}}}^{\mathrm{q}*}\le 1-{\mathrm{f}}_{{\hat{\mathrm{d}}}_{\mathrm{ij}}}^{\mathrm{q}}$$

$$\Rightarrow \left(1+{\mathrm{f}}_{{\hat{\mathrm{d}}}_{\mathrm{ij}}}^{\mathrm{q}}\right)-\left(1-{\mathrm{f}}_{{\hat{\mathrm{d}}}_{\mathrm{ij}}}^{\mathrm{q}}\right)\le \left(1+{\mathrm{f}}_{{\hat{\mathrm{d}}}_{\mathrm{ij}}}^{\mathrm{q}*}\right)-\left(1-{\mathrm{f}}_{{\hat{\mathrm{d}}}_{\mathrm{ij}}}^{\mathrm{q}*}\right)$$

$$\Rightarrow \frac{\left(1+{\mathrm{f}}_{{\hat{\mathrm{d}}}_{\mathrm{ij}}}^{\mathrm{q}}\right)-\left(1-{\mathrm{f}}_{{\hat{\mathrm{d}}}_{\mathrm{ij}}}^{\mathrm{q}}\right)}{\left(1+{\mathrm{f}}_{{\hat{\mathrm{d}}}_{\mathrm{ij}}}^{\mathrm{q}}\right)+\left(1-{\mathrm{f}}_{{\hat{\mathrm{d}}}_{\mathrm{ij}}}^{\mathrm{q}}\right)}\le \frac{\left(1+{\mathrm{f}}_{{\hat{\mathrm{d}}}_{\mathrm{ij}}}^{\mathrm{q}*}\right)-\left(1-{\mathrm{f}}_{{\hat{\mathrm{d}}}_{\mathrm{ij}}}^{\mathrm{q}*}\right)}{\left(1+{\mathrm{f}}_{{\hat{\mathrm{d}}}_{\mathrm{ij}}}^{\mathrm{q}*}\right)+\left(1-{\mathrm{f}}_{{\hat{\mathrm{d}}}_{\mathrm{ij}}}^{\mathrm{q}*}\right)}$$

where, ${\uptheta }_{\mathrm{i}}>0$, ${\sum }_{\mathrm{i}=1}^{\mathrm{n}}{\uptheta }_{\mathrm{i}}=1$ and ${\upomega }_{\mathrm{j}}>0,$ ${\sum }_{\mathrm{j}=1}^{\mathrm{m}}{\upomega }_{\mathrm{j}}=1$. So,

$$\Rightarrow \frac{\left(1+{\mathrm{f}}_{{\hat{\mathrm{d}}}_{\mathrm{ij}}}^{\mathrm{q}}\right)-\left(1-{\mathrm{f}}_{{\hat{\mathrm{d}}}_{\mathrm{ij}}}^{\mathrm{q}}\right)}{\left(1+{\mathrm{f}}_{{\hat{\mathrm{d}}}_{\mathrm{ij}}}^{\mathrm{q}}\right)+\left(1-{\mathrm{f}}_{{\hat{\mathrm{d}}}_{\mathrm{ij}}}^{\mathrm{q}}\right)}\le \frac{\left(1+{\mathrm{f}}_{{\hat{\mathrm{d}}}_{\mathrm{ij}}}^{\mathrm{q}*}\right)-\left(1-{\mathrm{f}}_{{\hat{\mathrm{d}}}_{\mathrm{ij}}}^{\mathrm{q}*}\right)}{\left(1+{\mathrm{f}}_{{\hat{\mathrm{d}}}_{\mathrm{ij}}}^{\mathrm{q}*}\right)+\left(1-{\mathrm{f}}_{{\hat{\mathrm{d}}}_{\mathrm{ij}}}^{\mathrm{q}*}\right)}$$

$$\begin{aligned}&\Rightarrow \frac{{\left({\left(\left(1+{\mathrm{f}}_{{\hat{\mathrm{d}}}_{\mathrm{ij}}}^{\mathrm{q}}\right)\right)}^{{\sum }_{\mathrm{i}=1}^{\mathrm{n}}{\uptheta }_{\mathrm{i}}}\right)}^{{\sum }_{\mathrm{j}=1}^{\mathrm{m}}{\upomega }_{\mathrm{j}}}-{\left({\left(\left(1-{\mathrm{f}}_{{\hat{\mathrm{d}}}_{\mathrm{ij}}}^{\mathrm{q}}\right)\right)}^{{\sum }_{\mathrm{i}=1}^{\mathrm{n}}{\uptheta }_{\mathrm{i}}}\right)}^{{\sum }_{\mathrm{j}=1}^{\mathrm{m}}{\upomega }_{\mathrm{j}}}}{{\left({\left(\left(1+{\mathrm{f}}_{{\hat{\mathrm{d}}}_{\mathrm{ij}}}^{\mathrm{q}}\right)\right)}^{{\sum }_{\mathrm{i}=1}^{\mathrm{n}}{\uptheta }_{\mathrm{i}}}\right)}^{{\sum }_{\mathrm{j}=1}^{\mathrm{m}}{\upomega }_{\mathrm{j}}}+{\left({\left(\left(1-{\mathrm{f}}_{{\hat{\mathrm{d}}}_{\mathrm{ij}}}^{\mathrm{q}}\right)\right)}^{{\sum }_{\mathrm{i}=1}^{\mathrm{n}}{\uptheta }_{\mathrm{i}}}\right)}^{{\sum }_{\mathrm{j}=1}^{\mathrm{m}}{\upomega }_{\mathrm{j}}}}\\ &\le \frac{{\left({\left(\left(1+{\mathrm{f}}_{{\hat{\mathrm{d}}}_{\mathrm{ij}}}^{\mathrm{q}*}\right)\right)}^{{\sum }_{\mathrm{i}=1}^{\mathrm{n}}{\uptheta }_{\mathrm{i}}}\right)}^{{\sum }_{\mathrm{j}=1}^{\mathrm{m}}{\upomega }_{\mathrm{j}}}-{\left({\left(\left(1-{\mathrm{f}}_{{\hat{\mathrm{d}}}_{\mathrm{ij}}}^{\mathrm{q}*}\right)\right)}^{{\sum }_{\mathrm{i}=1}^{\mathrm{n}}{\uptheta }_{\mathrm{i}}}\right)}^{{\sum }_{\mathrm{j}=1}^{\mathrm{m}}{\upomega }_{\mathrm{j}}}}{{\left({\left(\left(1+{\mathrm{f}}_{{\hat{\mathrm{d}}}_{\mathrm{ij}}}^{\mathrm{q}*}\right)\right)}^{{\sum }_{\mathrm{i}=1}^{\mathrm{n}}{\uptheta }_{\mathrm{i}}}\right)}^{{\sum }_{\mathrm{j}=1}^{\mathrm{m}}{\upomega }_{\mathrm{j}}}+{\left({\left(\left(1-{\mathrm{f}}_{{\hat{\mathrm{d}}}_{\mathrm{ij}}}^{\mathrm{q}*}\right)\right)}^{{\sum }_{\mathrm{i}=1}^{\mathrm{n}}{\uptheta }_{\mathrm{i}}}\right)}^{{\sum }_{\mathrm{j}=1}^{\mathrm{m}}{\upomega }_{\mathrm{j}}}}\end{aligned}$$

$$\begin{aligned}&\Rightarrow \frac{{\prod }_{\mathrm{j}=1}^{\mathrm{m}}{\left({\prod }_{\mathrm{i}=1}^{\mathrm{n}}{\left(1+{\mathrm{f}}_{{\hat{\mathrm{d}}}_{\mathrm{ij}}}^{\mathrm{q}}\right)}^{{\uptheta }_{\mathrm{i}}}\right)}^{{\upomega }_{\mathrm{j}}}-{\prod }_{\mathrm{j}=1}^{\mathrm{m}}{\left({\prod }_{\mathrm{i}=1}^{\mathrm{n}}{\left(1-{\mathrm{f}}_{{\hat{\mathrm{d}}}_{\mathrm{ij}}}^{\mathrm{q}}\right)}^{{\uptheta }_{\mathrm{i}}}\right)}^{{\upomega }_{\mathrm{j}}}}{{\prod }_{\mathrm{j}=1}^{\mathrm{m}}{\left({\prod }_{\mathrm{i}=1}^{\mathrm{n}}{\left(1+{\mathrm{f}}_{{\hat{\mathrm{d}}}_{\mathrm{ij}}}^{\mathrm{q}}\right)}^{{\uptheta }_{\mathrm{i}}}\right)}^{{\upomega }_{\mathrm{j}}}+{\prod }_{\mathrm{j}=1}^{\mathrm{m}}{\left({\prod }_{\mathrm{i}=1}^{\mathrm{n}}{\left(1-{\mathrm{f}}_{{\hat{\mathrm{d}}}_{\mathrm{ij}}}^{\mathrm{q}}\right)}^{{\uptheta }_{\mathrm{i}}}\right)}^{{\upomega }_{\mathrm{j}}}}\\ &\le \frac{{\prod }_{\mathrm{j}=1}^{\mathrm{m}}{\left({\prod }_{\mathrm{i}=1}^{\mathrm{n}}{\left(1+{\mathrm{f}}_{{\hat{\mathrm{d}}}_{\mathrm{ij}}}^{\mathrm{q}*}\right)}^{{\uptheta }_{\mathrm{i}}}\right)}^{{\upomega }_{\mathrm{j}}}-{\prod }_{\mathrm{j}=1}^{\mathrm{m}}{\left({\prod }_{\mathrm{i}=1}^{\mathrm{n}}{\left(1-{\mathrm{f}}_{{\hat{\mathrm{d}}}_{\mathrm{ij}}}^{\mathrm{q}*}\right)}^{{\uptheta }_{\mathrm{i}}}\right)}^{{\upomega }_{\mathrm{j}}}}{{\prod }_{\mathrm{j}=1}^{\mathrm{m}}{\left({\prod }_{\mathrm{i}=1}^{\mathrm{n}}{\left(1+{\mathrm{f}}_{{\hat{\mathrm{d}}}_{\mathrm{ij}}}^{\mathrm{q}*}\right)}^{{\uptheta }_{\mathrm{i}}}\right)}^{{\upomega }_{\mathrm{j}}}+{\prod }_{\mathrm{j}=1}^{\mathrm{m}}{\left({\prod }_{\mathrm{i}=1}^{\mathrm{n}}{\left(1-{\mathrm{f}}_{{\hat{\mathrm{d}}}_{\mathrm{ij}}}^{\mathrm{q}*}\right)}^{{\uptheta }_{\mathrm{i}}}\right)}^{{\upomega }_{\mathrm{j}}}}\end{aligned}$$

$$\begin{aligned}&\Rightarrow \frac{\sqrt[\mathrm{q}]{{\prod }_{\mathrm{j}=1}^{\mathrm{m}}{\left({\prod }_{\mathrm{i}=1}^{\mathrm{n}}{\left(1+{\mathrm{f}}_{{\hat{\mathrm{d}}}_{\mathrm{ij}}}^{\mathrm{q}}\right)}^{{\uptheta }_{\mathrm{i}}}\right)}^{{\upomega }_{\mathrm{j}}}-{\prod }_{\mathrm{j}=1}^{\mathrm{m}}{\left({\prod }_{\mathrm{i}=1}^{\mathrm{n}}{\left(1-{\mathrm{f}}_{{\hat{\mathrm{d}}}_{\mathrm{ij}}}^{\mathrm{q}}\right)}^{{\uptheta }_{\mathrm{i}}}\right)}^{{\upomega }_{\mathrm{j}}}}}{\sqrt[\mathrm{q}]{{\prod }_{\mathrm{j}=1}^{\mathrm{m}}{\left({\prod }_{\mathrm{i}=1}^{\mathrm{n}}{\left(1+{\mathrm{f}}_{{\hat{\mathrm{d}}}_{\mathrm{ij}}}^{\mathrm{q}}\right)}^{{\uptheta }_{\mathrm{i}}}\right)}^{{\upomega }_{\mathrm{j}}}+{\prod }_{\mathrm{j}=1}^{\mathrm{m}}{\left({\prod }_{\mathrm{i}=1}^{\mathrm{n}}{\left(1-{\mathrm{f}}_{{\hat{\mathrm{d}}}_{\mathrm{ij}}}^{\mathrm{q}}\right)}^{{\uptheta }_{\mathrm{i}}}\right)}^{{\upomega }_{\mathrm{j}}}}}\\ &\le \frac{\sqrt[\mathrm{q}]{{\prod }_{\mathrm{j}=1}^{\mathrm{m}}{\left({\prod }_{\mathrm{i}=1}^{\mathrm{n}}{\left(1+{\mathrm{f}}_{{\hat{\mathrm{d}}}_{\mathrm{ij}}}^{\mathrm{q}*}\right)}^{{\uptheta }_{\mathrm{i}}}\right)}^{{\upomega }_{\mathrm{j}}}-{\prod }_{\mathrm{j}=1}^{\mathrm{m}}{\left({\prod }_{\mathrm{i}=1}^{\mathrm{n}}{\left(1-{\mathrm{f}}_{{\hat{\mathrm{d}}}_{\mathrm{ij}}}^{\mathrm{q}*}\right)}^{{\uptheta }_{\mathrm{i}}}\right)}^{{\upomega }_{\mathrm{j}}}}}{\sqrt[\mathrm{q}]{{\prod }_{\mathrm{j}=1}^{\mathrm{m}}{\left({\prod }_{\mathrm{i}=1}^{\mathrm{n}}{\left(1+{\mathrm{f}}_{{\hat{\mathrm{d}}}_{\mathrm{ij}}}^{\mathrm{q}*}\right)}^{{\uptheta }_{\mathrm{i}}}\right)}^{{\upomega }_{\mathrm{j}}}+{\prod }_{\mathrm{j}=1}^{\mathrm{m}}{\left({\prod }_{\mathrm{i}=1}^{\mathrm{n}}{\left(1-{\mathrm{f}}_{{\hat{\mathrm{d}}}_{\mathrm{ij}}}^{\mathrm{q}*}\right)}^{{\uptheta }_{\mathrm{i}}}\right)}^{{\upomega }_{\mathrm{j}}}}}\end{aligned}$$

Let $\mathrm{k}\left(\mathrm{y}\right)=\sqrt[\mathrm{q}]{\frac{2-{\mathrm{y}}^{\mathrm{q}}}{{\mathrm{y}}^{\mathrm{q}}}}$, $\mathrm{y}\in \left[0, 1\right]$, then $\frac{\mathrm{d}}{\mathrm{dy}}\left(\mathrm{k}(\mathrm{y})\right)=-\frac{1}{\mathrm{q}}{\left(\frac{2-{\mathrm{y}}^{\mathrm{q}}}{{\mathrm{y}}^{\mathrm{q}}}\right)}^{\frac{1}{\mathrm{q}}-1}\left(\frac{2}{{\left({\mathrm{y}}^{\mathrm{q}}\right)}^{2}}\right)$. So, $\frac{\mathrm{d}}{\mathrm{dy}}\left(\mathrm{k}(\mathrm{y})\right)=-\frac{1}{\mathrm{q}}{\left(\frac{2-{\mathrm{y}}^{\mathrm{q}}}{{\mathrm{y}}^{\mathrm{q}}}\right)}^{\frac{1}{\mathrm{q}}-1}\left(\frac{2}{{\left({\mathrm{y}}^{\mathrm{q}}\right)}^{2}}\right)<0$. So, $\mathrm{k}(\mathrm{y})$ is decreasing on $\left[0, 1\right]$. If ${\mathrm{g}}_{{\hat{\mathrm{d}}}_{\mathrm{ij}}}^{*}\le {\mathrm{g}}_{{\hat{\mathrm{d}}}_{\mathrm{ij}}}$, then $\mathrm{k}\left({\mathrm{g}}_{{\hat{\mathrm{d}}}_{\mathrm{ij}}}^{*}\right)\ge \mathrm{k}\left({\mathrm{g}}_{{\hat{\mathrm{d}}}_{\mathrm{ij}}}\right)\forall \mathrm{ i},\mathrm{ j}$. There are two possibilities

(i) $${\mathrm{g}}_{{\hat{\mathrm{d}}}_{\mathrm{ij}}}^{*}\le {\mathrm{g}}_{{\hat{\mathrm{d}}}_{\mathrm{ij}} }\Rightarrow {\mathrm{g}}_{{\hat{\mathrm{d}}}_{\mathrm{ij}}}^{\mathrm{q}*}\le {\mathrm{g}}_{{\hat{\mathrm{d}}}_{\mathrm{ij}}}^{\mathrm{q}}$$
  where ${\uptheta }_{\mathrm{i}}>0$, ${\sum }_{\mathrm{i}=1}^{\mathrm{n}}{\uptheta }_{\mathrm{i}}=1$ and ${\upomega }_{\mathrm{j}}>0,$ ${\sum }_{\mathrm{j}=1}^{\mathrm{m}}{\upomega }_{\mathrm{j}}=1$. So,
  $${\left({\left(\left({\mathrm{g}}_{{\hat{\mathrm{d}}}_{\mathrm{ij}}}^{\mathrm{q}*}\right)\right)}^{{\sum }_{\mathrm{i}=1}^{\mathrm{n}}{\uptheta }_{\mathrm{i}}}\right)}^{{\sum }_{\mathrm{j}=1}^{\mathrm{m}}{\upomega }_{\mathrm{j}}}\le {\left({\left(\left({\mathrm{g}}_{{\hat{\mathrm{d}}}_{\mathrm{ij}}}^{\mathrm{q}}\right)\right)}^{{\sum }_{\mathrm{i}=1}^{\mathrm{n}}{\uptheta }_{\mathrm{i}}}\right)}^{{\sum }_{\mathrm{j}=1}^{\mathrm{m}}{\upomega }_{\mathrm{j}}}$$
  16 $$\Rightarrow 2{\left({\left(\left({\mathrm{g}}_{{\hat{\mathrm{d}}}_{\mathrm{ij}}}^{\mathrm{q}*}\right)\right)}^{{\sum }_{\mathrm{i}=1}^{\mathrm{n}}{\uptheta }_{\mathrm{i}}}\right)}^{{\sum }_{\mathrm{j}=1}^{\mathrm{m}}{\upomega }_{\mathrm{j}}}\le 2{\left({\left(\left({\mathrm{g}}_{{\hat{\mathrm{d}}}_{\mathrm{ij}}}^{\mathrm{q}}\right)\right)}^{{\sum }_{\mathrm{i}=1}^{\mathrm{n}}{\uptheta }_{\mathrm{i}}}\right)}^{{\sum }_{\mathrm{j}=1}^{\mathrm{m}}{\upomega }_{\mathrm{j}}}$$
(ii) $${\mathrm{g}}_{{\hat{\mathrm{d}}}_{\mathrm{ij}}}^{\mathrm{q}*}\le {\mathrm{g}}_{{\hat{\mathrm{d}}}_{\mathrm{ij}}}^{\mathrm{q}}$$
  $$\Rightarrow 2-{\mathrm{g}}_{{\hat{\mathrm{d}}}_{\mathrm{ij}}}^{\mathrm{q}}\le 2-{\mathrm{g}}_{{\hat{\mathrm{d}}}_{\mathrm{ij}}}^{\mathrm{q}*}$$
  $$\Rightarrow \left(2-{\mathrm{g}}_{{\hat{\mathrm{d}}}_{\mathrm{ij}}}^{\mathrm{q}}\right)+{\mathrm{g}}_{{\hat{\mathrm{d}}}_{\mathrm{ij}}}^{\mathrm{q}}\le \left(2-{\mathrm{g}}_{{\hat{\mathrm{d}}}_{\mathrm{ij}}}^{\mathrm{q}*}\right)+{\mathrm{g}}_{{\hat{\mathrm{d}}}_{\mathrm{ij}}}^{\mathrm{q}*}$$
  17 $$\begin{aligned}&\Rightarrow {\left({\left(\left(2-{\mathrm{g}}_{{\hat{\mathrm{d}}}_{\mathrm{ij}}}^{\mathrm{q}*}\right)\right)}^{{\sum }_{\mathrm{i}=1}^{\mathrm{n}}{\uptheta }_{\mathrm{i}}}\right)}^{{\sum }_{\mathrm{j}=1}^{\mathrm{m}}{\upomega }_{\mathrm{j}}}+{\left({\left(\left({\mathrm{g}}_{{\hat{\mathrm{d}}}_{\mathrm{ij}}}^{\mathrm{q}*}\right)\right)}^{{\sum }_{\mathrm{i}=1}^{\mathrm{n}}{\uptheta }_{\mathrm{i}}}\right)}^{{\sum }_{\mathrm{j}=1}^{\mathrm{m}}{\upomega }_{\mathrm{j}}}\\ &\ge {\left({\left(\left(2-{\mathrm{g}}_{{\hat{\mathrm{d}}}_{\mathrm{ij}}}^{\mathrm{q}}\right)\right)}^{{\sum }_{\mathrm{i}=1}^{\mathrm{n}}{\uptheta }_{\mathrm{i}}}\right)}^{{\sum }_{\mathrm{j}=1}^{\mathrm{m}}{\upomega }_{\mathrm{j}}}+{\left({\left(\left({\mathrm{g}}_{{\hat{\mathrm{d}}}_{\mathrm{ij}}}^{\mathrm{q}}\right)\right)}^{{\sum }_{\mathrm{i}=1}^{\mathrm{n}}{\uptheta }_{\mathrm{i}}}\right)}^{{\sum }_{\mathrm{j}=1}^{\mathrm{m}}{\upomega }_{\mathrm{j}}}\end{aligned}$$

From (16) and (17), we get

$$\begin{aligned}&\Rightarrow \frac{\sqrt[\mathrm{q}]{2\prod_{\mathrm{j}=1}^{\mathrm{m}}{\left({\prod_{\mathrm{i}=1}^{\mathrm{n}}\left({\mathrm{g}}_{{\hat{\mathrm{d}}}_{\mathrm{ij}}}^{\mathrm{q}*}\right)}^{{\uptheta }_{\mathrm{i}}}\right)}^{{\upomega }_{\mathrm{j}}}}}{\sqrt[\mathrm{q}]{\prod_{\mathrm{j}=1}^{\mathrm{m}}{\left(\prod_{\mathrm{i}=1}^{\mathrm{n}}{\left(2-{\mathrm{g}}_{{\hat{\mathrm{d}}}_{\mathrm{ij}}}^{\mathrm{q}*}\right)}^{{\uptheta }_{\mathrm{i}}}\right)}^{{\upomega }_{\mathrm{j}}}+ \prod_{\mathrm{j}=1}^{\mathrm{m}}{\left({\prod_{\mathrm{i}=1}^{\mathrm{n}}\left({\mathrm{g}}_{{\hat{\mathrm{d}}}_{\mathrm{ij}}}^{\mathrm{q}*}\right)}^{{\uptheta }_{\mathrm{i}}}\right)}^{{\upomega }_{\mathrm{j}}}}}\\ &\le \frac{\sqrt[\mathrm{q}]{2\prod_{\mathrm{j}=1}^{\mathrm{m}}{\left({\prod_{\mathrm{i}=1}^{\mathrm{n}}\left({\mathrm{g}}_{{\hat{\mathrm{d}}}_{\mathrm{ij}}}^{\mathrm{q}}\right)}^{{\uptheta }_{\mathrm{i}}}\right)}^{{\upomega }_{\mathrm{j}}}}}{\sqrt[\mathrm{q}]{\prod_{\mathrm{j}=1}^{\mathrm{m}}{\left(\prod_{\mathrm{i}=1}^{\mathrm{n}}{\left(2-{\mathrm{g}}_{{\hat{\mathrm{d}}}_{\mathrm{ij}}}^{\mathrm{q}}\right)}^{{\uptheta }_{\mathrm{i}}}\right)}^{{\upomega }_{\mathrm{j}}}+ \prod_{\mathrm{j}=1}^{\mathrm{m}}{\left({\prod_{\mathrm{i}=1}^{\mathrm{n}}\left({\mathrm{g}}_{{\hat{\mathrm{d}}}_{\mathrm{ij}}}^{\mathrm{q}}\right)}^{{\uptheta }_{\mathrm{i}}}\right)}^{{\upomega }_{\mathrm{j}}}}}\end{aligned}$$

$$\begin{aligned} & \Rightarrow \frac{\sqrt[\mathrm{q}]{2\prod_{\mathrm{j}=1}^{\mathrm{m}}{\left({\prod_{\mathrm{i}=1}^{\mathrm{n}}\left({\mathrm{g}}_{{\hat{\mathrm{d}}}_{\mathrm{ij}}}^{\mathrm{q}*}\right)}^{{\uptheta }_{\mathrm{i}}}\right)}^{{\upomega }_{\mathrm{j}}}}}{\sqrt[\mathrm{q}]{\prod_{\mathrm{j}=1}^{\mathrm{m}}{\left(\prod_{\mathrm{i}=1}^{\mathrm{n}}{\left(2-{\mathrm{g}}_{{\hat{\mathrm{d}}}_{\mathrm{ij}}}^{\mathrm{q}*}\right)}^{{\uptheta }_{\mathrm{i}}}\right)}^{{\upomega }_{\mathrm{j}}}+ \prod_{\mathrm{j}=1}^{\mathrm{m}}{\left({\prod_{\mathrm{i}=1}^{\mathrm{n}}\left({\mathrm{g}}_{{\hat{\mathrm{d}}}_{\mathrm{ij}}}^{\mathrm{q}*}\right)}^{{\uptheta }_{\mathrm{i}}}\right)}^{{\upomega }_{\mathrm{j}}}}}\\ &\quad\le \frac{\sqrt[\mathrm{q}]{2\prod_{\mathrm{j}=1}^{\mathrm{m}}{\left({\prod_{\mathrm{i}=1}^{\mathrm{n}}\left({\mathrm{g}}_{{\hat{\mathrm{d}}}_{\mathrm{ij}}}^{\mathrm{q}}\right)}^{{\uptheta }_{\mathrm{i}}}\right)}^{{\upomega }_{\mathrm{j}}}}}{\sqrt[\mathrm{q}]{\prod_{\mathrm{j}=1}^{\mathrm{m}}{\left(\prod_{\mathrm{i}=1}^{\mathrm{n}}{\left(2-{\mathrm{g}}_{{\hat{\mathrm{d}}}_{\mathrm{ij}}}^{\mathrm{q}}\right)}^{{\uptheta }_{\mathrm{i}}}\right)}^{{\upomega }_{\mathrm{j}}}+ \prod_{\mathrm{j}=1}^{\mathrm{m}}{\left({\prod_{\mathrm{i}=1}^{\mathrm{n}}\left({\mathrm{g}}_{{\hat{\mathrm{d}}}_{\mathrm{ij}}}^{\mathrm{q}}\right)}^{{\uptheta }_{\mathrm{i}}}\right)}^{{\upomega }_{\mathrm{j}}}}}\end{aligned}$$

So, it proved that

$$\mathrm{q}-\mathrm{ROFHSEWA}\left({\mathrm{J}}_{{\hat{\mathrm{d}}}_{11}}, {\mathrm{J}}_{{\hat{\mathrm{d}}}_{12}}, \dots ,{\mathrm{J}}_{{\hat{\mathrm{d}}}_{\mathrm{nm}}}\right)\le \mathrm{q}-\mathrm{ROFHSEWA}\left({\mathrm{J}}_{{\hat{\mathrm{d}}}_{11}}^{*}, {\mathrm{J}}_{{\hat{\mathrm{d}}}_{12}}^{*}, \dots ,{\mathrm{J}}_{{\hat{\mathrm{d}}}_{\mathrm{nm}}}^{*}\right).$$

## Einstein weighted geometric aggregation operator for q-rung orthopair fuzzy hypersoft set

This section will introduce a novel Einstein-weighted geometric aggregation operator for q-ROFHSNs with the most necessary properties.

### Definition

Let ${\mathrm{J}}_{{\hat{\mathrm{d}}}_{\mathrm{k}}}=\left({\mathrm{f}}_{{\hat{\mathrm{d}}}_{\mathrm{k}}}, {\mathrm{g}}_{{\hat{\mathrm{d}}}_{\mathrm{k}}}\right)$ be a collection of q-ROFHSNs; then the q-ROFHSEWG operator is defined as follows:

19 $$\mathrm{q}-\mathrm{ROFHSEWG }\left({\mathrm{J}}_{{\hat{\mathrm{d}}}_{11}}, {\mathrm{J}}_{{\hat{\mathrm{d}}}_{12}}, \dots ,{\mathrm{J}}_{{\hat{\mathrm{d}}}_{\mathrm{nm}}}\right)= {{\otimes}_{\mathrm{\upepsilon }}}_{\mathrm{j}=1}^{\mathrm{m}}{\left(\left({{\otimes}_{\mathrm{\upepsilon }}}_{\mathrm{i}=1}^{\mathrm{n}}{\left({\mathrm{J}}_{{\hat{\mathrm{d}}}_{\mathrm{ij}}}\right)}^{{\uptheta }_{\mathrm{i}}}\right)\right)}^{{\upomega }_{\mathrm{j}}}$$

where ${\uptheta }_{\mathrm{i}}$ and ${\upomega }_{\mathrm{j}}$ denote the weights, such as ${\uptheta }_{\mathrm{i}}>0$, ${\sum }_{\mathrm{i}=1}^{\mathrm{n}}{\uptheta }_{\mathrm{i}}=1$, and ${\upomega }_{\mathrm{j}}>0,$ ${\sum }_{\mathrm{j}=1}^{\mathrm{m}}{\upomega }_{\mathrm{j}}=1$.

### Theorem

Let ${\mathrm{J}}_{{\hat{\mathrm{d}}}_{\mathrm{k}}}=\left({\mathrm{f}}_{{\hat{\mathrm{d}}}_{\mathrm{k}}}, {\mathrm{g}}_{{\hat{\mathrm{d}}}_{\mathrm{k}}}\right)$ be a collection of q-ROFHSNs, then the attained aggregated value using Eq. (19) is specified as

$$\mathrm{q}-\mathrm{ROFHSEWG }\left({\mathrm{J}}_{{\hat{\mathrm{d}}}_{11}}, {\mathrm{J}}_{{\hat{\mathrm{d}}}_{12}}, \dots ,{\mathrm{J}}_{{\hat{\mathrm{d}}}_{\mathrm{nm}}}\right)= {{\otimes}_{\mathrm{\upepsilon }}}_{\mathrm{j}=1}^{\mathrm{m}}{\left(\left({{\otimes}_{\mathrm{\upepsilon }}}_{\mathrm{i}=1}^{\mathrm{n}}{\left({\mathrm{J}}_{{\hat{\mathrm{d}}}_{\mathrm{ij}}}\right)}^{{\uptheta }_{\mathrm{i}}}\right)\right)}^{{\upomega }_{\mathrm{j}}}$$

20 $$\begin{aligned}&=\Bigg\langle \frac{\sqrt[\mathrm{q}]{2\prod_{\mathrm{j}=1}^{\mathrm{m}}{\left({\prod }_{\mathrm{i}=1}^{\mathrm{n}}{\left({\mathrm{f}}_{{\hat{\mathrm{d}}}_{\mathrm{ij}}}^{\mathrm{q}}\right)}^{{\uptheta }_{\mathrm{i}}}\right)}^{{\upomega }_{\mathrm{j}}}}}{\sqrt[\mathrm{q}]{\prod_{\mathrm{j}=1}^{\mathrm{m}}{\left(\prod_{\mathrm{i}=1}^{\mathrm{n}}{\left(2-{\mathrm{f}}_{{\hat{\mathrm{d}}}_{\mathrm{ij}}}^{\mathrm{q}}\right)}^{{\uptheta }_{\mathrm{i}}}\right)}^{{\uplambda }_{\mathrm{j}}}+\prod_{\mathrm{j}=1}^{\mathrm{m}}{\left({\prod }_{\mathrm{i}=1}^{\mathrm{n}}{\left({\mathrm{f}}_{{\hat{\mathrm{d}}}_{\mathrm{ij}}}^{\mathrm{q}}\right)}^{{\uptheta }_{\mathrm{i}}}\right)}^{{\uplambda }_{\mathrm{j}}}}},\\&\quad \frac{\sqrt[\mathrm{q}]{\prod_{\mathrm{j}=1}^{\mathrm{m}}{\left({\prod }_{\mathrm{i}=1}^{\mathrm{n}}{\left(1+{\mathrm{g}}_{{\hat{\mathrm{d}}}_{\mathrm{ij}}}^{\mathrm{q}}\right)}^{{\uptheta }_{\mathrm{i}}}\right)}^{{\upomega }_{\mathrm{j}}}-\prod_{\mathrm{j}=1}^{\mathrm{m}}{\left({\prod }_{\mathrm{i}=1}^{\mathrm{n}}{\left(1-{\mathrm{g}}_{{\hat{\mathrm{d}}}_{\mathrm{ij}}}^{\mathrm{q}}\right)}^{{\uptheta }_{\mathrm{i}}}\right)}^{{\upomega }_{\mathrm{j}}}}}{\sqrt[\mathrm{q}]{\prod_{\mathrm{j}=1}^{\mathrm{m}}{\left({\prod }_{\mathrm{i}=1}^{\mathrm{n}}{\left(1+{\mathrm{g}}_{{\hat{\mathrm{d}}}_{\mathrm{ij}}}^{\mathrm{q}}\right)}^{{\uptheta }_{\mathrm{i}}}\right)}^{{\upomega }_{\mathrm{j}}}+ \prod_{\mathrm{j}=1}^{\mathrm{m}}{\left({\prod }_{\mathrm{i}=1}^{\mathrm{n}}{\left(1-{\mathrm{g}}_{{\hat{\mathrm{d}}}_{\mathrm{ij}}}^{\mathrm{q}}\right)}^{{\uptheta }_{\mathrm{i}}}\right)}^{{\upomega }_{\mathrm{j}}}}}\Bigg\rangle \end{aligned}$$

where ${\uptheta }_{\mathrm{i}}$ and ${\upomega }_{\mathrm{j}}$ denote the weighted vectors such as: ${\uptheta }_{\mathrm{i}}>0$, ${\sum }_{\mathrm{i}=1}^{\mathrm{n}}{\uptheta }_{\mathrm{i}}=1$, and ${\upomega }_{\mathrm{j}}>0,$ ${\sum }_{\mathrm{j}=1}^{\mathrm{m}}{\upomega }_{\mathrm{j}}=1$.

#### Proof

We will use mathematical induction to demonstrate the above result.

For $\mathrm{n }= 1$, we get ${\uptheta }_{\mathrm{i}}=1.$

$$\mathrm{q}-\mathrm{ROFHSEWG}\left({\mathrm{J}}_{{\hat{\mathrm{d}}}_{11}}, {\mathrm{J}}_{{\hat{\mathrm{d}}}_{12}}, \dots ,{\mathrm{J}}_{{\hat{\mathrm{d}}}_{\mathrm{nm}}}\right)= {{\otimes}_{\mathrm{\upepsilon }}}_{\mathrm{j}=1}^{\mathrm{m}}{\left({\mathrm{J}}_{{\hat{\mathrm{d}}}_{1\mathrm{j}}}\right)}^{{\upomega }_{\mathrm{j}}}$$

$$=\Bigg\langle \frac{\sqrt[\mathrm{q}]{2\prod_{\mathrm{j}=1}^{\mathrm{m}}{\left({\mathrm{f}}_{{\hat{\mathrm{d}}}_{1\mathrm{j}}}^{\mathrm{q}}\right)}^{{\upomega }_{\mathrm{j}}}}}{\sqrt[\mathrm{q}]{\prod_{\mathrm{j}=1}^{\mathrm{m}}{\left(2-{\mathrm{f}}_{{\hat{\mathrm{d}}}_{1\mathrm{j}}}^{\mathrm{q}}\right)}^{{\uplambda }_{\mathrm{j}}}+\prod_{\mathrm{j}=1}^{\mathrm{m}}{\left({\mathrm{f}}_{{\hat{\mathrm{d}}}_{1\mathrm{j}}}^{\mathrm{q}}\right)}^{{\uplambda }_{\mathrm{j}}}}} , \frac{\sqrt[\mathrm{q}]{\prod_{\mathrm{j}=1}^{\mathrm{m}}{\left(1+{\mathrm{g}}_{{\hat{\mathrm{d}}}_{1\mathrm{j}}}^{\mathrm{q}}\right)}^{{\upomega }_{\mathrm{j}}}-\prod_{\mathrm{j}=1}^{\mathrm{m}}{\left(1-{\mathrm{g}}_{{\hat{\mathrm{d}}}_{1\mathrm{j}}}^{\mathrm{q}}\right)}^{{\upomega }_{\mathrm{j}}}}}{\sqrt[\mathrm{q}]{\prod_{\mathrm{j}=1}^{\mathrm{m}}{\left(1+{\mathrm{g}}_{{\hat{\mathrm{d}}}_{1\mathrm{j}}}^{\mathrm{q}}\right)}^{{\upomega }_{\mathrm{j}}}+ \prod_{\mathrm{j}=1}^{\mathrm{m}}{\left(1-{\mathrm{g}}_{{\hat{\mathrm{d}}}_{1\mathrm{j}}}^{\mathrm{q}}\right)}^{{\upomega }_{\mathrm{j}}}}}\Bigg\rangle$$

$$\begin{aligned}&=\Bigg\langle \frac{\sqrt[\mathrm{q}]{2\prod_{\mathrm{j}=1}^{\mathrm{m}}{\left({\prod }_{\mathrm{i}=1}^{1}{\left({\mathrm{f}}_{{\hat{\mathrm{d}}}_{\mathrm{ij}}}^{\mathrm{q}}\right)}^{{\uptheta }_{\mathrm{i}}}\right)}^{{\upomega }_{\mathrm{j}}}}}{\sqrt[\mathrm{q}]{\prod_{\mathrm{j}=1}^{\mathrm{m}}{\left(\prod_{\mathrm{i}=1}^{1}{\left(2-{\mathrm{f}}_{{\hat{\mathrm{d}}}_{\mathrm{ij}}}^{\mathrm{q}}\right)}^{{\uptheta }_{\mathrm{i}}}\right)}^{{\uplambda }_{\mathrm{j}}}+\prod_{\mathrm{j}=1}^{\mathrm{m}}{\left({\prod }_{\mathrm{i}=1}^{1}{\left({\mathrm{f}}_{{\hat{\mathrm{d}}}_{\mathrm{ij}}}^{\mathrm{q}}\right)}^{{\uptheta }_{\mathrm{i}}}\right)}^{{\uplambda }_{\mathrm{j}}}}} ,\\ &\quad \frac{\sqrt[\mathrm{q}]{\prod_{\mathrm{j}=1}^{\mathrm{m}}{\left({\prod }_{\mathrm{i}=1}^{1}{\left(1+{\mathrm{g}}_{{\hat{d}}_{ij}}^{q}\right)}^{{\theta }_{i}}\right)}^{{\omega }_{j}}-\prod_{j=1}^{m}{\left({\prod }_{i=1}^{1}{\left(1-{g}_{{\hat{d}}_{ij}}^{q}\right)}^{{\theta }_{i}}\right)}^{{\omega }_{j}}}}{\sqrt[q]{\prod_{j=1}^{m}{\left({\prod }_{i=1}^{1}{\left(1+{g}_{{\hat{d}}_{ij}}^{q}\right)}^{{\theta }_{i}}\right)}^{{\omega }_{j}}+ \prod_{j=1}^{m}{\left({\prod }_{i=1}^{1}{\left(1-{g}_{{\hat{d}}_{ij}}^{q}\right)}^{{\theta }_{i}}\right)}^{{\omega }_{j}}}}\Bigg\rangle \end{aligned}$$

For $m = 1$, we get ${\omega }_{j}=1.$

$$q-ROFHSEWG\left({J}_{{\hat{d}}_{11}}, {J}_{{\hat{d}}_{12}}, \dots ,{J}_{{\hat{d}}_{nm}}\right)= {{\otimes}_{\mathrm{\upepsilon }}}_{i=1}^{n}{\left({J}_{{\hat{d}}_{i1}}\right)}^{{\theta }_{i}}$$

$$=\Bigg\langle \frac{\sqrt[q]{2{\prod }_{i=1}^{n}{\left({f}_{{\hat{d}}_{i1}}^{q}\right)}^{{\theta }_{i}}}}{\sqrt[q]{\prod_{i=1}^{n}{\left(2-{f}_{{\hat{d}}_{i1}}^{q}\right)}^{{\theta }_{i}}+{\prod }_{i=1}^{n}{\left({f}_{{\hat{d}}_{i1}}^{q}\right)}^{{\theta }_{i}}}} , \frac{\sqrt[q]{{\prod }_{i=1}^{n}{\left(1+{g}_{{\hat{d}}_{i1}}^{q}\right)}^{{\theta }_{i}}-{\prod }_{i=1}^{n}{\left(1-{g}_{{\hat{d}}_{i1}}^{q}\right)}^{{\theta }_{i}}}}{\sqrt[q]{{\prod }_{i=1}^{n}{\left(1+{g}_{{\hat{d}}_{i1}}^{q}\right)}^{{\theta }_{i}}+{\prod }_{i=1}^{n}{\left(1-{g}_{{\hat{d}}_{i1}}^{q}\right)}^{{\theta }_{i}}}}\Bigg\rangle$$

$$\begin{aligned}&=\Bigg\langle \frac{\sqrt[q]{2\prod_{j=1}^{1}{\left({\prod }_{i=1}^{n}{\left({f}_{{\hat{d}}_{ij}}^{q}\right)}^{{\theta }_{i}}\right)}^{{\omega }_{j}}}}{\sqrt[q]{\prod_{j=1}^{1}{\left(\prod_{i=1}^{n}{\left(2-{f}_{{\hat{d}}_{ij}}^{q}\right)}^{{\theta }_{i}}\right)}^{{\lambda }_{j}}+\prod_{j=1}^{1}{\left({\prod }_{i=1}^{n}{\left({f}_{{\hat{d}}_{ij}}^{q}\right)}^{{\theta }_{i}}\right)}^{{\lambda }_{j}}}},\\ &\quad \frac{\sqrt[q]{\prod_{j=1}^{1}{\left({\prod }_{i=1}^{n}{\left(1+{g}_{{\hat{d}}_{ij}}^{q}\right)}^{{\theta }_{i}}\right)}^{{\omega }_{j}}-\prod_{j=1}^{1}{\left({\prod }_{i=1}^{n}{\left(1-{g}_{{\hat{d}}_{ij}}^{q}\right)}^{{\theta }_{i}}\right)}^{{\omega }_{j}}}}{\sqrt[q]{\prod_{j=1}^{1}{\left({\prod }_{i=1}^{n}{\left(1+{g}_{{\hat{d}}_{ij}}^{q}\right)}^{{\theta }_{i}}\right)}^{{\omega }_{j}}+ \prod_{j=1}^{1}{\left({\prod }_{i=1}^{n}{\left(1-{g}_{{\hat{d}}_{ij}}^{q}\right)}^{{\theta }_{i}}\right)}^{{\omega }_{j}}}}\Bigg\rangle \end{aligned}$$

So, Eq. (20) is true for $n = 1$ and $m = 1$.

Assume that the equation grasps for $n$ = ${\delta }_{2}$, $m$ = ${\delta }_{1}+1$ and for $n$ = ${\delta }_{2}+1$, $m$ = ${\delta }_{1}$

$$\begin{aligned}{{\otimes}_{\mathrm{\upepsilon }}}_{j=1}^{{\delta }_{1}+1}{\left({{\otimes}_{\mathrm{\upepsilon }}}_{i=1}^{{\delta }_{2}}{\left({J}_{{\hat{d}}_{ij}}\right)}^{{\theta }_{i}}\right)}^{{\omega }_{j}}&=\Bigg\langle \frac{\sqrt[q]{2\prod_{j=1}^{{\delta }_{1}+1 }{\left({\prod }_{i=1}^{{\delta }_{2}}{\left({f}_{{\hat{d}}_{ij}}^{q}\right)}^{{\theta }_{i}}\right)}^{{\omega }_{j}}}}{\sqrt[q]{\prod_{j=1}^{{\delta }_{1}+1}{\left(\prod_{i=1}^{{\delta }_{2}}{\left(2-{f}_{{\hat{d}}_{ij}}^{q}\right)}^{{\theta }_{i}}\right)}^{{\omega }_{j}}+\prod_{j=1}^{{\delta }_{1}+1}{\left({\prod }_{i=1}^{{\delta }_{2}}{\left({f}_{{\hat{d}}_{ij}}^{q}\right)}^{{\theta }_{i}}\right)}^{{\omega }_{j}}}} , \\ &\quad\frac{\sqrt[q]{\prod_{j=1}^{{\delta }_{1}+1}{\left({\prod }_{i=1}^{{\delta }_{2}}{\left(1+{g}_{{\hat{d}}_{ij}}^{q}\right)}^{{\theta }_{i}}\right)}^{{\omega }_{j}}- \prod_{j=1}^{{\delta }_{1}+1}{\left({\prod }_{i=1}^{{\delta }_{2}}{\left(1-{g}_{{\hat{d}}_{ij}}^{q}\right)}^{{\theta }_{i}}\right)}^{{\omega }_{j}}}}{\sqrt[q]{\prod_{j=1}^{{\delta }_{1}+1}{\left({\prod }_{i=1}^{{\delta }_{2}}{\left(1+{g}_{{\hat{d}}_{ij}}^{q}\right)}^{{\theta }_{i}}\right)}^{{\omega }_{j}}+ \prod_{j=1}^{{\delta }_{1}+1}{\left({\prod }_{i=1}^{{\delta }_{2}}{\left(1-{g}_{{\hat{d}}_{ij}}^{q}\right)}^{{\theta }_{i}}\right)}^{{\omega }_{j}}}}\Bigg\rangle \end{aligned}$$

$$\begin{aligned}{{\otimes}_{\mathrm{\upepsilon }}}_{j=1}^{{\delta }_{1}}{\left({{\otimes}_{\mathrm{\upepsilon }}}_{i=1}^{{\delta }_{2}+1}{\left({J}_{{\hat{d}}_{ij}}\right)}^{{\theta }_{i}}\right)}^{{\omega }_{j}}&=\Bigg\langle \frac{\sqrt[q]{2\prod_{j=1}^{{\delta }_{1}}{\left({\prod }_{i=1}^{{\delta }_{2}+1 }{\left({f}_{{\hat{d}}_{ij}}^{q}\right)}^{{\theta }_{i}}\right)}^{{\omega }_{j}}}}{\sqrt[q]{\prod_{j=1}^{{\delta }_{1}}{\left(\prod_{i=1}^{{\delta }_{2}+1 }{\left(2-{f}_{{\hat{d}}_{ij}}^{q}\right)}^{{\theta }_{i}}\right)}^{{\omega }_{j}}+\prod_{j=1}^{{\delta }_{1}}{\left({\prod }_{i=1}^{{\delta }_{2}+1 }{\left({f}_{{\hat{d}}_{ij}}^{q}\right)}^{{\theta }_{i}}\right)}^{{\omega }_{j}}}} ,\\ &\quad \frac{\sqrt[q]{\prod_{j=1}^{{\delta }_{1}}{\left({\prod }_{i=1}^{{\delta }_{2}+1 }{\left(1+{g}_{{\hat{d}}_{ij}}^{q}\right)}^{{\theta }_{i}}\right)}^{{\omega }_{j}}- \prod_{j=1}^{{\delta }_{1}}{\left({\prod }_{i=1}^{{\delta }_{2}+1 }{\left(1-{g}_{{\hat{d}}_{ij}}^{q}\right)}^{{\theta }_{i}}\right)}^{{\omega }_{j}}}}{\sqrt[q]{\prod_{j=1}^{{\delta }_{1}}{\left({\prod }_{i=1}^{{\delta }_{2}+1 }{\left(1+{g}_{{\hat{d}}_{ij}}^{q}\right)}^{{\theta }_{i}}\right)}^{{\omega }_{j}}+ \prod_{j=1}^{{\delta }_{1}}{\left({\prod }_{i=1}^{{\delta }_{2}+1 }{\left(1-{g}_{{\hat{d}}_{ij}}^{q}\right)}^{{\theta }_{i}}\right)}^{{\omega }_{j}}}}\Bigg\rangle \end{aligned}$$

Now we show the Eq. (20) for $m$= ${\delta }_{1}+1$ and $n$ = ${\delta }_{2}+1$

$${{\otimes}_{\mathrm{\upepsilon }}}_{j=1}^{{\delta }_{1}+1}{\left({{\otimes}_{\mathrm{\upepsilon }}}_{i=1}^{{\delta }_{2}+1}{\left({J}_{{\hat{d}}_{ij}}\right)}^{{\theta }_{i}}\right)}^{{\omega }_{j}}={{\otimes}_{\mathrm{\upepsilon }}}_{j=1}^{{\delta }_{1}+1}{\left({{\otimes}_{\mathrm{\upepsilon }}}_{i=1}^{{\delta }_{2}}{{J}_{{\hat{d}}_{ij}}}^{{\theta }_{i}}{\otimes}_{\mathrm{\upepsilon }} {{J}_{{\hat{d}}_{({\delta }_{2}+1)j}}}^{{\theta }_{i+1}}\right)}^{{\omega }_{j}}$$

$$=\left({{\otimes}_{\mathrm{\upepsilon }}}_{j=1}^{{\delta }_{1}+1}{{\otimes}_{\mathrm{\upepsilon }}}_{i=1}^{{\delta }_{2}}{\left({J}_{{\hat{d}}_{ij}}\right)}^{{\theta }_{i}{\omega }_{j}}\right)\left({{\otimes}_{\mathrm{\upepsilon }}}_{j=1}^{{\delta }_{1}+1}{\left({J}_{{\hat{d}}_{({\delta }_{2}+1)j}}\right)}^{{\omega }_{j}{\theta }_{i+1}}\right)$$

$$\begin{aligned}&=\Bigg\langle \frac{\sqrt[q]{2\prod_{j=1}^{{\delta }_{1}+1}{\left({\prod }_{i=1}^{{\delta }_{2} }{\left({f}_{{\hat{d}}_{ij}}^{q}\right)}^{{\theta }_{i}}\right)}^{{\omega }_{j}}}}{\sqrt[q]{\prod_{j=1}^{{\delta }_{1}+1}{\left(\prod_{i=1}^{{\delta }_{2} }{\left(2-{f}_{{\hat{d}}_{ij}}^{q}\right)}^{{\theta }_{i}}\right)}^{{\omega }_{j}}+\prod_{j=1}^{{\delta }_{1}+1}{\left({\prod }_{i=1}^{{\delta }_{2} }{\left({f}_{{\hat{d}}_{ij}}^{q}\right)}^{{\theta }_{i}}\right)}^{{\omega }_{j}}}}\\&\quad{\otimes}_{\mathrm{\upepsilon }}\frac{\sqrt[q]{2\prod_{j=1}^{{\delta }_{1}+1}{\left({\left({f}_{{\hat{d}}_{{(\delta }_{2}+1)j}}^{q}\right)}^{{\theta }_{{\delta }_{2}+1}}\right)}^{{\omega }_{j}}}}{\sqrt[q]{\prod_{j=1}^{{\delta }_{1}+1}{\left({\left(2-{f}_{{\hat{d}}_{{(\delta }_{2}+1)j}}^{q}\right)}^{{\theta }_{{\delta }_{2}+1}}\right)}^{{\omega }_{j}}+\prod_{j=1}^{{\delta }_{1}+1}{\left({\left({f}_{{\hat{d}}_{{(\delta }_{2}+1)j}}^{q}\right)}^{{\theta }_{{\delta }_{2}+1}}\right)}^{{\omega }_{j}}}},\\&\quad \frac{\sqrt[q]{\prod_{j=1}^{{\delta }_{1}+1}{\left({\prod }_{i=1}^{{\delta }_{2} }{\left(1+{g}_{{\hat{d}}_{ij}}^{q}\right)}^{{\theta }_{i}}\right)}^{{\omega }_{j}}-\prod_{j=1}^{{\delta }_{1}+1}{\left({\prod }_{i=1}^{{\delta }_{2} }{\left(1-{g}_{{\hat{d}}_{ij}}^{q}\right)}^{{\theta }_{i}}\right)}^{{\omega }_{j}}}}{\sqrt[q]{\prod_{j=1}^{{\delta }_{1}+1}{\left({\prod }_{i=1}^{{\delta }_{2} }{\left(1+{g}_{{\hat{d}}_{ij}}^{q}\right)}^{{\theta }_{i}}\right)}^{{\omega }_{j}}+\prod_{j=1}^{{\delta }_{1}+1}{\left({\prod }_{i=1}^{{\delta }_{2} }{\left(1-{g}_{{\hat{d}}_{ij}}^{q}\right)}^{{\theta }_{i}}\right)}^{{\omega }_{j}}}}\\&\quad{\otimes}_{\mathrm{\upepsilon }}\frac{\sqrt[q]{\prod_{j=1}^{{\delta }_{1}+1}{\left({\left(1+{g}_{{\hat{d}}_{({\delta }_{2}+1)j}}^{q}\right)}^{{\theta }_{{\delta }_{2}+1}}\right)}^{{\omega }_{j}}-\prod_{j=1}^{{\delta }_{1}+1}{\left({\left(1-{g}_{{\hat{d}}_{({\delta }_{2}+1)j}}^{q}\right)}^{{\theta }_{{\delta }_{2}+1}}\right)}^{{\omega }_{j}}}}{\sqrt[q]{\prod_{j=1}^{{\delta }_{1}+1}{\left({\left(1+{g}_{{\hat{d}}_{({\delta }_{2}+1)j}}^{q}\right)}^{{\theta }_{{\delta }_{2}+1}}\right)}^{{\omega }_{j}}+\prod_{j=1}^{{\delta }_{1}+1}{\left({\left(1-{g}_{{\hat{d}}_{({\delta }_{2}+1)j}}^{q}\right)}^{{\theta }_{{\delta }_{2}+1}}\right)}^{{\omega }_{j}}}}\Bigg\rangle \end{aligned}$$

$$\begin{aligned}&=\Bigg\langle \frac{\sqrt[q]{2\prod_{j=1}^{{\delta }_{1}+1}{\left({\prod }_{i=1}^{{\delta }_{2}+1 }{\left({f}_{{\hat{d}}_{ij}}^{q}\right)}^{{\theta }_{i}}\right)}^{{\omega }_{j}}}}{\sqrt{\prod_{j=1}^{{\delta }_{1}+1}{\left(\prod_{i=1}^{{\delta }_{2}+1 }{\left(2-{f}_{{\hat{d}}_{ij}}^{2}\right)}^{{\theta }_{i}}\right)}^{{\omega }_{j}}+\prod_{j=1}^{{\delta }_{1}+1}{\left({\prod }_{i=1}^{{\delta }_{2}+1 }{\left({f}_{{\hat{d}}_{ij}}^{2}\right)}^{{\theta }_{i}}\right)}^{{\omega }_{j}}}},\\ &\quad \frac{\sqrt[q]{\prod_{j=1}^{{\delta }_{1}+1}{\left({\prod }_{i=1}^{{\delta }_{2}+1 }{\left(1+{g}_{{\hat{d}}_{ij}}^{q}\right)}^{{\theta }_{i}}\right)}^{{\omega }_{j}}-\prod_{j=1}^{{\delta }_{1}+1}{\left({\prod }_{i=1}^{{\delta }_{2}+1 }{\left(1-{g}_{{\hat{d}}_{ij}}^{q}\right)}^{{\theta }_{i}}\right)}^{{\omega }_{j}}}}{\sqrt[q]{\prod_{j=1}^{{\delta }_{1}+1}{\left({\prod }_{i=1}^{{\delta }_{2}+1 }{\left(1+{g}_{{\hat{d}}_{ij}}^{q}\right)}^{{\theta }_{i}}\right)}^{{\omega }_{j}}+ \prod_{j=1}^{{\delta }_{1}+1}{\left({\prod }_{i=1}^{{\delta }_{2}+1 }{\left(1-{g}_{{\hat{d}}_{ij}}^{q}s\right)}^{{\theta }_{i}}\right)}^{{\omega }_{j}}}}\Bigg\rangle \end{aligned}$$

$$={{\otimes}_{\mathrm{\upepsilon }}}_{j=1}^{{\delta }_{1}+1}{\left({{\otimes}_{\mathrm{\upepsilon }}}_{i=1}^{{\delta }_{2}+1}{\left({J}_{{\hat{d}}_{ij}}\right)}^{{\theta }_{i}}\right)}^{{\omega }_{j}}$$

So, it holds for $m$ = ${\delta }_{1}+1$ and $n$ = ${\delta }_{2}+1$.

### Example

Let $H={\{H}_{1}, {H}_{2}, {H}_{3}\}$ be a team of professionals with the most appropriate weighted vectors ${\theta }_{i}={\left(0.3, 0.4, 0.3\right) }^{T}$. The team of experts decided to buy a house under the set of attributes which are $A=\left\{{d}_{1}=infrastructure ,{d}_{2}=facilities ,{d}_{3}=sewerage system , {d}_{4}=security\right\}$. For the selection of house, the team of experts considered the multi sub-attributes of the deliberated parameters such as $\left\{{d}_{1}=Infrastructure= \left\{{d}_{11}=old style, {d}_{11}=new style\right\} ,{d}_{2}=Facilities=\left\{{d}_{21}=ho\mathrm{s}pital, {d}_{22}=school\right\} ,{d}_{3}=Sewerage system= \left\{{d}_{31}=excellent\right\}, {d}_{4}=Secuirty= \left\{{d}_{41}=excellent\right\}\right\}$. Let ${\mathfrak{L}}^{{^{\prime}}}$ = ${d}_{1}\times {d}_{2}\times {d}_{3}\times {d}_{4}$ represents the collection of multi-sub-attributes. $${\mathfrak{L}}^{{^{\prime}}}={d}_{1}\times {d}_{2}\times {d}_{3}\times {d}_{4}=\left\{{d}_{11}, {d}_{12}\right\}\times \left\{{d}_{21}, {d}_{22}\right\}\times \left\{{d}_{31}\right\}\times \left\{{d}_{42}\right\}=\left\{\begin{array}{c}\left({d}_{11}, {d}_{21}, {d}_{31}, {d}_{41}\right), \left({d}_{11}, {d}_{22}, {d}_{31}, {d}_{41}\right), \\ \left({d}_{12}, {d}_{21}, {d}_{31}, {d}_{41}\right), \left({d}_{12}, {d}_{22}, {d}_{31}, {d}_{41}\right)\end{array}\right\}=\left\{{\hat{d}}_{1},{\hat{d}}_{2}, {\hat{d}}_{3}, {\hat{d}}_{4}\right\}$$ describes the sub-attributes collection with weights ${\omega }_{j}={\left(0.2, 0.3, 0.4, 0.1\right)}^{T}$. The group of experts assumes rating values for each multi-sub-attribute in the form of q-ROFHSNs $\left({J}_{3\times 4} ,{\mathfrak{L}}^{{^{\prime}}}\right)={\left({f}_{{\hat{d}}_{ij}},{g}_{{\hat{d}}_{ij}}\right)}_{3\times 4}$ are given as follows:

$$\left({J}_{3\times 4}, {\mathfrak{L}}^{{^{\prime}}}\right)=\left[\begin{array}{cccc}\left(0.5, 0.3\right)& \left(0.8, 0.7\right)& \left(0.6, 0.3\right)& \left(0.2, 0.9\right)\\ \left(0.6, 0.3\right)& \left(0.4, 0.7\right)& \left(0.4, 0.5\right)& \left(0.5, 0.6\right)\\ \left(0.3, 0.4\right)& \left(0.6, 0.8\right)& \left(0.3, 0.9\right)& \left(0.2, 0.7\right)\end{array}\right]$$

As we know that

$$q-ROFHSEWG\left({J}_{{\hat{d}}_{11}}, {J}_{{\hat{d}}_{12}}, \dots ,{J}_{{\hat{d}}_{nm}}\right)= {{\otimes}_{\mathrm{\upepsilon }}}_{j=1}^{m}{\left(\left({{\otimes}_{\mathrm{\upepsilon }}}_{i=1}^{n}{\left({J}_{{\hat{d}}_{ij}}\right)}^{{\theta }_{i}}\right)\right)}^{{\omega }_{j}}$$

$$\begin{aligned}&=\Bigg\langle \frac{\sqrt[q]{2\prod_{j=1}^{m}{\left({\prod }_{i=1}^{n}{\left({f}_{{\hat{d}}_{ij}}^{q}\right)}^{{\theta }_{i}}\right)}^{{\omega }_{j}}}}{\sqrt[q]{\prod_{j=1}^{m}{\left(\prod_{i=1}^{n}{\left(2-{f}_{{\hat{d}}_{ij}}^{q}\right)}^{{\theta }_{i}}\right)}^{{\lambda }_{j}}+\prod_{j=1}^{m}{\left({\prod }_{i=1}^{n}{\left({f}_{{\hat{d}}_{ij}}^{q}\right)}^{{\theta }_{i}}\right)}^{{\lambda }_{j}}}},\\ &\quad \frac{\sqrt[q]{\prod_{j=1}^{m}{\left({\prod }_{i=1}^{n}{\left(1+{g}_{{\hat{d}}_{ij}}^{q}\right)}^{{\theta }_{i}}\right)}^{{\omega }_{j}}-\prod_{j=1}^{m}{\left({\prod }_{i=1}^{n}{\left(1-{g}_{{\hat{d}}_{ij}}^{q}\right)}^{{\theta }_{i}}\right)}^{{\omega }_{j}}}}{\sqrt[q]{\prod_{j=1}^{m}{\left({\prod }_{i=1}^{n}{\left(1+{g}_{{\hat{d}}_{ij}}^{q}\right)}^{{\theta }_{i}}\right)}^{{\omega }_{j}}+ \prod_{j=1}^{m}{\left({\prod }_{i=1}^{n}{\left(1-{g}_{{\hat{d}}_{ij}}^{q}\right)}^{{\theta }_{i}}\right)}^{{\omega }_{j}}}}\Bigg\rangle \end{aligned}$$

$$q-ROFHSEWG\left({J}_{{\hat{d}}_{11}}, {J}_{{\hat{d}}_{12}}, \dots ,{J}_{{\hat{d}}_{34}}\right)$$

$$\begin{aligned}&=\Bigg\langle \frac{\sqrt[q]{2\prod_{j=1}^{4}{\left({\prod }_{i=1}^{3}{\left({f}_{{\hat{d}}_{ij}}^{q}\right)}^{{\theta }_{i}}\right)}^{{\omega }_{j}}}}{\sqrt[q]{\prod_{j=1}^{4}{\left(\prod_{i=1}^{3}{\left(2-{f}_{{\hat{d}}_{ij}}^{q}\right)}^{{\theta }_{i}}\right)}^{{\lambda }_{j}}+\prod_{j=1}^{4}{\left({\prod }_{i=1}^{3}{\left({f}_{{\hat{d}}_{ij}}^{q}\right)}^{{\theta }_{i}}\right)}^{{\lambda }_{j}}}},\\ &\quad \frac{\sqrt[q]{\prod_{j=1}^{4}{\left({\prod }_{i=1}^{3}{\left(1+{g}_{{\hat{d}}_{ij}}^{q}\right)}^{{\theta }_{i}}\right)}^{{\omega }_{j}}-\prod_{j=1}^{4}{\left({\prod }_{i=1}^{3}{\left(1-{g}_{{\hat{d}}_{ij}}^{q}\right)}^{{\theta }_{i}}\right)}^{{\omega }_{j}}}}{\sqrt[q]{\prod_{j=1}^{4}{\left({\prod }_{i=1}^{3}{\left(1+{g}_{{\hat{d}}_{ij}}^{q}\right)}^{{\theta }_{i}}\right)}^{{\omega }_{j}}+ \prod_{j=1}^{4}{\left({\prod }_{i=1}^{3}{\left(1-{g}_{{\hat{d}}_{ij}}^{q}\right)}^{{\theta }_{i}}\right)}^{{\omega }_{j}}}}\Bigg\rangle \end{aligned}$$

$$=\Bigg\langle \begin{array}{c}\frac{\sqrt[3]{2\left[\begin{array}{c}{\left\{{\left(0.125\right)}^{0.3}{\left(0.216\right)}^{0.4}{\left(0.027\right)}^{0.3}\right\}}^{0.2}{\left\{{\left(0.512\right)}^{0.3}{\left(0.064\right)}^{0.4}{\left(0.216\right)}^{0.3}\right\}}^{0.3}\\ {\left\{{\left(0.216\right)}^{0.3}{\left(0.064\right)}^{0.4}{\left(0.027\right)}^{0.3}\right\}}^{0.4}{\left\{{\left(0.008\right)}^{0.3}{\left(0.125\right)}^{0.4}{\left(0.09\right)}^{0.3}\right\}}^{0.1}\end{array}\right]}}{\sqrt[3]{\begin{array}{c}\left[\begin{array}{c}{\left\{{\left(1.875\right)}^{0.3}{\left(1.784\right)}^{0.4}{\left(1.973\right)}^{0.3}\right\}}^{0.2}{\left\{{\left(1.488\right)}^{0.3}{\left(1.936\right)}^{0.4}{\left(1.784\right)}^{0.3}\right\}}^{0.3}\\ {\left\{{\left(1.784\right)}^{0.3}{\left(1.936\right)}^{0.4}{\left(1.973\right)}^{0.3}\right\}}^{0.4}{\left\{{\left(1.992\right)}^{0.3}{\left(1.875\right)}^{0.4}{\left(1.992\right)}^{0.3}\right\}}^{0.1}\end{array}\right]\\ +\\ \left[\begin{array}{c}{\left\{{\left(0.125\right)}^{0.3}{\left(0.216\right)}^{0.4}{\left(0.027\right)}^{0.3}\right\}}^{0.2}{\left\{{\left(0.512\right)}^{0.3}{\left(0.064\right)}^{0.4}{\left(0.216\right)}^{0.3}\right\}}^{0.3}\\ {\left\{{\left(0.216\right)}^{0.3}{\left(0.064\right)}^{0.4}{\left(0.027\right)}^{0.3}\right\}}^{0.4}{\left\{{\left(0.008\right)}^{0.3}{\left(0.125\right)}^{0.4}{\left(0.09\right)}^{0.3}\right\}}^{0.1}\end{array}\right]\end{array}}}, \\ \frac{\sqrt[3]{\begin{array}{c}\left[\begin{array}{c}{\left\{{\left(1.027\right)}^{0.3}{\left(1.027\right)}^{0.4}{\left(1.064\right)}^{0.3}\right\}}^{0.2}{\left\{{\left(1.343\right)}^{0.3}{\left(1.343\right)}^{0.4}{\left(1.512\right)}^{0.3}\right\}}^{0.3}\\ {\left\{{\left(1.027\right)}^{0.3}{\left(1.125\right)}^{0.4}{\left(1.729\right)}^{0.3}\right\}}^{0.4}{\left\{{\left(1.729\right)}^{0.3}{\left(1.216\right)}^{0.4}{\left(1.343\right)}^{0.3}\right\}}^{0.1}\end{array}\right]\\ -\\ \left[\begin{array}{c}\left[{\left\{{\left(0.973\right)}^{0.3}{\left(0.973\right)}^{0.4}{\left(0.936\right)}^{0.3}\right\}}^{0.2}\right.{\left\{{\left(0.657\right)}^{0.3}{\left(0.657\right)}^{0.4}{\left(0.488\right)}^{0.3}\right\}}^{0.3}\\ {\left\{{\left(0.973\right)}^{0.3}{\left(0.875\right)}^{0.4}{\left(0.271\right)}^{0.3}\right\}}^{0.4}{\left\{{\left(0.271\right)}^{0.3}{\left(0.784\right)}^{0.4}{\left(0.657\right)}^{0.3}\right\}}^{0.1}\end{array}\right]\end{array}}}{\sqrt[3]{\begin{array}{c}\left[\begin{array}{c}{\left\{{\left(1.027\right)}^{0.3}{\left(1.027\right)}^{0.4}{\left(1.064\right)}^{0.3}\right\}}^{0.2}{\left\{{\left(1.343\right)}^{0.3}{\left(1.343\right)}^{0.4}{\left(1.512\right)}^{0.3}\right\}}^{0.3}\\ {\left\{{\left(1.027\right)}^{0.3}{\left(1.125\right)}^{0.4}{\left(1.729\right)}^{0.3}\right\}}^{0.4}{\left\{{\left(1.729\right)}^{0.3}{\left(1.216\right)}^{0.4}{\left(1.343\right)}^{0.3}\right\}}^{0.1}\end{array}\right]\\ +\\ \left[\begin{array}{c}\left[{\left\{{\left(0.973\right)}^{0.3}{\left(0.973\right)}^{0.4}{\left(0.936\right)}^{0.3}\right\}}^{0.2}\right.{\left\{{\left(0.657\right)}^{0.3}{\left(0.657\right)}^{0.4}{\left(0.488\right)}^{0.3}\right\}}^{0.3}\\ {\left\{{\left(0.973\right)}^{0.3}{\left(0.875\right)}^{0.4}{\left(0.271\right)}^{0.3}\right\}}^{0.4}{\left\{{\left(0.271\right)}^{0.3}{\left(0.784\right)}^{0.4}{\left(0.657\right)}^{0.3}\right\}}^{0.1}\end{array}\right]\end{array}}}\end{array}\Bigg\rangle$$

$$=\Bigg\langle \begin{array}{c}\frac{\sqrt[3]{2\left[\begin{array}{c}{\left\{\left(0.5359\right)\left(0.5417\right)\left(0.3384\right)\right\}}^{0.2}{\left\{\left(0.8181\right)\left(0.3330\right)\left(0.6314\right)\right\}}^{0.3}\\ {\left\{\left(0.6314\right)\left(0.3330\right)\left(0.3384\right)\right\}}^{0.4}{\left\{\left(0.2349\right)\left(0.4353\right)\left(0.2349\right)\right\}}^{0.1}\end{array}\right]}}{\sqrt[3]{\begin{array}{c}\left[\begin{array}{c}{\left\{\left(1.2075\right)\left(1.2605\right)\left(1.2261\right)\right\}}^{0.2}{\left\{\left(1.1266\right)\left(1.3025\right)\left(1.1896\right)\right\}}^{0.3}\\ {\left\{\left(1.1896\right)\left(1.2605\right)\left(1.2261\right)\right\}}^{0.4}{\left\{\left(1.2297\right)\left(1.2590\right)\left(1.2297\right)\right\}}^{0.1}\end{array}\right]\\ +\\ \left[\begin{array}{c}{\left\{\left(0.5359\right)\left(0.5417\right)\left(0.3384\right)\right\}}^{0.2}{\left\{\left(0.8181\right)\left(0.3330\right)\left(0.6314\right)\right\}}^{0.3}\\ {\left\{\left(0.6314\right)\left(0.3330\right)\left(0.3384\right)\right\}}^{0.4}{\left\{\left(0.2349\right)\left(0.4353\right)\left(0.2349\right)\right\}}^{0.1}\end{array}\right]\end{array}}}, \\ \frac{\sqrt[3]{\begin{array}{c}\left[\begin{array}{c}{\left\{\left(1.0080\right)\left(1.0107\right)\left(1.0188\right)\right\}}^{0.2}{\left\{\left(1.0925\right)\left(1.1252\right)\left(1.1320\right)\right\}}^{0.3}\\ {\left\{\left(1.0080\right)\left(1.0482\right)\left(1.1785\right)\right\}}^{0.4}{\left\{\left(1.1785\right)\left(1.0814\right)\left(1.0925\right)\right\}}^{0.1}\end{array}\right]\\ -\\ \left[\begin{array}{c}\left[{\left\{\left(0.9918\right)\left(0.9891\right)\left(0.9904\right)\right\}}^{0.2}\right.{\left\{\left(0.8816\right)\left(0.8453\right)\left(0.8064\right)\right\}}^{0.3}\\ {\left\{\left(0.9918\right)\left(0.9479\right)\left(0.6759\right)\right\}}^{0.4}{\left\{\left(0.6759\right)\left(0.9072\right)\left(0.8816\right)\right\}}^{0.1}\end{array}\right]\end{array}}}{\sqrt[3]{\begin{array}{c}\left[\begin{array}{c}{\left\{\left(1.0080\right)\left(1.0107\right)\left(1.0188\right)\right\}}^{0.2}{\left\{\left(1.0925\right)\left(1.1252\right)\left(1.1320\right)\right\}}^{0.3}\\ {\left\{\left(1.0080\right)\left(1.0482\right)\left(1.1785\right)\right\}}^{0.4}{\left\{\left(1.1785\right)\left(1.0814\right)\left(1.0925\right)\right\}}^{0.1}\end{array}\right]\\ +\\ \left[\begin{array}{c}\left[{\left\{\left(0.9918\right)\left(0.9891\right)\left(0.9904\right)\right\}}^{0.2}\right.{\left\{\left(0.8816\right)\left(0.8453\right)\left(0.8064\right)\right\}}^{0.3}\\ {\left\{\left(0.9918\right)\left(0.9479\right)\left(0.6759\right)\right\}}^{0.4}{\left\{\left(0.6759\right)\left(0.9072\right)\left(0.8816\right)\right\}}^{0.1}\end{array}\right]\end{array}}}\end{array}\Bigg\rangle$$

$$=\Bigg\langle \begin{array}{c}\frac{\sqrt[3]{2\left[\begin{array}{c}\left(0.6287\right)\left(0.5997\right)\left(0.3474\right)\left(0.6887\right)\end{array}\right]}}{\sqrt[3]{\begin{array}{c}\left[\left(1.1329\right)\left(1.1819\right)\left(1.2758\right)\left(1.0665\right)\right]\\ +\\ \left[\begin{array}{c}\left(0.6287\right)\left(0.5997\right)\left(0.3474\right)\left(0.6887\right)\end{array}\right]\end{array}}}, \frac{\sqrt[3]{\begin{array}{c}\left[\left(1.0075\right)\left(1.1042\right)\left(1.0917\right)\left(1.0337\right)\right]\\ -\\ \left[\left(0.9942\right)\left(0.8583\right)\left(0.8341\right)\left(0.9003\right)\right]\end{array}}}{\sqrt[3]{\begin{array}{c}\left[\left(1.0075\right)\left(1.1042\right)\left(1.0917\right)\left(1.0337\right)\right]\\ +\\ \left[\left(0.9942\right)\left(0.8583\right)\left(0.8341\right)\left(0.9003\right)\right]\end{array}}}\end{array}\Bigg\rangle$$

$$=\Bigg\langle \begin{array}{c}\mathrm{0.3994,0.6895}\end{array}\Bigg\rangle .$$

### Theorem

Let ${J}_{{\hat{d}}_{ij}}=\Bigg\langle {f}_{{\hat{d}}_{ij}} , {g}_{{\hat{d}}_{ij}}\Bigg\rangle \forall i, j$ be a collection of q-ROFHSNs, then$q-ROFHSWG\left({J}_{{\hat{d}}_{11}}, {J}_{{\hat{d}}_{12}}, \dots ,{J}_{{\hat{d}}_{nm}}\right)\le q-ROFHSEWG\left({J}_{{\hat{d}}_{11}}, {J}_{{\hat{d}}_{12}}, \dots ,{J}_{{\hat{d}}_{nm}}\right)$. ${\theta }_{i}$ and ${\omega }_{j}$ signify the weight vectors, such as ${\theta }_{i}>0$, $\sum_{i=1}^{n}{\theta }_{i}=1$ and ${\omega }_{j}>0$,$\sum_{j=1}^{n}{\omega }_{j}=1$.

#### Proof

As we know that

$$\begin{aligned} & \sqrt[q]{\prod_{j=1}^{m}{\left(\prod_{i=1}^{n}{\left(2-{f}_{{\hat{d}}_{ij}}^{q}\right)}^{{\theta }_{i}}\right)}^{{\omega }_{j}}+\prod_{j=1}^{m}{\left({\prod }_{i=1}^{n}{\left({f}_{{\hat{d}}_{ij}}^{q}\right)}^{{\theta }_{i}}\right)}^{{\omega }_{j}}}\\ &\quad\le \sqrt[q]{\sum_{j=1}^{m}{\left(\sum_{i=1}^{n}{\left(2-{f}_{{\hat{d}}_{ij}}^{q}\right)}^{{\theta }_{i}}\right)}^{{\omega }_{j}}+\sum_{j=1}^{m}{\left({\sum }_{i=1}^{n}{\left({f}_{{\hat{d}}_{ij}}^{q}\right)}^{{\theta }_{i}}\right)}^{{\omega }_{j}}}\end{aligned}$$

$$\sqrt[q]{\sum_{j=1}^{m}{\left(\sum_{i=}^{n}{\left(2-\left({f}_{{\hat{d}}_{ij}}^{q}\right)\right)}^{{\theta }_{i}}\right)}^{{\omega }_{j}}+\sum_{j=1}^{m}{\left({\sum }_{i=1}^{n}{\left({f}_{{\hat{d}}_{ij}}^{q}\right)}^{{\theta }_{i}}\right)}^{{\omega }_{j}}}=\sqrt[q]{2}$$

$$\sqrt[q]{\prod_{j=1}^{m}{\left(\prod_{i=1}^{n}{\left(2-{f}_{{\hat{d}}_{ij}}^{q}\right)}^{{\theta }_{i}}\right)}^{{\omega }_{j}}+\prod_{j=1}^{m}{\left({\prod }_{i=1}^{n}{\left({f}_{{\hat{d}}_{ij}}^{q}\right)}^{{\theta }_{i}}\right)}^{{\omega }_{j}}} \le \sqrt[q]{2}$$

21 $$\frac{\sqrt[q]{2\prod_{j=1}^{m}{\left({\prod }_{i=1}^{n}{\left({f}_{{\hat{d}}_{ij}}^{q}\right)}^{{\theta }_{i}}\right)}^{{\omega }_{j}}}}{\sqrt[q]{\prod_{j=1}^{m}{\left(\prod_{i=1}^{n}{\left(2-{f}_{{\hat{d}}_{ij}}^{q}\right)}^{{\theta }_{i}}\right)}^{{\omega }_{j}}+\prod_{j=1}^{m}{\left({\prod }_{i=1}^{n}{\left({f}_{{\hat{d}}_{ij}}^{q}\right)}^{{\theta }_{i}}\right)}^{{\omega }_{j}}}}\ge \prod_{j=1}^{m}{\left({\prod }_{i=1}^{n}{\left({f}_{{\hat{d}}_{ij}}\right)}^{{\theta }_{i}}\right)}^{{\omega }_{j}}$$

Again

$$\begin{aligned}{}& \sqrt[q]{\prod_{j=1}^{m}{\left({\prod }_{i=1}^{n}{\left(1+{g}_{{\hat{d}}_{ij}}^{q}\right)}^{{\theta }_{i}}\right)}^{{\omega }_{j}}+\prod_{j=1}^{m}{\left({\prod }_{i=1}^{n}{\left(1-{g}_{{\hat{d}}_{ij}}^{q}\right)}^{{\theta }_{i}}\right)}^{{\omega }_{j}}}\\ &\quad\le \sqrt[q]{\sum_{j=1}^{m}{\left(\sum_{i=1}^{n}{\left(1+{g}_{{\hat{d}}_{ij}}^{q}\right)}^{{\theta }_{i}}\right)}^{{\omega }_{j}}+\sum_{j=1}^{m}{\left({\sum }_{i=1}^{n}{\left(1-{g}_{{\hat{d}}_{ij}}^{q}\right)}^{{\theta }_{i}}\right)}^{{\omega }_{j}}}\end{aligned}$$

$$\sqrt[q]{{\sum }_{j=1}^{m}{\left(\sum_{i=1}^{n}{\left(1+{g}_{{\hat{d}}_{ij}}^{q}\right)}^{{\theta }_{i}}\right)}^{{\omega }_{j}}+\sum_{j=1}^{m}{\left({\sum }_{i=1}^{n}{\left(\left(1-{g}_{{\hat{d}}_{ij}}^{q}\right)\right)}^{{\theta }_{i}}\right)}^{{\omega }_{j}}}=\sqrt[q]{2}$$

$$\sqrt[q]{\prod_{j=1}^{m}{\left({\prod }_{i=1}^{n}{\left(1+{g}_{{\hat{d}}_{ij}}^{q}\right)}^{{\theta }_{i}}\right)}^{{\omega }_{j}}+\prod_{j=1}^{m}{\left({\prod }_{i=1}^{n}{\left(1-{g}_{{\hat{d}}_{ij}}^{q}\right)}^{{\theta }_{i}}\right)}^{{\omega }_{j}}}\le \sqrt[q]{2}$$

22 $$\frac{\sqrt[q]{\prod_{j=1}^{m}{\left({\prod }_{i=1}^{n}{\left(1+{g}_{{\hat{d}}_{ij}}^{q}\right)}^{{\theta }_{i}}\right)}^{{\omega }_{j}}- \prod_{j=1}^{m}{\left({\prod }_{i=1}^{n}{\left(1-{g}_{{\hat{d}}_{ij}}^{q}\right)}^{{\theta }_{i}}\right)}^{{\omega }_{j}}}}{\sqrt[q]{\prod_{j=1}^{m}{\left({\prod }_{i=1}^{n}{\left(1+{g}_{{\hat{d}}_{ij}}^{q}\right)}^{{\theta }_{i}}\right)}^{{\lambda }_{j}}+ \prod_{j=1}^{m}{\left({\prod }_{i=1}^{n}{\left(1-{g}_{{\hat{d}}_{ij}}^{q}\right)}^{{\theta }_{i}}\right)}^{{\lambda }_{j}}}}\le \sqrt[q]{1-\prod_{j=1}^{m}{\left({\prod }_{i=1}^{n}{\left(1-{g}_{{\hat{d}}_{ij}}^{q}\right)}^{{\theta }_{i}}\right)}^{{\omega }_{j}}}$$

Let $$q-ROFHSWG\left({J}_{{\hat{d}}_{11}}, {J}_{{\hat{d}}_{12}}, \dots ,{J}_{{\hat{d}}_{nm}}\right)={J}_{{\hat{d}}_{ij}}=\left({f}_{{\hat{d}}_{ij}},{g}_{{\hat{d}}_{ij}}\right)$$ and $$q-ROFHSEWG\left({J}_{{\hat{d}}_{11}}, {J}_{{\hat{d}}_{12}}, \dots ,{J}_{{\hat{d}}_{nm}}\right)={{J}_{{\hat{d}}_{ij}}}^{\varepsilon }=\left({f}_{{{\hat{d}}_{ij}}^{\varepsilon }},{g}_{{{\hat{d}}_{ij}}^{\varepsilon }}\right)$$.

Then, (21) and (22) transformed into the following forms ${f}_{{\hat{d}}_{ij}}\le {f}_{{{\hat{d}}_{ij}}^{\varepsilon }}$ and ${g}_{{\hat{d}}_{ij}}\ge {g}_{{{\hat{d}}_{ij}}^{\varepsilon }}$.

So, $$\left({J}_{{\hat{d}}_{ij}}\right)={f}_{{\hat{d}}_{ij}}^{q}-{g}_{{\hat{d}}_{ij}}^{q}+\left(\frac{{e}^{{f}_{{\hat{d}}_{ij}}^{q}-{g}_{{\hat{d}}_{ij}}^{q}}}{{e}^{{f}_{{\hat{d}}_{ij}}^{q}-{g}_{{\hat{d}}_{ij}}^{q}}+1}-\frac{1}{2}\right){\mathrm{\beth }}_{{J}_{{\hat{d}}_{ij}}}^{q}\le {f}_{{\hat{d}}_{ij}^{\varepsilon }}^{q}-{g}_{{\hat{d}}_{ij}^{\varepsilon }}^{q}+\left(\frac{{e}^{{f}_{{\hat{d}}_{ij}^{\varepsilon }}^{q}-{g}_{{\hat{d}}_{ij}^{\varepsilon }}^{q}}}{{e}^{{f}_{{\hat{d}}_{ij}^{\varepsilon }}^{q}-{g}_{{\hat{d}}_{ij}^{\varepsilon }}^{q}}+1}-\frac{1}{2}\right){\mathrm{\beth }}_{{J}_{{\hat{d}}_{ij}^{\varepsilon }}}^{q}=S\left({{J}_{{\hat{d}}_{ij}}}^{\varepsilon }\right)$$. Hence, $$S\left({J}_{{\hat{d}}_{ij}}\right)\le S\left({{J}_{{\hat{d}}_{ij}}}^{\varepsilon }\right)$$

If $$S\left({J}_{{\hat{d}}_{ij}}\right)<S\left({{J}_{{\hat{d}}_{ij}}}^{\varepsilon }\right)$$, then

23 $$q-ROFHSWG\left({J}_{{\hat{d}}_{11}}, {J}_{{\hat{d}}_{12}}, \dots ,{J}_{{\hat{d}}_{nm}}\right)<q-ROFHSEWG\left({J}_{{\hat{d}}_{11}}, {J}_{{\hat{d}}_{12}}, \dots ,{J}_{{\hat{d}}_{nm}}\right)$$

If $S\left({J}_{{\hat{d}}_{ij}}\right)=S\left({{J}_{{\hat{d}}_{ij}}}^{\varepsilon }\right)$, then ${f}_{{\hat{d}}_{ij}}^{q}-{g}_{{\hat{d}}_{ij}}^{q}+\left(\frac{{e}^{{f}_{{\hat{d}}_{ij}}^{q}-{g}_{{\hat{d}}_{ij}}^{q}}}{{e}^{{f}_{{\hat{d}}_{ij}}^{q}-{g}_{{\hat{d}}_{ij}}^{q}}+1}-\frac{1}{2}\right){\mathrm{\beth }}_{{J}_{{\hat{d}}_{ij}}}^{q}={f}_{{\hat{d}}_{ij}^{\varepsilon }}^{q}-{g}_{{\hat{d}}_{ij}^{\varepsilon }}^{q}+\left(\frac{{e}^{{f}_{{\hat{d}}_{ij}^{\varepsilon }}^{q}-{g}_{{\hat{d}}_{ij}^{\varepsilon }}^{q}}}{{e}^{{f}_{{\hat{d}}_{ij}^{\varepsilon }}^{q}-{g}_{{\hat{d}}_{ij}^{\varepsilon }}^{q}}+1}-\frac{1}{2}\right){\mathrm{\beth }}_{{J}_{{\hat{d}}_{ij}^{\varepsilon }}}^{q}$, so ${f}_{{\hat{d}}_{ij}}={f}_{{{\hat{d}}_{ij}}^{\varepsilon }}$ and ${g}_{{\hat{d}}_{ij}}={g}_{{{\hat{d}}_{ij}}^{\varepsilon }}$.

Then,

24 $$q-ROFHSWG\left({J}_{{\hat{d}}_{11}}, {J}_{{\hat{d}}_{12}}, \dots ,{J}_{{\hat{d}}_{nm}}\right)= q-ROFHSEWG \left({J}_{{\hat{d}}_{11}}, {J}_{{\hat{d}}_{12}}, \dots ,{J}_{{\hat{d}}_{nm}}\right)$$

From (23) and (24), we get

$$q-ROFHSWG \left({J}_{{\hat{d}}_{11}}, {J}_{{\hat{d}}_{12}}, \dots ,{J}_{{\hat{d}}_{nm}}\right)\le q-ROFHSEWG\left({J}_{{\hat{d}}_{11}}, {J}_{{\hat{d}}_{12}}, \dots ,{J}_{{\hat{d}}_{nm}}\right).$$

### Example

Let $H={\{H}_{1}, {H}_{2}, {H}_{3}\}$ be a team of professionals with the most appropriate weighted vectors ${\theta }_{i}={\left(0.3, 0.4, 0.3\right) }^{T}$. The team of experts decided to buy a house under the set of attributes which are $A=\left\{{d}_{1}=infrastructure ,{d}_{2}=facilities ,{d}_{3}=sewerage system , {d}_{4}=security\right\}$. For the selection of house, the team of experts considered the multi sub-attributes of the deliberated parameters such as $\left\{{d}_{1}=Infrastructure= \left\{{d}_{11}=old style, {d}_{11}=new style\right\} ,{d}_{2}=Facilities=\left\{{d}_{21}=hospital, {d}_{22}=school\right\} ,{d}_{3}=Sewerage system= \left\{{d}_{31}=excellent\right\}, {d}_{4}=Secuirty= \left\{{d}_{41}=excellent\right\}\right\}$. Let ${\mathfrak{L}}^{{^{\prime}}}={d}_{1}\times {d}_{2}\times {d}_{3}\times {d}_{4}$ represents the collection of multi-sub-attributes. $${\mathfrak{L}}^{{^{\prime}}}={d}_{1}\times {d}_{2}\times {d}_{3}\times {d}_{4}=\left\{{d}_{11}, {d}_{12}\right\}\times \left\{{d}_{21}, {d}_{22}\right\}\times \left\{{d}_{31}\right\}\times \left\{{d}_{42}\right\}=\left\{\begin{array}{c}\left({d}_{11}, {d}_{21}, {d}_{31}, {d}_{41}\right), \left({d}_{11}, {d}_{22}, {d}_{31}, {d}_{41}\right), \\ \left({d}_{12}, {d}_{21}, {d}_{31}, {d}_{41}\right), \left({d}_{12}, {d}_{22}, {d}_{31}, {d}_{41}\right)\end{array}\right\}=\left\{{\hat{d}}_{1},{\hat{d}}_{2}, {\hat{d}}_{3}, {\hat{d}}_{4}\right\}$$ describes multi sub-attributes with weights ${\omega }_{j}={\left(0.2, 0.3, 0.4, 0.1\right)}^{T}$. The group of experts assumes rating values in a q-ROFHSNs form such as: $\left({J}_{3\times 4} ,{\mathfrak{L}}^{{^{\prime}}}\right)={\left({f}_{{\hat{d}}_{ij}},{g}_{{\hat{d}}_{ij}}\right)}_{3\times 4}$ are given as follows:

$$\left({J}_{3\times 4}, {\mathfrak{L}}^{{^{\prime}}}\right)=\left[\begin{array}{cccc}\left(0.5, 0.3\right)& \left(0.8, 0.7\right)& \left(0.6, 0.3\right)& \left(0.2, 0.9\right)\\ \left(0.6, 0.3\right)& \left(0.4, 0.7\right)& \left(0.4, 0.5\right)& \left(0.5, 0.6\right)\\ \left(0.3, 0.4\right)& \left(0.6, 0.8\right)& \left(0.3, 0.9\right)& \left(0.2, 0.7\right)\end{array}\right]$$

$$q-ROFHSWG\left({J}_{{\hat{d}}_{11}}, {J}_{{\hat{d}}_{12}}, \dots ,{J}_{{\hat{d}}_{nm}}\right)=\Bigg\langle \prod_{j=1}^{m}{\left(\prod_{i=1}^{n}{\left({f}_{{\hat{d}}_{ij}}\right)}^{{\theta }_{i}}\right)}^{{\omega }_{j}}, \sqrt[q]{1-\prod_{j=1}^{m}{\left(\prod_{i=1}^{n}{\left(1-{{g}_{{\hat{d}}_{ij}}}^{q}\right)}^{{\theta }_{i}}\right)}^{{\omega }_{j}}}\Bigg\rangle$$

For $$q=3$$.

$$\begin{aligned} & q-ROFHSWG \left({J}_{{\hat{d}}_{11}}, {J}_{{\hat{d}}_{12}}, \dots ,{J}_{{\hat{d}}_{34}}\right)\\ &\quad=\Bigg\langle \begin{array}{c}\left[\begin{array}{c}{\left\{{\left(0.5\right)}^{0.3}{\left(0.6\right)}^{0.4}{\left(0.3\right)}^{0.3}\right\}}^{0.2}{\left\{{\left(0.8\right)}^{0.3}{\left(0.4\right)}^{0.4}{\left(0.6\right)}^{0.3}\right\}}^{0.3}\\ {\left\{{\left(0.6\right)}^{0.3}{\left(0.4\right)}^{0.4}{\left(0.3\right)}^{0.3}\right\}}^{0.4}{\left\{{\left(0.2\right)}^{0.3}{\left(0.5\right)}^{0.4}{\left(0.2\right)}^{0.3}\right\}}^{0.1}\end{array}\right], \\ \sqrt[3]{1-\left[\begin{array}{c}\left[{\left\{{\left(0.973\right)}^{0.3}{\left(0.973\right)}^{0.4}{\left(0.936\right)}^{0.3}\right\}}^{0.2}\right.{\left\{{\left(0.657\right)}^{0.3}{\left(0.657\right)}^{0.4}{\left(0.488\right)}^{0.3}\right\}}^{0.3}\\ {\left\{{\left(0.973\right)}^{0.3}{\left(0.875\right)}^{0.4}{\left(0.271\right)}^{0.3}\right\}}^{0.4}{\left\{{\left(0.271\right)}^{0.3}{\left(0.784\right)}^{0.4}{\left(0.657\right)}^{0.3}\right\}}^{0.1}\end{array}\right]}\end{array}\Bigg\rangle \end{aligned}$$

$$=\Bigg\langle \begin{array}{c}\left[\left(0.8567\right)\left(0.8386\right)\left(0.7030\right)\left(0.8831\right)\right],\\ \sqrt[3]{1-\left[\left(0.9922\right)\left(0.8583\right)\left(0.8342\right)\left(0.9403\right)\right]}\end{array}\Bigg\rangle$$

$$=\langle 0.4460, 0.6951\rangle .$$

Hence, from Examples 4.3 and 4.5, it is proved that

$$\mathrm{q}-\mathrm{ROFHSWG}\left({\mathrm{J}}_{{\hat{\mathrm{d}}}_{11}}, {\mathrm{J}}_{{\hat{\mathrm{d}}}_{12}}, \dots ,{\mathrm{J}}_{{\hat{\mathrm{d}}}_{\mathrm{nm}}}\right)\le \mathrm{q}-\mathrm{ROFHSEWG}\left({\mathrm{J}}_{{\hat{\mathrm{d}}}_{11}}, {\mathrm{J}}_{{\hat{\mathrm{d}}}_{12}}, \dots ,{\mathrm{J}}_{{\hat{\mathrm{d}}}_{\mathrm{nm}}}\right).$$

### Properties of q-ROFHSEWG operator

#### Idempotency

If $${J}_{{\hat{d}}_{ij}}={J}_{{\hat{d}}_{k}}=\Bigg\langle {f}_{{\hat{d}}_{ij}} , {g}_{{\hat{d}}_{ij}}\Bigg\rangle \forall i, j,$$ then $$q-ROFHSEWG\left({J}_{{\hat{d}}_{11}}, {J}_{{\hat{d}}_{12}}, \dots ,{J}_{{\hat{d}}_{nm}}\right)={J}_{{\hat{d}}_{k}}$$.

##### Proof

As we know that

$$\begin{aligned} q-ROFHSEWG\left({J}_{{\hat{d}}_{11}}, {J}_{{\hat{d}}_{12}}, \dots ,{J}_{{\hat{d}}_{nm}}\right)&=\Bigg\langle \frac{\sqrt[q]{2\prod_{j=1}^{m}{\left({\prod }_{i=1}^{n}{\left({f}_{{\hat{d}}_{ij}}^{q}\right)}^{{\theta }_{i}}\right)}^{{\omega }_{j}}}}{\sqrt[q]{\prod_{j=1}^{m}{\left(\prod_{i=1}^{n}{\left(2-{f}_{{\hat{d}}_{ij}}^{q}\right)}^{{\theta }_{i}}\right)}^{{\lambda }_{j}}+\prod_{j=1}^{m}{\left({\prod }_{i=1}^{n}{\left({f}_{{\hat{d}}_{ij}}^{q}\right)}^{{\theta }_{i}}\right)}^{{\lambda }_{j}}}} ,\\ &\quad\quad \frac{\sqrt[q]{\prod_{j=1}^{m}{\left({\prod }_{i=1}^{n}{\left(1+{g}_{{\hat{d}}_{ij}}^{q}\right)}^{{\theta }_{i}}\right)}^{{\omega }_{j}}-\prod_{j=1}^{m}{\left({\prod }_{i=1}^{n}{\left(1-{g}_{{\hat{d}}_{ij}}^{q}\right)}^{{\theta }_{i}}\right)}^{{\omega }_{j}}}}{\sqrt[q]{\prod_{j=1}^{m}{\left({\prod }_{i=1}^{n}{\left(1+{g}_{{\hat{d}}_{ij}}^{q}\right)}^{{\theta }_{i}}\right)}^{{\omega }_{j}}+ \prod_{j=1}^{m}{\left({\prod }_{i=1}^{n}{\left(1-{g}_{{\hat{d}}_{ij}}^{q}\right)}^{{\theta }_{i}}\right)}^{{\omega }_{j}}}}\Bigg\rangle \end{aligned}$$

$$\begin{aligned}&=\Bigg\langle \frac{\sqrt[q]{2{\left({\left({f}_{{\hat{d}}_{ij}}^{q}\right)}^{\sum_{i=1}^{n}{\theta }_{i}}\right)}^{\sum_{j=1}^{m}{\omega }_{j}}}}{\sqrt[q]{{\left({\left(2-{f}_{{\hat{d}}_{ij}}^{q}\right)}^{\sum_{i=1}^{n}{\theta }_{i}}\right)}^{\sum_{j=1}^{m}{\omega }_{j}}+{\left({\left({f}_{{\hat{d}}_{ij}}^{q}\right)}^{\sum_{i=1}^{n}{\theta }_{i}}\right)}^{\sum_{j=1}^{m}{\omega }_{j}}}} ,\\ &\quad \frac{\sqrt[q]{{\left({\left(1+{g}_{{\hat{d}}_{ij}}^{q}\right)}^{\sum_{i=1}^{n}{\theta }_{i}}\right)}^{\sum_{j=1}^{m}{\omega }_{j}}-{\left({\left(1-{g}_{{\hat{d}}_{ij}}^{q}\right)}^{\sum_{i=1}^{n}{\theta }_{i}}\right)}^{\sum_{j=1}^{m}{\omega }_{j}}}}{\sqrt[q]{{\left({\left(1+{g}_{{\hat{d}}_{ij}}^{q}\right)}^{\sum_{i=1}^{n}{\theta }_{i}}\right)}^{\sum_{j=1}^{m}{\omega }_{j}}+{\left({\left(1-{g}_{{\hat{d}}_{ij}}^{q}\right)}^{\sum_{i=1}^{n}{\theta }_{i}}\right)}^{\sum_{j=1}^{m}{\omega }_{j}}}}\Bigg\rangle \end{aligned}$$

$$=\Bigg\langle \frac{\sqrt[q]{2{f}_{{\hat{d}}_{ij}}^{q}}}{\sqrt[q]{\left(2-{f}_{{\hat{d}}_{ij}}^{q}\right)+\left({f}_{{\hat{d}}_{ij}}^{q}\right)}} , \frac{\sqrt[q]{\left(1+{g}_{{\hat{d}}_{ij}}^{q}\right)-\left(1-{g}_{{\hat{d}}_{ij}}^{q}\right)}}{\sqrt[q]{\left(1+{g}_{{\hat{d}}_{ij}}^{q}\right)+\left(1-{g}_{{\hat{d}}_{ij}}^{q}\right)}}\Bigg\rangle$$

$$=\Bigg\langle {f}_{{\hat{d}}_{ij}}, {g}_{{\hat{d}}_{ij}}\Bigg\rangle ={J}_{{\hat{d}}_{k}.}$$

#### Boundedness

Let ${J}_{{\hat{d}}_{ij} }= \left({f}_{{\hat{d}}_{ij} }, {g}_{{\hat{d}}_{ij}}\right)$ be a collection of q-ROFHSNS and ${J}_{min}={J}_{{\hat{d}}_{ij} min}, {J}_{max}={J}_{{\hat{d}}_{ij} max}$. Then

$${J}_{{\hat{d}}_{ij} min}\le q-ROFHSEWG \le \left({J}_{{\hat{d}}_{11}}, {J}_{{\hat{d}}_{12}}, \dots ,{J}_{{\hat{d}}_{nm}}\right)\le {J}_{{\hat{d}}_{ij} max}.$$

##### Proof

Let $\mathrm{h}\left(\mathrm{x}\right)=\sqrt[\mathrm{q}]{\frac{2-{\mathrm{x}}^{\mathrm{q}}}{{\mathrm{x}}^{\mathrm{q}}}}$, $\mathrm{x}\in \left[0, 1\right]$, then $\frac{\mathrm{d}}{\mathrm{dx}}\left(\mathrm{h}(\mathrm{x})\right)=-\frac{1}{\mathrm{q}}{\left(\frac{2-{\mathrm{x}}^{\mathrm{q}}}{{\mathrm{x}}^{\mathrm{q}}}\right)}^{\frac{1}{\mathrm{q}}-1}\left(\frac{2}{{\left({\mathrm{x}}^{\mathrm{q}}\right)}^{2}}\right)$. So, $\frac{\mathrm{d}}{\mathrm{dx}}\left(\mathrm{k}(\mathrm{x})\right)=-\frac{1}{\mathrm{q}}{\left(\frac{2-{\mathrm{x}}^{\mathrm{q}}}{{\mathrm{x}}^{\mathrm{q}}}\right)}^{\frac{1}{\mathrm{q}}-1}\left(\frac{2}{{\left({\mathrm{x}}^{\mathrm{q}}\right)}^{2}}\right)<0$, which shows that $\mathrm{h}(\mathrm{x})$ is decreasing function on $\left[0, 1\right]$. So, $${\mathrm{f}}_{{\hat{\mathrm{d}}}_{\mathrm{ij}}\begin{array}{c}min\end{array}}\le {\mathrm{f}}_{{\hat{\mathrm{d}}}_{\mathrm{ij}}}\le {\mathrm{f}}_{{\hat{\mathrm{d}}}_{\mathrm{ij}}\mathrm{max}}$$ $\forall$ $\mathrm{i},\mathrm{j}$. Hence, $$\mathrm{h}\left({\mathrm{f}}_{{\hat{\mathrm{d}}}_{\mathrm{ij}}\begin{array}{c}max\end{array}}\right)\le h\left({f}_{{\hat{d}}_{ij}}\right)\le h\left({f}_{{\hat{d}}_{ij}min}\right)$$, $\forall$ $i,j$. We have

$$\begin{aligned} & \iff \sqrt[q]{{\left({\left(\frac{2-{f}_{{\hat{d}}_{ij}max}^{q}}{{f}_{{\hat{d}}_{ij}max}^{q}}\right)}^{\sum_{i=1}^{n}{\theta }_{i}}\right)}^{\sum_{j=1}^{m}{\omega }_{j}}}\le \sqrt[q]{\prod_{j=1}^{m}{\left({\prod }_{i=1}^{n}{\left(\frac{2-{f}_{{\hat{d}}_{ij}}^{q}}{{f}_{{\hat{d}}_{ij}}^{q}}\right)}^{{\theta }_{i}}\right)}^{{\omega }_{j}}}\\ &\quad\le \sqrt[q]{{\left({\left(\frac{2-{f}_{{\hat{d}}_{ij}min}^{q}}{{f}_{{\hat{d}}_{ij}min}^{q}}\right)}^{\sum_{i=1}^{n}{\theta }_{i}}\right)}^{\sum_{j=1}^{m}{\omega }_{j}}}\end{aligned}$$

$$\iff \sqrt[q]{{\left({\left(\frac{2-{f}_{{\hat{d}}_{ij}max}^{q}}{{f}_{{\hat{d}}_{ij}max}^{q}}\right)}^{\sum_{i=1}^{n}{\theta }_{i}}\right)}^{\sum_{j=1}^{m}{\omega }_{j}}}\le \sqrt[q]{\prod_{j=1}^{m}{\left({\prod }_{i=1}^{n}{\left(\frac{2-{f}_{{\hat{d}}_{ij}}^{q}}{{f}_{{\hat{d}}_{ij}}^{q}}\right)}^{{\theta }_{i}}\right)}^{{\omega }_{j}}}\le \sqrt[q]{{\left({\left(\frac{2-{f}_{{\hat{d}}_{ij}min}^{q}}{{f}_{{\hat{d}}_{ij}min}^{q}}\right)}^{\sum_{i=1}^{n}{\theta }_{i}}\right)}^{\sum_{j=1}^{m}{\omega }_{j}}}$$

$$\iff \sqrt[q]{\left(\frac{2-{f}_{{\hat{d}}_{ij}max}^{q}}{{f}_{{\hat{d}}_{ij}max}^{q}}\right)}\le \sqrt[q]{\prod_{j=1}^{m}{\left({\prod }_{i=1}^{n}{\left(\frac{2-{f}_{{\hat{d}}_{ij}}^{q}}{{f}_{{\hat{d}}_{ij}}^{q}}\right)}^{{\theta }_{i}}\right)}^{{\omega }_{j}}}\le \sqrt[q]{\left(\frac{2-{f}_{{\hat{d}}_{ij}min}^{q}}{{f}_{{\hat{d}}_{ij}min}^{q}}\right)}$$

$$\iff \sqrt[q]{1+\left(\frac{2-{f}_{{\hat{d}}_{ij}max}^{q}}{{f}_{{\hat{d}}_{ij}max}^{q}}\right)}\le \sqrt[q]{1+\prod_{j=1}^{m}{\left({\prod }_{i=1}^{n}{\left(\frac{2-{f}_{{\hat{d}}_{ij}}^{q}}{{f}_{{\hat{d}}_{ij}}^{q}}\right)}^{{\theta }_{i}}\right)}^{{\omega }_{j}}}\le \sqrt[q]{1+\left(\frac{2-{f}_{{\hat{d}}_{ij}min}^{q}}{{f}_{{\hat{d}}_{ij}min}^{q}}\right)}$$

$$\iff \sqrt[q]{\frac{2}{{f}_{{\hat{d}}_{ij}max}^{q}}}\le \sqrt[q]{1+\prod_{j=1}^{m}{\left({\prod }_{i=1}^{n}{\left(\frac{2-{f}_{{\hat{d}}_{ij}}^{q}}{{f}_{{\hat{d}}_{ij}}^{q}}\right)}^{{\theta }_{i}}\right)}^{{\omega }_{j}}}\le \sqrt[q]{\frac{2}{{f}_{{\hat{d}}_{ij}min}^{q}}}$$

$$\iff \sqrt[q]{\frac{{f}_{{\hat{d}}_{ij}min}^{q}}{2}}\le \frac{1}{\sqrt[q]{1+\prod_{j=1}^{m}{\left({\prod }_{i=1}^{n}{\left(\frac{2-{f}_{{\hat{d}}_{ij}}^{q}}{{f}_{{\hat{d}}_{ij}}^{q}}\right)}^{{\theta }_{i}}\right)}^{{\omega }_{j}}}}\le \sqrt[q]{\frac{{f}_{{\hat{d}}_{ij}max}^{q}}{2}}$$

$$\iff {f}_{{\hat{d}}_{ij}min}\le \frac{2}{\sqrt[q]{1+\prod_{j=1}^{m}{\left({\prod }_{i=1}^{n}{\left(\frac{2-{f}_{{\hat{d}}_{ij}}^{q}}{{f}_{{\hat{d}}_{ij}}^{q}}\right)}^{{\theta }_{i}}\right)}^{{\omega }_{j}}}}\le {f}_{{\hat{d}}_{ij}max}$$

25 $${f}_{{\hat{d}}_{ij}min}\le \frac{\sqrt{2\prod_{j=1}^{m}{\left({\prod }_{i=1}^{n}{\left({f}_{{\hat{d}}_{ij}}^{2}\right)}^{{\theta }_{i}}\right)}^{{\omega }_{j}}}}{\sqrt{\prod_{j=1}^{m}{\left(\prod_{i=1}^{n}{\left(2-{f}_{{\hat{d}}_{ij}}^{2}\right)}^{{\theta }_{i}}\right)}^{{\omega }_{j}}+\prod_{j=1}^{m}{\left({\prod }_{i=1}^{n}{\left({f}_{{\hat{d}}_{ij}}^{2}\right)}^{{\theta }_{i}}\right)}^{{\omega }_{j}}}}\le {f}_{{\hat{d}}_{ij}max}$$

Again, $k\left(y\right)=\sqrt[q]{\frac{1-{y}^{q}}{1+{y}^{q}}}$, $y\in \left[0, 1\right]$, then $\frac{d}{d\left(y\right)}h\left(y\right)=-\frac{1}{q}{\left(\frac{1-{y}^{q}}{1+{y}^{q}}\right)}^{\frac{1}{q}-1}\left\{\frac{q{y}^{2q-1}+q{y}^{2q-1}}{{\left(1+{y}^{q}\right)}^{2}}\right\}$ < 0, which shows that $h(y)$ is decreasing function on $\left[0, 1\right]$. So,

$${g}_{{\hat{d}}_{ij}min }\le {g}_{{\hat{d}}_{ij}}\le {g}_{{\hat{d}}_{ij}max}.\mathrm{Hence}, k\left({g}_{{\hat{d}}_{ij}max}\right)\le k\left({g}_{{\hat{d}}_{ij}}\right)\le k\left({g}_{{\hat{d}}_{ij}min}\right)\forall i,j.$$

$$\Rightarrow \sqrt[q]{\frac{1-{g}_{{\hat{d}}_{ij}max}^{q}}{1+{g}_{{\hat{d}}_{ij}max}^{q}}}\le \sqrt[q]{\frac{1-{g}_{{\hat{d}}_{ij}}^{q}}{1+{g}_{{\hat{d}}_{ij}}^{q}}}\le \sqrt[q]{\frac{1-{g}_{{\hat{d}}_{ij}min}^{q}}{1+{g}_{{\hat{d}}_{ij}min}^{q}}}$$

Let ${\uptheta }_{\mathrm{i}}$ and ${\upomega }_{\mathrm{j}}$ symbolize the weight vectors, such as ${\uptheta }_{\mathrm{i}}>0$, $\sum_{\mathrm{i}=1}^{\mathrm{n}}{\uptheta }_{\mathrm{i}}=1$ and ${\upomega }_{\mathrm{j}}>0$,$\sum_{\mathrm{j}=1}^{\mathrm{n}}{\upomega }_{\mathrm{j}}=1$. We have

$$\begin{aligned} & \iff \sqrt[q]{\prod_{j=1}^{m}{\left({\prod }_{i=1}^{n}{\left(\frac{1-{g}_{{\hat{d}}_{ij}max}^{q}}{1+{g}_{{\hat{d}}_{ij}max}^{q}}\right)}^{{\theta }_{i}}\right)}^{{\omega }_{j}}}\le \sqrt[q]{\prod_{j=1}^{m}{\left({\prod }_{i=1}^{n}{\left(\frac{1-{g}_{{\hat{d}}_{ij}}^{q}}{1+{g}_{{\hat{d}}_{ij}}^{q}}\right)}^{{\theta }_{i}}\right)}^{{\omega }_{j}}}\\ &\quad\le \sqrt[q]{\prod_{j=1}^{m}{\left({\prod }_{i=1}^{n}{\left(\frac{1-{g}_{{\hat{d}}_{ij}min}^{q}}{1+{g}_{{\hat{d}}_{ij}min}^{q}}\right)}^{{\theta }_{i}}\right)}^{{\omega }_{j}}}\end{aligned}$$

$$\begin{aligned} & \iff \sqrt[q]{{\left({\left(\frac{1-{g}_{{\hat{d}}_{ij}max}^{q}}{1+{g}_{{\hat{d}}_{ij}max}^{q}}\right)}^{\sum_{i=1}^{n}{\theta }_{i}}\right)}^{\sum_{j=1}^{m}{\omega }_{j}}}\le \sqrt[q]{\prod_{j=1}^{m}{\left({\prod }_{i=1}^{n}{\left(\frac{1-{g}_{{\hat{d}}_{ij}}^{q}}{1+{g}_{{\hat{d}}_{ij}}^{q}}\right)}^{{\theta }_{i}}\right)}^{{\omega }_{j}}}\\ &\quad\le \sqrt[q]{{\left({\left(\frac{1-{g}_{{\hat{d}}_{ij}min}^{q}}{1+{g}_{{\hat{d}}_{ij}min}^{q}}\right)}^{\sum_{i=1}^{n}{\theta }_{i}}\right)}^{\sum_{j=1}^{m}{\omega }_{j}}}\end{aligned}$$

$$\iff \sqrt[q]{\left(\frac{1-{g}_{{\hat{d}}_{ij}max}^{q}}{1+{g}_{{\hat{d}}_{ij}max}^{q}}\right)}\le \sqrt[q]{\prod_{j=1}^{m}{\left({\prod }_{i=1}^{n}{\left(\frac{1-{g}_{{\hat{d}}_{ij}}^{q}}{1+{g}_{{\hat{d}}_{ij}}^{q}}\right)}^{{\theta }_{i}}\right)}^{{\omega }_{j}}}\le \sqrt[q]{\left(\frac{1-{g}_{{\hat{d}}_{ij}min}^{q}}{1+{g}_{{\hat{d}}_{ij}min}^{q}}\right)}$$

$$\iff \sqrt[q]{1+\left(\frac{1-{g}_{{\hat{d}}_{ij}max}^{q}}{1+{g}_{{\hat{d}}_{ij}max}^{q}}\right)}\le \sqrt[q]{1+\prod_{j=1}^{m}{\left({\prod }_{i=1}^{n}{\left(\frac{1-{g}_{{\hat{d}}_{ij}}^{q}}{1+{g}_{{\hat{d}}_{ij}}^{q}}\right)}^{{\theta }_{i}}\right)}^{{\omega }_{j}}}\le \sqrt[q]{1+\left(\frac{1-{g}_{{\hat{d}}_{ij}min}^{q}}{1+{g}_{{\hat{d}}_{ij}min}^{q}}\right)}$$

$$\iff \sqrt[q]{\frac{2}{1+{g}_{{\hat{d}}_{ij}max}^{q}}}\le \sqrt[q]{1+\prod_{j=1}^{m}{\left({\prod }_{i=1}^{n}{\left(\frac{1-{g}_{{\hat{d}}_{ij}}^{q}}{1+{g}_{{\hat{d}}_{ij}}^{q}}\right)}^{{\theta }_{i}}\right)}^{{\omega }_{j}}}\le \sqrt[q]{\frac{2}{1+{g}_{{\hat{d}}_{ij}min}^{q}}}$$

$$\iff \sqrt[q]{\frac{1+{g}_{{\hat{d}}_{ij}min}^{q}}{2}}\le \frac{1}{\sqrt[q]{1+\prod_{j=1}^{m}{\left({\prod }_{i=1}^{n}{\left(\frac{1-{g}_{{\hat{d}}_{ij}}^{q}}{1+{g}_{{\hat{d}}_{ij}}^{q}}\right)}^{{\theta }_{i}}\right)}^{{\omega }_{j}}}}\le \sqrt[q]{\frac{1+{g}_{{\hat{d}}_{ij}max}^{q}}{2}}$$

$$\iff \sqrt[q]{1+{g}_{{\hat{d}}_{ij}min}^{q}}\le \sqrt[q]{\frac{2}{1+\prod_{j=1}^{m}{\left({\prod }_{i=1}^{n}{\left(\frac{1-{g}_{{\hat{d}}_{ij}}^{q}}{1+{g}_{{\hat{d}}_{ij}}^{q}}\right)}^{{\theta }_{i}}\right)}^{{\omega }_{j}}}}\le \sqrt[q]{1+{g}_{{\hat{d}}_{ij}max}^{q}}$$

$$\iff {g}_{{\hat{d}}_{ij}min}\le \sqrt[q]{\frac{2}{1+\prod_{j=1}^{m}{\left({\prod }_{i=1}^{n}{\left(\frac{1-{g}_{{\hat{d}}_{ij}}^{q}}{1+{g}_{{\hat{d}}_{ij}}^{q}}\right)}^{{\theta }_{i}}\right)}^{{\omega }_{j}}}-1}\le {g}_{{\hat{d}}_{ij}max}$$

26 $$\iff {g}_{{\hat{d}}_{ij}min}\le \frac{\sqrt[q]{\prod_{j=1}^{m}{\left({\prod }_{i=1}^{n}{\left(1+{g}_{{\hat{d}}_{ij}}^{q}\right)}^{{\theta }_{i}}\right)}^{{\omega }_{j}}-\prod_{j=1}^{m}{\left({\prod }_{i=1}^{n}{\left(1-{g}_{{\hat{d}}_{ij}}^{q}\right)}^{{\theta }_{i}}\right)}^{{\omega }_{j}}}}{\sqrt[q]{\prod_{j=1}^{m}{\left({\prod }_{i=1}^{n}{\left(1+{g}_{{\hat{d}}_{ij}}^{q}\right)}^{{\theta }_{i}}\right)}^{{\omega }_{j}}+ \prod_{j=1}^{m}{\left({\prod }_{i=1}^{n}{\left(1-{g}_{{\hat{d}}_{ij}}^{q}\right)}^{{\theta }_{i}}\right)}^{{\omega }_{j}}}}\le {g}_{{\hat{d}}_{ij}max}$$

Let $q-ROFHSEWG\left({J}_{{\hat{d}}_{11}}, {J}_{{\hat{d}}_{12}}, \dots ,{J}_{{\hat{d}}_{nm}}\right)={J}_{{\hat{d}}_{k}}$, then equations (25) and (26) can be written as ${f}_{{\hat{d}}_{ij}min} \le {f}_{{\hat{d}}_{ij}}\le {f}_{{\hat{d}}_{ij}max}$ and ${g}_{{\hat{d}}_{ij}max} \le {g}_{{\hat{d}}_{ij}}\le {g}_{{\hat{d}}_{ij}min}$. Thus, $S\left({J}_{{\hat{d}}_{k}}\right)={f}_{{\hat{d}}_{k}}^{q}-{g}_{{\hat{d}}_{k}}^{q}+\left(\frac{{e}^{{f}_{{\hat{d}}_{k}}^{q}-{g}_{{\hat{d}}_{k}}^{q}}}{{e}^{{f}_{{\hat{d}}_{k}}^{q}-{g}_{{\hat{d}}_{k}}^{q}}+1}-\frac{1}{2}\right){\mathrm{\beth }}_{{J}_{{\hat{d}}_{k}}}^{q}\le {\left(\genfrac{}{}{0pt}{}{max}{j}\genfrac{}{}{0pt}{}{max}{i}\left\{{f}_{{\hat{d}}_{k}}\right\}\right)}^{q}-{\left(\genfrac{}{}{0pt}{}{min}{j}\genfrac{}{}{0pt}{}{min}{i}\left\{{g}_{{\hat{d}}_{k}}\right\}\right)}^{q}+\left(\frac{{e}^{{\left(\genfrac{}{}{0pt}{}{max}{j}\genfrac{}{}{0pt}{}{max}{i}\left\{{f}_{{\hat{d}}_{k}}\right\}\right)}^{q}-{\left(\genfrac{}{}{0pt}{}{min}{j}\genfrac{}{}{0pt}{}{min}{i}\left\{{g}_{{\hat{d}}_{k}}\right\}\right)}^{q}}}{{e}^{{\left(\genfrac{}{}{0pt}{}{max}{j}\genfrac{}{}{0pt}{}{max}{i}\left\{{f}_{{\hat{d}}_{k}}\right\}\right)}^{q}-{\left(\genfrac{}{}{0pt}{}{min}{j}\genfrac{}{}{0pt}{}{min}{i}\left\{{g}_{{\hat{d}}_{k}}\right\}\right)}^{q}}+1}- \frac{1}{2}\right){{\mathrm{\beth }}_{{{J}_{{\hat{d}}_{k}}}^{+}}}^{q}=S\left({{J}_{{\hat{d}}_{k}}}_{max}\right)$ and $S\left({J}_{{\hat{d}}_{ij}}\right)={f}_{{\hat{d}}_{ij}}^{q}-{g}_{{\hat{d}}_{ij}}^{q}+\left(\frac{{e}^{{f}_{{\hat{d}}_{ij}}^{q}-{g}_{{\hat{d}}_{ij}}^{q}}}{{e}^{{f}_{{\hat{d}}_{ij}}^{q}-{g}_{{\hat{d}}_{ij}}^{q}}+1}-\frac{1}{2}\right){\mathrm{\beth }}_{{J}_{{\hat{d}}_{ij}}}^{q}\ge {\left(\genfrac{}{}{0pt}{}{min}{j}\genfrac{}{}{0pt}{}{min}{i}\left\{{f}_{{\hat{d}}_{ij}}\right\}\right)}^{q}-{\left(\genfrac{}{}{0pt}{}{max}{j}\genfrac{}{}{0pt}{}{max}{i}\left\{{g}_{{\hat{d}}_{ij}}\right\}\right)}^{q}+\left(\frac{{e}^{{\left(\genfrac{}{}{0pt}{}{min}{j}\genfrac{}{}{0pt}{}{min}{i}\left\{{f}_{{\hat{d}}_{ij}}\right\}\right)}^{q}-{\left(\genfrac{}{}{0pt}{}{max}{j}\genfrac{}{}{0pt}{}{max}{i}\left\{{g}_{{\hat{d}}_{ij}}\right\}\right)}^{q}}}{{e}^{{\left(\genfrac{}{}{0pt}{}{min}{j}\genfrac{}{}{0pt}{}{min}{i}\left\{{f}_{{\hat{d}}_{ij}}\right\}\right)}^{q}-{\left(\genfrac{}{}{0pt}{}{max}{j}\genfrac{}{}{0pt}{}{max}{i}\left\{{g}_{{\hat{d}}_{ij}}\right\}\right)}^{q}}+1}- \frac{1}{2}\right){{\mathrm{\beth }}_{{{J}_{{\hat{d}}_{ij}}}^{-}}}^{q}=S\left({{J}_{{\hat{d}}_{ij}}}_{min}\right).$

$$\Rightarrow S\left({J}_{{\hat{d}}_{k}}\right)\le S\left({J}_{{\hat{d}}_{ij}max}\right)$$ also

$$S\left({J}_{{\hat{d}}_{k}}\right)={f}_{{\hat{d}}_{k}}^{q}-{g}_{{\hat{d}}_{k}}^{q}+\left(\frac{{e}^{{f}_{{\hat{d}}_{k}}^{q}-{g}_{{\hat{d}}_{k}}^{q}}}{{e}^{{f}_{{\hat{d}}_{k}}^{q}-{g}_{{\hat{d}}_{k}}^{q}}+1}-\frac{1}{2}\right){\mathrm{\beth }}_{{J}_{{\hat{d}}_{k}}}^{q}\ge {\left(\genfrac{}{}{0pt}{}{min}{j}\genfrac{}{}{0pt}{}{min}{i}\left\{{f}_{{\hat{d}}_{ij}}\right\}\right)}^{q}-{\left(\genfrac{}{}{0pt}{}{max}{j}\genfrac{}{}{0pt}{}{max}{i}\left\{{g}_{{\hat{d}}_{ij}}\right\}\right)}^{q}+\left(\frac{{e}^{{\left(\genfrac{}{}{0pt}{}{min}{j}\genfrac{}{}{0pt}{}{min}{i}\left\{{f}_{{\hat{d}}_{ij}}\right\}\right)}^{q}-{\left(\genfrac{}{}{0pt}{}{max}{j}\genfrac{}{}{0pt}{}{max}{i}\left\{{g}_{{\hat{d}}_{ij}}\right\}\right)}^{q}}}{{e}^{{\left(\genfrac{}{}{0pt}{}{min}{j}\genfrac{}{}{0pt}{}{min}{i}\left\{{f}_{{\hat{d}}_{ij}}\right\}\right)}^{q}-{\left(\genfrac{}{}{0pt}{}{max}{j}\genfrac{}{}{0pt}{}{max}{i}\left\{{g}_{{\hat{d}}_{ij}}\right\}\right)}^{q}}+1}- \frac{1}{2}\right){{\mathrm{\beth }}_{{{J}_{{\hat{d}}_{ij}}}^{-}}}^{q}=S\left({J}_{{\hat{d}}_{ij}min}\right)$$

$$\Rightarrow S\left({J}_{{\hat{d}}_{k}}\right)\ge S\left({J}_{{\hat{d}}_{ij}min}\right).$$

If $$S\left( {J}_{{\hat{d}}_{k}}\right)< S\left({J}_{{\hat{d}}_{ij}max}\right) and S\left( {J}_{{\hat{d}}_{k}}\right)> S\left( {J}_{{\hat{d}}_{ij}min}\right)$$, then

27 $${J}_{{\hat{d}}_{ij}min}< q-ROFHSEWG\left({J}_{{\hat{d}}_{11}}, {J}_{{\hat{d}}_{12}}, \dots ,{J}_{{\hat{d}}_{nm}}\right)<{J}_{{\hat{d}}_{ij}max}$$

If $$S\left({J}_{{\hat{d}}_{k}}\right)=S\left({J}_{{\hat{d}}_{ij}max}\right),$$ then$${f}_{{\hat{d}}_{k}}^{q}-{g}_{{\hat{d}}_{k}}^{q}+\left(\frac{{e}^{{f}_{{\hat{d}}_{k}}^{q}-{g}_{{\hat{d}}_{k}}^{q}}}{{e}^{{f}_{{\hat{d}}_{k}}^{q}-{g}_{{\hat{d}}_{k}}^{q}}+1}-\frac{1}{2}\right){\mathrm{\beth }}_{{J}_{{\hat{d}}_{k}}}^{q}\le {\left(\genfrac{}{}{0pt}{}{max}{j}\genfrac{}{}{0pt}{}{max}{i}\left\{{f}_{{\hat{d}}_{ij}}\right\}\right)}^{q}-{\left(\genfrac{}{}{0pt}{}{min}{j}\genfrac{}{}{0pt}{}{min}{i}\left\{{g}_{{\hat{d}}_{ij}}\right\}\right)}^{q}+\left(\frac{{e}^{{\left(\genfrac{}{}{0pt}{}{max}{j}\genfrac{}{}{0pt}{}{max}{i}\left\{{f}_{{\hat{d}}_{ij}}\right\}\right)}^{q}-{\left(\genfrac{}{}{0pt}{}{min}{j}\genfrac{}{}{0pt}{}{min}{i}\left\{{g}_{{\hat{d}}_{ij}}\right\}\right)}^{q}}}{{e}^{{\left(\genfrac{}{}{0pt}{}{max}{j}\genfrac{}{}{0pt}{}{max}{i}\left\{{f}_{{\hat{d}}_{ij}}\right\}\right)}^{q}-{\left(\genfrac{}{}{0pt}{}{min}{j}\genfrac{}{}{0pt}{}{min}{i}\left\{{g}_{{\hat{d}}_{ij}}\right\}\right)}^{q}}+1}- \frac{1}{2}\right){{\mathrm{\beth }}_{{{J}_{{\hat{d}}_{ij}}}^{+}}}^{q}, \text{using the above inequalities}$$

$${f}_{{\hat{d}}_{k}}= \genfrac{}{}{0pt}{}{max}{j}\genfrac{}{}{0pt}{}{max}{i}\left\{{f}_{{\hat{d}}_{ij}}\right\},\mathrm{ and }{g}_{{\hat{d}}_{k}}= \genfrac{}{}{0pt}{}{min}{j}\genfrac{}{}{0pt}{}{min}{i}\left\{{g}_{{\hat{d}}_{ij}}\right\}.\mathrm{ Hence}, {{\mathrm{\beth }}_{{J}_{{\hat{d}}_{k}}}}^{q}={{\mathrm{\beth }}_{{{J}_{{\hat{d}}_{ij}}}^{+}}}^{q}.\mathrm{ Then }$$

28 $$q-ROFHSEWG\left({J}_{{\hat{d}}_{11}}, {J}_{{\hat{d}}_{12}}, \dots ,{J}_{{\hat{d}}_{nm}}\right)={J}_{{\hat{d}}_{ij}max}.$$

If $$S\left({J}_{{\hat{d}}_{k}}\right)=S\left({J}_{{\hat{d}}_{ij}min}\right),$$ then

$${f}_{{\hat{d}}_{k}}^{q}-{g}_{{\hat{d}}_{k}}^{q}+\left(\frac{{e}^{{f}_{{\hat{d}}_{k}}^{q}-{g}_{{\hat{d}}_{k}}^{q}}}{{e}^{{f}_{{\hat{d}}_{k}}^{q}-{g}_{{\hat{d}}_{k}}^{q}}+1}-\frac{1}{2}\right){\mathrm{\beth }}_{{J}_{{\hat{d}}_{k}}}^{q}\le {\left(\genfrac{}{}{0pt}{}{min}{j}\genfrac{}{}{0pt}{}{min}{i}\left\{{f}_{{\hat{d}}_{ij}}\right\}\right)}^{q}-{\left(\genfrac{}{}{0pt}{}{max}{j}\genfrac{}{}{0pt}{}{max}{i}\left\{{g}_{{\hat{d}}_{ij}}\right\}\right)}^{q}+\left(\frac{{e}^{{\left(\genfrac{}{}{0pt}{}{min}{j}\genfrac{}{}{0pt}{}{min}{i}\left\{{f}_{{\hat{d}}_{ij}}\right\}\right)}^{q}-{\left(\genfrac{}{}{0pt}{}{max}{j}\genfrac{}{}{0pt}{}{max}{i}\left\{{g}_{{\hat{d}}_{ij}}\right\}\right)}^{q}}}{{e}^{{\left(\genfrac{}{}{0pt}{}{min}{j}\genfrac{}{}{0pt}{}{min}{i}\left\{{f}_{{\hat{d}}_{ij}}\right\}\right)}^{q}-{\left(\genfrac{}{}{0pt}{}{max}{j}\genfrac{}{}{0pt}{}{max}{i}\left\{{g}_{{\hat{d}}_{ij}}\right\}\right)}^{q}}+1}- \frac{1}{2}\right){{\mathrm{\beth }}_{{{J}_{{\hat{d}}_{ij}}}^{-}}}^{q},\mathrm{ using the above inequalities}$$

${f}_{{\hat{d}}_{k}}= \genfrac{}{}{0pt}{}{min}{j}\genfrac{}{}{0pt}{}{min}{i}\left\{{f}_{{\hat{d}}_{ij}}\right\}$, and ${g}_{{\hat{d}}_{k}}= \genfrac{}{}{0pt}{}{max}{j}\genfrac{}{}{0pt}{}{max}{i}\left\{{g}_{{\hat{d}}_{ij}}\right\}$. Hence, ${{\mathrm{\beth }}_{{J}_{{\hat{d}}_{k}}}}^{q}={{\mathrm{\beth }}_{{{J}_{{\hat{d}}_{ij}}}^{-}}}^{q}$. Then

27 $$q-ROFHSEWG\left({J}_{{\hat{d}}_{11}}, {J}_{{\hat{d}}_{12}}, \dots ,{J}_{{\hat{d}}_{nm}}\right)={J}_{{\hat{d}}_{ij}min}.$$

So, it is proven that

$${\mathrm{J}}_{{\hat{\mathrm{d}}}_{\mathrm{ij}}\mathrm{min}}\le \mathrm{ q}-\mathrm{ROFHSEWG }\left({\mathrm{J}}_{{\hat{\mathrm{d}}}_{11}}, {\mathrm{J}}_{{\hat{\mathrm{d}}}_{12}}, \dots ,{\mathrm{J}}_{{\hat{\mathrm{d}}}_{\mathrm{nm}}}\right)\le {\mathrm{J}}_{{\hat{\mathrm{d}}}_{\mathrm{ij}}\mathrm{max}}.$$

#### Homogeneity

Prove that $q-ROFHSEWG\left({J}_{{\hat{d}}_{11}}, {J}_{{\hat{d}}_{12}}, \dots ,{J}_{{\hat{d}}_{nm}}\right)=\gamma q-ROFHSEWG \left({J}_{{\hat{d}}_{11}}, {J}_{{\hat{d}}_{12}}, \dots ,{J}_{{\hat{d}}_{nm}}\right)$ for $\gamma >0.$

##### Proof

Let ${J}_{{\hat{d}}_{ij}}$ be a q-ROFHSN and $\gamma >0$, then

$$\gamma {J}_{{\hat{d}}_{ij}}=\Bigg\langle \frac{\sqrt[q]{{\left(1+{{f}_{{\hat{d}}_{ij}}}^{q}\right)}^{\gamma }-{\left(1-{{f}_{{\hat{d}}_{ij}}}^{q}\right)}^{\gamma }}}{\sqrt[q]{{\left(1+{{f}_{{\hat{d}}_{ij}}}^{q}\right)}^{\gamma }+{\left(1-{{f}_{{\hat{d}}_{ij}}}^{q}\right)}^{\gamma }}} , \frac{\sqrt[q]{{2\left({{g}_{{\hat{d}}_{ij}}}^{q}\right)}^{\gamma }}}{\sqrt[q]{{\left(2-{{g}_{{\hat{d}}_{ij}}}^{q}\right)}^{\gamma }+{\left({{g}_{{\hat{d}}_{ij}}}^{q}\right)}^{\gamma }}}\Bigg\rangle$$

So,

$$\begin{aligned} & q-ROFHSEWG \left(\gamma {J}_{{\hat{d}}_{11}}, \gamma {J}_{{\hat{d}}_{12}},...,\gamma {J}_{{\hat{d}}_{nm}}\right) \\&\quad=\Bigg\langle \frac{\sqrt[q]{2\prod_{j=1}^{m}{\left({\prod }_{i=1}^{n}{\left({f}_{{\hat{d}}_{ij}}^{q}\right)}^{\gamma {\theta }_{i}}\right)}^{{\omega }_{j}}}}{\sqrt[q]{\prod_{j=1}^{m}{\left(\prod_{i=1}^{n}{\left(2-{f}_{{\hat{d}}_{ij}}^{q}\right)}^{\gamma {\theta }_{i}}\right)}^{{\lambda }_{j}}+\prod_{j=1}^{m}{\left({\prod }_{i=1}^{n}{\left({f}_{{\hat{d}}_{ij}}^{q}\right)}^{\gamma {\theta }_{i}}\right)}^{{\lambda }_{j}}}},\\ &\quad \frac{\sqrt[q]{\prod_{j=1}^{m}{\left({\prod }_{i=1}^{n}{\left(1+{g}_{{\hat{d}}_{ij}}^{q}\right)}^{\gamma {\theta }_{i}}\right)}^{{\omega }_{j}}-\prod_{j=1}^{m}{\left({\prod }_{i=1}^{n}{\left(1-{g}_{{\hat{d}}_{ij}}^{q}\right)}^{\gamma {\theta }_{i}}\right)}^{{\omega }_{j}}}}{\sqrt[q]{\prod_{j=1}^{m}{\left({\prod }_{i=1}^{n}{\left(1+{g}_{{\hat{d}}_{ij}}^{q}\right)}^{\gamma {\theta }_{i}}\right)}^{{\omega }_{j}}+ \prod_{j=1}^{m}{\left({\prod }_{i=1}^{n}{\left(1-{g}_{{\hat{d}}_{ij}}^{q}\right)}^{\gamma {\theta }_{i}}\right)}^{{\omega }_{j}}}}\Bigg\rangle \end{aligned}$$

$$\begin{aligned}&=\Bigg\langle \frac{\sqrt[q]{{\left(2\prod_{j=1}^{m}{\left({\prod }_{i=1}^{n}{\left({f}_{{\hat{d}}_{ij}}^{q}\right)}^{{\theta }_{i}}\right)}^{{\omega }_{j}}\right)}^{\gamma }}}{\sqrt[q]{\prod_{j=1}^{m}{\left(\prod_{i=1}^{n}{\left(2-{f}_{{\hat{d}}_{ij}}^{q}\right)}^{\gamma {\theta }_{i}}\right)}^{{\omega }_{j}}+\prod_{j=1}^{m}{\left({\prod }_{i=1}^{n}{\left({f}_{{\hat{d}}_{ij}}^{q}\right)}^{\gamma {\theta }_{i}}\right)}^{{\omega }_{j}}}},\\ &\quad \frac{\sqrt[q]{{\left(\prod_{j=1}^{m}{\left({\prod }_{i=1}^{n}{\left(1+{g}_{{\hat{d}}_{ij}}^{q}\right)}^{{\theta }_{i}}\right)}^{{\omega }_{j}}\right)}^{\gamma }-{\left(\prod_{j=1}^{m}{\left({\prod }_{i=1}^{n}{\left(1-{g}_{{\hat{d}}_{ij}}^{q}\right)}^{{\theta }_{i}}\right)}^{{\omega }_{j}}\right)}^{\gamma }}}{\sqrt[q]{{\left(\prod_{j=1}^{m}{\left({\prod }_{i=1}^{n}{\left(1+{g}_{{\hat{d}}_{ij}}^{q}\right)}^{{\theta }_{i}}\right)}^{{\omega }_{j}}\right)}^{\gamma }+ {\left(\prod_{j=1}^{m}{\left({\prod }_{i=1}^{n}{\left(1-{g}_{{\hat{d}}_{ij}}^{q}\right)}^{{\theta }_{i}}\right)}^{{\omega }_{j}}\right)}^{\gamma }}}\Bigg\rangle \end{aligned}$$

$$=\gamma q-ROFHSEWG \left({J}_{{\hat{d}}_{11}}, {J}_{{\hat{d}}_{12}}, \dots ,{J}_{{\hat{d}}_{nm}}\right).$$

#### Monotonicity

Let ${J}_{{\hat{d}}_{ij} }= \left({f}_{{\hat{d}}_{ij} }, {g}_{{\hat{d}}_{ij}}\right)$ and ${J}_{{\hat{d}}_{ij}}^{*}=\left({f}_{{\hat{d}}_{ij}}^{*}, {g}_{{\hat{d}}_{ij}}^{*}\right)$ be the collection of q-ROFHSNs. Then$q-ROFHSEWG\left({J}_{{\hat{d}}_{11}}, {J}_{{\hat{d}}_{12}}, \dots ,{J}_{{\hat{d}}_{nm}}\right)\le q-ROFHSEWG\left({J}_{{\hat{d}}_{11}}^{*}, {J}_{{\hat{d}}_{12}}^{*}, \dots ,{J}_{{\hat{d}}_{nm}}^{*}\right)$, if ${J}_{{\hat{d}}_{ij} }\le {J}_{{\hat{d}}_{ij}}^{*}$ $\forall i, j$.

##### Proof

Let $k\left(y\right)=\sqrt[q]{\frac{2-{y}^{q}}{{y}^{q}}}$, $y\in \left[0, 1\right]$, then $\frac{d}{dy}\left(k(y)\right)=-\frac{1}{q}{\left(\frac{2-{y}^{q}}{{y}^{q}}\right)}^{\frac{1}{q}-1}\left(\frac{2}{{\left({y}^{q}\right)}^{2}}\right)$. So, $\frac{d}{dy}\left(k(y)\right)=-\frac{1}{q}{\left(\frac{2-{y}^{q}}{{y}^{q}}\right)}^{\frac{1}{q}-1}\left(\frac{2}{{\left({y}^{q}\right)}^{2}}\right)<0$. So, $k(y)$ is decreasing on $\left[0, 1\right]$.

If ${f}_{{\hat{d}}_{ij}}^{*}\ge {f}_{{\hat{d}}_{ij}}$, then $k\left({f}_{{\hat{d}}_{ij}}^{*}\right)\ge k\left({f}_{{\hat{d}}_{ij}}\right)\forall i, j$. There are two possibilities

(i) $${f}_{{\hat{d}}_{ij}}^{*}\ge {f}_{{\hat{d}}_{ij} }\Rightarrow {f}_{{\hat{d}}_{ij}}^{q*}\ge {f}_{{\hat{d}}_{ij}}^{q}$$
  where ${\theta }_{i}>0$, ${\sum }_{i=1}^{n}{\theta }_{i}=1$ and ${\omega }_{j}>0$, ${\sum }_{j=1}^{m}{\omega }_{j}=1$. So,
  $${\left({\left(\left({f}_{{\hat{d}}_{ij}}^{q*}\right)\right)}^{{\sum }_{i=1}^{n}{\theta }_{i}}\right)}^{{\sum }_{j=1}^{m}{\omega }_{j}}\ge {\left({\left(\left({f}_{{\hat{d}}_{ij}}^{q}\right)\right)}^{{\sum }_{i=1}^{n}{\theta }_{i}}\right)}^{{\sum }_{j=1}^{m}{\omega }_{j}}$$
  28 $$\Rightarrow 2{\left({\left(\left({f}_{{\hat{d}}_{ij}}^{q*}\right)\right)}^{{\sum }_{i=1}^{n}{\theta }_{i}}\right)}^{{\sum }_{j=1}^{m}{\omega }_{j}}\ge 2{\left({\left(\left({f}_{{\hat{d}}_{ij}}^{q}\right)\right)}^{{\sum }_{i=1}^{n}{\theta }_{i}}\right)}^{{\sum }_{j=1}^{m}{\omega }_{j}}$$
(ii) $${f}_{{\hat{d}}_{ij}}^{q*}\ge {f}_{{\hat{d}}_{ij}}^{q}$$
  $$\Rightarrow 2-{f}_{{\hat{d}}_{ij}}^{q}\ge 2-{f}_{{\hat{d}}_{ij}}^{q*}$$
  $$\Rightarrow \left(2-{f}_{{\hat{d}}_{ij}}^{q}\right)+{f}_{{\hat{d}}_{ij}}^{q}\ge \left(2-{f}_{{\hat{d}}_{ij}}^{q*}\right)+{f}_{{\hat{d}}_{ij}}^{q*}$$
  29 $$\begin{aligned} & \Rightarrow {\left({\left(\left(2-{f}_{{\hat{d}}_{ij}}^{q*}\right)\right)}^{{\sum }_{i=1}^{n}{\theta }_{i}}\right)}^{{\sum }_{j=1}^{m}{\omega }_{j}}+{\left({\left(\left({f}_{{\hat{d}}_{ij}}^{q*}\right)\right)}^{{\sum }_{i=1}^{n}{\theta }_{i}}\right)}^{{\sum }_{j=1}^{m}{\omega }_{j}}\\ &\quad\le {\left({\left(\left(2-{f}_{{\hat{d}}_{ij}}^{q}\right)\right)}^{{\sum }_{i=1}^{n}{\theta }_{i}}\right)}^{{\sum }_{j=1}^{m}{\omega }_{j}}+{\left({\left(\left({f}_{{\hat{d}}_{ij}}^{q}\right)\right)}^{{\sum }_{i=1}^{n}{\theta }_{i}}\right)}^{{\sum }_{j=1}^{m}{\omega }_{j}}\end{aligned}$$
  From (28) and (29), we get
  $$\begin{aligned} & \Rightarrow \frac{\sqrt[q]{2\prod_{j=1}^{m}{\left({\prod_{i=1}^{n}\left({f}_{{\hat{d}}_{ij}}^{q*}\right)}^{{\theta }_{i}}\right)}^{{\omega }_{j}}}}{\sqrt[q]{\prod_{j=1}^{m}{\left(\prod_{i=1}^{n}{\left(2-{f}_{{\hat{d}}_{ij}}^{q*}\right)}^{{\theta }_{i}}\right)}^{{\omega }_{j}}+ \prod_{j=1}^{m}{\left({\prod_{i=1}^{n}\left({f}_{{\hat{d}}_{ij}}^{q*}\right)}^{{\theta }_{i}}\right)}^{{\omega }_{j}}}}\\ &\quad\le \frac{\sqrt[q]{2\prod_{j=1}^{m}{\left({\prod_{i=1}^{n}\left({f}_{{\hat{d}}_{ij}}^{q}\right)}^{{\theta }_{i}}\right)}^{{\omega }_{j}}}}{\sqrt[q]{\prod_{j=1}^{m}{\left(\prod_{i=1}^{n}{\left(2-{f}_{{\hat{d}}_{ij}}^{q}\right)}^{{\theta }_{i}}\right)}^{{\omega }_{j}}+ \prod_{j=1}^{m}{\left({\prod_{i=1}^{n}\left({f}_{{\hat{d}}_{ij}}^{q}\right)}^{{\theta }_{i}}\right)}^{{\omega }_{j}}}}\end{aligned}$$
  $$\Rightarrow \frac{\sqrt[q]{2\prod_{j=1}^{m}{\left({\prod_{i=1}^{n}\left({f}_{{\hat{d}}_{ij}}^{q*}\right)}^{{\theta }_{i}}\right)}^{{\omega }_{j}}}}{\sqrt[q]{\prod_{j=1}^{m}{\left(\prod_{i=1}^{n}{\left(2-{f}_{{\hat{d}}_{ij}}^{q*}\right)}^{{\theta }_{i}}\right)}^{{\omega }_{j}}+ \prod_{j=1}^{m}{\left({\prod_{i=1}^{n}\left({f}_{{\hat{d}}_{ij}}^{q*}\right)}^{{\theta }_{i}}\right)}^{{\omega }_{j}}}}\le \frac{\sqrt[q]{2\prod_{j=1}^{m}{\left({\prod_{i=1}^{n}\left({f}_{{\hat{d}}_{ij}}^{q}\right)}^{{\theta }_{i}}\right)}^{{\omega }_{j}}}}{\sqrt[q]{\prod_{j=1}^{m}{\left(\prod_{i=1}^{n}{\left(2-{f}_{{\hat{d}}_{ij}}^{q}\right)}^{{\theta }_{i}}\right)}^{{\omega }_{j}}+ \prod_{j=1}^{m}{\left({\prod_{i=1}^{n}\left({f}_{{\hat{d}}_{ij}}^{q}\right)}^{{\theta }_{i}}\right)}^{{\omega }_{j}}}}$$
  Let $h\left(x\right)=\sqrt[q]{\frac{2-{x}^{q}}{{x}^{q}}}$, $x\in \left[0, 1\right]$, then $\frac{d}{dx}\left(h(x)\right)=-\frac{1}{q}{\left(\frac{2-{x}^{q}}{{x}^{q}}\right)}^{\frac{1}{q}-1}\left(\frac{2}{{\left({x}^{q}\right)}^{2}}\right)$. So, $\frac{d}{dx}\left(k(x)\right)=-\frac{1}{q}{\left(\frac{2-{x}^{q}}{{x}^{q}}\right)}^{\frac{1}{q}-1}\left(\frac{2}{{\left({x}^{q}\right)}^{2}}\right)<0$. So, $h(x)$ is decreasing on $\left[0, 1\right]$. If ${g}_{{\hat{d}}_{ij} }\ge {g}_{{\hat{d}}_{ij}}^{*}$, then $h\left({g}_{{\hat{d}}_{ij}}^{*}\right)\le h\left({g}_{{\hat{d}}_{ij}}\right)\forall i, j$.
  $$1-{g}_{{\hat{d}}_{ij}}^{*}\le 1-{g}_{{\hat{d}}_{ij}}$$
  $$\Rightarrow 1-{g}_{{\hat{d}}_{ij}}^{q*}\le 1-{g}_{{\hat{d}}_{ij}}^{q}$$
  $$\Rightarrow \left(1+{g}_{{\hat{d}}_{ij}}^{q}\right)-\left(1-{g}_{{\hat{d}}_{ij}}^{q}\right)\le \left(1+{g}_{{\hat{d}}_{ij}}^{q*}\right)-\left(1-{g}_{{\hat{d}}_{ij}}^{q*}\right)$$
  $$\Rightarrow \frac{\left(1+{g}_{{\hat{d}}_{ij}}^{q}\right)-\left(1-{g}_{{\hat{d}}_{ij}}^{q}\right)}{\left(1+{g}_{{\hat{d}}_{ij}}^{q}\right)+\left(1-{g}_{{\hat{d}}_{ij}}^{q}\right)}\le \frac{\left(1+{g}_{{\hat{d}}_{ij}}^{q*}\right)-\left(1-{g}_{{\hat{d}}_{ij}}^{q*}\right)}{\left(1+{g}_{{\hat{d}}_{ij}}^{q*}\right)+\left(1-{g}_{{\hat{d}}_{ij}}^{q*}\right)}$$
  where, ${\theta }_{i}>0$, ${\sum }_{i=1}^{n}{\theta }_{i}=1$ and ${\omega }_{j}>0,$ ${\sum }_{j=1}^{m}{\omega }_{j}=1$. So,
  $$\Rightarrow \frac{\left(1+{g}_{{\hat{d}}_{ij}}^{q}\right)-\left(1-{g}_{{\hat{d}}_{ij}}^{q}\right)}{\left(1+{g}_{{\hat{d}}_{ij}}^{q}\right)+\left(1-{g}_{{\hat{d}}_{ij}}^{q}\right)}\le \frac{\left(1+{g}_{{\hat{d}}_{ij}}^{q*}\right)-\left(1-{g}_{{\hat{d}}_{ij}}^{q*}\right)}{\left(1+{g}_{{\hat{d}}_{ij}}^{q*}\right)+\left(1-{g}_{{\hat{d}}_{ij}}^{q*}\right)}$$
  $$\begin{aligned}& \Rightarrow \frac{{\left({\left(\left(1+{g}_{{\hat{d}}_{ij}}^{q}\right)\right)}^{{\sum }_{i=1}^{n}{\theta }_{i}}\right)}^{{\sum }_{j=1}^{m}{\omega }_{j}}-{\left({\left(\left(1-{g}_{{\hat{d}}_{ij}}^{q}\right)\right)}^{{\sum }_{i=1}^{n}{\theta }_{i}}\right)}^{{\sum }_{j=1}^{m}{\omega }_{j}}}{{\left({\left(\left(1+{g}_{{\hat{d}}_{ij}}^{q}\right)\right)}^{{\sum }_{i=1}^{n}{\theta }_{i}}\right)}^{{\sum }_{j=1}^{m}{\omega }_{j}}+{\left({\left(\left(1-{g}_{{\hat{d}}_{ij}}^{q}\right)\right)}^{{\sum }_{i=1}^{n}{\theta }_{i}}\right)}^{{\sum }_{j=1}^{m}{\omega }_{j}}}\\ &\quad\le \frac{{\left({\left(\left(1+{g}_{{\hat{d}}_{ij}}^{q*}\right)\right)}^{{\sum }_{i=1}^{n}{\theta }_{i}}\right)}^{{\sum }_{j=1}^{m}{\omega }_{j}}-{\left({\left(\left(1-{g}_{{\hat{d}}_{ij}}^{q*}\right)\right)}^{{\sum }_{i=1}^{n}{\theta }_{i}}\right)}^{{\sum }_{j=1}^{m}{\omega }_{j}}}{{\left({\left(\left(1+{g}_{{\hat{d}}_{ij}}^{q*}\right)\right)}^{{\sum }_{i=1}^{n}{\theta }_{i}}\right)}^{{\sum }_{j=1}^{m}{\omega }_{j}}+{\left({\left(\left(1-{g}_{{\hat{d}}_{ij}}^{q*}\right)\right)}^{{\sum }_{i=1}^{n}{\theta }_{i}}\right)}^{{\sum }_{j=1}^{m}{\omega }_{j}}}\end{aligned}$$
  $$\begin{aligned}& \Rightarrow \frac{{\prod }_{j=1}^{m}{\left({\prod }_{i=1}^{n}{\left(1+{g}_{{\hat{d}}_{ij}}^{q}\right)}^{{\theta }_{i}}\right)}^{{\omega }_{j}}-{\prod }_{j=1}^{m}{\left({\prod }_{i=1}^{n}{\left(1-{g}_{{\hat{d}}_{ij}}^{q}\right)}^{{\theta }_{i}}\right)}^{{\omega }_{j}}}{{\prod }_{j=1}^{m}{\left({\prod }_{i=1}^{n}{\left(1+{g}_{{\hat{d}}_{ij}}^{q}\right)}^{{\theta }_{i}}\right)}^{{\omega }_{j}}+{\prod }_{j=1}^{m}{\left({\prod }_{i=1}^{n}{\left(1-{g}_{{\hat{d}}_{ij}}^{q}\right)}^{{\theta }_{i}}\right)}^{{\omega }_{j}}}\\ &\quad\le \frac{{\prod }_{j=1}^{m}{\left({\prod }_{i=1}^{n}{\left(1+{g}_{{\hat{d}}_{ij}}^{q*}\right)}^{{\theta }_{i}}\right)}^{{\omega }_{j}}-{\prod }_{j=1}^{m}{\left({\prod }_{i=1}^{n}{\left(1-{g}_{{\hat{d}}_{ij}}^{q*}\right)}^{{\theta }_{i}}\right)}^{{\omega }_{j}}}{{\prod }_{j=1}^{m}{\left({\prod }_{i=1}^{n}{\left(1+{g}_{{\hat{d}}_{ij}}^{q*}\right)}^{{\theta }_{i}}\right)}^{{\omega }_{j}}+{\prod }_{j=1}^{m}{\left({\prod }_{i=1}^{n}{\left(1-{g}_{{\hat{d}}_{ij}}^{q*}\right)}^{{\theta }_{i}}\right)}^{{\omega }_{j}}}\end{aligned}$$
  $$\begin{aligned} & \Rightarrow \frac{\sqrt[q]{{\prod }_{j=1}^{m}{\left({\prod }_{i=1}^{n}{\left(1+{g}_{{\hat{d}}_{ij}}^{q}\right)}^{{\theta }_{i}}\right)}^{{\omega }_{j}}-{\prod }_{j=1}^{m}{\left({\prod }_{i=1}^{n}{\left(1-{g}_{{\hat{d}}_{ij}}^{q}\right)}^{{\theta }_{i}}\right)}^{{\omega }_{j}}}}{\sqrt[q]{{\prod }_{j=1}^{m}{\left({\prod }_{i=1}^{n}{\left(1+{g}_{{\hat{d}}_{ij}}^{q}\right)}^{{\theta }_{i}}\right)}^{{\omega }_{j}}+{\prod }_{j=1}^{m}{\left({\prod }_{i=1}^{n}{\left(1-{g}_{{\hat{d}}_{ij}}^{q}\right)}^{{\theta }_{i}}\right)}^{{\omega }_{j}}}}\\ &\quad\le \frac{\sqrt[q]{{\prod }_{j=1}^{m}{\left({\prod }_{i=1}^{n}{\left(1+{g}_{{\hat{d}}_{ij}}^{q*}\right)}^{{\theta }_{i}}\right)}^{{\omega }_{j}}-{\prod }_{j=1}^{m}{\left({\prod }_{i=1}^{n}{\left(1-{g}_{{\hat{d}}_{ij}}^{q*}\right)}^{{\theta }_{i}}\right)}^{{\omega }_{j}}}}{\sqrt[q]{{\prod }_{j=1}^{m}{\left({\prod }_{i=1}^{n}{\left(1+{g}_{{\hat{d}}_{ij}}^{q*}\right)}^{{\theta }_{i}}\right)}^{{\omega }_{j}}+{\prod }_{j=1}^{m}{\left({\prod }_{i=1}^{n}{\left(1-{g}_{{\hat{d}}_{ij}}^{q*}\right)}^{{\theta }_{i}}\right)}^{{\omega }_{j}}}}\end{aligned}$$
  So, it proved that
  $$q-ROFHSEWG\left({J}_{{\hat{d}}_{11}}, {J}_{{\hat{d}}_{12}}, \dots ,{J}_{{\hat{d}}_{nm}}\right)\le q-ROFHSEWG\left({J}_{{\hat{d}}_{11}}^{*}, {J}_{{\hat{d}}_{12}}^{*}, \dots ,{J}_{{\hat{d}}_{nm}}^{*}\right).$$

## MCGDM model under q-ROFHSS information

To substantiate the inference of the established Einstein-weighted AOs, there is a DM method to eradicate MCGDM constraints. Also, we used the developed approach to select the most appropriate construction company.

### Proposed MCGDM approach

Let $\mathrm{\aleph }=\left\{{\mathrm{\aleph }}^{1}, {\mathrm{\aleph }}^{2}, {\mathrm{\aleph }}^{3},\dots , {\mathrm{\aleph }}^{s}\right\}$ and $\mathcal{H}=\left\{{\mathcal{H}}_{1}, {\mathcal{H}}_{2}, {\mathcal{H}}_{3},\dots , {\mathcal{H}}_{n}\right\}$ be the collection of alternatives and a group of experts with weights of experts $\theta ={\left({\theta }_{1}, {\theta }_{2},\dots , {\theta }_{n}\right)}^{T}$ such as ${\theta }_{i}>0$, $\sum_{i=1}^{n}{\theta }_{i}=1$. Suppose $\mathfrak{L}=\left\{{d}_{1}, {d}_{2},\dots , {d}_{m}\right\}$ shows the set of parameters and ${\mathfrak{L}}^{{^{\prime}}}=\left\{\left({d}_{1\rho }\times {d}_{2\rho }\times \dots \times {d}_{m\rho }\right) for all \rho \in \left\{1, 2,\dots , t\right\}\right\}$ be a collection of multi sub-attributes with weights $\omega ={\left({\omega }_{1}, {\omega }_{2}, {\omega }_{3},\dots , {\omega }_{m}\right)}^{T}$ such as ${\omega }_{j}>0$, $\sum_{j=1}^{m}{\omega }_{j}=1$. The collection of sub-attributes can be designated as ${\mathfrak{L}}^{{^{\prime}}}=\left\{{\hat{d}}_{\partial }:\partial \in \left\{1, 2, \dots ,m\right\}\right\}$. The group of experts $\left\{{\mathcal{H}}_{i}: i = 1, 2,\dots , n\right\}$ evaluate the alternates $\left\{{\mathrm{\aleph }}^{(z)}: z = 1, 2, \dots , s\right\}$ in the form of q-ROFHSNs beneath the chosen sub-parameters $\left\{{\hat{d}}_{\partial }: \partial = 1, 2, \dots , m\right\}$ such as ${\left({J}_{{\hat{d}}_{ij}}^{(z)}\right)}_{n\times m}={\left({f}_{{\hat{d}}_{ij}}, {g}_{{\hat{d}}_{ij}}\right)}_{n\times m}$, where $0\le {f}_{{\hat{d}}_{ij}}, {g}_{{\hat{d}}_{ij}}\le 1$ and $0\le {\left({f}_{{\hat{d}}_{ij}}\right)}^{q}+{\left({g}_{{\hat{d}}_{ij}}\right)}^{q}\le 1 \forall i,j$. The group of experts conveys the verdict in q-ROFHSNs form for each alternate. A novel algorithm has been developed under q-ROFHSS settings to compute the appropriate alternative.

*Step 1* Compute the decision matrices for each alternate in terms of q-ROFHSNs $\left({\mathrm{\aleph }}^{(z)}, {\mathfrak{L}}^{{^{\prime}}}\right)={\left({f}_{{\hat{d}}_{ij}}, {g}_{{\hat{d}}_{ij}}\right)}_{n\times m}$.

$${\left({\mathrm{\aleph }}^{(z)}, {\mathfrak{L}}^{{^{\prime}}}\right)}_{n\times \partial }=\begin{array}{c}{\mathcal{H}}_{1}\\ {\mathcal{H}}_{2}\\ \vdots \\ {\mathcal{H}}_{n}\end{array}\left(\begin{array}{cccc}\left({f}_{{\hat{d}}_{11}}^{(z)}, {g}_{{\hat{d}}_{11}}^{(z)}\right)& \left({f}_{{\hat{d}}_{12}}^{(z)}, {g}_{{\hat{d}}_{12}}^{(z)}\right)& \cdots & \left({f}_{{\hat{d}}_{1\partial }}^{(z)}, {g}_{{\hat{d}}_{1\partial }}^{(z)}\right)\\ \left({f}_{{\hat{d}}_{21}}^{(z)}, {g}_{{\hat{d}}_{21}}^{(z)}\right)& \left({f}_{{\hat{d}}_{22}}^{(z)}, {g}_{{\hat{d}}_{22}}^{(z)}\right)& \cdots & \left({f}_{{\hat{d}}_{2\partial }}^{(z)}, {g}_{{\hat{d}}_{2\partial }}^{(z)}\right)\\ \vdots & \vdots & \vdots & \vdots \\ \left({f}_{{\hat{d}}_{n1}}^{(z)}, {g}_{{\hat{d}}_{n1}}^{(z)}\right)& \left({f}_{{\hat{d}}_{n2}}^{(z)}, {g}_{{\hat{d}}_{n2}}^{(z)}\right)& \cdots & \left({f}_{{\hat{d}}_{n\partial }}^{(z)}, {g}_{{\hat{d}}_{n\partial }}^{(z)}\right)\end{array}\right)$$

*Step 2* Converts the cost type aspects into benefit types using the normalization rule.

$${\left({\mathrm{\aleph }}^{(z)}, {\mathfrak{L}}^{{^{\prime}}}\right)}_{n\times \partial }=\left\{\begin{array}{c}{J}_{{\hat{d}}_{ij}}^{c}; cost type parameter\\ {J}_{{\hat{d}}_{ij}}; benefit type parameter\end{array}\right.$$

*Step 3* Settled Einstein weighted AOs, compute the collective decision matrix ${\mathcal{L}}_{k}$.

*Step 4* For ranking alternatives, find the score values using the score function.

*Step 5* Analyze the aptest construction industry based on the maximum score value ${\mathcal{L}}_{k}$.

*Step 6* Compute the ordering of the substitutes.

**Figure 1:**
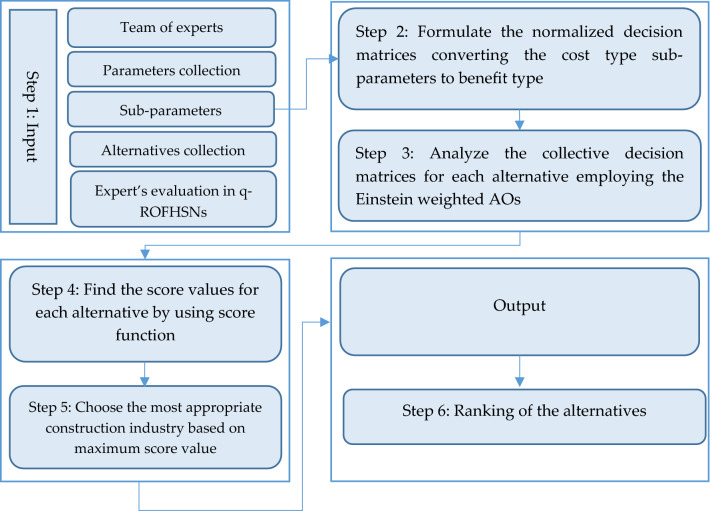
Graphical demonstration of the proposed model.

The graphical presentation of our developed MCGDM technique is given in the following Fig. 1.

### Application of proposed MCGDM model

Making appropriate decisions in the construction industry requires a thorough understanding of the project's goals, requirements, constraints, and risks. To make informed decisions, construction professionals typically use a process called construction decision-making. Construction decision-making involves the process of identifying and analyzing various options to choose the best alternative that meets project requirements and objectives. It involves a systematic approach of evaluating options, considering risks and benefits, and selecting the most appropriate course of action. Effective construction decision-making requires a clear understanding of project goals, available resources, budget constraints, and potential risks. It also involves the collaboration of various stakeholders, including architects, engineers, contractors, and project managers, to ensure that decisions are made in the best interest of the project. The significant decision-makers in the construction business must be understood to exploit deal realization. Deprived of this understanding, it can be challenging to distinguish who you would target and at what step of the manufacturing procedure to describe and use your goods. To comfort you understand who's who, we've formed an outline for each superior construction and manufacturing engineering expert. So let us tell your spectators. The uniqueness of the construction industry poses substantial contests, parting the building engineering overdue other businesses, such as the motorized business or aeronautics. While it is extensively assumed that the strategy stage of a task has the most substantial influence on construction life cycle consequences, severe scheme assessment methods are still lacking in the construction industry. This problem is exacerbated when a building needs to be more sustainable through its life cycle, as it involves the intention to be estimated alongside multiple criteria, namely, societal, environmentally friendly, and monetary. Building administration and expertise are the two core features manipulating the enlargement of construction engineering. For 40 years, the industry has been ineffective despite some new and advanced technologies being applied to construction projects^61^. The investigators believed that digital technology projects could enable faster and more flexible forms of organization^62^. The construction scheme organization procedure institutes realistic goals to achieve user needs, project constraints, resource requirements, and premeditated objectives. Iterative practice as new facts develops presented through the energies of several experts elaborate on the task. Mobile hardware, cloud computing, and incorporated software are used for storing and reclamation, robotic exploration, and prototyping and model capabilities.

Project management aims to implement the project so that deliverables meet economic and agenda capability necessities and are at conventional stages of risk, feature, protection, and sanctuary^63^. One of the construction's most significant chores is selecting the factual contractor. Selecting the right contractor from the crowd of contenders in today's market is a multifarious problem for clientele. Accomplishing this objective is fundamentally influenced by the enactment of designated contractors^64^. The study of contractor assortment epochs back to the 1960s. Because of their classical contributions, the most frequently mentioned papers of the period are rarely those of Busch, Dickson, Hakansson, and Woots, as well as Dempsey. These studies have proven the significance of product eminence and provision in supplier selection^65–67^. All building progressions are hazardous.

Contract threat administration solitary forms part of the corporation's permitted risk organization, and as such, it is a measure of the corporation's inclusive overall risk administration. The purpose of contract risk management does not limit legal risk in contracts. Contract risk organizations also conceal other corporate hazards through contract scheduling and administration techniques^68,69^. Further complications in construction organizations in emerging states were acknowledged. Mohamed^70^ describes the most significant construction managing structure problem: all construction organization complications must be addressed after identifying them. Senaratne and Sexton^71^ accentuate that organizational theory has implemented problem resolving as an information dispensation bustle in the information age. But, in this epoch, co-solutions are gradually acknowledged as a substance for awareness construction with the consciousness of knowledge-based approaches in administrations. In the collective problem-solving method, participants carry multiple pieces of information into delinquent states apprehended, fashioned, and mutual by group affiliates. In construction developments, joint solutions are often achieved through practical problem-solving on-site, especially by supervising project modification. The core parameters for the assortment of the construction corporation are specified as follows:

Quality Assurance: Through regular construction work, servicers occasionally combine the idea of quality assurance (QA) with quality control (QC). Meanwhile, the two respond to each other; it appears ordinary to classify them into one procedure. However, mystifying the two is a mistake. If vendors and clients appropriately appliance the peculiarity among QA for construction schemes and QC for construction projects, it will be tough for construction corporations to certify significant consequences through creation. These two dispersed constituents of feature administration are not substitutable. Each assists an explicit set of methods, intentions, and targets. To meet excellently appliance-proven construction scheme superiority criteria, your field workers, machinists, administrators, and assistants must exert character in the feature package edifice. A fruitful QA package is intended to confirm that the superiority processes instigated through the enterprise stage of the construction project successfully encounter the corporation's proven quality facility, productivity, and fabrication principles. The main specifications focus on conventional recommendations for builders, originators, engineers, and profit margin documentation associated with projects and portrayals, from groundwork to modification to absolute agreement. QA describes a scheme for influencing how and when construction criteria are indomitable; although the superiority governor confirms that the arena operates, operatives and executives react decorously to explicit QA principles. QA explains a technique that qualifies your group for periodical and concludes construction criteria that meet the following standards:

Experience in Construction: Construction is the sentiment of a state's budget. It's not just approximately construction communities – it's observing infrastructures, substructures, and other schemes that associate the nation with each other. An occupation in this manufacturing not only resources employed in the arena all the time but also numerous scenarios to move up the stepladder and exertion in characters absorbed on more organization. You will absorb around particular of the significant parts and tasks of aspirants in the primary steps of their occupation in construction engineering. Not only that, but you'll absorb more about onboarding desires and career paths while equipping you with the assistance and perceptions required to grow your head on your career.

Cost criteria: The material can provide interpretation for 70% of the project's construction cost! Consumption guesstimate software to precisely guess material convention, project material tolerance, and material waste aspects. Correct cost assessment is significant in construction developments as mistakes can lead to substantial financial plan concerns and prospective fatalities for any plan. By apparent contravention depressed the material prerequisite for a task, location administration can take control to accomplish material use. It is a virtuous notion to use guesstimate software for construction corporations that famine to deliver precise cost assessments to condense discarded. This will not only mark the procedure calmer and extra rationalized, but it will also produce more perfect outcomes. Suitably assessing the budget is one of the crucial aspects of a project's accomplishment. So, here's what you want to distinguish between certifying and correct cost assessment development. Construction cost assessment is the procedure of forecasting the total cost of a new construction plan. Actual intelligence to precisely predict is an imperative zone of the project. Formerly scheduled with the project, you want to guesstimate all costs and elaborate correctly. This will comfortably regulate the latitude of the project, the obligatory properties, the time structure, and, of course, the reasonable. Precise estimations support constructors to confirm that they assemble the correct content and incarnate on a budget. In most circumstances, construction software will be openly connected to the acquisitions compendium. This association certifies that the contractor purchases the substantial at the acceptable amount and in the apportioned capacity. Any deviations from cost estimations are directly flagged to govern probable fatalities or obtaining profits.

### Numerical description

The current research discusses the MCGDM approach for making construction decisions, which involves considering multiple, often conflicting criteria. These criteria can have different characteristics and weights, some of which can be mathematically defined while others require intuitive definitions. Various approaches can be used to solve MCGDM problems, and this methodology can help address several administrative challenges. The main goal of the research is to use Einstein-weighted AOs in the q-ROFHSS environment to select the most appropriate construction company for a project. Let {$${\mathrm{\aleph }}^{(1)}$$, $${\mathrm{\aleph }}^{(2)}$$, $${\mathrm{\aleph }}^{(3)}$$} be a set of alternatives that represents some construction companies. Let {$${\mathcal{H}}_{1}$$, $${\mathcal{H}}_{2}$$, $${\mathcal{H}}_{3}$$} be a team of experts with weights $${\theta }_{i}={\left( 0.3, 0.3, 0.4\right)}^{T}$$. initially, the team of experts short-listed three construction companies such as; $${\mathrm{\aleph }}^{(1)}$$: Hefei furong construction labor service, $${\mathrm{\aleph }}^{(2)}$$: Anhui construction engineering Hefei building materials, $${\mathrm{\aleph }}^{(3)}$$: Hefei new port construction investment company. The team of experts considered the set of parameters for the selection of the most appropriate construction companies given as  = $$\left\{{d}_{1}= \{quality assurance, {d}_{2}=experience in construction, {d}_{3}=cost criteria\right\}$$. The multi sub-attributes of the considered parameters are given as $$quality assurance$$ = $${d}_{1}$$  = $$\left\{{d}_{11}=70\mathrm{\%}, {d}_{12}=80\mathrm{\%}\right\}$$, $$experience in construction$$ = $${d}_{2}$$  = $$\left\{{d}_{21}=more than 10 years, {d}_{22}=less than 10 years\right\}$$, $$cost criteria$$ = $${d}_{3}$$  = $$\left\{{d}_{31}=20\mathrm{ \% }management fee of project cost, {d}_{32}=25\mathrm{ \% }management fee of project cost\right\}$$. Let $${\mathfrak{L}}^{{^{\prime}}}={d}_{1}\times {d}_{2}\times {d}_{3}$$ shows the 3-tuple sub-attributes

$${\mathfrak{L}}^{{^{\prime}}}={d}_{1}\times {d}_{2}\times {d}_{3}=\left\{{d}_{11}, {d}_{12}\right\}\times \left\{{d}_{21}, {d}_{22}\right\}\times \left\{{d}_{31}, {d}_{32}\right\}$$

$$=\left\{\begin{array}{c}\left({\mathrm{d}}_{11}, {\mathrm{d}}_{21}, {\mathrm{d}}_{31}\right), \left({\mathrm{d}}_{11}, {\mathrm{d}}_{21}, {\mathrm{d}}_{32}\right), \left({\mathrm{d}}_{11}, {\mathrm{d}}_{22}, {\mathrm{d}}_{31}\right), \left({\mathrm{d}}_{11}, {\mathrm{d}}_{22}, {\mathrm{d}}_{32}\right), \\ \left({\mathrm{d}}_{12}, {\mathrm{d}}_{21}, {\mathrm{d}}_{31}\right), \left({\mathrm{d}}_{12}, {\mathrm{d}}_{21}, {\mathrm{d}}_{32}\right), \left({\mathrm{d}}_{12}, {\mathrm{d}}_{22}, {\mathrm{d}}_{31}\right), \left({\mathrm{d}}_{12}, {\mathrm{d}}_{22}, {\mathrm{d}}_{32}\right)\end{array}\right\}$$, ${\mathfrak{L}}^{{^{\prime}}}=\left\{{\hat{\mathrm{d}}}_{1},{\hat{\mathrm{d}}}_{2}, {\hat{\mathrm{d}}}_{3}, {\hat{\mathrm{d}}}_{4}, {\hat{\mathrm{d}}}_{5}, {\hat{\mathrm{d}}}_{6}, {\hat{\mathrm{d}}}_{7}, {\hat{\mathrm{d}}}_{8}\right\}$ with weights ${\upomega }_{\mathrm{j}}={\left(0.1, 0.22, 0.05, 0.15, 0.08, 0.1, 0.18, 0.12\right)}^{\mathrm{T}}$. Experts provide their partialities in q-ROFHSNs form. The numerical data is taken from^58^.

### By using the q-ROFHSEWA operator

Step 1. Compute the decision matrices for each alternate in terms of q-ROFHSNs, and their predilections are given in Tables 1, 2, 3.

**Table 1:** Decision matrix for ${\mathbf{\aleph }}_{1}$.

	${\hat{d}}_{1}$	${\hat{d}}_{2}$	${\hat{d}}_{3}$	${\hat{d}}_{4}$	${\hat{d}}_{5}$	${\hat{d}}_{6}$	${\hat{d}}_{7}$	${\hat{d}}_{8}$
${\mathcal{H}}_{1}$	$\left(0.5, 0.4\right)$	$\left(0.4, 0.6\right)$	$\left(0.5, 0.7\right)$	$\left(0.2, 0.9\right)$	$\left(0.7, 0.8\right)$	$\left(0.4, 0.5\right)$	$\left(0.2, 0.6\right)$	$\left(0.1, 0.7\right)$
${\mathcal{H}}_{2}$	$\left(0.3, 0.6\right)$	$\left(0.1, 0.4\right)$	$\left(0.2, 0.4\right)$	$\left(0.5, 0.3\right)$	$\left(0.6, 0.4\right)$	$\left(0.5, 0.8\right)$	$\left(0.3, 0.2\right)$	$\left(0.9, 0.2\right)$
${\mathcal{H}}_{3}$	$\left(0.7, 0.8\right)$	$\left(0.8, 0.5\right)$	$\left(0.4, 0.6\right)$	$\left(0.7, 0.2\right)$	$\left(0.6, 0.1\right)$	$\left(0.4, 0.5\right)$	$\left(0.3, 0.2\right)$	$\left(0.9, 0.2\right)$

**Table 2:** Decision matrix for ${\mathbf{\aleph }}_{2}$

	${\hat{d}}_{1}$	${\hat{d}}_{2}$	${\hat{d}}_{3}$	${\hat{d}}_{4}$	${\hat{d}}_{5}$	${\hat{d}}_{6}$	${\hat{d}}_{7}$	${\hat{d}}_{8}$
${\mathcal{H}}_{1}$	$\left(0.\mathrm{3,0.7}\right)$	$\left(\mathrm{0.1,0.4}\right)$	$\left(\mathrm{0.9,0.3}\right)$	$\left(\mathrm{0.5,0.2}\right)$	$\left(\mathrm{0.2,0.1}\right)$	$\left(\mathrm{0.4,0.6}\right)$	$\left(\mathrm{0.3,0.8}\right)$	$\left(\mathrm{0.5,0.1}\right)$
${\mathcal{H}}_{2}$	$\left(0.\mathrm{3,0.8}\right)$	$\left(0.\mathrm{4,0.3}\right)$	$\left(0.\mathrm{5,0.7}\right)$	$\left(0.\mathrm{6,0.8}\right)$	$\left(0.\mathrm{7,0.9}\right)$	$\left(0.\mathrm{7,0.2}\right)$	$\left(0.\mathrm{5,0.3}\right)$	$\left(0.\mathrm{8,0.9}\right)$
${\mathcal{H}}_{3}$	$\left(0.\mathrm{7,0.2}\right)$	$\left(0.\mathrm{8,0.1}\right)$	$\left(0.\mathrm{5,0.6}\right)$	$\left(0.\mathrm{4,0.9}\right)$	$\left(0.\mathrm{3,0.6}\right)$	$\left(0.\mathrm{2,0.4}\right)$	$\left(0.\mathrm{5,0.2}\right)$	$\left(0.\mathrm{1,0.4}\right)$

**Table 3:** Decision matrix for ${\mathbf{\aleph }}_{3}$.

	${\hat{d}}_{1}$	${\hat{d}}_{2}$	${\hat{d}}_{3}$	${\hat{d}}_{4}$	${\hat{d}}_{5}$	${\hat{d}}_{6}$	${\hat{d}}_{7}$	${\hat{d}}_{8}$
${\mathcal{H}}_{1}$	$\left(0.\mathrm{5,0.4}\right)$	$\left(0.\mathrm{6,0.3}\right)$	$\left(\mathrm{0.7,0.2}\right)$	$\left(\mathrm{0.1,0.4}\right)$	$\left(\mathrm{0.8,0.5}\right)$	$\left(0.\mathrm{9,0.4}\right)$	$\left(\mathrm{0.1,0.5}\right)$	$\left(\mathrm{0.3,0.7}\right)$
${\mathcal{H}}_{2}$	$\left(0.\mathrm{1,0.7}\right)$	$\left(0.\mathrm{4,0.9}\right)$	$\left(0.\mathrm{5,0.6}\right)$	$\left(0.\mathrm{2,0.9}\right)$	$\left(0.\mathrm{6,0.7}\right)$	$\left(0.\mathrm{4,0.3}\right)$	$\left(0.\mathrm{8,0.2}\right)$	$\left(0.\mathrm{4,0.6}\right)$
${\mathcal{H}}_{3}$	$\left(0.\mathrm{3,0.5}\right)$	$\left(0.\mathrm{2,0.4}\right)$	$\left(0.\mathrm{1,0.8}\right)$	$\left(0.\mathrm{7,0.8}\right)$	$\left(0.\mathrm{5,0.9}\right)$	$\left(0.\mathrm{8,0.1}\right)$	$\left(0.\mathrm{2,0.9}\right)$	$\left(0.\mathrm{7,0.3}\right)$

Step 2. No need to normalize.

Step 3. Determine the collective aggregated values of alternatives from Tables 1, 2, 3 using the q-ROFHSEWA operator given as: ${\mathcal{L}}_{1}$= $\langle 0.5154, 0.4485\rangle$, ${\mathcal{L}}_{2}$= $\langle 0.5583, 0.4754\rangle$, ${\mathcal{L}}_{3}$= $\langle 0.6136, 0.3749\rangle$.

Step 4. Find the score values such as $S({\mathcal{L}}_{1})$ = 0.0574, $S({\mathcal{L}}_{2})$ = 0.0815, $S({\mathcal{L}}_{3})$ = 0.2181.

Step 5. ${\mathrm{\aleph }}^{3}$ is the best construction company because of the maximum score value.

**Figure 2:**
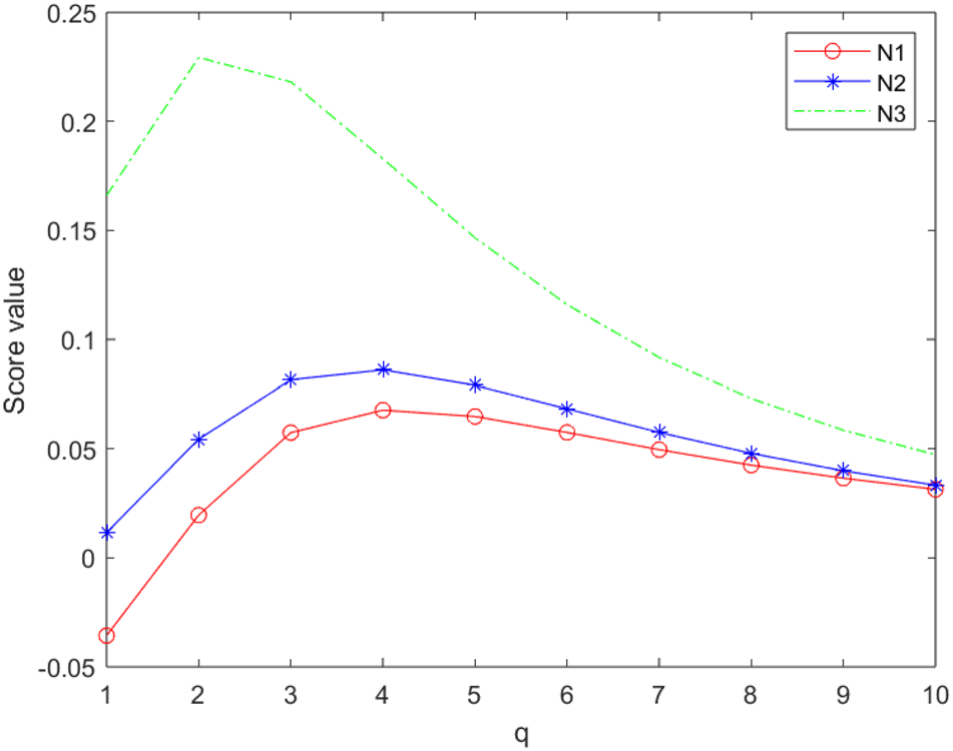
score values of the alternatives for $1\le q\le 10$ under Q-Rofhsewa.

Step 6. Investigate the ordering of the substitutes:

$S({\mathcal{L}}_{3})>S({\mathcal{L}}_{2})>S({\mathcal{L}}_{1})$. So, ${\mathrm{\aleph }}^{(3)}>{\mathrm{\aleph }}^{(2)}>{\mathrm{\aleph }}^{(1)}$. It is perceived that ${\mathrm{\aleph }}^{(3)}$ is the most applicable construction company. The influence of $q$ on assessment consequences for the q-ROFHSEWA operator is specified in Table 4. Also, the graphical demonstration of the influence of $q$ displayed in Fig. 2.

**Table 4:** Effects of parameter ${\varvec{q}}$ on decision results using Q-ROFHSEWA operator.

Parameter	Score value	Ranking
$q=1$	$S({\mathcal{L}}_{1})$= $-$ 0.0357, $S({\mathcal{L}}_{2})$ = 0.0116, $S({\mathcal{L}}_{3})$ = 0.1660	${\mathrm{\aleph }}^{(3)}>{\mathrm{\aleph }}^{(2)}>{\mathrm{\aleph }}^{(1)}$
$q=2$	$S({\mathcal{L}}_{1})$= 0.0196, $S({\mathcal{L}}_{2})$ = 0.0544, $S({\mathcal{L}}_{3})$ = 0.2293	${\mathrm{\aleph }}^{(3)}>{\mathrm{\aleph }}^{(2)}>{\mathrm{\aleph }}^{(1)}$
$q=3$	$S({\mathcal{L}}_{1})$= 0.0574, $S({\mathcal{L}}_{2})$ = 0.0815, $S({\mathcal{L}}_{3})$ = 0.2181	${\mathrm{\aleph }}^{(3)}>{\mathrm{\aleph }}^{(2)}>{\mathrm{\aleph }}^{(1)}$
$q=4$	$S({\mathcal{L}}_{1})$= 0.0677, $S({\mathcal{L}}_{2})$ = 0.0860, $S({\mathcal{L}}_{3})$ = 0.1828	${\mathrm{\aleph }}^{(3)}>{\mathrm{\aleph }}^{(2)}>{\mathrm{\aleph }}^{(1)}$
$q=5$	$S({\mathcal{L}}_{1})$= 0.0648, $S({\mathcal{L}}_{2})$ = 0.0790, $S({\mathcal{L}}_{3})$ = 0.1466	${\mathrm{\aleph }}^{(3)}>{\mathrm{\aleph }}^{(2)}>{\mathrm{\aleph }}^{(1)}$
$q=6$	$S({\mathcal{L}}_{1})$= 0.0575, $S({\mathcal{L}}_{2})$ = 0.0683, $S({\mathcal{L}}_{3})$ = 0.1159	${\mathrm{\aleph }}^{(3)}>{\mathrm{\aleph }}^{(2)}>{\mathrm{\aleph }}^{(1)}$
$q=7$	$S({\mathcal{L}}_{1})$= 0.0496, $S({\mathcal{L}}_{2})$ = 0.0575, $S({\mathcal{L}}_{3})$ = 0.0917	${\mathrm{\aleph }}^{(3)}>{\mathrm{\aleph }}^{(2)}>{\mathrm{\aleph }}^{(1)}$
$q=8$	$S({\mathcal{L}}_{1})$= 0.0425, $S({\mathcal{L}}_{2})$ = 0.0479, $S({\mathcal{L}}_{3})$ = 0.0730	${\mathrm{\aleph }}^{(3)}>{\mathrm{\aleph }}^{(2)}>{\mathrm{\aleph }}^{(1)}$
$q=9$	$S({\mathcal{L}}_{1})$= 0.0365, $S({\mathcal{L}}_{2})$ = 0.0399, $S({\mathcal{L}}_{3})$ = 0.0585	${\mathrm{\aleph }}^{(3)}>{\mathrm{\aleph }}^{(2)}>{\mathrm{\aleph }}^{(1)}$
$q=10$	$S({\mathcal{L}}_{1})$= 0.0314, $S({\mathcal{L}}_{2})$ = 0.0333, $S({\mathcal{L}}_{3})$ = 0.0473	${\mathrm{\aleph }}^{(3)}>{\mathrm{\aleph }}^{(2)}>{\mathrm{\aleph }}^{(1)}$

### By using the q-ROFHSEWG operator

Step 1 and 2 are similar to (C).

Step 3. Determine the collective accumulated values of alternatives from Tables 1, 2, 3 using the q-ROFHSEWG operator given as: ${\mathcal{L}}_{1}=\langle 0.3069, 0.5824\rangle$, ${\mathcal{L}}_{2}=\langle 0.3998, 0.6754\rangle$, ${\mathcal{L}}_{3}=\langle 0.4347, 0.6194\rangle$.

Step 4. Find the score values such as $S\left({\mathcal{L}}_{1}\right)=-$ 0.2072, $S\left({\mathcal{L}}_{2}\right)=-$ 0.2963, $S\left({\mathcal{L}}_{3}\right)=-$ 0.1896.

Step 5. ${\mathrm{\aleph }}^{3}$ is the best construction company because of the maximum score value.

Step 6. Investigate the ordering of the substitutes.

$$S({\mathcal{L}}_{3})>S({\mathcal{L}}_{1})>S({\mathcal{L}}_{2})$$. So, $${\mathrm{\aleph }}^{(3)}>{\mathrm{\aleph }}^{(1)}>{\mathrm{\aleph }}^{(2)}$$.

**Figure 3:**
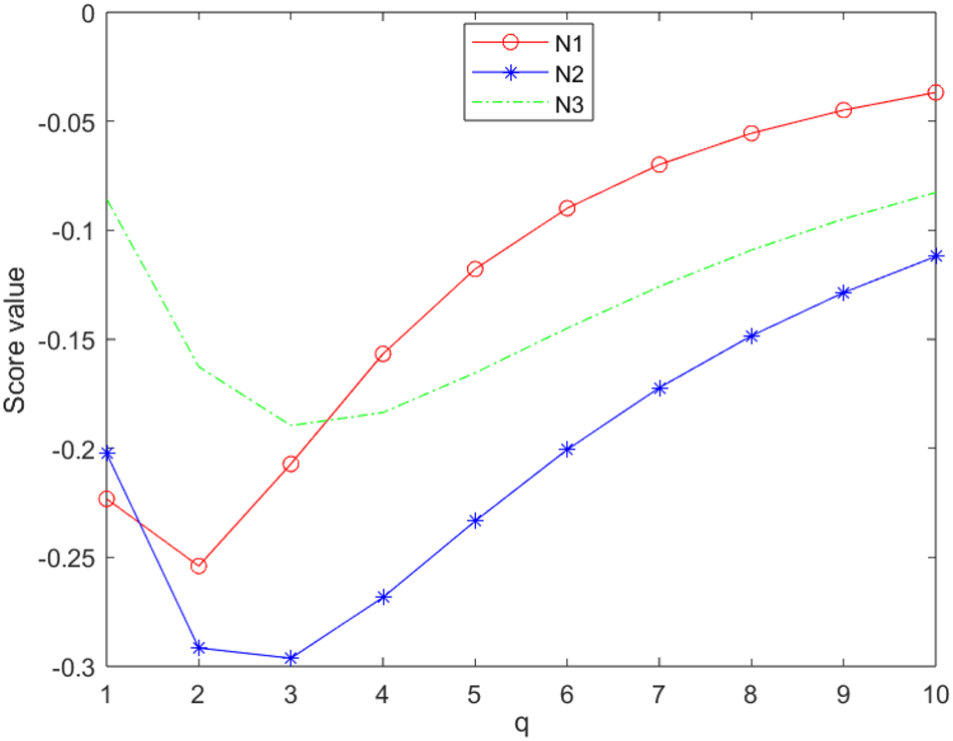
Score values of the alternatives for $1 \le q \le 10$ under Q-ROFHSEWG.

The influence of $q$ on assessment consequences for the q-ROFHSEWG operator is specified in Table 5. Also, the graphical demonstration of the influence of $q$ displayed in Fig. 3.

**Table 5:** Effects of parameter ${\varvec{q}}$ on decision results using Q-ROFHSEWG operator.

Parameter	Score value	Ranking
${\varvec{q}} = 1$	$S\left( {{\mathcal{L}}_{1} } \right)$ = $-$ 0.2232, $S\left( {{\mathcal{L}}_{2} } \right)$ = $-$ 0.2024, $S\left( {{\mathcal{L}}_{3} } \right)$ = $-$ 0.0856	$\aleph^{\left( 3 \right)} > \aleph^{\left( 2 \right)}$ $>$ $\aleph^{\left( 1 \right)}$
${\varvec{q}} = 2$	$S\left( {{\mathcal{L}}_{1} } \right)$ = $-$ 0.2540, $S\left( {{\mathcal{L}}_{2} } \right)$ = $-$ 0.2916, $S\left( {{\mathcal{L}}_{3} } \right)$ = $-$ 0.1626	$\aleph^{\left( 3 \right)} > \aleph^{\left( 1 \right)}$ $>$ $\aleph^{\left( 2 \right)}$
${\varvec{q}} = 3$	$S\left( {{\mathcal{L}}_{1} } \right)$ = $-$ 0.2072, $S\left( {{\mathcal{L}}_{2} } \right)$ = $-$ 0.2963, $S\left( {{\mathcal{L}}_{3} } \right)$ = $-$ 0.1896	$\aleph^{\left( 3 \right)} > \aleph^{\left( 1 \right)}$ $>$ $\aleph^{\left( 2 \right)}$
${\varvec{q}} = 4$	$S\left( {{\mathcal{L}}_{1} } \right)$ = $-$ 0.1567, $S\left( {{\mathcal{L}}_{2} } \right)$ = $-$ 0.2684, $S\left( {{\mathcal{L}}_{3} } \right)$ = $-$ 0.1836	$\aleph^{\left( 1 \right)} > \aleph^{\left( 3 \right)}$ $>$ $\aleph^{\left( 2 \right)}$
${\varvec{q}} = 5$	$S\left( {{\mathcal{L}}_{1} } \right)$ = $-$ 0.1178, $S\left( {{\mathcal{L}}_{2} } \right)$ = $-$ 0.2334, $S\left( {{\mathcal{L}}_{3} } \right)$ = $-$ 0.1654	$\aleph^{\left( 1 \right)} > \aleph^{\left( 3 \right)}$ $>$ $\aleph^{\left( 2 \right)}$
${\varvec{q}} = 6$	$S\left( {{\mathcal{L}}_{1} } \right)$ = $-$ 0.0899, $S\left( {{\mathcal{L}}_{2} } \right)$ = $-$ 0.2006, $S\left( {{\mathcal{L}}_{3} } \right)$ = $-$ 0.1449	$\aleph^{\left( 1 \right)} > \aleph^{\left( 3 \right)}$ $>$ $\aleph^{\left( 2 \right)}$
${\varvec{q}} = 7$	$S\left( {{\mathcal{L}}_{1} } \right)$ = $-$ 0.0699, $S\left( {{\mathcal{L}}_{2} } \right)$ = $-$ 0.1722, $S\left( {{\mathcal{L}}_{3} } \right)$ = $-$ 0.1258	$\aleph^{\left( 1 \right)} > \aleph^{\left( 3 \right)}$ $>$ $\aleph^{\left( 2 \right)}$
${\varvec{q}} = 8$	$S\left( {{\mathcal{L}}_{1} } \right)$ = $-$ 0.0555, $S\left( {{\mathcal{L}}_{2} } \right)$ = $-$ 0.1484, $S\left( {{\mathcal{L}}_{3} } \right)$ = $-$ 0.1091	$\aleph^{\left( 1 \right)} > \aleph^{\left( 3 \right)}$ $>$ $\aleph^{\left( 2 \right)}$
${\varvec{q}} = 9$	$S\left( {{\mathcal{L}}_{1} } \right)$ = $-$ 0.0449, $S\left( {{\mathcal{L}}_{2} } \right)$ = $-$ 0.1286, $S\left( {{\mathcal{L}}_{3} } \right)$ = $-$ 0.0948	$\aleph^{\left( 1 \right)} > \aleph^{\left( 3 \right)}$ $>$ $\aleph^{\left( 2 \right)}$
${\varvec{q}} = 10$	$S\left( {{\mathcal{L}}_{1} } \right)$ = $-$ 0.0368, $S\left( {{\mathcal{L}}_{2} } \right)$ = $-$ 0.1120, $S\left( {{\mathcal{L}}_{3} } \right)$ = $-$ 0.0827	$\aleph^{\left( 1 \right)} > \aleph^{\left( 3 \right)}$ $>$ $\aleph^{\left( 2 \right)}$

## Sensitivity analysis and comparative studies

The subsequent section equates the offered approach and prevailing methodologies to confirm the practicality of the delivered scheme.

### Sensitivity analysis

#### Influence on alternatives rank by the deviancy of the $"q"$ for q-Rofhsewa operator

The organization training guides that the ${\mathrm{\aleph }}^{(3)}$ and ${\mathrm{\aleph }}^{(1)}$ are the optimum and poorest alternates, respectively. It can be observed from Table 4 that there is no variation in the alternatives' ordering while "$q$" is between 1 and 10, which is ${\mathrm{\aleph }}^{(3)}>{\mathrm{\aleph }}^{(2)}>{\mathrm{\aleph }}^{(1)}$. Additionally, it can be identified that as the values of "$q$" is increasing, the score values of the alternatives decrease, which shows that the score values are dependent on the parameter "$q$". Moreover, IFHSS^49^ and PFHSS^52^ fail to deal with the situation in the case of ${\left(MD\right)}^{2}+{\left(NMD\right)}^{2}>1$. It is thought that the method proven in^59^ can designate fuzzy information. However, the parameter ''$q$'' marks the facts-gathering procedure as extra supple. Through this analysis, it has been noticed that a parameter's presentation can make it easier for experts to assess any object. They are advised to take the parameter's value according to their needs.

The scheduled method makes fuzzy information easier to describe and makes it extra pliable to combine facts by factors. When assembling some sequences, numerous amalgam structures of FS are converted into the special detail of q-ROFHSS (see Table 6). The parameter "$q"$ helps experts review any project more generally. Therefore, specialists are advised to choose $"q"$ to get the trend. Over this exploration and evaluation, we resolute that the results achieved from the projected model are more perfect than prevalent models.

**Table 6:** Feature analysis of different models with a proposed model.

	Fuzzy information	MD	NMD	Parameterization	Sub-parameters	Advantages
FS^1^	✓	×	✓	×	×	Deals uncertainty by $MD$
IFS^4^	✓	×	✓	×	×	Deals uncertainty by $MD + NMD > 1$
PFS^15^	✓	×	✓	×	×	Deals uncertainty by $MD$ and $NMD$
q-ROFS^27^	✓	✓	✓	×	×	Deals uncertainty by $\left( {MD} \right)^{2} + \left( {NMD} \right)^{2} > 1$
FSS^34^	✓	✓	×	✓	×	Deals uncertainty by using parametric values of $MD$
IFSS^36^	✓	✓	×	×	×	Deals uncertainty by parametric values of $MD$ and $NMD$;$MD + { }NMD > 1$
PFSS^40^	✓	✓	×	×	×	Deals uncertainty by if $\left( {MD} \right)^{2} + \left( {NMD} \right)^{2} > 1$
q-ROFSS^46^	✓	✓	✓	✓	×	Deals uncertainty by, if $\left( {MD} \right)^{q} + \left( {NMD} \right)^{q} > 1$
IFHSS^49^	✓	✓	✓	✓	✓	Deals uncertainty of multi sub-parameters such as $MD + NMD > 1$
PFHSS^52^	✓	✓	✓	✓	✓	Deals uncertainty of multi sub-parameters $\left( {MD} \right)^{2} + \left( {{\text{NMD}}} \right)^{2} > 1$
q-ROFHSS	✓	✓	✓	✓	✓	Deals uncertainty of multi sub-parameters $\left( {{\text{MD}}} \right)^{{\text{q}}} + \left( {{\text{NMD}}} \right)^{{\text{q}}} > 1$

#### Influence on alternatives rank by the deviancy of the ''$q$'' for q-ROFHSEWG operator

To restrain the impact of “$q$” judgment results, we tried for disparate values of $q$, as an organizational mandate for alternates. ${\mathrm{\aleph }}^{(3)}$ is the most appropriate alternative when $q=1-3$, with two dissimilar ranking; ${\mathrm{\aleph }}^{(3)}>{\mathrm{\aleph }}^{(2)}>{\mathrm{\aleph }}^{(1)}$ and ${\mathrm{\aleph }}^{(3)}>{\mathrm{\aleph }}^{(1)}>{\mathrm{\aleph }}^{(2)}$ But, when $q=$ 4–10, the finest substitutes are somewhat altered, which is $\aleph^{\left( 1 \right)}$ with the classification order of substitutes $\aleph^{\left( 1 \right)} > \aleph^{\left( 3 \right)} > \aleph^{\left( 2 \right)}$. Moreover, it can be observed that as the values of $q$ is increasing, the score values of the alternatives also increase, which shows that the score values depend on the parameter $q$. The graphical description of Table 5 is presented in Fig. 3. The above-presented analysis showed that if we change $^{^{\prime\prime}} q^{^{\prime\prime}} ,$ it will disturb the hierarchical imperative of the alternatives. As a result, professionals can choose the value of $^{^{\prime\prime}} q^{^{\prime\prime}}$ to evaluate the most suitable object. It is decided that specialists desired to deliberate the value of $^{^{\prime\prime}} q^{^{\prime\prime}}$ when the alternative rating is stable.

From this investigation, we observed that the hierarchical order of alternatives is affected by the variation of parameter $q$. In some situations, when information data cannot be dealt with IFHSSs and PFHSSs, q-ROFHSS appears to be a valuable tool to tackle this type of problem. The presentation of a parameter can allow the experts to provide their assessment freely. The restriction they faced in IFHSS and PFHSS was eliminated because of the parameter. As a result, experts can choose the suitable value of $q$ to evaluate the target for this delivery. Experts must deliberate the parameter values when ordering the superlative alternatives in a secure situation. In the above example, by using the q-ROFHSEWA operator, the ranking order is the same as $\ge 1$. Here the value of $q$ can be chosen from one and above. But when we apply the q-ROFHSEWG operator, the ranking order is stable when $q \ge 4$. In this situation, the experts can choose a value of 4 or above.

### Superiority of the planned technique

The proposed scheme is talented and substantial. We settled an innovative MCGDM approach by q-ROFHSEWA and q-ROFHSEWG operators. The developed methodology in this research is more extraordinary than prevalent methods and compatibility contracts with MCGDM problems. The provision methodology is versatile and familiar, with disparities, accountabilities, and changes allowing for different outputs. Unlike models with explicit taxonomic comportment, there is a conventional alteration to the projected scheme classification to encounter its perspective. Methodological studies and estimations consider that the consequences accomplished from prevalent approaches are similar to hybrid substances. Also, after adding some suitable conditions, numerous amalgam configurations of FS become the q-ROFHSS. Adding infrequent and blurred facts to the current practical plan is unexpected. In this, data about prosperity can be described more completely and reasonably. Through the DM procedure, fabricated and troubling details are miscellaneous together. So, our proposed methodology will be extra dedicated, significant, superior, and enhanced than several amalgam FS sceneries. Table 6 presents the feature analysis of our developed and prevalent approaches.

### Comparative analysis

To demonstrate the capability of the established organization, we linked the inferences gained from some well-known systems. Table 7 summarizes the comparison between our developed model and existing AOs. The AOs PFSEWA^43^, PFSEWG^43^, PFSEOWA^44^, PFSEOWG^45^, q-ROFSWA^46^, q-ROFSOWA^46^, q-ROFSWG^72^, q-ROFSOWG^72^, q-ROFSEWA^47^, q-ROFSEOWA^47^, q-ROFSEWG^48^, and q-ROFSEOWG^48^ are used to analyze the parametric values of the substitute. These AOs are unable to deal with the sub-attributes of the deliberated parameters. Meanwhile, the AOs presented in^51^ under the IFHSS environment can diminish with the sub-parameters of substitutes. However, these AOs fail to deal with the decision outcomes when the sum of ${\text{MD}} + {\text{NMD}} > 1$. Sunthrayuth et al.^54^ and Zulqarnain et al.^55^ prolonged the Einstein weighted average and geometric AOs for PFHSS and confirmed the novel MCDM techniques to solve MCDM obstacles because of the parameterization of sub-attributes. But these AOs also flop when the $\left( {{\text{MD}}} \right)^{2} + \left( {{\text{NMD}}} \right)^{2} > 1$. Khan et al.^59^ prolonged the algebraic operational laws and AOs for q-ROFHSS to compact the above hurdles. However, these AOs cannot carry the desirable outcomes in some situations. So, to solve these composite troubles, we introduce Einstein's weighted AOs for q-ROFHSS. It is an appropriate extension of a q-ROFSS and a generalized form of PFHSS. From the above facts, it will be claimed that the proposed AOs are competent, reliable, and prosperous compared to prevalent AOs. The comparison between the developed AOs and some usual AOs is explored in Table 7.

**Table 7:** Comparative analysis with existing operators.

Method	$\aleph^{\left( 1 \right)}$	$\aleph^{\left( 2 \right)}$	$\aleph^{\left( 3 \right)}$	Ranking order
PFSEWA^43^	0.3287	0.2634	0.4532	$\aleph^{\left( 3 \right)} { } > \aleph^{\left( 1 \right)} > \aleph^{\left( 2 \right)}$
PFSEWG^43^	0.2924	0.2418	0.3726	$\aleph^{\left( 3 \right)} > \aleph^{\left( 1 \right)} > \aleph^{\left( 2 \right)}$
PFSEOWA^44^	0.4105	0.4156	0.4281	$\aleph^{\left( 3 \right)} > \aleph^{\left( 2 \right)} > \aleph^{\left( 1 \right)}$
PFSEOWG^45^	0.3951	0.3849	0.4083	$\aleph^{\left( 3 \right)} > \aleph^{\left( 1 \right)} > \aleph^{\left( 2 \right)}$
IFHSWA^51^	0.3894	0.4071	0.4712	$\aleph^{\left( 3 \right)} { } > \aleph^{\left( 2 \right)} > \aleph^{\left( 1 \right)}$
IFHSWG^51^	0.3123	0.4436	0.4927	$\aleph^{\left( 3 \right)} > \aleph^{\left( 2 \right)} > \aleph^{\left( 1 \right)}$
PFHSEWA^54^	0.1959	0.2426	0.2763	$\aleph^{\left( 3 \right)} > \aleph^{\left( 2 \right)} > \aleph^{\left( 1 \right)}$
PFHSEWG^55^	− 0.0264	− 0.0217	− 0.0157	$\aleph^{\left( 3 \right)} > \aleph^{\left( 2 \right)} > \aleph^{\left( 1 \right)}$
q-ROFSWA^46^	0.4194	0.4375	0.4463	$\aleph^{\left( 3 \right)} > \aleph^{\left( 2 \right)} > \aleph^{\left( 1 \right)}$
q-ROFSOWA^46^	0.2964	0.3159	0.3571	$\aleph^{\left( 3 \right)} > \aleph^{\left( 2 \right)} > \aleph^{\left( 1 \right)}$
q-ROFSWG^72^	0.3493	0.4048	0.4648	$\aleph^{\left( 3 \right)} > \aleph^{\left( 2 \right)} > \aleph^{\left( 1 \right)}$
q-ROFSOWG^72^	0.3601	0.4132	0.4676	$\aleph^{\left( 3 \right)} > \aleph^{\left( 2 \right)} > \aleph^{\left( 1 \right)}$
q-ROFSEWA^47^	0.4059	0.4567	0.5143	$\aleph^{\left( 3 \right)} > \aleph^{\left( 2 \right)} > \aleph^{\left( 1 \right)}$
q-ROFSEOWA^47^	0.4367	0.4638	0.5338	$\aleph^{\left( 3 \right)} > \aleph^{\left( 2 \right)} > \aleph^{\left( 1 \right)}$
q-ROFSEWG^48^	0.4158	0.4307	0.4942	$\aleph^{\left( 3 \right)} > \aleph^{\left( 2 \right)} > \aleph^{\left( 1 \right)}$
q-ROFSEOWG^48^	0.4251	0.4467	0.5138	$\aleph^{\left( 3 \right)} > \aleph^{\left( 2 \right)} > \aleph^{\left( 1 \right)}$
q-ROFHSWA^59^	0.0125	0.0187	0.0247	$\aleph^{\left( 3 \right)} > \aleph^{\left( 2 \right)} > \aleph^{\left( 1 \right)}$
q-ROFHSWG^59^	− 0.0263	− 0.0157	− 0.0104	$\aleph^{\left( 3 \right)} > \aleph^{\left( 2 \right)} > \aleph^{\left( 1 \right)}$
q-ROFHSEWA	0.0574	0.0815	0.2181	$\aleph^{\left( 3 \right)} > \aleph^{\left( 2 \right)} > \aleph^{\left( 1 \right)}$
q-ROFHSEWG	− 0.2070	− 0.2963	− 0.1896	$\aleph^{\left( 3 \right)} > \aleph^{\left( 1 \right)} > \aleph^{\left( 2 \right)}$

**Figure 4:**
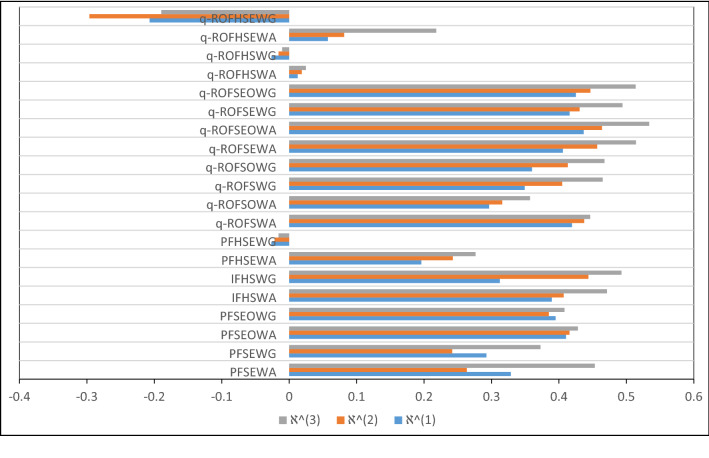
Comparative analysis.

Therefore, we have the right to be amazed by the exploitation and unreliability of the DM procedure for the prevailing operators we have recognized. Intentional sustenance for this method-related action has a slight influence on adverse reasons. In this way, it relaxes the organization of unreliable and assumed details in the amplification of DM. Figure 4 parades the graphical demonstration of the comparison analysis.

### Advantages of the proposed research

In this section, we will discuss the advantages of the structured approach proposed in this study.

- The structured approach combines the concept of parametrization with q-ROFHSS to assess the impact of DM constraints. The constant parametrization of MD and NMD simulates the possibility of designation and degree of validity. This correspondence enables the calculation of practical demonstrations in an interpolated universe with these features. By using this approach, decision-makers can easily understand and analyze the impact of various parameters on the DM process.
- The model emphasizes a comprehensive examination of the parameter values and their associated subparameters. This supports decision-makers in DM labeling combinations and making reliable decisions. The structured approach provides a more detailed and accurate representation of the DM process, enabling decision-makers to make informed decisions with a high degree of confidence.

This approach confirms all forms and properties of the significant theory and is not considered a general system of existing approaches. By combining the principles of parametrization and q-ROFHSS, this approach provides a unique and powerful tool for decision-makers in a variety of settings.

## Conclusion

The lack of contemplation on abstruse conditions in the features can obstruct some of the complex implications of MCGDM. The mathematical model in MCGDM achievements all special possessions while fascinating intent under the limits of finance, superiority, and welfare boundaries. It is necessary to limit the investigation to make decisions at the highest level and capture the need for decisions. In factual DM, estimates of alternative details recognized by professionals are often inaccurate, asymmetrical, and insignificant, so q-ROFHSNs can be used to calculate these defective facts. The fundamental impartiality of this purpose is to perform the Einstein operational laws for q-ROFHSS. We proposed q-ROFHSEWA and q-ROFHSEWG operators with their ideal possessions. In addition, the DM approach is planned to solve MCGDM bottlenecks based on proven operators. To illustrate the strength of the presented method, we convey a comprehensive mathematical description of the most appropriate construction firm. Finally, based on the results obtained, it is undeniable that the scheme offered in this study is the most realistic and feasible approach to illuminate the MCGDM problem. Future investigation will focus on defining Einstein-ordered AOs, distance, and similarity measures with their conforming characteristics. Moreover, it can be extend to interval valued q-ROFHSS with fundamental operations and numerous AOs with their DM methodologies. We can also integrate q-ROFHSNs with other MCGDM methods and further engage in practical application in matters of medical diagnosis, material selection, pattern recognition, information fusion, and supply chain management. Also, several topological, algebraic, and ordered structures can be present for q-ROFHSNs with their DM methodologies.

## Data Availability

The datasets used during the current study available from the corresponding author on reasonable request.
